# Role of epigenetics in the clinical evolution of COVID-19 disease. Epigenome-wide association study identifies markers of severe outcome

**DOI:** 10.1186/s40001-023-01032-7

**Published:** 2023-02-17

**Authors:** Luciano Calzari, Lucia Zanotti, Elvira Inglese, Francesco Scaglione, Rebecca Cavagnola, Francesco Ranucci, Anna Maria Di Blasio, Giulio Stefanini, Gaetano Carlo, Gianfranco Parati, Davide Gentilini

**Affiliations:** 1 Bioinformatics and Statistical Genomics Unit, IRCCS Istituto Auxologico Italiano, Cusano Milanino, Milan, Italy; 2 Sleep Disorders Center & Department of Cardiovascular, Neural and Metabolic Sciences, IRCCS Istituto Auxologico Italiano, San Luca Hospital, Milan, Italy; 3 Department of Brain and Behavioral Sciences, University of Pavia, Via Bassi 21, Pavia, Italy; 4 Chemical-Clinical Analysis Unit, ASST Grande Ospedale Metropolitano Niguarda, Milan, Italy; 5 Department of Oncology and Hemato-Oncology, University of Milan, Milan, Italy; 6 Molecular Biology Laboratory, IRCCS Istituto Auxologico Italiano, Cusano Milanino, Milan, Italy; 7 Department of Biomedical Sciences, Humanitas University, Pieve Emanuele-Milan, Italy; 8 IRCCS Humanitas Research Hospital, Rozzano-Milan, Italy; 9 Laboratorio di Epigenetica, Istituti Clinici Scientifici Maugeri IRCCS, Via Maugeri 4, 27100 Pavia, Italy; 10 Department of Medicine and Surgery, University of Milan‐Bicocca, Milan, Italy

**Keywords:** SARS-CoV-2, COVID-19, EWAS, DNA methylation, Epigenetics, COVID signature, Stochastic epigenetic mutation, Epigenetic drift

## Abstract

**Background:**

COVID-19 has a wide spectrum of clinical manifestations and given its impact on morbidity and mortality, there is an unmet medical need to discover endogenous cellular and molecular biomarkers that predict the expected clinical course of the disease. Recently, epigenetics and especially DNA methylation have been pointed out as a promising tool for outcome prediction in several diseases.

**Methods and results:**

Using the Illumina Infinium Methylation EPIC BeadChip850K, we investigated genome-wide differences in DNA methylation in an Italian Cohort of patients with comorbidities and compared severe (*n* = 64) and mild (123) prognosis. Results showed that the epigenetic signature, already present at the time of Hospital admission, can significantly predict risk of severe outcomes. Further analyses provided evidence of an association between age acceleration and a severe prognosis after COVID-19 infection. The burden of Stochastic Epigenetic Mutation (SEMs) has been significantly increased in patients with poor prognosis. Results have been replicated in silico considering COVID-19 negative subjects and available previously published datasets.

**Conclusions:**

Using original methylation data and taking advantage of already published datasets, we confirmed in the blood that epigenetics is actively involved in immune response after COVID-19 infection, allowing the identification of a specific signature able to discriminate the disease evolution. Furthermore, the study showed that epigenetic drift and age acceleration are associated with severe prognosis. All these findings prove that host epigenetics undergoes notable and specific rearrangements to respond to COVID-19 infection which can be used for a personalized, timely, and targeted management of COVID-19 patients during the first stages of hospitalization.

**Supplementary Information:**

The online version contains supplementary material available at 10.1186/s40001-023-01032-7.

## Background

The severe acute respiratory syndrome coronavirus 2 (SARS-CoV-2) virus was identified in Wuhan, China, in late 2019 and has provoked an ongoing global pandemic of the resulting illness, COVID-19. COVID-19 has a broad spectrum of clinical manifestations, with most infected subjects showing only mild symptoms or being asymptomatic [1, 2]. The leading group of patients with high mortality rates comprises those with severe respiratory failure associated with acute respiratory distress syndrome (ARDS) and interstitial pneumonia: these high-risk patients require early and prolonged support by mechanical ventilation to compensate for their respiratory failure [3, 4]. The reasons for the heterogeneous clinical repertoire of COVID-19 are mainly unknown. Only three risk factors have been consistently related to life-threatening COVID-19-associated respiratory failure: male sex, old age and concomitant medical conditions, such as diabetes, obesity, hypertension and cardiovascular pathology [5, 6]. However, despite all these components, there is significant inter-individual variability in each demographic and epidemiological group. Thus, given the immense impact of COVID-19 on morbidity and mortality, there is an unmet medical need to discover endogenous cellular and molecular biomarkers that predict the expected clinical course of the disease.

Based on this critical lack of knowledge regarding the molecular mechanism underlying COVID-19 infection response, recent epigenome-wide association studies (EWAS) explored a particular layer of biological information: the impact of epigenetic variation and in particular DNA methylation in establishing a severe clinical course of COVID-19 disease [7–11]. These studies demonstrated that epigenetics plays a central role in the progression of COVID-19 through the identification of specific methylation signatures associated with the severe clinical evolution of the infection. However, there is only a partial overlap between their results: this dissimilarity is due to different choices in study design. For example, the main differences concern the number of samples analyzed, the inclusion criteria used to enroll patients, and the bioinformatics methods/strategies adopted to analyze data. Bernardes and colleagues [8] performed a longitudinal multi-omics approach but focused on the DNA methylation profile of a limited number of patients. Also, Zhou et al. analyzed a small cohort of patients stratifying the methylation cohort into three groups: mild and severe COVID-19 patients and healthy subjects. Balnis and colleagues [7] improved the number of patients and compared mild (non-ICU) and severe (ICU admitted) vs healthy subjects obtaining a signature of 77 differentially methylated positions associated with the degree of severity of COVID-19. Konigsberg et al. [10] compared the methylation status of three groups of patients: SARS-CoV-2-Positive, SARS-CoV-2-Negative, and subjects with other respiratory infections. In Castro de Moura et al. [9], only young patients without comorbidities were enrolled. These studies shed light on different epigenetic aspects underlying response to SARS-CoV-2 infection, however, mainly not targeting high-risk patients. There is still a lack of knowledge regarding the role of epigenetics in characterizing severe outcomes in this specific patient group.

Based on these considerations, we conducted a genome-wide study using the Illumina 850 K Beadchip on 190 blood samples from Italian COVID-19 patients who were at high risk for comorbidities and clinical factors. The goal of this study was to identify epigenetic biomarkers that could predict the clinical prognosis of these patients and provide insights into the role of epigenetic mechanisms in the evolution of COVID-19 severity.

## Results

### Description of patients

A total of 190 individuals (124 mild and 66 severe) were initially processed. After the quality control step performed both at probe and sample level a total of 187 subjects (123 classified as mild and 64 classified as severe) were considered for the analysis. The available clinical data of these patients are summarized in Table 1 (detailed information is provided in Additional File 1).

**Table 1:** Classification of clinical characteristics of the COVID-19 cohorts

Characteristics	Mild (References) Cohort	Severe (Cases) Cohort	*p*-value
	*N* = 123	*N* = 64	
Host factors			
Age (years)—Median (IQR)	62 (21–90)	66.8 (30–95)	> 0.05
Gender—(Male/Female)	1.5	2.5	> 0.05
ICU admission	0.0	68.0%	–
Death	0.0	48.0%	–
Smoke	21.0%	14.0%	> 0.05
Fever(> 37.3 °C)	24.0%	40.0%	0.03
Comorbilities			
Obesity	13.0%	14.0%	> 0.05
Hypertension	51.2%	67.1%	> 0.05
Diabetes	16.2%	50.0%	2.4 e-6
Cardiovascular/Coronary heart disease	38.2%	32.8%	> 0.05
Respiratory disease	14.6%	23.4%	> 0.05
Cerebrovascular disease	5.6%	7.8%	> 0.05
History of cancer	12.1%	3.1%	> 0.05
No comorbility	17.8%	6.5%	> 0.05
Biochemical parameters			
Elevated creatinine (> 1.33 mg/dL)	4.0%	7.0%	> 0.05
Reduced albumine (< 4 g/dL)	82.2%	92.3%	> 0.05
Elevated AsT (> 40 U/L)	26.6%	53.8%	> 0.05
Elevated LDH (> 245 mU/ml)	60.0%	76.9%	> 0.05
Elevated C reactive protein (> 8.2 mg/L)	31.0%	61.5%	> 0.05
Elevated D-dimer (> 1000 ng/mL)	48.8%	69.2%	> 0.05
Elevated white cell count (> 4 × 10^9^/L)	0.0%	0.0%	> 0.05

Considering the whole cohort males resulted more represented than females (63.6% males vs. 36.4% females). The two groups showed slight but not significant differences in the male/female ratio (Fisher’s exact test, *p* = 0.109). No significant differences in age were also observed between the two cohorts (Mann–Whitney test, *p* = 0.2). Severe patients who died after admission to ICU were 11 (17.2%). Taking into account the presence of comorbidities, 88% of subjects had at least one pathological condition; however, only diabetes showed a significant difference between severe and mild groups (*p*-value = 2.4 × 10^–6^).

### Sample group-level differential methylation analysis

**Fig. 1:**
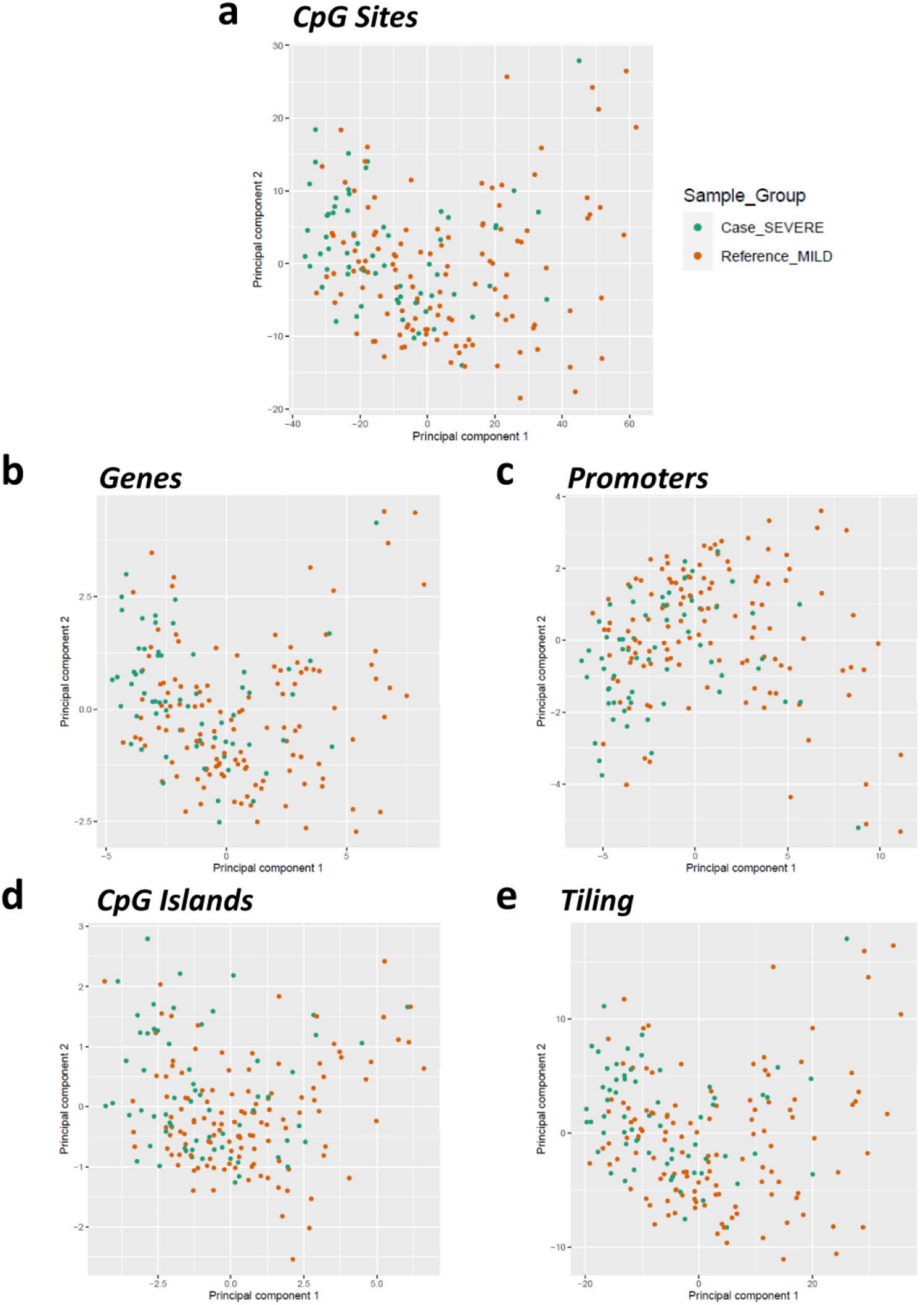
Scatter plots of principal component analysis (PCA). Scatter plot distribution of samples along with the first two principal components at **a** sites, **b** genes, **c** promoters, **d** CpG islands, and e tiling (5 kb fixed regions)

A sample group-level analysis was conducted using RnBeads. The quality control and data preparation are described in the methods section. To reduce the complexity of the data, a principal component analysis (PCA) was performed at different levels: CpG sites and regions (such as genes, promoters, CpG islands, and tiling). This analysis was used to identify macro-variations in the epigenetic characteristics of the sample groups. We did not observe a strong separation between the two groups for any considered region (variance explained: Sites: PC1 = 34.5%, PC2 = 4.8%; Genes: PC1 = 31.1%, PC2 = 6.9%; Promoters: PC1 = 33.9%, PC2 = 6.4%; CpG Islands: PC1 = 21.6%, PC2 = 3.6%; Tiling: PC1 = 37.3%, PC2 = 6.1%) (Fig. 1).

**Fig. 2:**
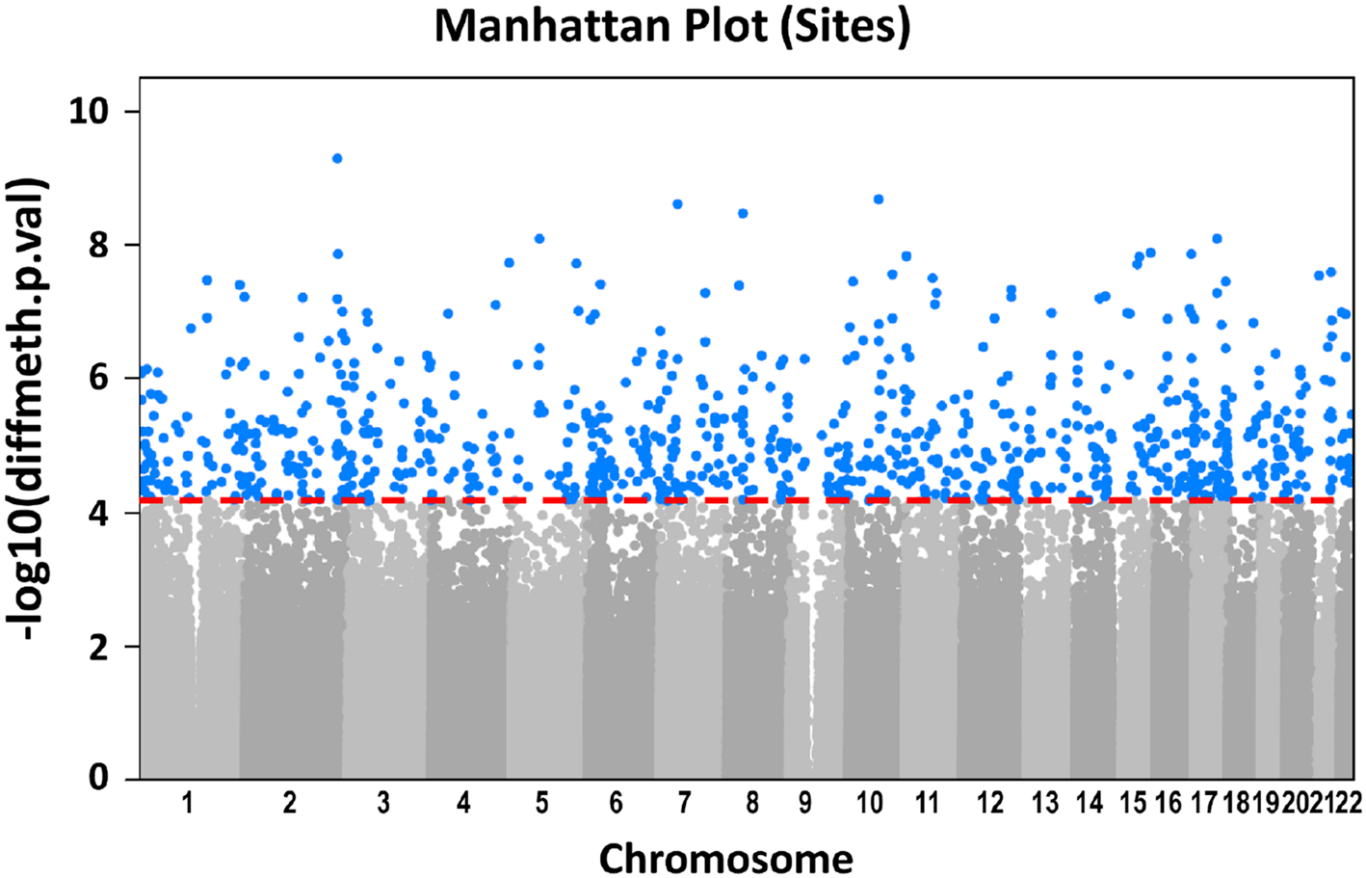
Manhattan Plot. Manhattan plot showing the distribution of *p*-values of differentially methylated CpG sites. The ordinate axis represents the negative log10 of the unadjusted *p*-value of methylation differences between “severe” and control “mild” groups while the abscissa axis is the location of differentially methylated points concerning chromosomes. Dots in blue color represent the 880 significant differentially methylated CpG sites. The dashed red line represents the threshold of significance (False Discovery Rate)

Differential methylation analysis was conducted at site level adjusting for potential confounding factors. We performed a PCA considering critical confounding variables such as age and cellular components estimation (obtained from Steve Horvath’s Epigenetic Clock) [12]. Among the first 9 PCs capturing 90% of the variance, only PC1 resulted associated with the disease outcome (*p* = 4.59e-07) and was considered as covariate in the differential methylation analysis. After multiple testing correction a list of 880 probes resulted differentially methylated: 448 were hyper-methylated, while the remaining 432 were hypo-methylated. (Fig. 2) (the list of significant differentially methylated CpG sites is provided in Additional File 2).

The functional annotation of 880 CpG sites identified several genes that are involved in immune response and related biological processes and pathways. These include SAMHD1, SETD2, IRF2, IL12B, TRIM8, NLRP3, MAPK10, and PIK3CD, which are found within hyper-methylated genes while, ARID5A, CD226, CD244, IL1R1, STAT6 were annotated from hypo-methylated CpG sites (Additional File 3).

No significant genome-wide results emerged from the analysis conducted at the regional level considering gene, promoters, CpG islands, tiling. Therefore, a prioritization approach through Over Representation Analysis (ORA) and Gene Set Enrichment Analysis (GSEA) was adopted considering the first 100 best ranked differentially methylated genes. Concerning hyper-methylated positions, the ORA revealed a significant over representation of immune-related biological processes, including, for example, immune system process (GO:0002376; OR = 13, adj.*p*-value < 0.05), regulation of T cell proliferation, activation, selection (GO:0046640; GO:0050863; GO:0045058; adj.*p*-value < 0.05), negative regulation of viral genome replication (GO:0045071), regulation of leukocyte activation (GO:0002694), positive regulation of interferon-gamma production (GO:0032729). As regards the hypo-methylated positions, the over-representation analysis produced very heterogeneous results, comprising suggestive processes such as immune (GO: 0002523) or anti-inflammatory responses (GO: 0050727) (the list of enriched gene ontology terms is provided in Additional File 4). Top 100 ranked differentially methylated genes were furtherly investigated through Gene Set Enrichment Analysis (GSEA) [13, 14]: results showed enrichment of immune-related terms including “adaptative immune response” or “response to virus” (among “hyper-methylated” terms) and “regulation of inflammatory response” (among “hypo-methylated” terms).

### Epi-signature analysis

**Fig. 3:**
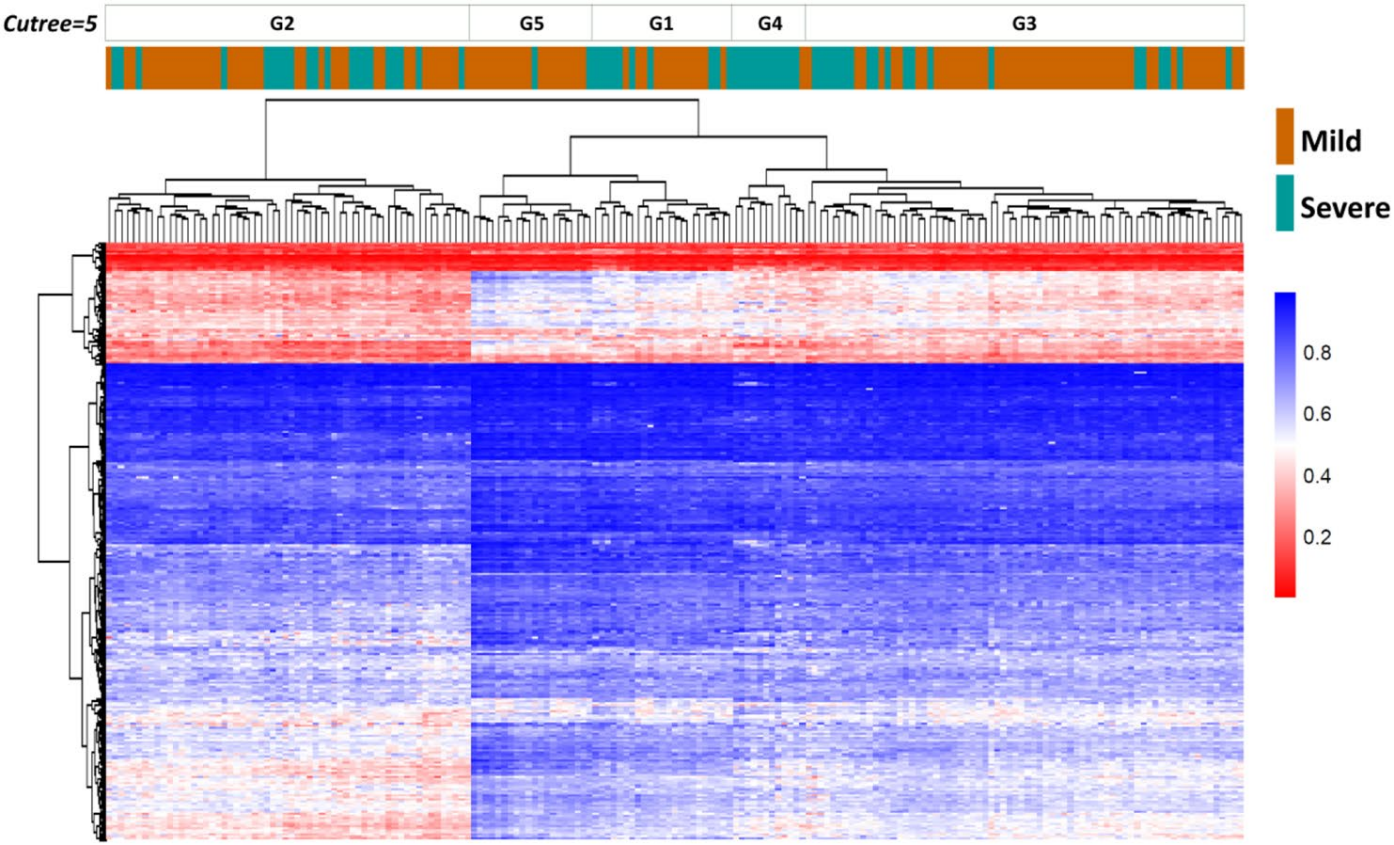
Unsupervised hierarchical clustering. Heatmap showing unsupervised hierarchical clustering of 187 samples using the 880 differentially methylated CpG sites obtained in differential methylation analysis (RnBeads). The blue color indicates hyper-methylation while red indicates hypo-methylation. Green bars represent “severe” COVID-19 patients. Orange bars represent “mild” patients (used as controls). Cluster analysis was performed using the “complete” clustering method and assuming Euclidean distances

The list of 880 CpG sites was then used for unsupervised hierarchical clustering and the resulting dendrogram was analyzed to identify subgroups that may be associated with disease evolution. As shown in Fig. 3, the clustering signature was not able to fully distinguish severe patients from mild samples. However, when the dendrogram was partitioned into progressively increasing numbers of clusters, a group (G4) resulted strongly enriched in severe patients (11 out of 12 patients, or 92% of the group). This association was statistically significant (*p*-value = 3.4 × 10^–5^) according to a hypergeometric distribution test (Fig. 3).

**Fig. 4:**
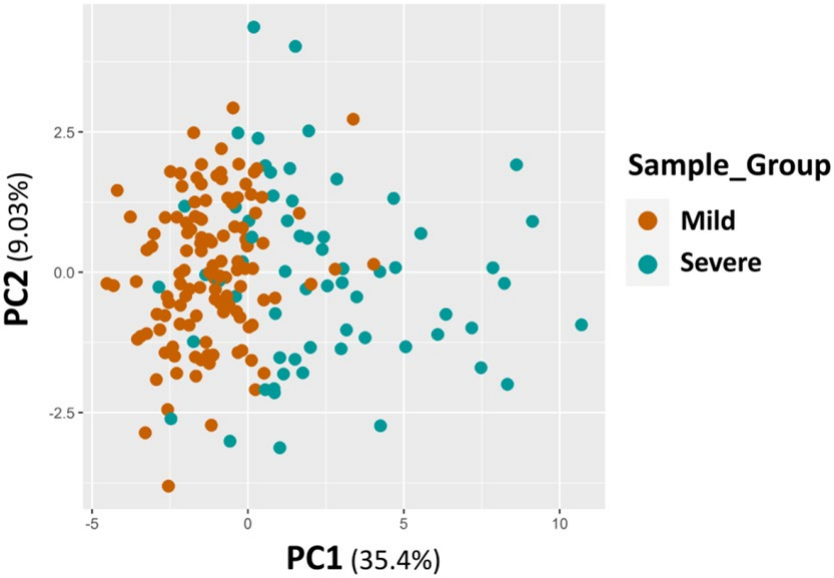
Scatter plots of principal component analysis (PCA) on 21 Differentially methylated sites. Scatter plot distribution of the methylation profiles of 187 samples restricted to the 21 CpG sites constituting our COVID-19 signature

Starting from the significant enrichment of cluster 4 in patients with severe outcomes, we tried to identify the differentially methylated probes with the highest predictive power. We then compared the 5 clusters to each other to select the markers that were differentially methylated and specific to cluster 4. The analysis resulted in a list of 21 CpG sites, which were summarized using PCA to obtain a principal component that could distinguish between severe and mild patients. This information is presented in Fig. 4.

The first principal component (PC1) captured a high percentage of variation (35.4%) and was able to distinguish the two groups showing a significant association with severity (OR = 2.55 (95% CI 1.9–3.5)). Furthermore, both severe and mild groups were compared with a cohort of Covid negative subjects. The results showed that the 21 CpG epi-signature was still able to distinguish between patients with severe outcomes and COVID-negative subjects [OR = 2.3 (95% CI 1.78–3.17)]. Conversely, the epi-signature was not able to differentiate patients with mild outcomes from COVID-negative subjects. Results are reported in Table 2.

**Table 2:** Logistic regression analysis by considering the 21 CpGs signature

Dataset	Number of samples	Variable	Coef	Std.Err	*p*-value	OR	(CI)		Sig
This study									
Covid + mild (123) vs Severe (64)	*n* = 187	PC1	0.94	0.149	3.3E-10	2.55	1.96	3.53	***
Covid + mild (123) vs Covid- (75)	*n* = 198	PC1	0.11	0.074	0.11	1.12	0.97	1.30	ns
Covid + severe (64) vs Covid- (75)	*n* = 139	PC1	0.83	0.146	1.1E-08	2.3	1.78	3.17	***
GSE167202 (*Konigsberg I.R. *et al*. 2021)*									
Covid + Severe (48) vs mild (115)	*n* = 163	PC1	0.33	0.088	1.16E-04	1.4	1.19	1.68	***
GSE174818 *(Balnis J. *et al*. 2021)*									
Covid + Severe (55) vs mild (45)	*n* = 100	PC1	0.52	0.128	3.75E-05	1.69	1.34	2.23	***

*Coef.* logistic coefficients, *Std.Err.* Standard Error, *OR* Odds Ratio, *CI* Confidence Interval, *Sig.* Statistical Significance

^***^p <  < 0.01

The 21 CpG site epi-signature was also validated using additional replication cohorts from published datasets available in the Gene Expression Omnibus (GEO) repository. We took advantage of two datasets (GSE167202 [10] and GSE174818 [7]) and found that the results were consistent with those obtained in this study (Table 2). In both datasets, the logistic regression model demonstrated that the epi-signature was significantly associated with severe outcomes (PCA scatter plots are available in Additional File 4).

Finally, we evaluated the effectiveness of the epi-signature when considering various clinical factors including (i) host characteristics, (ii) comorbidities, (iii) biochemical measurements and accounting also for pharmacological treatments. We compared the results of the three models obtained with and without the inclusion of the epi-signature. The results showed that the models that included the epi-signature were always significantly more predictive, explaining a higher proportion of the variance in severe outcomes. More specifically, when we included the epi-signature in the model that took into account host factors, the McFadden’s R-Squared model goodness index increased from 0.06 to 0.37. Similarly, in the model that considered comorbidities, the McFadden’s R-Squared model goodness index increased from 0.11 to 0.37, and in the model that considered biochemical values, it increased from 0.15 to 0.37. Pharmacologic treatments were considered as potential confounders and included in the regression model, however, results excluded a relation between treatments and the epi-signature.

### GrimAge epigenetic clock

**Fig. 5:**
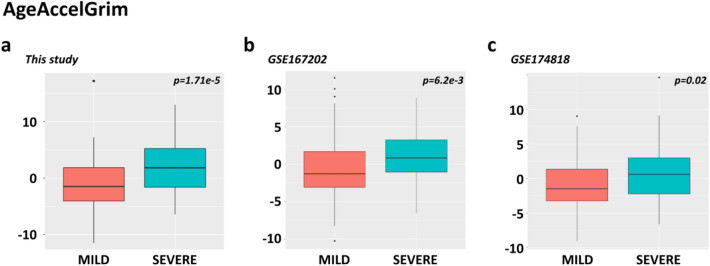
Boxplots of GrimAge epigenetic clock. Boxplots showing the distribution of AgeAccelGrim measures in “mild” and “severe” patients in **a** our study, **b** GSE167202 [10], and **c** GSE174818 [7]. The thick horizontal line in the box represents the median of the distribution while the box represents the interquartile range. Whiskers are set as the default option for the “ggplot” boxplot function and extend to the most extreme data point, which is no more than 1.5 times the interquartile range from the box. Dots represent outliers (single values exceeding 1.5 interquartile ranges)

To better understand the role of epigenetics in the progression of COVID-19, we examined age acceleration using two epigenetic clocks developed by Horvath: AgeAccelerationDiff, which measures the difference between chronological age and epigenetic age, and GrimAge, an epigenetic clock known for its excellent predictive ability when it comes to survival. As shown in the Additional File 4, we did not find significant differences between the mild and severe groups in terms of AgeAccelerationDiff (Mann–Whitney test, *p* = 0.96), and this result was also supported by the replication datasets GSE167202 [10] (Mann–Whitney test, *p* = 0.67) and GSE174818 [7] (Mann–Whitney test, *p* = 0.107) (Boxplots are reported in the Additional File 4). However, significant differences between the two groups emerged when using the GrimAge clock. Specifically, the group of patients with severe outcomes showed significantly higher GrimAge values compared to the mild group (Mann–Whitney test; *p*-value = 1.71 × 10^–5^) (Fig. 5a).

Albeit with different significance, this trend was also noted in the two validation datasets: GSE167202 (Mann–Whitney test, *p* = 0.00625) and GSE174818 (Mann–Whitney test, *p* = 0.02) (Fig. 5b, c, respectively). Combining the statistical significance of AgeAccelGrim differences between the three studies, a Fisher’s combined Benjamini–Hochberg adjusted *p*-value equal to 4.67 × 10^–7^ was obtained.

### Stochastic epigenetic mutations (SEMs)

Epigenetic drift was investigated by analyzing the burden of Stochastic Epigenetic Mutations (SEMs) [15–18]. A SEM at a specific CpG site was defined as an extreme outlier in the distribution of DNA methylation values across individuals. To identify SEMs, the distribution and variability of methylation levels were first studied in control populations for all the probes. A reference interval for methylation levels was then calculated for each probe using the formula Q1-(3 × IQR) and Q3 + (3 × IQR). Any extreme outliers, with methylation levels outside this interval, were identified as SEMs. Finally, for each subject, all the SEMs were recorded in a new data matrix, including whether they were hyper-methylated or hypo-methylated. The method used for this analysis is described in more detail in the materials and methods section.

**Fig. 6:**
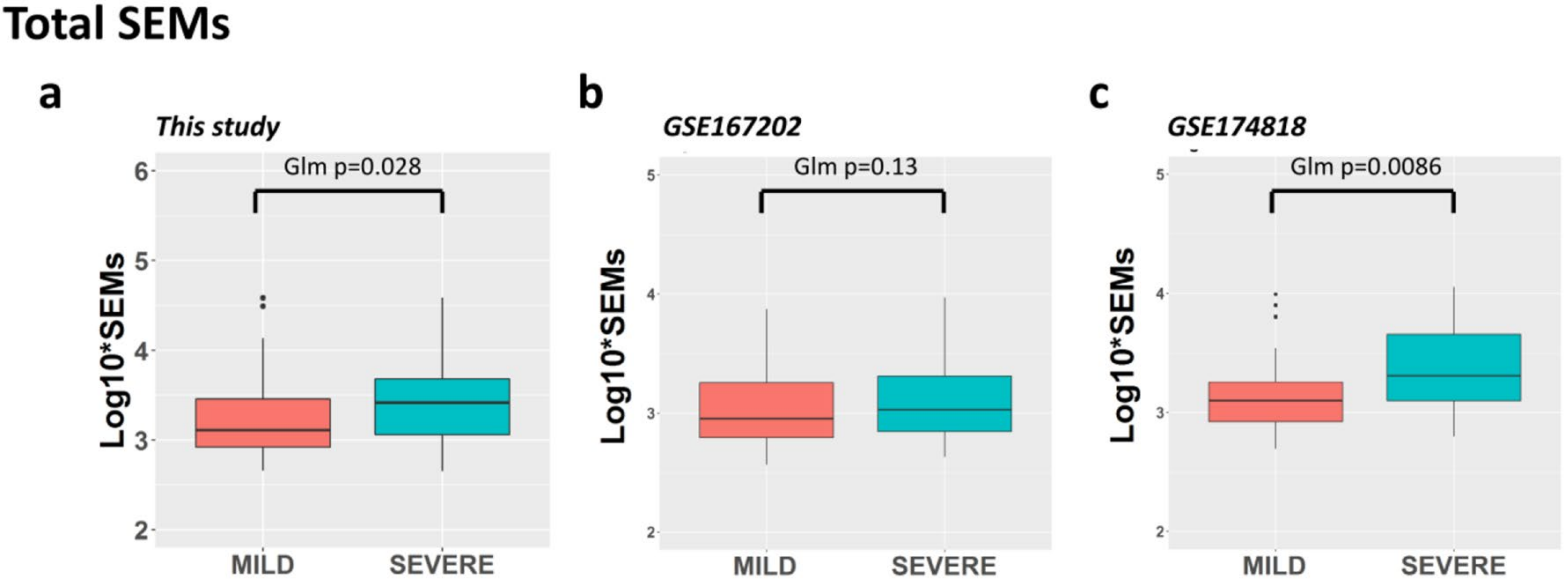
Boxplots of Stochastic Epigenetic Mutations (SEMs). Boxplots showing the distribution of SEMs in “mild” and “severe” patients in **a** our study, **b** GSE167202, and **c** GSE174818. The left panel shows the non-transformed SEMs values while in the panel on the right the log10 transformed SEMs values. The thick horizontal line in the box represents the median of the distribution while the box represents the interquartile range. Whiskers are the default option for the “ggplot2” boxplot function and extend to the most extreme data point, which is no more than 1.5 times the interquartile range from the box. Dots represent outliers (single values exceeding 1.5 interquartile ranges)

The burden of SEMs in the severe group was found to be statistically higher than in the mild group. The median burden of SEMs in the severe group was 2600 (IQR: 1148–4808) while the median burden in the mild group was 1290 (IQR: 830.5–2875). A multiple regression model that took into account sex and age as covariates confirmed that this difference was statistically significant (*p* = 0.0281) (Fig. 6a).

The trend seen in the original dataset was also present in the two replication datasets, but it was only statistically significant in one of them (GSE174818, *p* = 0.0086). In the other dataset (GSE167202), the trend was not statistically significant (*p* = 0.13). Fisher’s method was then used to analyze the combined results of all three studies and assess their overall statistical significance. This analysis yielded an adjusted *p*-value of 0.002.

The impact of epigenetic drift was studied in more detail by dividing the SEMs into those that were hypermethylated and those that were hypomethylated. The trend of differences between the groups was still evident in the lists of hypermethylated SEMs, but statistical differences were mainly found in the lists of hypomethylated SEMs (Boxplots illustrating these results can be found in Additional File 4).

## Discussion

In this study, we investigated epigenetic differences that may play a role in the development of severe COVID-19 in a group of high-risk Italian patients (i.e., with high prevalence of comorbidities). We identified a group of 21 epigenetic markers that were able to predict the risk of severe outcomes, such as death or the need for mechanical ventilation in the intensive care unit. To confirm the validity of our findings, we also analyzed publicly available methylation datasets from other COVID-19 patient groups, including GSE167202 [10] and GSE174818 [7], which had a similar research design and enough clinical information to classify patients as mild or severe using our method.

The differential methylation analysis at the group level took into account cellular heterogeneity or confounders, highlighting 880 differentially methylated CpG sites equally constituted by hyper- and hypo-methylation. Functional annotation of the 880 CpG sites (Additional File 3) pointed out several potentially relevant genes involved in biological processes/pathways related to immune response and already implicated in COVID-19 response.

Among the hypermethylated genes, it is worth mentioning SAMHD1 (SAM And HD Domain Containing Deoxynucleoside Triphosphate Triphosphohydrolase 1), a gene that plays a role in the regulation of the innate immune response. The encoded protein is upregulated in asymptomatic subjects compared to severe COVID-19 cases in response to SARS-CoV-2 infection [19]. In addition, this molecule is reported to be involved in the molecular mechanisms associated with neurological complications related to COVID-19 [20]. Another interesting gene is SETD2 (SET domain containing 2, histone lysine methyltransferase), which enhances the expression of some Interferon-Stimulated Genes (ISGs) by depositing H3K36me3 on their promoters [21, 22].

IRF2 (interferon regulatory factor 2) codes for a member of the interferon regulatory transcription factor (IRF) family, which has been identified as a potential candidate gene for SARS-CoV-2 gender susceptibility [23]. IL12B (interleukin 12B) encodes a subunit of interleukin 12, a cytokine that acts on T and natural killer cells by enhancing their lytic activity and stimulating the production of IFN-gamma. It has been hypothesized that death from COVID-19 may be associated with immunogenetic markers including IL12B, along with HLA-B, IL6, and IL10 [24]. TRIM8 (tripartite motif-containing 8) is suspected to be an E3 ubiquitin-protein ligase involved in multiple biological processes, including the innate immune (IFN-mediated) response [25–27]. Other genes that have been annotated in public database KEGG for their role in the COVID-19 pathway include NLRP3 (NLR family pyrin domain containing 3), MAPK10 (mitogen-activated protein kinase 10), and PIK3CD (phosphatidylinositol-4,5-bisphosphate 3-kinase catalytic subunit delta). Among the hypomethylated genes, several gene loci are worth mentioning: ARID5A (AT-rich interaction domain 5A), which codes for a nucleic acid binding protein involved in immune regulation and cellular homeostasis [28]; CD226 (CD226 molecule), a glycoprotein expressed on the surface of immune cells that has been linked to tissue infiltration and organ dysfunction in severe COVID-19 cases [29]; CD244, a transmembrane protein that acts as a cell surface receptor on immune cells and has been linked to decreased serum cytotoxic effector molecules in severe COVID-19 cases [30]; IL1R1 (interleukin 1 receptor type 1), a cytokine receptor involved in cytokine-induced immune and inflammatory responses that has been linked to cytokine storm and the risk of venous thrombosis events among COVID-19 complications [31, 32]; STAT6 (signal transducer and activator of transcription 6), a nuclear transcription factor that plays a role in IL4-mediated biological responses and has been found to be increased in the lungs of COVID-19 patients [33].

Furthermore, the gene over representation analysis and the gene set enrichment analysis, conducted on the 100 best ranked differentially methylated genes also supported the functional annotation results uncovering an epigenetic impairment of biological processes related to the immune system regulation and interferon-response pathways (Additional File 4). The published genome-wide epigenetic studies on COVID disease [7, 9, 10] showed similar and consistent results. The 880 CpG signature was unable to distinguish all severe COVID-19 patients from mild subjects after unsupervised clustering (Fig. 3) but a subgroup (G4), strongly enriched in severe patients, clearly emerged. Focusing on clinical data of severe patients enriching group 4 we did not observe any significant association with clinical variables able to explain such a clustering. A further analysis of this cluster identified 21 specific CpG sites that were able to distinguish between clinical outcomes, along with the first principal component (PC1) (Fig. 4). The scores of PC1 were then used to estimate the risk of developing a severe outcome, and the logistic regression analysis showed a significant association between PC1 scores and severe outcomes [OR = 2.55 (95% CI 1.9–3.5)]. The regression model took into account important covariates such as clinical factors and pharmacological treatments. Clinical factors included host characteristics (such as gender, age, and smoker status), comorbidities (such as obesity, hypertension, diabetes, cardiovascular disease, and pre-existing cancer), and biochemical parameters (such as elevated creatinine, reduced albumin, elevated aspartate aminotransferase, elevated LDH, elevated C-reactive protein, elevated d-dimer, elevated leukocyte count and, elevated LDL levels). Pharmacological treatments were divided into chronic treatments related to comorbidities and therapeutic treatments administered in the hospital. The regression model showed that neither pharmacological treatments nor clinical variables affected the association between the epi-signature and disease outcome. We further compared the performance of three models for predicting severe outcomes in patients considering: host factors, comorbidities, and biochemical parameters with the aim of verifying whether the epi-signature was able to improve the model based only on clinical information. In each case, we evaluated the models with and without the epi-signature by means of likelihood ratio testing and McFadden's pseudo-R squared. The results showed that the models that also included the epi-signature were always significantly more predictive, explaining a higher proportion of the variance in severe outcomes.

One explanation for this result may lie in the type of patients enrolled in the study. It is known that the presence of comorbidities plays a significant role in determining the risk of developing a severe form of the disease. However, not all subjects with comorbidities or risk factors experience adverse events. The cohort selected in this study mainly consists of fragile subjects with comorbidities, considered at high risk. In fact, the presence of risk factors was so widespread that the analysis of clinical data did not reveal any differences between the two groups examined, with the exception of diabetes. The results seem to indicate that epigenetics could be an additional tool able to improve the ability to discriminate and predict the severe outcome of the disease among fragile subjects. Further strengthening these findings is the observation of a higher grimage clock in patients with more severe prognosis: it has been demonstrated that the grimage clock, the epigenetic signature associated with important mortality risk factors, was better at predicting survival than the risk factors themselves.

Moreover, to evaluate the effectiveness of the 21 CpG epi-signature, we compared patients with severe outcomes and those with mild outcomes to a cohort of individuals, COVID-19 negative. The results confirmed that the epi-signature was able to distinguish between the group with severe COVID-19 outcomes and the COVID-negative group [OR = 2.3 (95% CI 1.78–3.17)]. However, it was not able to differentiate between the group with mild COVID-19 outcomes and the COVID-negative group. This result suggests that the epi-signature may be more specifically related to severe COVID-19 outcomes and not influenced by the presence of COVID-19 infection. Finally, the predictive capacity was confirmed by the efficiency in discriminating cases from controls in two external validation datasets generated from raw data downloaded from public repositories (GEO) which gave consistent and similar odds ratio values GSE167202: OR = 1.4 (95% CI 1.19–1.68) and GSE174818: OR = 1.69 (95% CI 1.34–2.23). Therefore we can hypothesize that the signature of 21 CpG sites may be a valid measure in predicting outcome.

The evidence of an epigenetic perturbation following COVID-19 infection also emerges from subsequent investigations conducted after the classical differential methylation analysis.

When evaluating epigenetic markers used to estimate the biological age [12] we observed a significantly increased epigenetic age acceleration (DNAm GrimAge) in COVID-19 severe cases compared to mild cases. A significant DNAm GrimAge increase is unequivocally present even after evaluating this variable in the two independent GEO validation datasets (GSE167202 and GSE174818) confirming the robustness of the results. This increase occurs even if no appreciable differences in patients' chronological age between the two groups were observed. The hypothesis of a strong relationship between accelerated aging and COVID-19 also emerges from literature data: for example, in Corley et al. [34], the authors correlated the severity of COVID-19 phenotype (with a higher risk of mortality) to a significant increase in DNAm age, proposing the epigenetic clock estimates (both Steve Horvath’s DNAmAge and Grimage) as the main predictor of the disease evolution. In Ying et al. [35], the authors investigated the causal relationship between aging and COVID-19 by analyzing biological age-correlated measurements, suggesting accelerated aging as the cause of enhanced susceptibility to COVID-19 infection and to severe forms of the disease. These results support the hypothesis that epigenetic impairment plays a role in the evolution of the COVID-19 disease.

Another piece of evidence proving epigenetic involvement after COVID-19 infection emerges from the analysis of stochastic epigenetic mutations (SEMs) which represent a robust biomarker of a possible epigenetic drift and an effective indicator of the accumulation of DNA damage related to environmental exposure [15]. In our study, severe COVID-19 patients show a higher burden of SEMs than their mild counterparts, especially hypo-methylations.

This analysis highlights another important aspect related to epigenetics in COVID-19 patients, and the result can be considered robust since we validated it in other cohorts.

We are aware of the limitations and strengths of our EWAS approach: concerning limitations, we limited the analysis to whole blood as representative of the methylation status of the disease but additional studies in alternative tissues may be necessary to confirm results. In addition, the presence of missing data amongst clinical information may have reduced statistical power in some analyses. To mitigate the effect of missing data and optimize statistical power, we adopted the pairwise deletion approach. Another limitation concerns the information on medications and treatments. This information was considered a potential confounder in the regression models and is reported in the Additional materials, however, no conclusions could be drawn about the effect of medications on survival because the study was not designed for this purpose. The reason is that we know that treatments were decided based on clinical conditions and therefore some associations may be biased.

Concerning strengths, the study focused on patients with high-risk clinical factors, enabling the evaluation of an additional layer of epigenetic involvement and improving the ability to explain the disease outcome. The study also added further information by investigating some innovative aspects such as the assessment of epigenetic drift. Finally, the results were successfully replicated in validation cohorts.

## Conclusions

In conclusion, the present study confirms that DNA methylation is involved in the progression of COVID-19 infection. The study extends the current knowledge about the role of epigenetics in the evolution of COVID-19 infection: we focused on Italian patients with comorbidities who are more frequently hospitalized but, despite the frailty burden at admission, may differ in terms of outcomes (good vs poor). Results show that the epigenetic signature already present at the time of admission can predict the risk of severe outcomes in a significant manner. Furthermore, our results were confirmed in a cohort of Covid negative individuals as well as in already published datasets. Finally, the study confirmed that epigenetic drift and age acceleration are associated with severe outcomes both in our as well as in other cohorts. These results indicate an association between host epigenetics and the progression of the disease, which appears to reflect the subject's state of frailty and the resulting response to the infection itself. This information may be useful for personalized, timely, and targeted treatment of COVID-19 patients during the early stages of hospitalization.

## Methods

### Study design/population

This is an observational study evaluating patients with COVID-19 who were admitted to the COVID wards after arriving at the emergency room from February to December 2020. Blood samples and clinical data were collected in the first stages after the COVID-19 attestation. Patients were clinically evaluated from admission to the emergency room to hospitalization in the covid ward. The severity of the disease outcome was determined by evaluating the clinical evolution of COVID-19 patients: 66 subjects who developed a negative evolution (admitted to intensive care unit or dead) were classified as “severe” cases while 124 patients with a less inauspicious clinical course (discharge at home or in other facilities with lower intensity) were assigned to the “mild” group. Patients hospitalized with an already compromised clinical condition (e.g., too low oxygen saturation levels) were filtered out, while, to ensure the representation of the whole spectrum of COVID-19 clinical presentation/evolution, individuals presenting some risk factors associated with comorbidities were accepted. Written informed consent was obtained from all patients. After quality control procedures, performed both at probe and sample level, 187 samples (123 classified as “mild” and 64 classified as “severe”) resulted suitable for analysis. Clinical variables known to be important risk factors associated with the severe outcome were collected. Furthermore, DNA collected before the pandemia obtained from a cohort of 75 gender-and age-matched subjects were analyzed and considered as COVID-19 negative group.

### Predictive model comparison (Clinical vs Epigenetic)

To address whether our epigenetic signature was able to improve a clinical predictive model, we used all available clinical risk factors such as host information, comorbidities and biochemical parameters to build a predictive model, which was then compared to a new one obtained by including the epigenetic signature of 21 CpGs (PC1 score). Clinical data are available in Additional File 1. Specifically, we considered (i) the host factors (Gender [36, 37], Age [6, 37–40], Smoking status [37, 39], Absence of fever [37], (ii) comorbidities (Obesity [41, 42], Hypertension [37, 43], Diabetes [37, 44], Cardiovascular Heart Disease [37, 38], and Pre-existing cancer [45, 46], and (iii) biochemical parameters (Elevated Creatinine (> 1.33 mg/dL) [37], Reduced Albumine(< 4 g/dL) [47], Elevated AsT(> 40 U/L) [37, 38], Elevated LDH(> 245 mU/ml) [37], Elevated C reactive protein(> 8.2 mg/L) [48], Elevated D-Dimer(> 1000 ng/mL) [37, 38], Elevated White Cell Count (> 4 × 109/L) [37], High Levels LDL [49]. Moreover, pharmacological treatments administered during hospitalization and chronicle pharmacological treatments for comorbidities were also considered. The complete list of clinical and pharmacological variables is reported in Additional File 1.

### DNA extraction and quality control

Genomic DNA was extracted from whole blood samples using automatic equipment and a commercial kit based on magnetic beads separation. First, 1.5 μl of total genomic DNA was quantified using an N60 Implen Nanophotometer. Next, DNAs were diluted to a theoretical concentration of 20 ng/μl using a Tris-EDTA buffer (pH 8.0) and re-quantified to ensure the correct sample concentration. Finally, samples showing aberrant protein (260/280) as well as organic compounds (230/260) ratios were discarded or purified.

### Bisulfite conversion and DNA methylation assay

Since methylated cytosine is genetically indistinguishable from the unmethylated, genomic DNA was chemically treated with bisulfite to convert cytosine (C) residues to uracil (U) (thymine (T) after amplification), but leaving 5-methylcytosine residues unaffected. Only good-quality genomic DNA was used as input for bisulfite conversion. 900 ng of good quality genomic DNA were bisulfite converted using the EZ DNA Methylation Kit (Ref: D5001, Zymo Research Corporation) according to the manufacturer’s protocol. Specific incubation conditions (Illumina Protocol) were applied to improve conversion efficiency. To evaluate conversion yield, a single-strand quantification of bisulfite converted DNA (bsDNA) was performed using an N60 Implen Nanophotometer. Fragmented or too diluted DNA samples were discarded or reprocessed. 200 ng/ul of bisulfite-converted DNA were used for hybridization on Illumina Infinium Methylation EPIC BeadChip: these Chips are designed to quantitatively profile the methylation status of 850,000 sites across the genome at single-nucleotide resolution. The samples were processed according to the manufacturer’s protocols in a semi-automated procedure. BeadChips were scanned using the Illumina iScan scanner, a two-color laser (532 nm/660 nm) fluorescent scanner with a 0.375 μm spatial resolution. The fluorescence intensities were stored as intensity data files (*.idat) which can be used as input for most of the available analysis software/packages. The methylation score for each CpG site is represented as β values according to the fluorescent intensity ratio between methylated and unmethylated probes. β values may range between 0 (non methylated) and 1 (completely methylated).

### Average DNA methylation

Differences in the average DNA methylation level between groups were evaluated by comparing the relative distribution of beta values of a subset of 3773 loci classified as “Random Loci” in the Illumina manifest. The results are discussed in the Additional File 4.

### Blood cell type counts and biological age estimation

An estimate of the proportions of blood cells including CD8T cells, CD4T cells, natural killer (NK) cells, B cells, monocytes, granulocytes, and others, was assessed using the DNA Methylation Age Calculator analysis software (https://dnamage.genetics.ucla.edu/) [12]. Estimations were incorporated as covariates in the differential methylation analysis to adjust for confounding effects. DNA methylation measures were also used to predict biological age using different approaches including the pan tissue epigenetic clock by Horvath or DNAm-based biomarker of mortality “DNAm GrimAge” [50]. Details are provided as Additional File 4.

### Differential methylation analyses

We adopted a complementary strategy to explore genome-wide methylation data by combining both EWAS and Stochastic Epigenetic Mutations (SEMs) analyses.

Concerning group-level comparison we took advantage of the RnBeads package in the R environment [51]. Differential methylation analysis was computed both at the site- and region- [Genes, Promoters, CpG island, and Tiling (fixed 5 kb windows)] level, providing a comprehensive evaluation of potential methylation differences.

Stochastic Epigenetic Mutation (SEM) analysis was used as a complementary approach to estimate epigenetic drift at a single individual level. This analysis method, developed by Gentilini et al. [15–18] is widely used in several studies that provided evidence that the number of SEMs correlates with several events such as aging, X chromosome inactivation skewing in women, hepatocellular carcinoma tumor staging, unhealthy exposure such as cigarette smoking, alcohol intake, exposure to toxicants, and finally with socioeconomic position, lifestyle and habits. SEMs have been recognized as possible biomarkers of exposure-related accumulation of DNA damage during lifespan [52].

- a) Sample group analysis and quality control
  Using its integrated modules, RnBeads [51] represents a powerful tool to perform quality control, normalization, and exploratory (e.g., Principal Component Analysis) steps on raw data as well as differential methylation analysis on different regions (genes, promoters, CpG islands, and tiling regions), or ontology enrichment of differentially methylated genes. SNP-enriched probes, unreliable measurements, context-specific, and sex chromosome probes were filtered out in the quality control step using the Greedycut algorithm provided within the RnBeads package. Signal intensities were normalized using the BMIQ (Beta MIxture Quantile) normalization method [53]. Differential methylation analyses were conducted according to the paired sample groups by computing p-values using the limma method for the site level analysis. For the analysis of predefined (Genes, promoters, CpG island, tiling) regions, a combined *p*-value was calculated from the *p*-values of single sites. To avoid potential confounding factors (e.g., age, sex, cell composition, and batch), principal component (PC) and correlation analyses were performed to evaluate the association with both dependent (degree of severity of the disease) and independent (methylation values) variables: according to these analyses, associations were used as covariates in the differential methylation module. Prioritization of differentially methylated genes was conducted by GO Enrichment Analysis via RnBeads using an algorithm (GOstats) based on a hypergeometric test and the hierarchical structure of the gene ontology database [54]. Functional annotation was carried out using the Database for Annotation, Visualization and Integrated Discovery (DAVID) service [55, 56]. Prioritization of top ranked differentially methylated genes was carried out using Gene Set Enrichment Analysis (GSEA) [13, 14] through the WEB-based GEne SeT AnaLysis Toolkit (WebGestalt - http://www.webgestalt.org/).
- b) Individual sample analysis [Stochastic epigenetic mutations (SEMs)]
  This method was applied to the single (individual) methylation profiles to identify, through a non-parametric statistical approach, single aberrant methylation values (extreme outliers—SEMs) according to a reference methylation range (obtained from the same reference subjects). Stochastic Epigenetic Mutations (SEMs) were identified as aberrant beta-values (defined as extreme outliers) falling outside a reference methylation range obtained by the methylation profiles of a reference population and calculated as follows: upper value = Q3+(k*IQR); lower value=*Q*1-(k*IQR); where Q1 is the first quartile, Q3 the third quartile, IQR (Interquartile range)=Q3-Q1 and *k*=3. For each case, extreme outlier values of single methylation profiles were annotated and classified as hyper-methylated or hypo-methylated to controls' relative probe median values. The number of SEMs was compared between the two groups to identify potential differences in epigenetic drift. Moreover, other potential relations between SEMs and other clinical outcomes were also investigated. Quality control, pre-processing, and generation of the *β*-values dataset was performed using the ChAMP R package (Chip Analysis Methylation Pipeline) [57, 58] using a BMIQ normalization method. Sites with a detection *p*-value above 0.01 and a bead count <3 in at least 5% of samples, non-GpG probes, potentially SNP affected probes, probes aligning to multiple locations, and related to X/Y chromosomes were filtered out. Samples with an aberrant number of SEMs were also discarded.

### Statistics

Pairwise deletion approach (available-case analysis) has been adopted to manage missing data to minimize the loss of information and optimize statistical power. The Generalized Linear Regression (glm) model was adopted to evaluate the differences of variables between severe and mild groups.

The likelihood ratio test has been used to compare regression models while McFadden’s pseudo-R squared has been used to test model fits and predictive power. Values were log10 transformed to address skewed/non-normal data. Alternatively, the “Wilcox.test” function provided in the R package “class” was also used. Kolmogorov–Smirnov test was adopted to compare the average DNA methylation levels in the two groups. Gene ontology enrichment analysis was carried out using the R package GOstats, implemented in RnBeads modules [54]. Unless otherwise stated, the statistical significance threshold was set to 0.05.

### Data visualization

Data/results were visualized using specific packages in the R environment. The “ggplot2” package produced PCA charts and boxplots. Heatmaps and Manhattan plots using “pheatmap” and “gap” packages, respectively.

## Supplementary Information

**Additional file 1: **Detailed clinical information of COVID-19 patients.

**Additional file 2: **List of the 880 significant differentially methylated CpG sites obtained by the RnBeads Differential Methylation analysis. Annotation of Illumina positions was obtained using wANNOVAR. The different columns are explained as: Illumina_TargetID: site id; Chr: chromosome of the site; Start: start coordinate of the site; End: end coordinate of the site; mean.diff: difference in methylation means between the two groups: mean.g1(mild)-mean.g2(severe); diffmeth.p.val: p-value obtained from linear models employed in the limma package; diffmeth.p.adj.fdr: FDR adjusted *p*-value of all sites, Epimutation_Type: classification of the differentially methylated site; Func.refGene: CpG site classification according to the position of the annotated gene; Gene.refGene: Gene name associated with the differentially methylated site; GeneDetail.refGene: relative distance(s) from nearest gene(s); 21_CpG_SIGNATURE: CpG univocally associated to Group 4.

**Additional file 3: **Functional annotations of intragenic differentially methylated sites (splitted as hyper- and hypo-methylated lists) using David tool.

**Additional file 4: **Extended results.

## Data Availability

Complementary results are provided in a separate file as Additional File 4. Raw data related to our study are available on the GEO repository under accession number GSE199591 (https://www.ncbi.nlm.nih.gov/geo/query/acc.cgi?&acc=GSE199591). Public access to the GEO repository is open, and thus administrative permission to access and use the data is not needed.

## Abbreviations

COVID-19: Coronavirus disease 2019
EWAS: Epigenome-wide association studies
PCA: Principal component analysis
IQR: InterQuartile range
SEM: Stochastic epigenetic mutation
OR: Odds ratio
ORA: Over representation analysis
GSEA: Gene set enrichment analysis
