# Supplementary material for: Role of epigenetics in the clinical evolution of COVID-19 disease. Epigenome-wide association study identifies markers of severe outcome
Source: Eur J Med Res. 2023 Feb 17;28:81. doi: 10.1186/s40001-023-01032-7 (PMC9936487; doi:10.1186/s40001-023-01032-7)
Supplement: Supplementary file 3 — Additional file 3: Functional annotations of intragenic differentially methylated sites (splitted as hyper- and hypo-methylated lists) using David tool. [file 40001_2023_1032_MOESM3_ESM.docx]

**Additional File 3**

**Functional Annotation of all intragenic hyper-methylated CpG sites**

| **ABR** | [**ABR activator of RhoGEF and GTPase(ABR)**](https://david.ncifcrf.gov/geneReportFull.jsp?rowids=29) | [**Related Genes**](https://david.ncifcrf.gov/relatedGenes.jsp?id=29) | [**Homo sapiens**](http://www.ncbi.nlm.nih.gov/Taxonomy/Browser/wwwtax.cgi?name=Homo%20sapiens) |
| --- | --- | --- | --- |
| **GOTERM_BP_DIRECT** | [signal transduction](http://www.ebi.ac.uk/QuickGO/GTerm?id=GO:0007165), [G-protein coupled receptor signaling pathway](http://www.ebi.ac.uk/QuickGO/GTerm?id=GO:0007186), [small GTPase mediated signal transduction](http://www.ebi.ac.uk/QuickGO/GTerm?id=GO:0007264), [intracellular signal transduction](http://www.ebi.ac.uk/QuickGO/GTerm?id=GO:0035556), [positive regulation of apoptotic process](http://www.ebi.ac.uk/QuickGO/GTerm?id=GO:0043065), [positive regulation of GTPase activity](http://www.ebi.ac.uk/QuickGO/GTerm?id=GO:0043547), [modulation of synaptic transmission](http://www.ebi.ac.uk/QuickGO/GTerm?id=GO:0050804), [regulation of small GTPase mediated signal transduction](http://www.ebi.ac.uk/QuickGO/GTerm?id=GO:0051056), [activation of GTPase activity](http://www.ebi.ac.uk/QuickGO/GTerm?id=GO:0090630), | | |
| **GOTERM_CC_DIRECT** | [cytosol](http://www.ebi.ac.uk/QuickGO/GTerm?id=GO:0005829), [membrane](http://www.ebi.ac.uk/QuickGO/GTerm?id=GO:0016020), [axon](http://www.ebi.ac.uk/QuickGO/GTerm?id=GO:0030424), [dendritic spine](http://www.ebi.ac.uk/QuickGO/GTerm?id=GO:0043197), [Schaffer collateral - CA1 synapse](http://www.ebi.ac.uk/QuickGO/GTerm?id=GO:0098685), [glutamatergic synapse](http://www.ebi.ac.uk/QuickGO/GTerm?id=GO:0098978), | | |
| **GOTERM_MF_DIRECT** | [protein serine/threonine kinase activity](http://www.ebi.ac.uk/QuickGO/GTerm?id=GO:0004674), [guanyl-nucleotide exchange factor activity](http://www.ebi.ac.uk/QuickGO/GTerm?id=GO:0005085), [GTPase activator activity](http://www.ebi.ac.uk/QuickGO/GTerm?id=GO:0005096), [protein binding](http://www.ebi.ac.uk/QuickGO/GTerm?id=GO:0005515), | | |
| **INTERPRO** | [C2 calcium-dependent membrane targeting](https://www.ebi.ac.uk/interpro/entry/InterPro/IPR000008), [Rho GTPase-activating protein domain](https://www.ebi.ac.uk/interpro/entry/InterPro/IPR000198), [Dbl homology (DH) domain](https://www.ebi.ac.uk/interpro/entry/InterPro/IPR000219), [Guanine-nucleotide dissociation stimulator, CDC24, conserved site](https://www.ebi.ac.uk/interpro/entry/InterPro/IPR001331), [Pleckstrin homology domain](https://www.ebi.ac.uk/interpro/entry/InterPro/IPR001849), [Rho GTPase activation protein](https://www.ebi.ac.uk/interpro/entry/InterPro/IPR008936), [Pleckstrin homology-like domain](https://www.ebi.ac.uk/interpro/entry/InterPro/IPR011993), [Bcr-Abl oncoprotein oligomerisation](https://www.ebi.ac.uk/interpro/entry/InterPro/IPR015123), | | |
| **SMART** | [PH](http://smart.embl.de/smart/do_annotation.pl?DOMAIN=SM00233), [C2](http://smart.embl.de/smart/do_annotation.pl?DOMAIN=SM00239), [RhoGAP](http://smart.embl.de/smart/do_annotation.pl?DOMAIN=SM00324), [RhoGEF](http://smart.embl.de/smart/do_annotation.pl?DOMAIN=SM00325), | | |
| **UP_KW_CELLULAR_COMPONENT** | [Synapse](http://www.uniprot.org/keywords/?query=KW-0770), [Cell junction](http://www.uniprot.org/keywords/?query=KW-0965), [Cell projection](http://www.uniprot.org/keywords/?query=KW-0966), | | |
| **UP_KW_DOMAIN** | [Coiled coil](http://www.uniprot.org/keywords/?query=KW-0175), | | |
| **UP_KW_MOLECULAR_FUNCTION** | [GTPase activation](http://www.uniprot.org/keywords/?query=KW-0343), [Guanine-nucleotide releasing factor](http://www.uniprot.org/keywords/?query=KW-0344), | | |
| **UP_KW_PTM** | [Phosphoprotein](http://www.uniprot.org/keywords/?query=KW-0597), | | |
| **UP_SEQ_FEATURE** | COMPBIAS:Polar residues, DOMAIN:C2, DOMAIN:DH, DOMAIN:PH, DOMAIN:Rho-GAP, MUTAGEN:N->A: Loss of GAP activity; when associated with A-683., MUTAGEN:R->A: Reduces GAP activity. Loss of GAP activity; when associated with A-795., MUTAGEN:V->A: Abolishes interaction with DLG4. No effect on synaptic localization., REGION:Disordered, | | |
| **ART4** | [**ADP-ribosyltransferase 4 (inactive) (Dombrock blood group)(ART4)**](https://david.ncifcrf.gov/geneReportFull.jsp?rowids=420) | [**Related Genes**](https://david.ncifcrf.gov/relatedGenes.jsp?id=420) | [**Homo sapiens**](http://www.ncbi.nlm.nih.gov/Taxonomy/Browser/wwwtax.cgi?name=Homo%20sapiens) |
| **GOTERM_BP_DIRECT** | [protein ADP-ribosylation](http://www.ebi.ac.uk/QuickGO/GTerm?id=GO:0006471), [arginine metabolic process](http://www.ebi.ac.uk/QuickGO/GTerm?id=GO:0006525), [peptidyl-arginine ADP-ribosylation](http://www.ebi.ac.uk/QuickGO/GTerm?id=GO:0018120), | | |
| **GOTERM_CC_DIRECT** | [extracellular region](http://www.ebi.ac.uk/QuickGO/GTerm?id=GO:0005576), [plasma membrane](http://www.ebi.ac.uk/QuickGO/GTerm?id=GO:0005886), [membrane](http://www.ebi.ac.uk/QuickGO/GTerm?id=GO:0016020), [integral component of membrane](http://www.ebi.ac.uk/QuickGO/GTerm?id=GO:0016021), [anchored component of membrane](http://www.ebi.ac.uk/QuickGO/GTerm?id=GO:0031225), | | |
| **GOTERM_MF_DIRECT** | [NAD+ ADP-ribosyltransferase activity](http://www.ebi.ac.uk/QuickGO/GTerm?id=GO:0003950), [NAD(P)+-protein-arginine ADP-ribosyltransferase activity](http://www.ebi.ac.uk/QuickGO/GTerm?id=GO:0003956), [transferase activity](http://www.ebi.ac.uk/QuickGO/GTerm?id=GO:0016740), | | |
| **INTERPRO** | [NAD](https://www.ebi.ac.uk/interpro/entry/InterPro/IPR000768), | | |
| **OMIM_DISEASE** | [Blood group, Dombrock](http://omim.org/entry/616060), | | |
| **UP_KW_CELLULAR_COMPONENT** | [Membrane](http://www.uniprot.org/keywords/?query=KW-0472), [Cell membrane](http://www.uniprot.org/keywords/?query=KW-1003), | | |
| **UP_KW_DOMAIN** | [Signal](http://www.uniprot.org/keywords/?query=KW-0732), [Transmembrane](http://www.uniprot.org/keywords/?query=KW-0812), [Transmembrane helix](http://www.uniprot.org/keywords/?query=KW-1133), | | |
| **UP_KW_LIGAND** | [NAD](http://www.uniprot.org/keywords/?query=KW-0520), [NADP](http://www.uniprot.org/keywords/?query=KW-0521), | | |
| **UP_KW_MOLECULAR_FUNCTION** | [Blood group antigen](http://www.uniprot.org/keywords/?query=KW-0095), [Glycosyltransferase](http://www.uniprot.org/keywords/?query=KW-0328), [Transferase](http://www.uniprot.org/keywords/?query=KW-0808), | | |
| **UP_KW_PTM** | [Glycoprotein](http://www.uniprot.org/keywords/?query=KW-0325), [GPI-anchor](http://www.uniprot.org/keywords/?query=KW-0336), [Lipoprotein](http://www.uniprot.org/keywords/?query=KW-0449), [Disulfide bond](http://www.uniprot.org/keywords/?query=KW-1015), | | |
| **UP_SEQ_FEATURE** | BINDING:NAD, CARBOHYD:N-linked (GlcNAc...) asparagine, LIPID:GPI-anchor amidated alanine, PROPEP:Removed in mature form, TRANSMEM:Helical, | | |
| **AGBL4** | [**AGBL carboxypeptidase 4(AGBL4)**](https://david.ncifcrf.gov/geneReportFull.jsp?rowids=84871) | [**Related Genes**](https://david.ncifcrf.gov/relatedGenes.jsp?id=84871) | [**Homo sapiens**](http://www.ncbi.nlm.nih.gov/Taxonomy/Browser/wwwtax.cgi?name=Homo%20sapiens) |
| **COG_ONTOLOGY** | [Amino acid transport and metabolism](http://www.ncbi.nlm.nih.gov/COG/new/), | | |
| **GOTERM_BP_DIRECT** | [proteolysis](http://www.ebi.ac.uk/QuickGO/GTerm?id=GO:0006508), [negative regulation of cell proliferation](http://www.ebi.ac.uk/QuickGO/GTerm?id=GO:0008285), [central nervous system neuron development](http://www.ebi.ac.uk/QuickGO/GTerm?id=GO:0021954), [protein deglutamylation](http://www.ebi.ac.uk/QuickGO/GTerm?id=GO:0035608), [C-terminal protein deglutamylation](http://www.ebi.ac.uk/QuickGO/GTerm?id=GO:0035609), [protein side chain deglutamylation](http://www.ebi.ac.uk/QuickGO/GTerm?id=GO:0035610), [defense response to virus](http://www.ebi.ac.uk/QuickGO/GTerm?id=GO:0051607), [anterograde axonal transport of mitochondrion](http://www.ebi.ac.uk/QuickGO/GTerm?id=GO:0098957), [retrograde axonal transport of mitochondrion](http://www.ebi.ac.uk/QuickGO/GTerm?id=GO:0098958), [positive regulation of protein ubiquitination involved in ubiquitin-dependent protein catabolic process](http://www.ebi.ac.uk/QuickGO/GTerm?id=GO:2000060), | | |
| **GOTERM_CC_DIRECT** | [Golgi apparatus](http://www.ebi.ac.uk/QuickGO/GTerm?id=GO:0005794), [centriole](http://www.ebi.ac.uk/QuickGO/GTerm?id=GO:0005814), [cytosol](http://www.ebi.ac.uk/QuickGO/GTerm?id=GO:0005829), [ciliary basal body](http://www.ebi.ac.uk/QuickGO/GTerm?id=GO:0036064), [axon cytoplasm](http://www.ebi.ac.uk/QuickGO/GTerm?id=GO:1904115), | | |
| **GOTERM_MF_DIRECT** | [metallocarboxypeptidase activity](http://www.ebi.ac.uk/QuickGO/GTerm?id=GO:0004181), [zinc ion binding](http://www.ebi.ac.uk/QuickGO/GTerm?id=GO:0008270), [tubulin binding](http://www.ebi.ac.uk/QuickGO/GTerm?id=GO:0015631), | | |
| **INTERPRO** | [Peptidase M14, carboxypeptidase A](https://www.ebi.ac.uk/interpro/entry/InterPro/IPR000834), | | |
| **SMART** | [Zn_pept](http://smart.embl.de/smart/do_annotation.pl?DOMAIN=SM00631), | | |
| **UP_KW_CELLULAR_COMPONENT** | [Cytoskeleton](http://www.uniprot.org/keywords/?query=KW-0206), [Golgi apparatus](http://www.uniprot.org/keywords/?query=KW-0333), [Cytoplasm](http://www.uniprot.org/keywords/?query=KW-0963), [Cell projection](http://www.uniprot.org/keywords/?query=KW-0966), | | |
| **UP_KW_LIGAND** | [Metal-binding](http://www.uniprot.org/keywords/?query=KW-0479), [Zinc](http://www.uniprot.org/keywords/?query=KW-0862), | | |
| **UP_KW_MOLECULAR_FUNCTION** | [Carboxypeptidase](http://www.uniprot.org/keywords/?query=KW-0121), [Hydrolase](http://www.uniprot.org/keywords/?query=KW-0378), [Metalloprotease](http://www.uniprot.org/keywords/?query=KW-0482), [Protease](http://www.uniprot.org/keywords/?query=KW-0645), | | |
| **UP_SEQ_FEATURE** | ACT_SITE:Nucleophile, COMPBIAS:Polar residues, DOMAIN:Pepdidase_M14_N, DOMAIN:Peptidase_M14, METAL:Zinc, REGION:Disordered, | | |
| **ABCA1** | [**ATP binding cassette subfamily A member 1(ABCA1)**](https://david.ncifcrf.gov/geneReportFull.jsp?rowids=19) | [**Related Genes**](https://david.ncifcrf.gov/relatedGenes.jsp?id=19) | [**Homo sapiens**](http://www.ncbi.nlm.nih.gov/Taxonomy/Browser/wwwtax.cgi?name=Homo%20sapiens) |
| **BIOCARTA** | [FXR and LXR Regulation of Cholesterol Metabolism](https://david.ncifcrf.gov/biocarta.jsp?path=h_fxrPathway$FXR%20and%20LXR%20Regulation%20of%20Cholesterol%20Metabolism&termId=30000119&source=biocarta), [Nuclear Receptors in Lipid Metabolism and Toxicity](https://david.ncifcrf.gov/biocarta.jsp?path=h_nuclearRsPathway$Nuclear%20Receptors%20in%20Lipid%20Metabolism%20and%20Toxicity&termId=30000212&source=biocarta), | | |
| **GOTERM_BP_DIRECT** | [peptide secretion](http://www.ebi.ac.uk/QuickGO/GTerm?id=GO:0002790), [protein lipidation](http://www.ebi.ac.uk/QuickGO/GTerm?id=GO:0006497), [lipid transport](http://www.ebi.ac.uk/QuickGO/GTerm?id=GO:0006869), [phagocytosis, engulfment](http://www.ebi.ac.uk/QuickGO/GTerm?id=GO:0006911), [lysosome organization](http://www.ebi.ac.uk/QuickGO/GTerm?id=GO:0007040), [G-protein coupled receptor signaling pathway](http://www.ebi.ac.uk/QuickGO/GTerm?id=GO:0007186), [adenylate cyclase-activating G-protein coupled receptor signaling pathway](http://www.ebi.ac.uk/QuickGO/GTerm?id=GO:0007189), [cholesterol metabolic process](http://www.ebi.ac.uk/QuickGO/GTerm?id=GO:0008203), [protein secretion](http://www.ebi.ac.uk/QuickGO/GTerm?id=GO:0009306), [negative regulation of macrophage derived foam cell differentiation](http://www.ebi.ac.uk/QuickGO/GTerm?id=GO:0010745), [positive regulation of cholesterol efflux](http://www.ebi.ac.uk/QuickGO/GTerm?id=GO:0010875), [negative regulation of cholesterol storage](http://www.ebi.ac.uk/QuickGO/GTerm?id=GO:0010887), [endosomal transport](http://www.ebi.ac.uk/QuickGO/GTerm?id=GO:0016197), [regulation of metabolic process](http://www.ebi.ac.uk/QuickGO/GTerm?id=GO:0019222), [signal release](http://www.ebi.ac.uk/QuickGO/GTerm?id=GO:0023061), [intracellular receptor signaling pathway](http://www.ebi.ac.uk/QuickGO/GTerm?id=GO:0030522), [intracellular cholesterol transport](http://www.ebi.ac.uk/QuickGO/GTerm?id=GO:0032367), [regulation of Cdc42 protein signal transduction](http://www.ebi.ac.uk/QuickGO/GTerm?id=GO:0032489), [cholesterol efflux](http://www.ebi.ac.uk/QuickGO/GTerm?id=GO:0033344), [phospholipid efflux](http://www.ebi.ac.uk/QuickGO/GTerm?id=GO:0033700), [high-density lipoprotein particle assembly](http://www.ebi.ac.uk/QuickGO/GTerm?id=GO:0034380), [response to laminar fluid shear stress](http://www.ebi.ac.uk/QuickGO/GTerm?id=GO:0034616), [apolipoprotein A-I-mediated signaling pathway](http://www.ebi.ac.uk/QuickGO/GTerm?id=GO:0038027), [cholesterol homeostasis](http://www.ebi.ac.uk/QuickGO/GTerm?id=GO:0042632), [reverse cholesterol transport](http://www.ebi.ac.uk/QuickGO/GTerm?id=GO:0043691), [phospholipid translocation](http://www.ebi.ac.uk/QuickGO/GTerm?id=GO:0045332), [phospholipid homeostasis](http://www.ebi.ac.uk/QuickGO/GTerm?id=GO:0055091), [platelet dense granule organization](http://www.ebi.ac.uk/QuickGO/GTerm?id=GO:0060155), [cellular response to lipopolysaccharide](http://www.ebi.ac.uk/QuickGO/GTerm?id=GO:0071222), [cellular response to retinoic acid](http://www.ebi.ac.uk/QuickGO/GTerm?id=GO:0071300), [cellular response to low-density lipoprotein particle stimulus](http://www.ebi.ac.uk/QuickGO/GTerm?id=GO:0071404), [protein transmembrane transport](http://www.ebi.ac.uk/QuickGO/GTerm?id=GO:0071806), [regulation of high-density lipoprotein particle assembly](http://www.ebi.ac.uk/QuickGO/GTerm?id=GO:0090107), [positive regulation of high-density lipoprotein particle assembly](http://www.ebi.ac.uk/QuickGO/GTerm?id=GO:0090108), [sphingolipid translocation](http://www.ebi.ac.uk/QuickGO/GTerm?id=GO:0099039), | | |
| **GOTERM_CC_DIRECT** | [endosome](http://www.ebi.ac.uk/QuickGO/GTerm?id=GO:0005768), [endoplasmic reticulum membrane](http://www.ebi.ac.uk/QuickGO/GTerm?id=GO:0005789), [Golgi apparatus](http://www.ebi.ac.uk/QuickGO/GTerm?id=GO:0005794), [plasma membrane](http://www.ebi.ac.uk/QuickGO/GTerm?id=GO:0005886), [integral component of plasma membrane](http://www.ebi.ac.uk/QuickGO/GTerm?id=GO:0005887), [external side of plasma membrane](http://www.ebi.ac.uk/QuickGO/GTerm?id=GO:0009897), [integral component of membrane](http://www.ebi.ac.uk/QuickGO/GTerm?id=GO:0016021), [endocytic vesicle](http://www.ebi.ac.uk/QuickGO/GTerm?id=GO:0030139), [intracellular membrane-bounded organelle](http://www.ebi.ac.uk/QuickGO/GTerm?id=GO:0043231), [membrane raft](http://www.ebi.ac.uk/QuickGO/GTerm?id=GO:0045121), [phagocytic vesicle](http://www.ebi.ac.uk/QuickGO/GTerm?id=GO:0045335), [perinuclear region of cytoplasm](http://www.ebi.ac.uk/QuickGO/GTerm?id=GO:0048471), [intracellular vesicle](http://www.ebi.ac.uk/QuickGO/GTerm?id=GO:0097708), | | |
| **GOTERM_MF_DIRECT** | [receptor binding](http://www.ebi.ac.uk/QuickGO/GTerm?id=GO:0005102), [lipid transporter activity](http://www.ebi.ac.uk/QuickGO/GTerm?id=GO:0005319), [protein binding](http://www.ebi.ac.uk/QuickGO/GTerm?id=GO:0005515), [ATP binding](http://www.ebi.ac.uk/QuickGO/GTerm?id=GO:0005524), [phospholipid transporter activity](http://www.ebi.ac.uk/QuickGO/GTerm?id=GO:0005548), [protein transmembrane transporter activity](http://www.ebi.ac.uk/QuickGO/GTerm?id=GO:0008320), [cholesterol binding](http://www.ebi.ac.uk/QuickGO/GTerm?id=GO:0015485), [syntaxin binding](http://www.ebi.ac.uk/QuickGO/GTerm?id=GO:0019905), [phosphatidylcholine binding](http://www.ebi.ac.uk/QuickGO/GTerm?id=GO:0031210), [small GTPase binding](http://www.ebi.ac.uk/QuickGO/GTerm?id=GO:0031267), [apolipoprotein binding](http://www.ebi.ac.uk/QuickGO/GTerm?id=GO:0034185), [apolipoprotein A-I binding](http://www.ebi.ac.uk/QuickGO/GTerm?id=GO:0034186), [apolipoprotein A-I receptor activity](http://www.ebi.ac.uk/QuickGO/GTerm?id=GO:0034188), [ATPase activity, coupled to transmembrane movement of substances](http://www.ebi.ac.uk/QuickGO/GTerm?id=GO:0042626), [sphingolipid-translocating ATPase activity](http://www.ebi.ac.uk/QuickGO/GTerm?id=GO:0046623), [ATPase binding](http://www.ebi.ac.uk/QuickGO/GTerm?id=GO:0051117), [phosphatidylcholine-translocating ATPase activity](http://www.ebi.ac.uk/QuickGO/GTerm?id=GO:0090554), [phosphatidylserine-translocating ATPase activity](http://www.ebi.ac.uk/QuickGO/GTerm?id=GO:0090556), | | |
| **INTERPRO** | [ABC transporter-like](https://www.ebi.ac.uk/interpro/entry/InterPro/IPR003439), [AAA+ ATPase domain](https://www.ebi.ac.uk/interpro/entry/InterPro/IPR003593), [ABC transporter, conserved site](https://www.ebi.ac.uk/interpro/entry/InterPro/IPR017871), [ABC transporter A, ABCA](https://www.ebi.ac.uk/interpro/entry/InterPro/IPR026082), [P-loop containing nucleoside triphosphate hydrolase](https://www.ebi.ac.uk/interpro/entry/InterPro/IPR027417), | | |
| **KEGG_PATHWAY** | [ABC transporters](https://david.ncifcrf.gov/kegg.jsp?path=hsa02010$ABC%20transporters&termId=520047922&source=kegg), [Fat digestion and absorption](https://david.ncifcrf.gov/kegg.jsp?path=hsa04975$Fat%20digestion%20and%20absorption&termId=520048082&source=kegg), [Cholesterol metabolism](https://david.ncifcrf.gov/kegg.jsp?path=hsa04979$Cholesterol%20metabolism&termId=520048086&source=kegg), [Lipid and atherosclerosis](https://david.ncifcrf.gov/kegg.jsp?path=hsa05417$Lipid%20and%20atherosclerosis&termId=520048169&source=kegg), | | |
| **OMIM_DISEASE** | [Tangier disease](http://omim.org/entry/205400), [HDL deficiency, familial, 1](http://omim.org/entry/604091), | | |
| **SMART** | [AAA](http://smart.embl.de/smart/do_annotation.pl?DOMAIN=SM00382), | | |
| **UP_KW_BIOLOGICAL_PROCESS** | [Cholesterol metabolism](http://www.uniprot.org/keywords/?query=KW-0153), [Lipid metabolism](http://www.uniprot.org/keywords/?query=KW-0443), [Steroid metabolism](http://www.uniprot.org/keywords/?query=KW-0753), [Transport](http://www.uniprot.org/keywords/?query=KW-0813), [Sterol metabolism](http://www.uniprot.org/keywords/?query=KW-1207), | | |
| **UP_KW_CELLULAR_COMPONENT** | [Membrane](http://www.uniprot.org/keywords/?query=KW-0472), [Endosome](http://www.uniprot.org/keywords/?query=KW-0967), [Cell membrane](http://www.uniprot.org/keywords/?query=KW-1003), | | |
| **UP_KW_DISEASE** | [Atherosclerosis](http://www.uniprot.org/keywords/?query=KW-0065), [Disease variant](http://www.uniprot.org/keywords/?query=KW-0225), | | |
| **UP_KW_DOMAIN** | [Repeat](http://www.uniprot.org/keywords/?query=KW-0677), [Transmembrane](http://www.uniprot.org/keywords/?query=KW-0812), [Transmembrane helix](http://www.uniprot.org/keywords/?query=KW-1133), | | |
| **UP_KW_LIGAND** | [ATP-binding](http://www.uniprot.org/keywords/?query=KW-0067), [Nucleotide-binding](http://www.uniprot.org/keywords/?query=KW-0547), | | |
| **UP_KW_MOLECULAR_FUNCTION** | [Translocase](http://www.uniprot.org/keywords/?query=KW-1278), | | |
| **UP_KW_PTM** | [Glycoprotein](http://www.uniprot.org/keywords/?query=KW-0325), [Lipoprotein](http://www.uniprot.org/keywords/?query=KW-0449), [Palmitate](http://www.uniprot.org/keywords/?query=KW-0564), [Phosphoprotein](http://www.uniprot.org/keywords/?query=KW-0597), [Disulfide bond](http://www.uniprot.org/keywords/?query=KW-1015), | | |
| **UP_SEQ_FEATURE** | CARBOHYD:N-linked (GlcNAc...) asparagine, COMPBIAS:Basic and acidic residues, DOMAIN:ABC transporter, DOMAIN:ABC transporter 1, DOMAIN:ABC transporter 2, LIPID:S-palmitoyl cysteine, MUTAGEN:C->S: Decreased palmitoylation; when associated with S-3, S-23 and S-1110., MUTAGEN:C->S: Decreased palmitoylation; when associated with S-3, S-23 and S-1111., MUTAGEN:C->S: Mild decrease of palmitoylation. Loss of localization to plasma membrane. Decreased cholesterol efflux. Decreased phospholipid efflux. Decreased palmitoylation; when associated with S-23, S-1110 and S-1111., MUTAGEN:C->S: Mild decrease of palmitoylation. Loss of localization to plasma membrane. Decreased palmitoylation; when associated with S-3, S-1110 and S-1111., MUTAGEN:F->L: Moderately decreased protein abundance. Highly decreased ATPase activity. Highly decreased phospholipid translocase activity., MUTAGEN:K->M: Inhibits ATPase activity; when associated with M-1952. Decreases translocase activity; when associated with M-1952. Does not affect protein subcellular localization in plasma membrane and endosome; when associated with M-1952., MUTAGEN:K->M: Inhibits ATPase activity; when associated with M-939. Decreases translocase activity; when associated with M-939. Does not affect protein subcellular localization in plasma membrane and endosome; when associated with M-939., MUTAGEN:S->C: Highly decreased protein abundance. Highly decreased ATPase activity. Highly decreased phospholipid translocase activity., MUTAGEN:T->M: Moderately decreased protein abundance. Does not affect ATPase activity. Moderately decreased phospholipid translocase activity., NP_BIND:ATP, NP_BIND:ATP 1, NP_BIND:ATP 2, REGION:Disordered, TOPO_DOM:Extracellular, TRANSMEM:Helical, | | |
| **ABCA2** | [**ATP binding cassette subfamily A member 2(ABCA2)**](https://david.ncifcrf.gov/geneReportFull.jsp?rowids=20) | [**Related Genes**](https://david.ncifcrf.gov/relatedGenes.jsp?id=20) | [**Homo sapiens**](http://www.ncbi.nlm.nih.gov/Taxonomy/Browser/wwwtax.cgi?name=Homo%20sapiens) |
| **GOTERM_BP_DIRECT** | [ganglioside metabolic process](http://www.ebi.ac.uk/QuickGO/GTerm?id=GO:0001573), [regulation of transcription from RNA polymerase II promoter](http://www.ebi.ac.uk/QuickGO/GTerm?id=GO:0006357), [lipid metabolic process](http://www.ebi.ac.uk/QuickGO/GTerm?id=GO:0006629), [sphingomyelin metabolic process](http://www.ebi.ac.uk/QuickGO/GTerm?id=GO:0006684), [glycosphingolipid metabolic process](http://www.ebi.ac.uk/QuickGO/GTerm?id=GO:0006687), [lipid transport](http://www.ebi.ac.uk/QuickGO/GTerm?id=GO:0006869), [locomotory behavior](http://www.ebi.ac.uk/QuickGO/GTerm?id=GO:0007626), [response to xenobiotic stimulus](http://www.ebi.ac.uk/QuickGO/GTerm?id=GO:0009410), [regulation of cholesterol esterification](http://www.ebi.ac.uk/QuickGO/GTerm?id=GO:0010872), [central nervous system myelin formation](http://www.ebi.ac.uk/QuickGO/GTerm?id=GO:0032289), [regulation of intracellular cholesterol transport](http://www.ebi.ac.uk/QuickGO/GTerm?id=GO:0032383), [negative regulation of intracellular cholesterol transport](http://www.ebi.ac.uk/QuickGO/GTerm?id=GO:0032384), [positive regulation of low-density lipoprotein particle receptor catabolic process](http://www.ebi.ac.uk/QuickGO/GTerm?id=GO:0032805), [response to drug](http://www.ebi.ac.uk/QuickGO/GTerm?id=GO:0042493), [cholesterol homeostasis](http://www.ebi.ac.uk/QuickGO/GTerm?id=GO:0042632), [positive regulation of amyloid precursor protein biosynthetic process](http://www.ebi.ac.uk/QuickGO/GTerm?id=GO:0042986), [sphingosine biosynthetic process](http://www.ebi.ac.uk/QuickGO/GTerm?id=GO:0046512), [response to steroid hormone](http://www.ebi.ac.uk/QuickGO/GTerm?id=GO:0048545), [regulation of endopeptidase activity](http://www.ebi.ac.uk/QuickGO/GTerm?id=GO:0052548), [transmembrane transport](http://www.ebi.ac.uk/QuickGO/GTerm?id=GO:0055085), [regulation of protein glycosylation](http://www.ebi.ac.uk/QuickGO/GTerm?id=GO:0060049), [response to cholesterol](http://www.ebi.ac.uk/QuickGO/GTerm?id=GO:0070723), [negative regulation of phospholipid biosynthetic process](http://www.ebi.ac.uk/QuickGO/GTerm?id=GO:0071072), [negative regulation of sphingolipid biosynthetic process](http://www.ebi.ac.uk/QuickGO/GTerm?id=GO:0090155), [cellular sphingolipid homeostasis](http://www.ebi.ac.uk/QuickGO/GTerm?id=GO:0090156), [negative regulation of cholesterol efflux](http://www.ebi.ac.uk/QuickGO/GTerm?id=GO:0090370), [ceramide translocation](http://www.ebi.ac.uk/QuickGO/GTerm?id=GO:0099040), [regulation of post-translational protein modification](http://www.ebi.ac.uk/QuickGO/GTerm?id=GO:1901873), [positive regulation of beta-amyloid formation](http://www.ebi.ac.uk/QuickGO/GTerm?id=GO:1902004), [positive regulation of amyloid precursor protein catabolic process](http://www.ebi.ac.uk/QuickGO/GTerm?id=GO:1902993), [regulation of protein localization to cell periphery](http://www.ebi.ac.uk/QuickGO/GTerm?id=GO:1904375), [negative regulation of low-density lipoprotein receptor activity](http://www.ebi.ac.uk/QuickGO/GTerm?id=GO:1905598), [negative regulation of receptor-mediated endocytosis involved in cholesterol transport](http://www.ebi.ac.uk/QuickGO/GTerm?id=GO:1905601), [regulation of protein localization to cell surface](http://www.ebi.ac.uk/QuickGO/GTerm?id=GO:2000008), | | |
| **GOTERM_CC_DIRECT** | [lysosome](http://www.ebi.ac.uk/QuickGO/GTerm?id=GO:0005764), [lysosomal membrane](http://www.ebi.ac.uk/QuickGO/GTerm?id=GO:0005765), [endosome](http://www.ebi.ac.uk/QuickGO/GTerm?id=GO:0005768), [microtubule organizing center](http://www.ebi.ac.uk/QuickGO/GTerm?id=GO:0005815), [plasma membrane](http://www.ebi.ac.uk/QuickGO/GTerm?id=GO:0005886), [endosome membrane](http://www.ebi.ac.uk/QuickGO/GTerm?id=GO:0010008), [membrane](http://www.ebi.ac.uk/QuickGO/GTerm?id=GO:0016020), [integral component of membrane](http://www.ebi.ac.uk/QuickGO/GTerm?id=GO:0016021), [cytoplasmic vesicle](http://www.ebi.ac.uk/QuickGO/GTerm?id=GO:0031410), [ATP-binding cassette (ABC) transporter complex](http://www.ebi.ac.uk/QuickGO/GTerm?id=GO:0043190), [intracellular membrane-bounded organelle](http://www.ebi.ac.uk/QuickGO/GTerm?id=GO:0043231), | | |
| **GOTERM_MF_DIRECT** | [nucleotide binding](http://www.ebi.ac.uk/QuickGO/GTerm?id=GO:0000166), [lipid transporter activity](http://www.ebi.ac.uk/QuickGO/GTerm?id=GO:0005319), [ATP binding](http://www.ebi.ac.uk/QuickGO/GTerm?id=GO:0005524), [ATPase activity](http://www.ebi.ac.uk/QuickGO/GTerm?id=GO:0016887), [ATPase activity, coupled to transmembrane movement of substances](http://www.ebi.ac.uk/QuickGO/GTerm?id=GO:0042626), [endopeptidase regulator activity](http://www.ebi.ac.uk/QuickGO/GTerm?id=GO:0061135), [ceramide-translocating ATPase activity](http://www.ebi.ac.uk/QuickGO/GTerm?id=GO:0099038), | | |
| **INTERPRO** | [ABC transporter-like](https://www.ebi.ac.uk/interpro/entry/InterPro/IPR003439), [AAA+ ATPase domain](https://www.ebi.ac.uk/interpro/entry/InterPro/IPR003593), [ATPase, AAA-type, core](https://www.ebi.ac.uk/interpro/entry/InterPro/IPR003959), [ABC transporter, conserved site](https://www.ebi.ac.uk/interpro/entry/InterPro/IPR017871), [ABC transporter A, ABCA](https://www.ebi.ac.uk/interpro/entry/InterPro/IPR026082), [P-loop containing nucleoside triphosphate hydrolase](https://www.ebi.ac.uk/interpro/entry/InterPro/IPR027417), | | |
| **KEGG_PATHWAY** | [ABC transporters](https://david.ncifcrf.gov/kegg.jsp?path=hsa02010$ABC%20transporters&termId=520047922&source=kegg), [Lysosome](https://david.ncifcrf.gov/kegg.jsp?path=hsa04142$Lysosome&termId=520047968&source=kegg), | | |
| **OMIM_DISEASE** | [Intellectual developmental disorder with poor growth and with or without seizures or ataxia](http://omim.org/entry/618808), | | |
| **SMART** | [AAA](http://smart.embl.de/smart/do_annotation.pl?DOMAIN=SM00382), | | |
| **UP_KW_BIOLOGICAL_PROCESS** | [Transport](http://www.uniprot.org/keywords/?query=KW-0813), | | |
| **UP_KW_CELLULAR_COMPONENT** | [Lysosome](http://www.uniprot.org/keywords/?query=KW-0458), [Membrane](http://www.uniprot.org/keywords/?query=KW-0472), [Endosome](http://www.uniprot.org/keywords/?query=KW-0967), | | |
| **UP_KW_DISEASE** | [Disease variant](http://www.uniprot.org/keywords/?query=KW-0225), [Epilepsy](http://www.uniprot.org/keywords/?query=KW-0887), [Mental retardation](http://www.uniprot.org/keywords/?query=KW-0991), | | |
| **UP_KW_DOMAIN** | [Coiled coil](http://www.uniprot.org/keywords/?query=KW-0175), [Repeat](http://www.uniprot.org/keywords/?query=KW-0677), [Transmembrane](http://www.uniprot.org/keywords/?query=KW-0812), [Transmembrane helix](http://www.uniprot.org/keywords/?query=KW-1133), | | |
| **UP_KW_LIGAND** | [ATP-binding](http://www.uniprot.org/keywords/?query=KW-0067), [Nucleotide-binding](http://www.uniprot.org/keywords/?query=KW-0547), | | |
| **UP_KW_MOLECULAR_FUNCTION** | [Translocase](http://www.uniprot.org/keywords/?query=KW-1278), | | |
| **UP_KW_PTM** | [Glycoprotein](http://www.uniprot.org/keywords/?query=KW-0325), [Methylation](http://www.uniprot.org/keywords/?query=KW-0488), [Phosphoprotein](http://www.uniprot.org/keywords/?query=KW-0597), | | |
| **UP_SEQ_FEATURE** | CARBOHYD:N-linked (GlcNAc...) asparagine, COMPBIAS:Basic and acidic residues, COMPBIAS:Pro residues, DOMAIN:ABC transporter, DOMAIN:ABC transporter 1, DOMAIN:ABC transporter 2, DOMAIN:ATPase_AAA_core, MUTAGEN:Q->R: Abolishes methylation by N6AMT1., NP_BIND:ATP, NP_BIND:ATP 1, NP_BIND:ATP 2, REGION:Disordered, TRANSMEM:Helical, | | |
| **ATP2B4** | [**ATPase plasma membrane Ca2+ transporting 4(ATP2B4)**](https://david.ncifcrf.gov/geneReportFull.jsp?rowids=493) | [**Related Genes**](https://david.ncifcrf.gov/relatedGenes.jsp?id=493) | [**Homo sapiens**](http://www.ncbi.nlm.nih.gov/Taxonomy/Browser/wwwtax.cgi?name=Homo%20sapiens) |
| **GOTERM_BP_DIRECT** | [neural retina development](http://www.ebi.ac.uk/QuickGO/GTerm?id=GO:0003407), [regulation of transcription from RNA polymerase II promoter](http://www.ebi.ac.uk/QuickGO/GTerm?id=GO:0006357), [cellular calcium ion homeostasis](http://www.ebi.ac.uk/QuickGO/GTerm?id=GO:0006874), [spermatogenesis](http://www.ebi.ac.uk/QuickGO/GTerm?id=GO:0007283), [negative regulation of gene expression](http://www.ebi.ac.uk/QuickGO/GTerm?id=GO:0010629), [negative regulation of nitric oxide mediated signal transduction](http://www.ebi.ac.uk/QuickGO/GTerm?id=GO:0010751), [urinary bladder smooth muscle contraction](http://www.ebi.ac.uk/QuickGO/GTerm?id=GO:0014832), [negative regulation of angiogenesis](http://www.ebi.ac.uk/QuickGO/GTerm?id=GO:0016525), [hippocampus development](http://www.ebi.ac.uk/QuickGO/GTerm?id=GO:0021766), [flagellated sperm motility](http://www.ebi.ac.uk/QuickGO/GTerm?id=GO:0030317), [positive regulation of peptidyl-serine phosphorylation](http://www.ebi.ac.uk/QuickGO/GTerm?id=GO:0033138), [ion transmembrane transport](http://www.ebi.ac.uk/QuickGO/GTerm?id=GO:0034220), [negative regulation of blood vessel endothelial cell migration](http://www.ebi.ac.uk/QuickGO/GTerm?id=GO:0043537), [negative regulation of nitric oxide biosynthetic process](http://www.ebi.ac.uk/QuickGO/GTerm?id=GO:0045019), [negative regulation of nitric-oxide synthase activity](http://www.ebi.ac.uk/QuickGO/GTerm?id=GO:0051001), [regulation of cytosolic calcium ion concentration](http://www.ebi.ac.uk/QuickGO/GTerm?id=GO:0051480), [response to hydrostatic pressure](http://www.ebi.ac.uk/QuickGO/GTerm?id=GO:0051599), [calcium ion transmembrane transport](http://www.ebi.ac.uk/QuickGO/GTerm?id=GO:0070588), [negative regulation of calcineurin-NFAT signaling cascade](http://www.ebi.ac.uk/QuickGO/GTerm?id=GO:0070885), [cellular response to epinephrine stimulus](http://www.ebi.ac.uk/QuickGO/GTerm?id=GO:0071872), [calcium ion transmembrane import into cytosol](http://www.ebi.ac.uk/QuickGO/GTerm?id=GO:0097553), [calcium ion import across plasma membrane](http://www.ebi.ac.uk/QuickGO/GTerm?id=GO:0098703), [negative regulation of the force of heart contraction](http://www.ebi.ac.uk/QuickGO/GTerm?id=GO:0098736), [negative regulation of arginine catabolic process](http://www.ebi.ac.uk/QuickGO/GTerm?id=GO:1900082), [calcium ion export](http://www.ebi.ac.uk/QuickGO/GTerm?id=GO:1901660), [negative regulation of peptidyl-cysteine S-nitrosylation](http://www.ebi.ac.uk/QuickGO/GTerm?id=GO:1902083), [regulation of sodium ion transmembrane transport](http://www.ebi.ac.uk/QuickGO/GTerm?id=GO:1902305), [negative regulation of cellular response to vascular endothelial growth factor stimulus](http://www.ebi.ac.uk/QuickGO/GTerm?id=GO:1902548), [regulation of cell cycle G1/S phase transition](http://www.ebi.ac.uk/QuickGO/GTerm?id=GO:1902806), [positive regulation of protein localization to plasma membrane](http://www.ebi.ac.uk/QuickGO/GTerm?id=GO:1903078), [negative regulation of cardiac muscle hypertrophy in response to stress](http://www.ebi.ac.uk/QuickGO/GTerm?id=GO:1903243), [negative regulation of citrulline biosynthetic process](http://www.ebi.ac.uk/QuickGO/GTerm?id=GO:1903249), [regulation of cardiac conduction](http://www.ebi.ac.uk/QuickGO/GTerm?id=GO:1903779), [cellular response to acetylcholine](http://www.ebi.ac.uk/QuickGO/GTerm?id=GO:1905145), [positive regulation of cAMP-dependent protein kinase activity](http://www.ebi.ac.uk/QuickGO/GTerm?id=GO:2000481), | | |
| **GOTERM_CC_DIRECT** | [plasma membrane](http://www.ebi.ac.uk/QuickGO/GTerm?id=GO:0005886), [integral component of plasma membrane](http://www.ebi.ac.uk/QuickGO/GTerm?id=GO:0005887), [caveola](http://www.ebi.ac.uk/QuickGO/GTerm?id=GO:0005901), [membrane](http://www.ebi.ac.uk/QuickGO/GTerm?id=GO:0016020), [integral component of membrane](http://www.ebi.ac.uk/QuickGO/GTerm?id=GO:0016021), [basolateral plasma membrane](http://www.ebi.ac.uk/QuickGO/GTerm?id=GO:0016323), [Z disc](http://www.ebi.ac.uk/QuickGO/GTerm?id=GO:0030018), [T-tubule](http://www.ebi.ac.uk/QuickGO/GTerm?id=GO:0030315), [macromolecular complex](http://www.ebi.ac.uk/QuickGO/GTerm?id=GO:0032991), [sperm flagellum](http://www.ebi.ac.uk/QuickGO/GTerm?id=GO:0036126), [neuron projection](http://www.ebi.ac.uk/QuickGO/GTerm?id=GO:0043005), [intracellular membrane-bounded organelle](http://www.ebi.ac.uk/QuickGO/GTerm?id=GO:0043231), [membrane raft](http://www.ebi.ac.uk/QuickGO/GTerm?id=GO:0045121), [sperm principal piece](http://www.ebi.ac.uk/QuickGO/GTerm?id=GO:0097228), [glutamatergic synapse](http://www.ebi.ac.uk/QuickGO/GTerm?id=GO:0098978), [integral component of presynaptic active zone membrane](http://www.ebi.ac.uk/QuickGO/GTerm?id=GO:0099059), | | |
| **GOTERM_MF_DIRECT** | [calcium-transporting ATPase activity](http://www.ebi.ac.uk/QuickGO/GTerm?id=GO:0005388), [protein binding](http://www.ebi.ac.uk/QuickGO/GTerm?id=GO:0005515), [calmodulin binding](http://www.ebi.ac.uk/QuickGO/GTerm?id=GO:0005516), [ATP binding](http://www.ebi.ac.uk/QuickGO/GTerm?id=GO:0005524), [calcium ion transmembrane transporter activity](http://www.ebi.ac.uk/QuickGO/GTerm?id=GO:0015085), [sodium channel regulator activity](http://www.ebi.ac.uk/QuickGO/GTerm?id=GO:0017080), [cation-transporting ATPase activity](http://www.ebi.ac.uk/QuickGO/GTerm?id=GO:0019829), [protein kinase binding](http://www.ebi.ac.uk/QuickGO/GTerm?id=GO:0019901), [PDZ domain binding](http://www.ebi.ac.uk/QuickGO/GTerm?id=GO:0030165), [protein phosphatase 2B binding](http://www.ebi.ac.uk/QuickGO/GTerm?id=GO:0030346), [nitric-oxide synthase inhibitor activity](http://www.ebi.ac.uk/QuickGO/GTerm?id=GO:0036487), [metal ion binding](http://www.ebi.ac.uk/QuickGO/GTerm?id=GO:0046872), [calcium-dependent protein binding](http://www.ebi.ac.uk/QuickGO/GTerm?id=GO:0048306), [nitric-oxide synthase binding](http://www.ebi.ac.uk/QuickGO/GTerm?id=GO:0050998), [scaffold protein binding](http://www.ebi.ac.uk/QuickGO/GTerm?id=GO:0097110), | | |
| **INTERPRO** | [Cation-transporting P-type ATPase](https://www.ebi.ac.uk/interpro/entry/InterPro/IPR001757), [Cation-transporting P-type ATPase, N-terminal](https://www.ebi.ac.uk/interpro/entry/InterPro/IPR004014), [Cation-transporting P-type ATPase, C-terminal](https://www.ebi.ac.uk/interpro/entry/InterPro/IPR006068), [Calcium-transporting P-type ATPase, subfamily IIB](https://www.ebi.ac.uk/interpro/entry/InterPro/IPR006408), [P-type ATPase, A domain](https://www.ebi.ac.uk/interpro/entry/InterPro/IPR008250), [P-type ATPase, phosphorylation site](https://www.ebi.ac.uk/interpro/entry/InterPro/IPR018303), [Calcium transporting P-type ATPase, C-terminal, plasma membrane](https://www.ebi.ac.uk/interpro/entry/InterPro/IPR022141), [HAD-like domain](https://www.ebi.ac.uk/interpro/entry/InterPro/IPR023214), [P-type ATPase, transmembrane domain](https://www.ebi.ac.uk/interpro/entry/InterPro/IPR023298), [P-type ATPase, cytoplasmic domain N](https://www.ebi.ac.uk/interpro/entry/InterPro/IPR023299), | | |
| **KEGG_PATHWAY** | [Calcium signaling pathway](https://david.ncifcrf.gov/kegg.jsp?path=hsa04020$Calcium%20signaling%20pathway&termId=520047945&source=kegg), [cGMP-PKG signaling pathway](https://david.ncifcrf.gov/kegg.jsp?path=hsa04022$cGMP-PKG%20signaling%20pathway&termId=520047946&source=kegg), [cAMP signaling pathway](https://david.ncifcrf.gov/kegg.jsp?path=hsa04024$cAMP%20signaling%20pathway&termId=520047947&source=kegg), [Adrenergic signaling in cardiomyocytes](https://david.ncifcrf.gov/kegg.jsp?path=hsa04261$Adrenergic%20signaling%20in%20cardiomyocytes&termId=520047983&source=kegg), [Aldosterone synthesis and secretion](https://david.ncifcrf.gov/kegg.jsp?path=hsa04925$Aldosterone%20synthesis%20and%20secretion&termId=520048058&source=kegg), [Endocrine and other factor-regulated calcium reabsorption](https://david.ncifcrf.gov/kegg.jsp?path=hsa04961$Endocrine%20and%20other%20factor-regulated%20calcium%20reabsorption&termId=520048073&source=kegg), [Salivary secretion](https://david.ncifcrf.gov/kegg.jsp?path=hsa04970$Salivary%20secretion&termId=520048077&source=kegg), [Pancreatic secretion](https://david.ncifcrf.gov/kegg.jsp?path=hsa04972$Pancreatic%20secretion&termId=520048079&source=kegg), [Mineral absorption](https://david.ncifcrf.gov/kegg.jsp?path=hsa04978$Mineral%20absorption&termId=520048085&source=kegg), | | |
| **SMART** | [SM00831](http://smart.embl.de/smart/do_annotation.pl?DOMAIN=SM00831), | | |
| **UP_KW_BIOLOGICAL_PROCESS** | [Calcium transport](http://www.uniprot.org/keywords/?query=KW-0109), [Ion transport](http://www.uniprot.org/keywords/?query=KW-0406), [Transport](http://www.uniprot.org/keywords/?query=KW-0813), | | |
| **UP_KW_CELLULAR_COMPONENT** | [Flagellum](http://www.uniprot.org/keywords/?query=KW-0282), [Membrane](http://www.uniprot.org/keywords/?query=KW-0472), [Cell projection](http://www.uniprot.org/keywords/?query=KW-0966), [Cilium](http://www.uniprot.org/keywords/?query=KW-0969), [Cell membrane](http://www.uniprot.org/keywords/?query=KW-1003), | | |
| **UP_KW_DOMAIN** | [Transmembrane](http://www.uniprot.org/keywords/?query=KW-0812), [Transmembrane helix](http://www.uniprot.org/keywords/?query=KW-1133), | | |
| **UP_KW_LIGAND** | [ATP-binding](http://www.uniprot.org/keywords/?query=KW-0067), [Calcium](http://www.uniprot.org/keywords/?query=KW-0106), [Magnesium](http://www.uniprot.org/keywords/?query=KW-0460), [Metal-binding](http://www.uniprot.org/keywords/?query=KW-0479), [Nucleotide-binding](http://www.uniprot.org/keywords/?query=KW-0547), | | |
| **UP_KW_MOLECULAR_FUNCTION** | [Calmodulin-binding](http://www.uniprot.org/keywords/?query=KW-0112), [Translocase](http://www.uniprot.org/keywords/?query=KW-1278), | | |
| **UP_KW_PTM** | [Phosphoprotein](http://www.uniprot.org/keywords/?query=KW-0597), | | |
| **UP_SEQ_FEATURE** | ACT_SITE:4-aspartylphosphate intermediate, COMPBIAS:Basic and acidic residues, DOMAIN:ATP_Ca_trans_C, DOMAIN:Cation_ATPase_C, DOMAIN:Cation_ATPase_N, METAL:Magnesium, MUTAGEN:D->Q: Strongly decreased calcium transport activity. Slowed decomposition of the phosphorylated intermediate., MUTAGEN:K->L: Decreased calcium transport activity., MUTAGEN:R->I: Mildly decreased calcium transport activity., MUTAGEN:R->K,D,L: Decreased calcium transport activity., MUTAGEN:V->P: Decreased calcium transport activity., REGION:Calmodulin-binding subdomain A, REGION:Calmodulin-binding subdomain B, REGION:Disordered, TOPO_DOM:Cytoplasmic, TOPO_DOM:Extracellular, TRANSMEM:Helical, | | |
| **ACAP2** | [**ArfGAP with coiled-coil, ankyrin repeat and PH domains 2(ACAP2)**](https://david.ncifcrf.gov/geneReportFull.jsp?rowids=23527) | [**Related Genes**](https://david.ncifcrf.gov/relatedGenes.jsp?id=23527) | [**Homo sapiens**](http://www.ncbi.nlm.nih.gov/Taxonomy/Browser/wwwtax.cgi?name=Homo%20sapiens) |
| **GOTERM_BP_DIRECT** | [actin filament-based process](http://www.ebi.ac.uk/QuickGO/GTerm?id=GO:0030029), [endocytic recycling](http://www.ebi.ac.uk/QuickGO/GTerm?id=GO:0032456), [positive regulation of GTPase activity](http://www.ebi.ac.uk/QuickGO/GTerm?id=GO:0043547), [regulation of catalytic activity](http://www.ebi.ac.uk/QuickGO/GTerm?id=GO:0050790), [cellular response to nerve growth factor stimulus](http://www.ebi.ac.uk/QuickGO/GTerm?id=GO:1990090), | | |
| **GOTERM_CC_DIRECT** | [ruffle](http://www.ebi.ac.uk/QuickGO/GTerm?id=GO:0001726), [endosome membrane](http://www.ebi.ac.uk/QuickGO/GTerm?id=GO:0010008), [membrane](http://www.ebi.ac.uk/QuickGO/GTerm?id=GO:0016020), | | |
| **GOTERM_MF_DIRECT** | [GTPase activator activity](http://www.ebi.ac.uk/QuickGO/GTerm?id=GO:0005096), [metal ion binding](http://www.ebi.ac.uk/QuickGO/GTerm?id=GO:0046872), | | |
| **INTERPRO** | [Arf GTPase activating protein](https://www.ebi.ac.uk/interpro/entry/InterPro/IPR001164), [Pleckstrin homology domain](https://www.ebi.ac.uk/interpro/entry/InterPro/IPR001849), [Ankyrin repeat](https://www.ebi.ac.uk/interpro/entry/InterPro/IPR002110), [Pleckstrin homology-like domain](https://www.ebi.ac.uk/interpro/entry/InterPro/IPR011993), [Ankyrin repeat-containing domain](https://www.ebi.ac.uk/interpro/entry/InterPro/IPR020683), [Arfaptin homology (AH) domain/BAR domain](https://www.ebi.ac.uk/interpro/entry/InterPro/IPR027267), | | |
| **KEGG_PATHWAY** | [Endocytosis](https://david.ncifcrf.gov/kegg.jsp?path=hsa04144$Endocytosis&termId=520047969&source=kegg), | | |
| **SMART** | [ArfGap](http://smart.embl.de/smart/do_annotation.pl?DOMAIN=SM00105), [PH](http://smart.embl.de/smart/do_annotation.pl?DOMAIN=SM00233), [ANK](http://smart.embl.de/smart/do_annotation.pl?DOMAIN=SM00248), | | |
| **UP_KW_CELLULAR_COMPONENT** | [Membrane](http://www.uniprot.org/keywords/?query=KW-0472), [Endosome](http://www.uniprot.org/keywords/?query=KW-0967), | | |
| **UP_KW_DOMAIN** | [ANK repeat](http://www.uniprot.org/keywords/?query=KW-0040), [Coiled coil](http://www.uniprot.org/keywords/?query=KW-0175), [Repeat](http://www.uniprot.org/keywords/?query=KW-0677), [Zinc-finger](http://www.uniprot.org/keywords/?query=KW-0863), | | |
| **UP_KW_LIGAND** | [Metal-binding](http://www.uniprot.org/keywords/?query=KW-0479), [Zinc](http://www.uniprot.org/keywords/?query=KW-0862), | | |
| **UP_KW_MOLECULAR_FUNCTION** | [GTPase activation](http://www.uniprot.org/keywords/?query=KW-0343), | | |
| **UP_KW_PTM** | [Phosphoprotein](http://www.uniprot.org/keywords/?query=KW-0597), | | |
| **UP_SEQ_FEATURE** | COMPBIAS:Polar residues, DOMAIN:Ankyrin_rpt-contain_dom, DOMAIN:Arf-GAP, DOMAIN:BAR, DOMAIN:PH, MUTAGEN:R->Q: Loss of GAP activity., REGION:Disordered, REPEAT:ANK, REPEAT:ANK 1, REPEAT:ANK 2, REPEAT:ANK 3, ZN_FING:C4-type, | | |
| **BAHCC1** | [**BAH domain and coiled-coil containing 1(BAHCC1)**](https://david.ncifcrf.gov/geneReportFull.jsp?rowids=57597) | [**Related Genes**](https://david.ncifcrf.gov/relatedGenes.jsp?id=57597) | [**Homo sapiens**](http://www.ncbi.nlm.nih.gov/Taxonomy/Browser/wwwtax.cgi?name=Homo%20sapiens) |
| **GOTERM_MF_DIRECT** | [chromatin binding](http://www.ebi.ac.uk/QuickGO/GTerm?id=GO:0003682), | | |
| **INTERPRO** | [Bromo adjacent homology (BAH) domain](https://www.ebi.ac.uk/interpro/entry/InterPro/IPR001025), | | |
| **SMART** | [BAH](http://smart.embl.de/smart/do_annotation.pl?DOMAIN=SM00439), | | |
| **UP_KW_DOMAIN** | [Coiled coil](http://www.uniprot.org/keywords/?query=KW-0175), | | |
| **UP_KW_PTM** | [Acetylation](http://www.uniprot.org/keywords/?query=KW-0007), [Phosphoprotein](http://www.uniprot.org/keywords/?query=KW-0597), | | |
| **UP_SEQ_FEATURE** | COMPBIAS:Acidic residues, COMPBIAS:Basic and acidic residues, COMPBIAS:Polar residues, COMPBIAS:Pro residues, DOMAIN:BAH, REGION:Disordered, | | |
| **BAIAP2** | [**BAR/IMD domain containing adaptor protein 2(BAIAP2)**](https://david.ncifcrf.gov/geneReportFull.jsp?rowids=10458) | [**Related Genes**](https://david.ncifcrf.gov/relatedGenes.jsp?id=10458) | [**Homo sapiens**](http://www.ncbi.nlm.nih.gov/Taxonomy/Browser/wwwtax.cgi?name=Homo%20sapiens) |
| **BIOCARTA** | [Rho cell motility signaling pathway](https://david.ncifcrf.gov/biocarta.jsp?path=h_rhoPathway$Rho%20cell%20motility%20signaling%20pathway&termId=30000262&source=biocarta), | | |
| **GOTERM_BP_DIRECT** | [plasma membrane organization](http://www.ebi.ac.uk/QuickGO/GTerm?id=GO:0007009), [axonogenesis](http://www.ebi.ac.uk/QuickGO/GTerm?id=GO:0007409), [brain development](http://www.ebi.ac.uk/QuickGO/GTerm?id=GO:0007420), [insulin receptor signaling pathway](http://www.ebi.ac.uk/QuickGO/GTerm?id=GO:0008286), [regulation of cell shape](http://www.ebi.ac.uk/QuickGO/GTerm?id=GO:0008360), [response to bacterium](http://www.ebi.ac.uk/QuickGO/GTerm?id=GO:0009617), [dendrite development](http://www.ebi.ac.uk/QuickGO/GTerm?id=GO:0016358), [positive regulation of actin filament polymerization](http://www.ebi.ac.uk/QuickGO/GTerm?id=GO:0030838), [regulation of actin cytoskeleton organization](http://www.ebi.ac.uk/QuickGO/GTerm?id=GO:0032956), [protein localization to synapse](http://www.ebi.ac.uk/QuickGO/GTerm?id=GO:0035418), [Fc-gamma receptor signaling pathway involved in phagocytosis](http://www.ebi.ac.uk/QuickGO/GTerm?id=GO:0038096), [vascular endothelial growth factor receptor signaling pathway](http://www.ebi.ac.uk/QuickGO/GTerm?id=GO:0048010), [regulation of synaptic plasticity](http://www.ebi.ac.uk/QuickGO/GTerm?id=GO:0048167), [actin filament bundle assembly](http://www.ebi.ac.uk/QuickGO/GTerm?id=GO:0051017), [actin crosslink formation](http://www.ebi.ac.uk/QuickGO/GTerm?id=GO:0051764), [positive regulation of dendritic spine morphogenesis](http://www.ebi.ac.uk/QuickGO/GTerm?id=GO:0061003), [cellular response to epidermal growth factor stimulus](http://www.ebi.ac.uk/QuickGO/GTerm?id=GO:0071364), [cell-cell adhesion](http://www.ebi.ac.uk/QuickGO/GTerm?id=GO:0098609), [modification of synaptic structure, modulating synaptic transmission](http://www.ebi.ac.uk/QuickGO/GTerm?id=GO:0099564), [cellular response to L-glutamate](http://www.ebi.ac.uk/QuickGO/GTerm?id=GO:1905232), [regulation of modification of postsynaptic actin cytoskeleton](http://www.ebi.ac.uk/QuickGO/GTerm?id=GO:1905274), [positive regulation of actin cytoskeleton reorganization](http://www.ebi.ac.uk/QuickGO/GTerm?id=GO:2000251), [positive regulation of excitatory postsynaptic potential](http://www.ebi.ac.uk/QuickGO/GTerm?id=GO:2000463), | | |
| **GOTERM_CC_DIRECT** | [ruffle](http://www.ebi.ac.uk/QuickGO/GTerm?id=GO:0001726), [nucleoplasm](http://www.ebi.ac.uk/QuickGO/GTerm?id=GO:0005654), [cytoplasm](http://www.ebi.ac.uk/QuickGO/GTerm?id=GO:0005737), [rough endoplasmic reticulum](http://www.ebi.ac.uk/QuickGO/GTerm?id=GO:0005791), [Golgi apparatus](http://www.ebi.ac.uk/QuickGO/GTerm?id=GO:0005794), [cytosol](http://www.ebi.ac.uk/QuickGO/GTerm?id=GO:0005829), [cytoskeleton](http://www.ebi.ac.uk/QuickGO/GTerm?id=GO:0005856), [microtubule](http://www.ebi.ac.uk/QuickGO/GTerm?id=GO:0005874), [plasma membrane](http://www.ebi.ac.uk/QuickGO/GTerm?id=GO:0005886), [adherens junction](http://www.ebi.ac.uk/QuickGO/GTerm?id=GO:0005912), [actin cytoskeleton](http://www.ebi.ac.uk/QuickGO/GTerm?id=GO:0015629), [membrane](http://www.ebi.ac.uk/QuickGO/GTerm?id=GO:0016020), [lamellipodium](http://www.ebi.ac.uk/QuickGO/GTerm?id=GO:0030027), [secretory granule](http://www.ebi.ac.uk/QuickGO/GTerm?id=GO:0030141), [filopodium](http://www.ebi.ac.uk/QuickGO/GTerm?id=GO:0030175), [neuronal cell body](http://www.ebi.ac.uk/QuickGO/GTerm?id=GO:0043025), [dendritic shaft](http://www.ebi.ac.uk/QuickGO/GTerm?id=GO:0043198), [neuron projection terminus](http://www.ebi.ac.uk/QuickGO/GTerm?id=GO:0044306), [excitatory synapse](http://www.ebi.ac.uk/QuickGO/GTerm?id=GO:0060076), [neuron projection branch point](http://www.ebi.ac.uk/QuickGO/GTerm?id=GO:0061845), [dendritic spine cytoplasm](http://www.ebi.ac.uk/QuickGO/GTerm?id=GO:0061846), [extracellular exosome](http://www.ebi.ac.uk/QuickGO/GTerm?id=GO:0070062), [synaptic membrane](http://www.ebi.ac.uk/QuickGO/GTerm?id=GO:0097060), [Schaffer collateral - CA1 synapse](http://www.ebi.ac.uk/QuickGO/GTerm?id=GO:0098685), [glutamatergic synapse](http://www.ebi.ac.uk/QuickGO/GTerm?id=GO:0098978), [postsynaptic density, intracellular component](http://www.ebi.ac.uk/QuickGO/GTerm?id=GO:0099092), [presynaptic cytosol](http://www.ebi.ac.uk/QuickGO/GTerm?id=GO:0099523), [postsynaptic cytosol](http://www.ebi.ac.uk/QuickGO/GTerm?id=GO:0099524), | | |
| **GOTERM_MF_DIRECT** | [transcription cofactor binding](http://www.ebi.ac.uk/QuickGO/GTerm?id=GO:0001221), [protein binding](http://www.ebi.ac.uk/QuickGO/GTerm?id=GO:0005515), [protein C-terminus binding](http://www.ebi.ac.uk/QuickGO/GTerm?id=GO:0008022), [cytoskeletal adaptor activity](http://www.ebi.ac.uk/QuickGO/GTerm?id=GO:0008093), [PDZ domain binding](http://www.ebi.ac.uk/QuickGO/GTerm?id=GO:0030165), [identical protein binding](http://www.ebi.ac.uk/QuickGO/GTerm?id=GO:0042802), [proline-rich region binding](http://www.ebi.ac.uk/QuickGO/GTerm?id=GO:0070064), [scaffold protein binding](http://www.ebi.ac.uk/QuickGO/GTerm?id=GO:0097110), [cadherin binding involved in cell-cell adhesion](http://www.ebi.ac.uk/QuickGO/GTerm?id=GO:0098641), | | |
| **INTERPRO** | [Src homology-3 domain](https://www.ebi.ac.uk/interpro/entry/InterPro/IPR001452), [IRSp53/MIM homology domain (IMD)](https://www.ebi.ac.uk/interpro/entry/InterPro/IPR013606), [Arfaptin homology (AH) domain/BAR domain](https://www.ebi.ac.uk/interpro/entry/InterPro/IPR027267), | | |
| **KEGG_PATHWAY** | [Adherens junction](https://david.ncifcrf.gov/kegg.jsp?path=hsa04520$Adherens%20junction&termId=520047998&source=kegg), [Regulation of actin cytoskeleton](https://david.ncifcrf.gov/kegg.jsp?path=hsa04810$Regulation%20of%20actin%20cytoskeleton&termId=520048042&source=kegg), [Pathogenic Escherichia coli infection](https://david.ncifcrf.gov/kegg.jsp?path=hsa05130$Pathogenic%20Escherichia%20coli%20infection&termId=520048102&source=kegg), [Yersinia infection](https://david.ncifcrf.gov/kegg.jsp?path=hsa05135$Yersinia%20infection&termId=520048107&source=kegg), | | |
| **SMART** | [SH3](http://smart.embl.de/smart/do_annotation.pl?DOMAIN=SM00326), | | |
| **UP_KW_CELLULAR_COMPONENT** | [Cytoskeleton](http://www.uniprot.org/keywords/?query=KW-0206), [Membrane](http://www.uniprot.org/keywords/?query=KW-0472), [Cytoplasm](http://www.uniprot.org/keywords/?query=KW-0963), [Cell projection](http://www.uniprot.org/keywords/?query=KW-0966), | | |
| **UP_KW_DOMAIN** | [Coiled coil](http://www.uniprot.org/keywords/?query=KW-0175), [SH3 domain](http://www.uniprot.org/keywords/?query=KW-0728), | | |
| **UP_KW_PTM** | [Phosphoprotein](http://www.uniprot.org/keywords/?query=KW-0597), | | |
| **UP_SEQ_FEATURE** | COMPBIAS:Polar residues, DOMAIN:IMD, DOMAIN:SH3, MUTAGEN:F->A: Loss of interaction with ENAH and no induction of filopodia; when associated with A-428., MUTAGEN:I->N: Loss of interaction with CDC42. Loss of stimulation of neurite growth., MUTAGEN:K->E: Abolishes actin-bundling and filopodia formation; when associated with E-142; E-143 and E146., MUTAGEN:K->E: Abolishes actin-bundling and filopodia formation; when associated with E-142; E-143 and E147., MUTAGEN:K->E: Abolishes actin-bundling and filopodia formation; when associated with E-142; E-146 and E147., MUTAGEN:K->E: Abolishes actin-bundling and filopodia formation; when associated with E-143; E-146 and E147., MUTAGEN:P->A: Loss of interaction with ENAH and no induction of filopodia; when associated with A-427., MUTAGEN:W->G: Impairs the SH3 domain and abolishes the interaction with EPS8., REGION:Disordered, | | |
| **BEND5** | [**BEN domain containing 5(BEND5)**](https://david.ncifcrf.gov/geneReportFull.jsp?rowids=79656) | [**Related Genes**](https://david.ncifcrf.gov/relatedGenes.jsp?id=79656) | [**Homo sapiens**](http://www.ncbi.nlm.nih.gov/Taxonomy/Browser/wwwtax.cgi?name=Homo%20sapiens) |
| **GOTERM_BP_DIRECT** | [negative regulation of transcription, DNA-templated](http://www.ebi.ac.uk/QuickGO/GTerm?id=GO:0045892), | | |
| **GOTERM_CC_DIRECT** | [Golgi apparatus](http://www.ebi.ac.uk/QuickGO/GTerm?id=GO:0005794), | | |
| **GOTERM_MF_DIRECT** | [DNA binding](http://www.ebi.ac.uk/QuickGO/GTerm?id=GO:0003677), [protein binding](http://www.ebi.ac.uk/QuickGO/GTerm?id=GO:0005515), | | |
| **INTERPRO** | [BEN domain](https://www.ebi.ac.uk/interpro/entry/InterPro/IPR018379), | | |
| **SMART** | [SM01025](http://smart.embl.de/smart/do_annotation.pl?DOMAIN=SM01025), | | |
| **UP_KW_BIOLOGICAL_PROCESS** | [Transcription](http://www.uniprot.org/keywords/?query=KW-0804), [Transcription regulation](http://www.uniprot.org/keywords/?query=KW-0805), | | |
| **UP_KW_DOMAIN** | [Coiled coil](http://www.uniprot.org/keywords/?query=KW-0175), | | |
| **UP_KW_MOLECULAR_FUNCTION** | [DNA-binding](http://www.uniprot.org/keywords/?query=KW-0238), [Repressor](http://www.uniprot.org/keywords/?query=KW-0678), | | |
| **UP_KW_PTM** | [Acetylation](http://www.uniprot.org/keywords/?query=KW-0007), [Ubl conjugation](http://www.uniprot.org/keywords/?query=KW-0832), [Isopeptide bond](http://www.uniprot.org/keywords/?query=KW-1017), | | |
| **UP_SEQ_FEATURE** | CROSSLNK:Glycyl lysine isopeptide (Lys-Gly) (interchain with G-Cter in SUMO2), DOMAIN:BEN, | | |
| **CTBP2** | [**C-terminal binding protein 2(CTBP2)**](https://david.ncifcrf.gov/geneReportFull.jsp?rowids=1488) | [**Related Genes**](https://david.ncifcrf.gov/relatedGenes.jsp?id=1488) | [**Homo sapiens**](http://www.ncbi.nlm.nih.gov/Taxonomy/Browser/wwwtax.cgi?name=Homo%20sapiens) |
| **GOTERM_BP_DIRECT** | [negative regulation of transcription from RNA polymerase II promoter](http://www.ebi.ac.uk/QuickGO/GTerm?id=GO:0000122), [negative regulation of cell proliferation](http://www.ebi.ac.uk/QuickGO/GTerm?id=GO:0008285), [synaptic vesicle docking](http://www.ebi.ac.uk/QuickGO/GTerm?id=GO:0016081), [viral genome replication](http://www.ebi.ac.uk/QuickGO/GTerm?id=GO:0019079), [positive regulation of chromatin binding](http://www.ebi.ac.uk/QuickGO/GTerm?id=GO:0035563), [negative regulation of transcription, DNA-templated](http://www.ebi.ac.uk/QuickGO/GTerm?id=GO:0045892), [positive regulation of transcription from RNA polymerase II promoter](http://www.ebi.ac.uk/QuickGO/GTerm?id=GO:0045944), [positive regulation of retinoic acid receptor signaling pathway](http://www.ebi.ac.uk/QuickGO/GTerm?id=GO:0048386), [maintenance of presynaptic active zone structure](http://www.ebi.ac.uk/QuickGO/GTerm?id=GO:0048790), [white fat cell differentiation](http://www.ebi.ac.uk/QuickGO/GTerm?id=GO:0050872), [cellular response to leukemia inhibitory factor](http://www.ebi.ac.uk/QuickGO/GTerm?id=GO:1990830), | | |
| **GOTERM_CC_DIRECT** | [nucleus](http://www.ebi.ac.uk/QuickGO/GTerm?id=GO:0005634), [transcriptional repressor complex](http://www.ebi.ac.uk/QuickGO/GTerm?id=GO:0017053), [photoreceptor ribbon synapse](http://www.ebi.ac.uk/QuickGO/GTerm?id=GO:0098684), [presynaptic active zone cytoplasmic component](http://www.ebi.ac.uk/QuickGO/GTerm?id=GO:0098831), [glutamatergic synapse](http://www.ebi.ac.uk/QuickGO/GTerm?id=GO:0098978), [GABA-ergic synapse](http://www.ebi.ac.uk/QuickGO/GTerm?id=GO:0098982), [presynaptic cytosol](http://www.ebi.ac.uk/QuickGO/GTerm?id=GO:0099523), | | |
| **GOTERM_MF_DIRECT** | [transcription corepressor binding](http://www.ebi.ac.uk/QuickGO/GTerm?id=GO:0001222), [RNA polymerase II transcription corepressor binding](http://www.ebi.ac.uk/QuickGO/GTerm?id=GO:0001226), [chromatin binding](http://www.ebi.ac.uk/QuickGO/GTerm?id=GO:0003682), [transcription coactivator activity](http://www.ebi.ac.uk/QuickGO/GTerm?id=GO:0003713), [transcription corepressor activity](http://www.ebi.ac.uk/QuickGO/GTerm?id=GO:0003714), [protein binding](http://www.ebi.ac.uk/QuickGO/GTerm?id=GO:0005515), [oxidoreductase activity, acting on the CH-OH group of donors, NAD or NADP as acceptor](http://www.ebi.ac.uk/QuickGO/GTerm?id=GO:0016616), [protein kinase binding](http://www.ebi.ac.uk/QuickGO/GTerm?id=GO:0019901), [identical protein binding](http://www.ebi.ac.uk/QuickGO/GTerm?id=GO:0042802), [retinoic acid receptor binding](http://www.ebi.ac.uk/QuickGO/GTerm?id=GO:0042974), [macromolecular complex binding](http://www.ebi.ac.uk/QuickGO/GTerm?id=GO:0044877), [NAD binding](http://www.ebi.ac.uk/QuickGO/GTerm?id=GO:0051287), [structural constituent of presynaptic active zone](http://www.ebi.ac.uk/QuickGO/GTerm?id=GO:0098882), | | |
| **INTERPRO** | [D-isomer specific 2-hydroxyacid dehydrogenase, catalytic domain](https://www.ebi.ac.uk/interpro/entry/InterPro/IPR006139), [D-isomer specific 2-hydroxyacid dehydrogenase, NAD-binding](https://www.ebi.ac.uk/interpro/entry/InterPro/IPR006140), | | |
| **KEGG_PATHWAY** | [Wnt signaling pathway](https://david.ncifcrf.gov/kegg.jsp?path=hsa04310$Wnt%20signaling%20pathway&termId=520047985&source=kegg), [Notch signaling pathway](https://david.ncifcrf.gov/kegg.jsp?path=hsa04330$Notch%20signaling%20pathway&termId=520047986&source=kegg), [Pathways in cancer](https://david.ncifcrf.gov/kegg.jsp?path=hsa05200$Pathways%20in%20cancer&termId=520048128&source=kegg), [Chronic myeloid leukemia](https://david.ncifcrf.gov/kegg.jsp?path=hsa05220$Chronic%20myeloid%20leukemia&termId=520048146&source=kegg), | | |
| **UP_KW_BIOLOGICAL_PROCESS** | [Differentiation](http://www.uniprot.org/keywords/?query=KW-0221), [Transcription](http://www.uniprot.org/keywords/?query=KW-0804), [Transcription regulation](http://www.uniprot.org/keywords/?query=KW-0805), [Host-virus interaction](http://www.uniprot.org/keywords/?query=KW-0945), | | |
| **UP_KW_CELLULAR_COMPONENT** | [Nucleus](http://www.uniprot.org/keywords/?query=KW-0539), [Synapse](http://www.uniprot.org/keywords/?query=KW-0770), [Cell junction](http://www.uniprot.org/keywords/?query=KW-0965), | | |
| **UP_KW_LIGAND** | [NAD](http://www.uniprot.org/keywords/?query=KW-0520), | | |
| **UP_KW_MOLECULAR_FUNCTION** | [Oxidoreductase](http://www.uniprot.org/keywords/?query=KW-0560), [Repressor](http://www.uniprot.org/keywords/?query=KW-0678), | | |
| **UP_KW_PTM** | [Methylation](http://www.uniprot.org/keywords/?query=KW-0488), [Phosphoprotein](http://www.uniprot.org/keywords/?query=KW-0597), | | |
| **UP_SEQ_FEATURE** | ACT_SITE:Proton donor, BINDING:NAD, COMPBIAS:Polar residues, DOMAIN:2-Hacid_dh, NP_BIND:NAD, REGION:Disordered, | | |
| **CNOT1** | [**CCR4-NOT transcription complex subunit 1(CNOT1)**](https://david.ncifcrf.gov/geneReportFull.jsp?rowids=23019) | [**Related Genes**](https://david.ncifcrf.gov/relatedGenes.jsp?id=23019) | [**Homo sapiens**](http://www.ncbi.nlm.nih.gov/Taxonomy/Browser/wwwtax.cgi?name=Homo%20sapiens) |
| **GOTERM_BP_DIRECT** | [negative regulation of transcription from RNA polymerase II promoter](http://www.ebi.ac.uk/QuickGO/GTerm?id=GO:0000122), [nuclear-transcribed mRNA catabolic process, deadenylation-dependent decay](http://www.ebi.ac.uk/QuickGO/GTerm?id=GO:0000288), [nuclear-transcribed mRNA poly(A) tail shortening](http://www.ebi.ac.uk/QuickGO/GTerm?id=GO:0000289), [trophectodermal cell differentiation](http://www.ebi.ac.uk/QuickGO/GTerm?id=GO:0001829), [regulation of translation](http://www.ebi.ac.uk/QuickGO/GTerm?id=GO:0006417), [DNA damage response, signal transduction by p53 class mediator resulting in cell cycle arrest](http://www.ebi.ac.uk/QuickGO/GTerm?id=GO:0006977), [positive regulation of cytoplasmic mRNA processing body assembly](http://www.ebi.ac.uk/QuickGO/GTerm?id=GO:0010606), [negative regulation of translation](http://www.ebi.ac.uk/QuickGO/GTerm?id=GO:0017148), [negative regulation of intracellular estrogen receptor signaling pathway](http://www.ebi.ac.uk/QuickGO/GTerm?id=GO:0033147), [gene silencing by miRNA](http://www.ebi.ac.uk/QuickGO/GTerm?id=GO:0035195), [negative regulation of retinoic acid receptor signaling pathway](http://www.ebi.ac.uk/QuickGO/GTerm?id=GO:0048387), [positive regulation of nuclear-transcribed mRNA poly(A) tail shortening](http://www.ebi.ac.uk/QuickGO/GTerm?id=GO:0060213), [positive regulation of mRNA catabolic process](http://www.ebi.ac.uk/QuickGO/GTerm?id=GO:0061014), [RNA phosphodiester bond hydrolysis, exonucleolytic](http://www.ebi.ac.uk/QuickGO/GTerm?id=GO:0090503), [positive regulation of nuclear-transcribed mRNA catabolic process, deadenylation-dependent decay](http://www.ebi.ac.uk/QuickGO/GTerm?id=GO:1900153), [regulation of stem cell population maintenance](http://www.ebi.ac.uk/QuickGO/GTerm?id=GO:2000036), | | |
| **GOTERM_CC_DIRECT** | [P-body](http://www.ebi.ac.uk/QuickGO/GTerm?id=GO:0000932), [extracellular space](http://www.ebi.ac.uk/QuickGO/GTerm?id=GO:0005615), [nucleus](http://www.ebi.ac.uk/QuickGO/GTerm?id=GO:0005634), [peroxisomal membrane](http://www.ebi.ac.uk/QuickGO/GTerm?id=GO:0005778), [cytosol](http://www.ebi.ac.uk/QuickGO/GTerm?id=GO:0005829), [membrane](http://www.ebi.ac.uk/QuickGO/GTerm?id=GO:0016020), [CCR4-NOT complex](http://www.ebi.ac.uk/QuickGO/GTerm?id=GO:0030014), [CCR4-NOT core complex](http://www.ebi.ac.uk/QuickGO/GTerm?id=GO:0030015), | | |
| **GOTERM_MF_DIRECT** | [RNA binding](http://www.ebi.ac.uk/QuickGO/GTerm?id=GO:0003723), [poly(A)-specific ribonuclease activity](http://www.ebi.ac.uk/QuickGO/GTerm?id=GO:0004535), [protein binding](http://www.ebi.ac.uk/QuickGO/GTerm?id=GO:0005515), [protein domain specific binding](http://www.ebi.ac.uk/QuickGO/GTerm?id=GO:0019904), [estrogen receptor binding](http://www.ebi.ac.uk/QuickGO/GTerm?id=GO:0030331), [retinoic acid receptor binding](http://www.ebi.ac.uk/QuickGO/GTerm?id=GO:0042974), [binding, bridging](http://www.ebi.ac.uk/QuickGO/GTerm?id=GO:0060090), [armadillo repeat domain binding](http://www.ebi.ac.uk/QuickGO/GTerm?id=GO:0070016), | | |
| **INTERPRO** | [CCR4-Not complex component, Not1, C-terminal](https://www.ebi.ac.uk/interpro/entry/InterPro/IPR007196), [MIF4-like, type 1/2/3](https://www.ebi.ac.uk/interpro/entry/InterPro/IPR016021), [CCR4-Not complex, Not1 subunit, domain of unknown function DUF3819](https://www.ebi.ac.uk/interpro/entry/InterPro/IPR024557), | | |
| **KEGG_PATHWAY** | [RNA degradation](https://david.ncifcrf.gov/kegg.jsp?path=hsa03018$RNA%20degradation&termId=520047927&source=kegg), | | |
| **OMIM_DISEASE** | [Holoprosencephaly 12, with or without pancreatic agenesis](http://omim.org/entry/618500), [Vissers-Bodmer syndrome](http://omim.org/entry/619033), | | |
| **UP_KW_BIOLOGICAL_PROCESS** | [Transcription](http://www.uniprot.org/keywords/?query=KW-0804), [Transcription regulation](http://www.uniprot.org/keywords/?query=KW-0805), [Translation regulation](http://www.uniprot.org/keywords/?query=KW-0810), [RNA-mediated gene silencing](http://www.uniprot.org/keywords/?query=KW-0943), | | |
| **UP_KW_CELLULAR_COMPONENT** | [Nucleus](http://www.uniprot.org/keywords/?query=KW-0539), [Cytoplasm](http://www.uniprot.org/keywords/?query=KW-0963), | | |
| **UP_KW_DISEASE** | [Disease variant](http://www.uniprot.org/keywords/?query=KW-0225), [Holoprosencephaly](http://www.uniprot.org/keywords/?query=KW-0370), [Mental retardation](http://www.uniprot.org/keywords/?query=KW-0991), [Autism spectrum disorder](http://www.uniprot.org/keywords/?query=KW-1268), | | |
| **UP_KW_MOLECULAR_FUNCTION** | [Developmental protein](http://www.uniprot.org/keywords/?query=KW-0217), [Repressor](http://www.uniprot.org/keywords/?query=KW-0678), [Developmental protein](http://www.uniprot.org/keywords/?query=KW-9996), | | |
| **UP_KW_PTM** | [Phosphoprotein](http://www.uniprot.org/keywords/?query=KW-0597), | | |
| **UP_SEQ_FEATURE** | COMPBIAS:Basic and acidic residues, COMPBIAS:Polar residues, DOMAIN:CNOT1_CAF1_bind, DOMAIN:CNOT1_HEAT, DOMAIN:CNOT1_TTP_bind, DOMAIN:DUF3819, DOMAIN:Not1, MOTIF:LXXLL, MUTAGEN:H->Y: Impairs interaction with CNOT7; when associated with E-1208 and E-1218., MUTAGEN:K->E: Impairs interaction with CNOT7; when associated with E-1208 and Y-1212., MUTAGEN:K->E: Impairs interaction with CNOT7; when associated with Y-1212 and E-1218., MUTAGEN:P->Y: Abolishes interaction with CNOT7; when associated with Y-1209., MUTAGEN:P->Y: Abolishes interaction with CNOT7; when associated with Y-1257., MUTAGEN:V->R: Abolishes interaction with CNOT7., REGION:Disordered, REGION:Interaction with CNOT6, CNOT6L, CNOT7 and CNOT8, REGION:Interaction with ZFP36, | | |
| **CD101** | [**CD101 molecule(CD101)**](https://david.ncifcrf.gov/geneReportFull.jsp?rowids=9398) | [**Related Genes**](https://david.ncifcrf.gov/relatedGenes.jsp?id=9398) | [**Homo sapiens**](http://www.ncbi.nlm.nih.gov/Taxonomy/Browser/wwwtax.cgi?name=Homo%20sapiens) |
| **GOTERM_BP_DIRECT** | [positive regulation of myeloid leukocyte differentiation](http://www.ebi.ac.uk/QuickGO/GTerm?id=GO:0002763), [cell surface receptor signaling pathway](http://www.ebi.ac.uk/QuickGO/GTerm?id=GO:0007166), | | |
| **GOTERM_CC_DIRECT** | [plasma membrane](http://www.ebi.ac.uk/QuickGO/GTerm?id=GO:0005886), [integral component of membrane](http://www.ebi.ac.uk/QuickGO/GTerm?id=GO:0016021), [extracellular exosome](http://www.ebi.ac.uk/QuickGO/GTerm?id=GO:0070062), | | |
| **GOTERM_MF_DIRECT** | [hydrolase activity, acting on carbon-nitrogen (but not peptide) bonds, in cyclic amides](http://www.ebi.ac.uk/QuickGO/GTerm?id=GO:0016812), | | |
| **INTERPRO** | [Dihydroorotase, conserved site](https://www.ebi.ac.uk/interpro/entry/InterPro/IPR002195), [Immunoglobulin subtype](https://www.ebi.ac.uk/interpro/entry/InterPro/IPR003599), [Immunoglobulin-like domain](https://www.ebi.ac.uk/interpro/entry/InterPro/IPR007110), [Immunoglobulin I-set](https://www.ebi.ac.uk/interpro/entry/InterPro/IPR013098), [Immunoglobulin V-set](https://www.ebi.ac.uk/interpro/entry/InterPro/IPR013106), [Immunoglobulin-like fold](https://www.ebi.ac.uk/interpro/entry/InterPro/IPR013783), | | |
| **SMART** | [IGv](http://smart.embl.de/smart/do_annotation.pl?DOMAIN=SM00406), [IG](http://smart.embl.de/smart/do_annotation.pl?DOMAIN=SM00409), | | |
| **UP_KW_CELLULAR_COMPONENT** | [Membrane](http://www.uniprot.org/keywords/?query=KW-0472), | | |
| **UP_KW_DOMAIN** | [Immunoglobulin domain](http://www.uniprot.org/keywords/?query=KW-0393), [Repeat](http://www.uniprot.org/keywords/?query=KW-0677), [Signal](http://www.uniprot.org/keywords/?query=KW-0732), [Transmembrane](http://www.uniprot.org/keywords/?query=KW-0812), [Transmembrane helix](http://www.uniprot.org/keywords/?query=KW-1133), | | |
| **UP_KW_PTM** | [Glycoprotein](http://www.uniprot.org/keywords/?query=KW-0325), [Disulfide bond](http://www.uniprot.org/keywords/?query=KW-1015), | | |
| **UP_SEQ_FEATURE** | CARBOHYD:N-linked (GlcNAc...) asparagine, DOMAIN:IG, DOMAIN:Ig-like, DOMAIN:Ig-like C2-type 1, DOMAIN:Ig-like C2-type 2, DOMAIN:Ig-like C2-type 3, DOMAIN:Ig-like C2-type 4, DOMAIN:Ig-like C2-type 5, DOMAIN:Ig-like C2-type 6, DOMAIN:Ig-like C2-type 7, MOTIF:EWI motif, TOPO_DOM:Cytoplasmic, TOPO_DOM:Extracellular, TRANSMEM:Helical, | | |
| **CDC42BPB** | [**CDC42 binding protein kinase beta(CDC42BPB)**](https://david.ncifcrf.gov/geneReportFull.jsp?rowids=9578) | [**Related Genes**](https://david.ncifcrf.gov/relatedGenes.jsp?id=9578) | [**Homo sapiens**](http://www.ncbi.nlm.nih.gov/Taxonomy/Browser/wwwtax.cgi?name=Homo%20sapiens) |
| **GOTERM_BP_DIRECT** | [protein phosphorylation](http://www.ebi.ac.uk/QuickGO/GTerm?id=GO:0006468), [cytoskeleton organization](http://www.ebi.ac.uk/QuickGO/GTerm?id=GO:0007010), [establishment or maintenance of cell polarity](http://www.ebi.ac.uk/QuickGO/GTerm?id=GO:0007163), [signal transduction](http://www.ebi.ac.uk/QuickGO/GTerm?id=GO:0007165), [cell migration](http://www.ebi.ac.uk/QuickGO/GTerm?id=GO:0016477), [peptidyl-threonine phosphorylation](http://www.ebi.ac.uk/QuickGO/GTerm?id=GO:0018107), [actin cytoskeleton organization](http://www.ebi.ac.uk/QuickGO/GTerm?id=GO:0030036), [actomyosin structure organization](http://www.ebi.ac.uk/QuickGO/GTerm?id=GO:0031032), [actin cytoskeleton reorganization](http://www.ebi.ac.uk/QuickGO/GTerm?id=GO:0031532), [intracellular signal transduction](http://www.ebi.ac.uk/QuickGO/GTerm?id=GO:0035556), | | |
| **GOTERM_CC_DIRECT** | [cytoplasm](http://www.ebi.ac.uk/QuickGO/GTerm?id=GO:0005737), [cytosol](http://www.ebi.ac.uk/QuickGO/GTerm?id=GO:0005829), [cytoskeleton](http://www.ebi.ac.uk/QuickGO/GTerm?id=GO:0005856), [plasma membrane](http://www.ebi.ac.uk/QuickGO/GTerm?id=GO:0005886), [cell-cell junction](http://www.ebi.ac.uk/QuickGO/GTerm?id=GO:0005911), [membrane](http://www.ebi.ac.uk/QuickGO/GTerm?id=GO:0016020), [lamellipodium](http://www.ebi.ac.uk/QuickGO/GTerm?id=GO:0030027), [cell leading edge](http://www.ebi.ac.uk/QuickGO/GTerm?id=GO:0031252), [actomyosin](http://www.ebi.ac.uk/QuickGO/GTerm?id=GO:0042641), [extracellular exosome](http://www.ebi.ac.uk/QuickGO/GTerm?id=GO:0070062), | | |
| **GOTERM_MF_DIRECT** | [magnesium ion binding](http://www.ebi.ac.uk/QuickGO/GTerm?id=GO:0000287), [protein kinase activity](http://www.ebi.ac.uk/QuickGO/GTerm?id=GO:0004672), [protein serine/threonine kinase activity](http://www.ebi.ac.uk/QuickGO/GTerm?id=GO:0004674), [protein serine/threonine/tyrosine kinase activity](http://www.ebi.ac.uk/QuickGO/GTerm?id=GO:0004712), [ATP binding](http://www.ebi.ac.uk/QuickGO/GTerm?id=GO:0005524), [small GTPase binding](http://www.ebi.ac.uk/QuickGO/GTerm?id=GO:0031267), [macromolecular complex binding](http://www.ebi.ac.uk/QuickGO/GTerm?id=GO:0044877), | | |
| **INTERPRO** | [PAK-box/P21-Rho-binding](https://www.ebi.ac.uk/interpro/entry/InterPro/IPR000095), [Protein kinase, catalytic domain](https://www.ebi.ac.uk/interpro/entry/InterPro/IPR000719), [AGC-kinase, C-terminal](https://www.ebi.ac.uk/interpro/entry/InterPro/IPR000961), [Citron-like](https://www.ebi.ac.uk/interpro/entry/InterPro/IPR001180), [Pleckstrin homology domain](https://www.ebi.ac.uk/interpro/entry/InterPro/IPR001849), [Protein kinase C-like, phorbol ester/diacylglycerol binding](https://www.ebi.ac.uk/interpro/entry/InterPro/IPR002219), [Serine/threonine-protein kinase, active site](https://www.ebi.ac.uk/interpro/entry/InterPro/IPR008271), [Protein kinase-like domain](https://www.ebi.ac.uk/interpro/entry/InterPro/IPR011009), [Pleckstrin homology-like domain](https://www.ebi.ac.uk/interpro/entry/InterPro/IPR011993), [Myotonic dystrophy protein kinase, coiled coil](https://www.ebi.ac.uk/interpro/entry/InterPro/IPR014930), [Protein kinase, ATP binding site](https://www.ebi.ac.uk/interpro/entry/InterPro/IPR017441), [Diacylglycerol/phorbol-ester binding](https://www.ebi.ac.uk/interpro/entry/InterPro/IPR020454), | | |
| **SMART** | [CNH](http://smart.embl.de/smart/do_annotation.pl?DOMAIN=SM00036), [C1](http://smart.embl.de/smart/do_annotation.pl?DOMAIN=SM00109), [S_TK_X](http://smart.embl.de/smart/do_annotation.pl?DOMAIN=SM00133), [S_TKc](http://smart.embl.de/smart/do_annotation.pl?DOMAIN=SM00220), [PH](http://smart.embl.de/smart/do_annotation.pl?DOMAIN=SM00233), [PBD](http://smart.embl.de/smart/do_annotation.pl?DOMAIN=SM00285), | | |
| **UP_KW_CELLULAR_COMPONENT** | [Membrane](http://www.uniprot.org/keywords/?query=KW-0472), [Cytoplasm](http://www.uniprot.org/keywords/?query=KW-0963), [Cell junction](http://www.uniprot.org/keywords/?query=KW-0965), [Cell projection](http://www.uniprot.org/keywords/?query=KW-0966), [Cell membrane](http://www.uniprot.org/keywords/?query=KW-1003), | | |
| **UP_KW_DOMAIN** | [Coiled coil](http://www.uniprot.org/keywords/?query=KW-0175), [Zinc-finger](http://www.uniprot.org/keywords/?query=KW-0863), | | |
| **UP_KW_LIGAND** | [ATP-binding](http://www.uniprot.org/keywords/?query=KW-0067), [Magnesium](http://www.uniprot.org/keywords/?query=KW-0460), [Metal-binding](http://www.uniprot.org/keywords/?query=KW-0479), [Nucleotide-binding](http://www.uniprot.org/keywords/?query=KW-0547), [Zinc](http://www.uniprot.org/keywords/?query=KW-0862), | | |
| **UP_KW_MOLECULAR_FUNCTION** | [Kinase](http://www.uniprot.org/keywords/?query=KW-0418), [Serine/threonine-protein kinase](http://www.uniprot.org/keywords/?query=KW-0723), [Transferase](http://www.uniprot.org/keywords/?query=KW-0808), | | |
| **UP_KW_PTM** | [Methylation](http://www.uniprot.org/keywords/?query=KW-0488), [Phosphoprotein](http://www.uniprot.org/keywords/?query=KW-0597), | | |
| **UP_SEQ_FEATURE** | ACT_SITE:Proton acceptor, BINDING:ATP, COMPBIAS:Basic and acidic residues, COMPBIAS:Polar residues, DOMAIN:AGC-kinase C-terminal, DOMAIN:CNH, DOMAIN:CRIB, DOMAIN:PH, DOMAIN:Phorbol-ester/DAG-type, DOMAIN:Protein kinase, NP_BIND:ATP, REGION:Disordered, ZN_FING:Phorbol-ester/DAG-type, | | |
| **CDC42EP3** | [**CDC42 effector protein 3(CDC42EP3)**](https://david.ncifcrf.gov/geneReportFull.jsp?rowids=10602) | [**Related Genes**](https://david.ncifcrf.gov/relatedGenes.jsp?id=10602) | [**Homo sapiens**](http://www.ncbi.nlm.nih.gov/Taxonomy/Browser/wwwtax.cgi?name=Homo%20sapiens) |
| **GOTERM_BP_DIRECT** | [signal transduction](http://www.ebi.ac.uk/QuickGO/GTerm?id=GO:0007165), [Rho protein signal transduction](http://www.ebi.ac.uk/QuickGO/GTerm?id=GO:0007266), [regulation of cell shape](http://www.ebi.ac.uk/QuickGO/GTerm?id=GO:0008360), [positive regulation of actin filament polymerization](http://www.ebi.ac.uk/QuickGO/GTerm?id=GO:0030838), [positive regulation of pseudopodium assembly](http://www.ebi.ac.uk/QuickGO/GTerm?id=GO:0031274), | | |
| **GOTERM_CC_DIRECT** | [cytoplasm](http://www.ebi.ac.uk/QuickGO/GTerm?id=GO:0005737), [cytosol](http://www.ebi.ac.uk/QuickGO/GTerm?id=GO:0005829), [cytoskeleton](http://www.ebi.ac.uk/QuickGO/GTerm?id=GO:0005856), [plasma membrane](http://www.ebi.ac.uk/QuickGO/GTerm?id=GO:0005886), [endomembrane system](http://www.ebi.ac.uk/QuickGO/GTerm?id=GO:0012505), [actin cytoskeleton](http://www.ebi.ac.uk/QuickGO/GTerm?id=GO:0015629), | | |
| **GOTERM_MF_DIRECT** | [protein binding](http://www.ebi.ac.uk/QuickGO/GTerm?id=GO:0005515), [cytoskeletal regulatory protein binding](http://www.ebi.ac.uk/QuickGO/GTerm?id=GO:0005519), | | |
| **INTERPRO** | [PAK-box/P21-Rho-binding](https://www.ebi.ac.uk/interpro/entry/InterPro/IPR000095), | | |
| **SMART** | [PBD](http://smart.embl.de/smart/do_annotation.pl?DOMAIN=SM00285), | | |
| **UP_KW_BIOLOGICAL_PROCESS** | [Cell shape](http://www.uniprot.org/keywords/?query=KW-0133), | | |
| **UP_KW_CELLULAR_COMPONENT** | [Cytoskeleton](http://www.uniprot.org/keywords/?query=KW-0206), [Membrane](http://www.uniprot.org/keywords/?query=KW-0472), [Cytoplasm](http://www.uniprot.org/keywords/?query=KW-0963), | | |
| **UP_KW_PTM** | [Phosphoprotein](http://www.uniprot.org/keywords/?query=KW-0597), | | |
| **UP_SEQ_FEATURE** | COMPBIAS:Basic and acidic residues, COMPBIAS:Polar residues, DOMAIN:CRIB, REGION:Disordered, | | |
| **CELF2-AS1** | [**CELF2 antisense RNA 1(CELF2-AS1)**](https://david.ncifcrf.gov/geneReportFull.jsp?rowids=414196) | [**Related Genes**](https://david.ncifcrf.gov/relatedGenes.jsp?id=414196) | [**Homo sapiens**](http://www.ncbi.nlm.nih.gov/Taxonomy/Browser/wwwtax.cgi?name=Homo%20sapiens) |
| **GOTERM_CC_DIRECT** | [extracellular region](http://www.ebi.ac.uk/QuickGO/GTerm?id=GO:0005576), | | |
| **UP_KW_CELLULAR_COMPONENT** | [Secreted](http://www.uniprot.org/keywords/?query=KW-0964), | | |
| **UP_KW_DOMAIN** | [Signal](http://www.uniprot.org/keywords/?query=KW-0732), | | |
| **CORO7-PAM16** | [**CORO7-PAM16 readthrough(CORO7-PAM16)**](https://david.ncifcrf.gov/geneReportFull.jsp?rowids=100529144) | [**Related Genes**](https://david.ncifcrf.gov/relatedGenes.jsp?id=100529144) | [**Homo sapiens**](http://www.ncbi.nlm.nih.gov/Taxonomy/Browser/wwwtax.cgi?name=Homo%20sapiens) |
| **GOTERM_CC_DIRECT** | [Golgi membrane](http://www.ebi.ac.uk/QuickGO/GTerm?id=GO:0000139), [cytosol](http://www.ebi.ac.uk/QuickGO/GTerm?id=GO:0005829), | | |
| **INTERPRO** | [WD40 repeat](https://www.ebi.ac.uk/interpro/entry/InterPro/IPR001680), [Domain of unknown function DUF1899](https://www.ebi.ac.uk/interpro/entry/InterPro/IPR015048), [Coronin](https://www.ebi.ac.uk/interpro/entry/InterPro/IPR015505), [WD40/YVTN repeat-like-containing domain](https://www.ebi.ac.uk/interpro/entry/InterPro/IPR015943), [WD40-repeat-containing domain](https://www.ebi.ac.uk/interpro/entry/InterPro/IPR017986), [WD40 repeat, conserved site](https://www.ebi.ac.uk/interpro/entry/InterPro/IPR019775), [Coronin 7](https://www.ebi.ac.uk/interpro/entry/InterPro/IPR027331), | | |
| **SMART** | [WD40](http://smart.embl.de/smart/do_annotation.pl?DOMAIN=SM00320), [SM01166](http://smart.embl.de/smart/do_annotation.pl?DOMAIN=SM01166), | | |
| **UP_KW_DOMAIN** | [Repeat](http://www.uniprot.org/keywords/?query=KW-0677), | | |
| **UP_KW_PTM** | [Phosphoprotein](http://www.uniprot.org/keywords/?query=KW-0597), [Isopeptide bond](http://www.uniprot.org/keywords/?query=KW-1017), | | |
| **UP_SEQ_FEATURE** | COMPBIAS:Polar residues, DOMAIN:DUF1899, REGION:Disordered, REPEAT:WD, | | |
| **CRK** | [**CRK proto-oncogene, adaptor protein(CRK)**](https://david.ncifcrf.gov/geneReportFull.jsp?rowids=1398) | [**Related Genes**](https://david.ncifcrf.gov/relatedGenes.jsp?id=1398) | [**Homo sapiens**](http://www.ncbi.nlm.nih.gov/Taxonomy/Browser/wwwtax.cgi?name=Homo%20sapiens) |
| **BIOCARTA** | [CXCR4 Signaling Pathway](https://david.ncifcrf.gov/biocarta.jsp?path=h_cxcr4Pathway$CXCR4%20Signaling%20Pathway&termId=30000078&source=biocarta), [Signaling of Hepatocyte Growth Factor Receptor](https://david.ncifcrf.gov/biocarta.jsp?path=h_metPathway$Signaling%20of%20Hepatocyte%20Growth%20Factor%20Receptor&termId=30000187&source=biocarta), | | |
| **GOTERM_BP_DIRECT** | [activation of MAPKK activity](http://www.ebi.ac.uk/QuickGO/GTerm?id=GO:0000186), [neuron migration](http://www.ebi.ac.uk/QuickGO/GTerm?id=GO:0001764), [response to yeast](http://www.ebi.ac.uk/QuickGO/GTerm?id=GO:0001878), [regulation of transcription from RNA polymerase II promoter](http://www.ebi.ac.uk/QuickGO/GTerm?id=GO:0006357), [lipid metabolic process](http://www.ebi.ac.uk/QuickGO/GTerm?id=GO:0006629), [signal transduction](http://www.ebi.ac.uk/QuickGO/GTerm?id=GO:0007165), [regulation of cell shape](http://www.ebi.ac.uk/QuickGO/GTerm?id=GO:0008360), [regulation of signal transduction](http://www.ebi.ac.uk/QuickGO/GTerm?id=GO:0009966), [positive regulation of smooth muscle cell migration](http://www.ebi.ac.uk/QuickGO/GTerm?id=GO:0014911), [dendrite development](http://www.ebi.ac.uk/QuickGO/GTerm?id=GO:0016358), [cell migration](http://www.ebi.ac.uk/QuickGO/GTerm?id=GO:0016477), [cytokine-mediated signaling pathway](http://www.ebi.ac.uk/QuickGO/GTerm?id=GO:0019221), [hippocampus development](http://www.ebi.ac.uk/QuickGO/GTerm?id=GO:0021766), [cerebral cortex development](http://www.ebi.ac.uk/QuickGO/GTerm?id=GO:0021987), [establishment of cell polarity](http://www.ebi.ac.uk/QuickGO/GTerm?id=GO:0030010), [actin cytoskeleton organization](http://www.ebi.ac.uk/QuickGO/GTerm?id=GO:0030036), [positive regulation of cell growth](http://www.ebi.ac.uk/QuickGO/GTerm?id=GO:0030307), [regulation of actin cytoskeleton organization](http://www.ebi.ac.uk/QuickGO/GTerm?id=GO:0032956), [regulation of cell adhesion mediated by integrin](http://www.ebi.ac.uk/QuickGO/GTerm?id=GO:0033628), [regulation of Rac protein signal transduction](http://www.ebi.ac.uk/QuickGO/GTerm?id=GO:0035020), [helper T cell diapedesis](http://www.ebi.ac.uk/QuickGO/GTerm?id=GO:0035685), [response to hepatocyte growth factor](http://www.ebi.ac.uk/QuickGO/GTerm?id=GO:0035728), [reelin-mediated signaling pathway](http://www.ebi.ac.uk/QuickGO/GTerm?id=GO:0038026), [Fc-gamma receptor signaling pathway involved in phagocytosis](http://www.ebi.ac.uk/QuickGO/GTerm?id=GO:0038096), [response to hydrogen peroxide](http://www.ebi.ac.uk/QuickGO/GTerm?id=GO:0042542), [regulation of GTPase activity](http://www.ebi.ac.uk/QuickGO/GTerm?id=GO:0043087), [regulation of protein binding](http://www.ebi.ac.uk/QuickGO/GTerm?id=GO:0043393), [negative regulation of natural killer cell mediated cytotoxicity](http://www.ebi.ac.uk/QuickGO/GTerm?id=GO:0045953), [vascular endothelial growth factor receptor signaling pathway](http://www.ebi.ac.uk/QuickGO/GTerm?id=GO:0048010), [ephrin receptor signaling pathway](http://www.ebi.ac.uk/QuickGO/GTerm?id=GO:0048013), [regulation of dendrite development](http://www.ebi.ac.uk/QuickGO/GTerm?id=GO:0050773), [cell chemotaxis](http://www.ebi.ac.uk/QuickGO/GTerm?id=GO:0060326), [negative regulation of wound healing](http://www.ebi.ac.uk/QuickGO/GTerm?id=GO:0061045), [response to cholecystokinin](http://www.ebi.ac.uk/QuickGO/GTerm?id=GO:0061847), [cellular response to transforming growth factor beta stimulus](http://www.ebi.ac.uk/QuickGO/GTerm?id=GO:0071560), [cellular response to nitric oxide](http://www.ebi.ac.uk/QuickGO/GTerm?id=GO:0071732), [activation of GTPase activity](http://www.ebi.ac.uk/QuickGO/GTerm?id=GO:0090630), [cerebellar neuron development](http://www.ebi.ac.uk/QuickGO/GTerm?id=GO:0098749), [positive regulation of substrate adhesion-dependent cell spreading](http://www.ebi.ac.uk/QuickGO/GTerm?id=GO:1900026), [regulation of intracellular signal transduction](http://www.ebi.ac.uk/QuickGO/GTerm?id=GO:1902531), [cellular response to nerve growth factor stimulus](http://www.ebi.ac.uk/QuickGO/GTerm?id=GO:1990090), [cellular response to insulin-like growth factor stimulus](http://www.ebi.ac.uk/QuickGO/GTerm?id=GO:1990314), [cellular response to endothelin](http://www.ebi.ac.uk/QuickGO/GTerm?id=GO:1990859), [negative regulation of cell motility](http://www.ebi.ac.uk/QuickGO/GTerm?id=GO:2000146), [regulation of T cell migration](http://www.ebi.ac.uk/QuickGO/GTerm?id=GO:2000404), | | |
| **GOTERM_CC_DIRECT** | [nucleus](http://www.ebi.ac.uk/QuickGO/GTerm?id=GO:0005634), [cytoplasm](http://www.ebi.ac.uk/QuickGO/GTerm?id=GO:0005737), [cytosol](http://www.ebi.ac.uk/QuickGO/GTerm?id=GO:0005829), [plasma membrane](http://www.ebi.ac.uk/QuickGO/GTerm?id=GO:0005886), [actin cytoskeleton](http://www.ebi.ac.uk/QuickGO/GTerm?id=GO:0015629), [membrane](http://www.ebi.ac.uk/QuickGO/GTerm?id=GO:0016020), [macromolecular complex](http://www.ebi.ac.uk/QuickGO/GTerm?id=GO:0032991), [membrane raft](http://www.ebi.ac.uk/QuickGO/GTerm?id=GO:0045121), [extracellular exosome](http://www.ebi.ac.uk/QuickGO/GTerm?id=GO:0070062), | | |
| **GOTERM_MF_DIRECT** | [phosphotyrosine binding](http://www.ebi.ac.uk/QuickGO/GTerm?id=GO:0001784), [insulin-like growth factor receptor binding](http://www.ebi.ac.uk/QuickGO/GTerm?id=GO:0005159), [protein binding](http://www.ebi.ac.uk/QuickGO/GTerm?id=GO:0005515), [cytoskeletal protein binding](http://www.ebi.ac.uk/QuickGO/GTerm?id=GO:0008092), [SH3 domain binding](http://www.ebi.ac.uk/QuickGO/GTerm?id=GO:0017124), [kinase binding](http://www.ebi.ac.uk/QuickGO/GTerm?id=GO:0019900), [protein domain specific binding](http://www.ebi.ac.uk/QuickGO/GTerm?id=GO:0019904), [receptor signaling complex scaffold activity](http://www.ebi.ac.uk/QuickGO/GTerm?id=GO:0030159), [receptor tyrosine kinase binding](http://www.ebi.ac.uk/QuickGO/GTerm?id=GO:0030971), [ubiquitin protein ligase binding](http://www.ebi.ac.uk/QuickGO/GTerm?id=GO:0031625), [signaling adaptor activity](http://www.ebi.ac.uk/QuickGO/GTerm?id=GO:0035591), [SH2 domain binding](http://www.ebi.ac.uk/QuickGO/GTerm?id=GO:0042169), [protein self-association](http://www.ebi.ac.uk/QuickGO/GTerm?id=GO:0043621), [protein phosphorylated amino acid binding](http://www.ebi.ac.uk/QuickGO/GTerm?id=GO:0045309), [ephrin receptor binding](http://www.ebi.ac.uk/QuickGO/GTerm?id=GO:0046875), [scaffold protein binding](http://www.ebi.ac.uk/QuickGO/GTerm?id=GO:0097110), [protein tyrosine kinase binding](http://www.ebi.ac.uk/QuickGO/GTerm?id=GO:1990782), | | |
| **INTERPRO** | [SH2 domain](https://www.ebi.ac.uk/interpro/entry/InterPro/IPR000980), [Src homology-3 domain](https://www.ebi.ac.uk/interpro/entry/InterPro/IPR001452), | | |
| **KEGG_PATHWAY** | [MAPK signaling pathway](https://david.ncifcrf.gov/kegg.jsp?path=hsa04010$MAPK%20signaling%20pathway&termId=520047941&source=kegg), [ErbB signaling pathway](https://david.ncifcrf.gov/kegg.jsp?path=hsa04012$ErbB%20signaling%20pathway&termId=520047942&source=kegg), [Rap1 signaling pathway](https://david.ncifcrf.gov/kegg.jsp?path=hsa04015$Rap1%20signaling%20pathway&termId=520047944&source=kegg), [Chemokine signaling pathway](https://david.ncifcrf.gov/kegg.jsp?path=hsa04062$Chemokine%20signaling%20pathway&termId=520047950&source=kegg), [Focal adhesion](https://david.ncifcrf.gov/kegg.jsp?path=hsa04510$Focal%20adhesion&termId=520047995&source=kegg), [Fc gamma R-mediated phagocytosis](https://david.ncifcrf.gov/kegg.jsp?path=hsa04666$Fc%20gamma%20R-mediated%20phagocytosis&termId=520048021&source=kegg), [Neurotrophin signaling pathway](https://david.ncifcrf.gov/kegg.jsp?path=hsa04722$Neurotrophin%20signaling%20pathway&termId=520048030&source=kegg), [Regulation of actin cytoskeleton](https://david.ncifcrf.gov/kegg.jsp?path=hsa04810$Regulation%20of%20actin%20cytoskeleton&termId=520048042&source=kegg), [Insulin signaling pathway](https://david.ncifcrf.gov/kegg.jsp?path=hsa04910$Insulin%20signaling%20pathway&termId=520048043&source=kegg), [Growth hormone synthesis, secretion and action](https://david.ncifcrf.gov/kegg.jsp?path=hsa04935$Growth%20hormone%20synthesis,%20secretion%20and%20action&termId=520048068&source=kegg), [Bacterial invasion of epithelial cells](https://david.ncifcrf.gov/kegg.jsp?path=hsa05100$Bacterial%20invasion%20of%20epithelial%20cells&termId=520048099&source=kegg), [Shigellosis](https://david.ncifcrf.gov/kegg.jsp?path=hsa05131$Shigellosis&termId=520048103&source=kegg), [Yersinia infection](https://david.ncifcrf.gov/kegg.jsp?path=hsa05135$Yersinia%20infection&termId=520048107&source=kegg), [Human cytomegalovirus infection](https://david.ncifcrf.gov/kegg.jsp?path=hsa05163$Human%20cytomegalovirus%20infection&termId=520048119&source=kegg), [Human immunodeficiency virus 1 infection](https://david.ncifcrf.gov/kegg.jsp?path=hsa05170$Human%20immunodeficiency%20virus%201%20infection&termId=520048126&source=kegg), [Pathways in cancer](https://david.ncifcrf.gov/kegg.jsp?path=hsa05200$Pathways%20in%20cancer&termId=520048128&source=kegg), [MicroRNAs in cancer](https://david.ncifcrf.gov/kegg.jsp?path=hsa05206$MicroRNAs%20in%20cancer&termId=520048133&source=kegg), [Renal cell carcinoma](https://david.ncifcrf.gov/kegg.jsp?path=hsa05211$Renal%20cell%20carcinoma&termId=520048137&source=kegg), [Chronic myeloid leukemia](https://david.ncifcrf.gov/kegg.jsp?path=hsa05220$Chronic%20myeloid%20leukemia&termId=520048146&source=kegg), | | |
| **SMART** | [SH2](http://smart.embl.de/smart/do_annotation.pl?DOMAIN=SM00252), [SH3](http://smart.embl.de/smart/do_annotation.pl?DOMAIN=SM00326), | | |
| **UP_KW_CELLULAR_COMPONENT** | [Membrane](http://www.uniprot.org/keywords/?query=KW-0472), [Cytoplasm](http://www.uniprot.org/keywords/?query=KW-0963), [Cell membrane](http://www.uniprot.org/keywords/?query=KW-1003), | | |
| **UP_KW_DISEASE** | [Proto-oncogene](http://www.uniprot.org/keywords/?query=KW-0656), | | |
| **UP_KW_DOMAIN** | [Repeat](http://www.uniprot.org/keywords/?query=KW-0677), [SH2 domain](http://www.uniprot.org/keywords/?query=KW-0727), [SH3 domain](http://www.uniprot.org/keywords/?query=KW-0728), | | |
| **UP_KW_PTM** | [Acetylation](http://www.uniprot.org/keywords/?query=KW-0007), [Phosphoprotein](http://www.uniprot.org/keywords/?query=KW-0597), | | |
| **UP_SEQ_FEATURE** | COMPBIAS:Pro residues, DOMAIN:SH2, DOMAIN:SH3, DOMAIN:SH3 1, DOMAIN:SH3 2, MUTAGEN:D->K: Abolishes interaction with DOCK1., MUTAGEN:W->K: Abolishes interaction with PEAK3., MUTAGEN:W->K: No effect on interaction with PEAK3., MUTAGEN:W->L: Abolishes interaction with DOCK5. Abolishes RAP1 activation., REGION:Disordered, SITE:Proline switch, | | |
| **DAZAP1** | [**DAZ associated protein 1(DAZAP1)**](https://david.ncifcrf.gov/geneReportFull.jsp?rowids=26528) | [**Related Genes**](https://david.ncifcrf.gov/relatedGenes.jsp?id=26528) | [**Homo sapiens**](http://www.ncbi.nlm.nih.gov/Taxonomy/Browser/wwwtax.cgi?name=Homo%20sapiens) |
| **GOTERM_BP_DIRECT** | [maternal placenta development](http://www.ebi.ac.uk/QuickGO/GTerm?id=GO:0001893), [spermatogenesis](http://www.ebi.ac.uk/QuickGO/GTerm?id=GO:0007283), [cell proliferation](http://www.ebi.ac.uk/QuickGO/GTerm?id=GO:0008283), [cell differentiation](http://www.ebi.ac.uk/QuickGO/GTerm?id=GO:0030154), [positive regulation of mRNA splicing, via spliceosome](http://www.ebi.ac.uk/QuickGO/GTerm?id=GO:0048026), | | |
| **GOTERM_CC_DIRECT** | [nucleoplasm](http://www.ebi.ac.uk/QuickGO/GTerm?id=GO:0005654), [cytosol](http://www.ebi.ac.uk/QuickGO/GTerm?id=GO:0005829), [macromolecular complex](http://www.ebi.ac.uk/QuickGO/GTerm?id=GO:0032991), [ribonucleoprotein complex](http://www.ebi.ac.uk/QuickGO/GTerm?id=GO:1990904), | | |
| **GOTERM_MF_DIRECT** | [nucleic acid binding](http://www.ebi.ac.uk/QuickGO/GTerm?id=GO:0003676), [RNA binding](http://www.ebi.ac.uk/QuickGO/GTerm?id=GO:0003723), [mRNA 3'-UTR binding](http://www.ebi.ac.uk/QuickGO/GTerm?id=GO:0003730), [protein binding](http://www.ebi.ac.uk/QuickGO/GTerm?id=GO:0005515), [poly(U) RNA binding](http://www.ebi.ac.uk/QuickGO/GTerm?id=GO:0008266), [poly(G) binding](http://www.ebi.ac.uk/QuickGO/GTerm?id=GO:0034046), [RNA stem-loop binding](http://www.ebi.ac.uk/QuickGO/GTerm?id=GO:0035613), | | |
| **INTERPRO** | [RNA recognition motif domain](https://www.ebi.ac.uk/interpro/entry/InterPro/IPR000504), [Nucleotide-binding, alpha-beta plait](https://www.ebi.ac.uk/interpro/entry/InterPro/IPR012677), | | |
| **KEGG_PATHWAY** | [mRNA surveillance pathway](https://david.ncifcrf.gov/kegg.jsp?path=hsa03015$mRNA%20surveillance%20pathway&termId=520047926&source=kegg), | | |
| **SMART** | [RRM](http://smart.embl.de/smart/do_annotation.pl?DOMAIN=SM00360), | | |
| **UP_KW_BIOLOGICAL_PROCESS** | [Differentiation](http://www.uniprot.org/keywords/?query=KW-0221), [Spermatogenesis](http://www.uniprot.org/keywords/?query=KW-0744), | | |
| **UP_KW_CELLULAR_COMPONENT** | [Nucleus](http://www.uniprot.org/keywords/?query=KW-0539), [Cytoplasm](http://www.uniprot.org/keywords/?query=KW-0963), | | |
| **UP_KW_DOMAIN** | [Repeat](http://www.uniprot.org/keywords/?query=KW-0677), | | |
| **UP_KW_MOLECULAR_FUNCTION** | [Developmental protein](http://www.uniprot.org/keywords/?query=KW-0217), [RNA-binding](http://www.uniprot.org/keywords/?query=KW-0694), [Developmental protein](http://www.uniprot.org/keywords/?query=KW-9996), | | |
| **UP_KW_PTM** | [Acetylation](http://www.uniprot.org/keywords/?query=KW-0007), [Methylation](http://www.uniprot.org/keywords/?query=KW-0488), | | |
| **UP_SEQ_FEATURE** | COMPBIAS:Basic and acidic residues, COMPBIAS:Polar residues, COMPBIAS:Pro residues, DOMAIN:RRM, DOMAIN:RRM 1, DOMAIN:RRM 2, REGION:Disordered, | | |
| **DLGAP2** | [**DLG associated protein 2(DLGAP2)**](https://david.ncifcrf.gov/geneReportFull.jsp?rowids=9228) | [**Related Genes**](https://david.ncifcrf.gov/relatedGenes.jsp?id=9228) | [**Homo sapiens**](http://www.ncbi.nlm.nih.gov/Taxonomy/Browser/wwwtax.cgi?name=Homo%20sapiens) |
| **GOTERM_BP_DIRECT** | [neuron-neuron synaptic transmission](http://www.ebi.ac.uk/QuickGO/GTerm?id=GO:0007270), [regulation of postsynaptic neurotransmitter receptor activity](http://www.ebi.ac.uk/QuickGO/GTerm?id=GO:0098962), | | |
| **GOTERM_CC_DIRECT** | [neurofilament](http://www.ebi.ac.uk/QuickGO/GTerm?id=GO:0005883), [plasma membrane](http://www.ebi.ac.uk/QuickGO/GTerm?id=GO:0005886), [postsynaptic density](http://www.ebi.ac.uk/QuickGO/GTerm?id=GO:0014069), [synapse](http://www.ebi.ac.uk/QuickGO/GTerm?id=GO:0045202), [glutamatergic synapse](http://www.ebi.ac.uk/QuickGO/GTerm?id=GO:0098978), [postsynaptic specialization](http://www.ebi.ac.uk/QuickGO/GTerm?id=GO:0099572), | | |
| **GOTERM_MF_DIRECT** | [protein binding](http://www.ebi.ac.uk/QuickGO/GTerm?id=GO:0005515), [binding, bridging](http://www.ebi.ac.uk/QuickGO/GTerm?id=GO:0060090), | | |
| **INTERPRO** | [Guanylate-kinase-associated protein](https://www.ebi.ac.uk/interpro/entry/InterPro/IPR005026), | | |
| **UP_KW_CELLULAR_COMPONENT** | [Membrane](http://www.uniprot.org/keywords/?query=KW-0472), [Synapse](http://www.uniprot.org/keywords/?query=KW-0770), [Cell junction](http://www.uniprot.org/keywords/?query=KW-0965), [Cell membrane](http://www.uniprot.org/keywords/?query=KW-1003), | | |
| **UP_KW_PTM** | [Phosphoprotein](http://www.uniprot.org/keywords/?query=KW-0597), | | |
| **UP_SEQ_FEATURE** | COMPBIAS:Basic and acidic residues, COMPBIAS:Polar residues, REGION:Disordered, | | |
| **DNMT3A** | [**DNA methyltransferase 3 alpha(DNMT3A)**](https://david.ncifcrf.gov/geneReportFull.jsp?rowids=1788) | [**Related Genes**](https://david.ncifcrf.gov/relatedGenes.jsp?id=1788) | [**Homo sapiens**](http://www.ncbi.nlm.nih.gov/Taxonomy/Browser/wwwtax.cgi?name=Homo%20sapiens) |
| **GOTERM_BP_DIRECT** | [negative regulation of transcription from RNA polymerase II promoter](http://www.ebi.ac.uk/QuickGO/GTerm?id=GO:0000122), [mitotic cell cycle](http://www.ebi.ac.uk/QuickGO/GTerm?id=GO:0000278), [DNA methylation](http://www.ebi.ac.uk/QuickGO/GTerm?id=GO:0006306), [methylation-dependent chromatin silencing](http://www.ebi.ac.uk/QuickGO/GTerm?id=GO:0006346), [regulation of gene expression by genetic imprinting](http://www.ebi.ac.uk/QuickGO/GTerm?id=GO:0006349), [protein methylation](http://www.ebi.ac.uk/QuickGO/GTerm?id=GO:0006479), [spermatogenesis](http://www.ebi.ac.uk/QuickGO/GTerm?id=GO:0007283), [aging](http://www.ebi.ac.uk/QuickGO/GTerm?id=GO:0007568), [response to xenobiotic stimulus](http://www.ebi.ac.uk/QuickGO/GTerm?id=GO:0009410), [response to toxic substance](http://www.ebi.ac.uk/QuickGO/GTerm?id=GO:0009636), [response to ionizing radiation](http://www.ebi.ac.uk/QuickGO/GTerm?id=GO:0010212), [response to lead ion](http://www.ebi.ac.uk/QuickGO/GTerm?id=GO:0010288), [regulation of gene expression](http://www.ebi.ac.uk/QuickGO/GTerm?id=GO:0010468), [positive regulation of cell death](http://www.ebi.ac.uk/QuickGO/GTerm?id=GO:0010942), [neuron differentiation](http://www.ebi.ac.uk/QuickGO/GTerm?id=GO:0030182), [response to estradiol](http://www.ebi.ac.uk/QuickGO/GTerm?id=GO:0032355), [DNA methylation on cytosine](http://www.ebi.ac.uk/QuickGO/GTerm?id=GO:0032776), [response to vitamin A](http://www.ebi.ac.uk/QuickGO/GTerm?id=GO:0033189), [response to cocaine](http://www.ebi.ac.uk/QuickGO/GTerm?id=GO:0042220), [response to drug](http://www.ebi.ac.uk/QuickGO/GTerm?id=GO:0042493), [DNA methylation involved in embryo development](http://www.ebi.ac.uk/QuickGO/GTerm?id=GO:0043045), [DNA methylation involved in gamete generation](http://www.ebi.ac.uk/QuickGO/GTerm?id=GO:0043046), [negative regulation of gene expression, epigenetic](http://www.ebi.ac.uk/QuickGO/GTerm?id=GO:0045814), [negative regulation of transcription, DNA-templated](http://www.ebi.ac.uk/QuickGO/GTerm?id=GO:0045892), [cellular response to amino acid stimulus](http://www.ebi.ac.uk/QuickGO/GTerm?id=GO:0071230), [cellular response to ethanol](http://www.ebi.ac.uk/QuickGO/GTerm?id=GO:0071361), [cellular response to hypoxia](http://www.ebi.ac.uk/QuickGO/GTerm?id=GO:0071456), [C-5 methylation of cytosine](http://www.ebi.ac.uk/QuickGO/GTerm?id=GO:0090116), [hepatocyte apoptotic process](http://www.ebi.ac.uk/QuickGO/GTerm?id=GO:0097284), [cellular response to bisphenol A](http://www.ebi.ac.uk/QuickGO/GTerm?id=GO:1903926), | | |
| **GOTERM_CC_DIRECT** | [chromosome, centromeric region](http://www.ebi.ac.uk/QuickGO/GTerm?id=GO:0000775), [euchromatin](http://www.ebi.ac.uk/QuickGO/GTerm?id=GO:0000791), [heterochromatin](http://www.ebi.ac.uk/QuickGO/GTerm?id=GO:0000792), [XY body](http://www.ebi.ac.uk/QuickGO/GTerm?id=GO:0001741), [nucleus](http://www.ebi.ac.uk/QuickGO/GTerm?id=GO:0005634), [nucleoplasm](http://www.ebi.ac.uk/QuickGO/GTerm?id=GO:0005654), [cytoplasm](http://www.ebi.ac.uk/QuickGO/GTerm?id=GO:0005737), [nuclear matrix](http://www.ebi.ac.uk/QuickGO/GTerm?id=GO:0016363), [catalytic complex](http://www.ebi.ac.uk/QuickGO/GTerm?id=GO:1902494), | | |
| **GOTERM_MF_DIRECT** | [RNA polymerase II core promoter proximal region sequence-specific DNA binding](http://www.ebi.ac.uk/QuickGO/GTerm?id=GO:0000978), [DNA binding](http://www.ebi.ac.uk/QuickGO/GTerm?id=GO:0003677), [chromatin binding](http://www.ebi.ac.uk/QuickGO/GTerm?id=GO:0003682), [transcription corepressor activity](http://www.ebi.ac.uk/QuickGO/GTerm?id=GO:0003714), [DNA (cytosine-5-)-methyltransferase activity](http://www.ebi.ac.uk/QuickGO/GTerm?id=GO:0003886), [protein binding](http://www.ebi.ac.uk/QuickGO/GTerm?id=GO:0005515), [transcription factor binding](http://www.ebi.ac.uk/QuickGO/GTerm?id=GO:0008134), [DNA-methyltransferase activity](http://www.ebi.ac.uk/QuickGO/GTerm?id=GO:0009008), [identical protein binding](http://www.ebi.ac.uk/QuickGO/GTerm?id=GO:0042802), [metal ion binding](http://www.ebi.ac.uk/QuickGO/GTerm?id=GO:0046872), [RNA polymerase II sequence-specific DNA binding transcription factor binding](http://www.ebi.ac.uk/QuickGO/GTerm?id=GO:0061629), | | |
| **INTERPRO** | [PWWP](https://www.ebi.ac.uk/interpro/entry/InterPro/IPR000313), [C-5 cytosine methyltransferase](https://www.ebi.ac.uk/interpro/entry/InterPro/IPR001525), [DNA methylase, C-5 cytosine-specific, active site](https://www.ebi.ac.uk/interpro/entry/InterPro/IPR018117), [ADD domain](https://www.ebi.ac.uk/interpro/entry/InterPro/IPR025766), | | |
| **KEGG_PATHWAY** | [Cysteine and methionine metabolism](https://david.ncifcrf.gov/kegg.jsp?path=hsa00270$Cysteine%20and%20methionine%20metabolism&termId=520047847&source=kegg), [Metabolic pathways](https://david.ncifcrf.gov/kegg.jsp?path=hsa01100$Metabolic%20pathways&termId=520047911&source=kegg), [MicroRNAs in cancer](https://david.ncifcrf.gov/kegg.jsp?path=hsa05206$MicroRNAs%20in%20cancer&termId=520048133&source=kegg), | | |
| **OMIM_DISEASE** | [Acute myeloid leukemia, somatic](http://omim.org/entry/601626), [Tatton-Brown-Rahman syndrome](http://omim.org/entry/615879), [Heyn-Sproul-Jackson syndrome](http://omim.org/entry/618724), | | |
| **SMART** | [PWWP](http://smart.embl.de/smart/do_annotation.pl?DOMAIN=SM00293), | | |
| **UP_KW_CELLULAR_COMPONENT** | [Chromosome](http://www.uniprot.org/keywords/?query=KW-0158), [Nucleus](http://www.uniprot.org/keywords/?query=KW-0539), [Cytoplasm](http://www.uniprot.org/keywords/?query=KW-0963), | | |
| **UP_KW_DISEASE** | [Disease variant](http://www.uniprot.org/keywords/?query=KW-0225), [Dwarfism](http://www.uniprot.org/keywords/?query=KW-0242), [Mental retardation](http://www.uniprot.org/keywords/?query=KW-0991), | | |
| **UP_KW_DOMAIN** | [Zinc-finger](http://www.uniprot.org/keywords/?query=KW-0863), | | |
| **UP_KW_LIGAND** | [Metal-binding](http://www.uniprot.org/keywords/?query=KW-0479), [Zinc](http://www.uniprot.org/keywords/?query=KW-0862), [S-adenosyl-L-methionine](http://www.uniprot.org/keywords/?query=KW-0949), | | |
| **UP_KW_MOLECULAR_FUNCTION** | [Chromatin regulator](http://www.uniprot.org/keywords/?query=KW-0156), [DNA-binding](http://www.uniprot.org/keywords/?query=KW-0238), [Methyltransferase](http://www.uniprot.org/keywords/?query=KW-0489), [Repressor](http://www.uniprot.org/keywords/?query=KW-0678), [Transferase](http://www.uniprot.org/keywords/?query=KW-0808), | | |
| **UP_KW_PTM** | [Methylation](http://www.uniprot.org/keywords/?query=KW-0488), [Phosphoprotein](http://www.uniprot.org/keywords/?query=KW-0597), [Ubl conjugation](http://www.uniprot.org/keywords/?query=KW-0832), [Isopeptide bond](http://www.uniprot.org/keywords/?query=KW-1017), | | |
| **UP_SEQ_FEATURE** | BINDING:S-adenosyl-L-methionine, COMPBIAS:Basic and acidic residues, COMPBIAS:Pro residues, CROSSLNK:Glycyl lysine isopeptide (Lys-Gly) (interchain with G-Cter in SUMO2), DOMAIN:ADD, DOMAIN:PHD-type, DOMAIN:PWWP, DOMAIN:SAM-dependent MTase C5-type, MUTAGEN:F->A: Loss of activity due to the incapacity to bind the regulatory subunit DNMT3L., REGION:Disordered, REGION:Interaction with DNMT1 and DNMT3B, REGION:Interaction with the PRC2/EED-EZH2 complex, REGION:S-adenosyl-L-methionine binding, ZN_FING:GATA-type; atypical, ZN_FING:PHD-type; atypical, | | |
| **POLA2** | [**DNA polymerase alpha 2, accessory subunit(POLA2)**](https://david.ncifcrf.gov/geneReportFull.jsp?rowids=23649) | [**Related Genes**](https://david.ncifcrf.gov/relatedGenes.jsp?id=23649) | [**Homo sapiens**](http://www.ncbi.nlm.nih.gov/Taxonomy/Browser/wwwtax.cgi?name=Homo%20sapiens) |
| **GOTERM_BP_DIRECT** | [DNA replication](http://www.ebi.ac.uk/QuickGO/GTerm?id=GO:0006260), [DNA replication, synthesis of RNA primer](http://www.ebi.ac.uk/QuickGO/GTerm?id=GO:0006269), [DNA replication initiation](http://www.ebi.ac.uk/QuickGO/GTerm?id=GO:0006270), [telomere maintenance via semi-conservative replication](http://www.ebi.ac.uk/QuickGO/GTerm?id=GO:0032201), | | |
| **GOTERM_CC_DIRECT** | [nucleus](http://www.ebi.ac.uk/QuickGO/GTerm?id=GO:0005634), [nucleoplasm](http://www.ebi.ac.uk/QuickGO/GTerm?id=GO:0005654), [alpha DNA polymerase:primase complex](http://www.ebi.ac.uk/QuickGO/GTerm?id=GO:0005658), [cytosol](http://www.ebi.ac.uk/QuickGO/GTerm?id=GO:0005829), | | |
| **GOTERM_MF_DIRECT** | [DNA binding](http://www.ebi.ac.uk/QuickGO/GTerm?id=GO:0003677), [protein binding](http://www.ebi.ac.uk/QuickGO/GTerm?id=GO:0005515), | | |
| **INTERPRO** | [DNA polymerase alpha/epsilon, subunit B](https://www.ebi.ac.uk/interpro/entry/InterPro/IPR007185), [DNA polymerase alpha, subunit B N-terminal](https://www.ebi.ac.uk/interpro/entry/InterPro/IPR013627), [DNA polymerase alpha, subunit B](https://www.ebi.ac.uk/interpro/entry/InterPro/IPR016722), | | |
| **KEGG_PATHWAY** | [DNA replication](https://david.ncifcrf.gov/kegg.jsp?path=hsa03030$DNA%20replication&termId=520047930&source=kegg), | | |
| **PIR_SUPERFAMILY** | [DNA polymerase alpha, subunit B](http://pir.georgetown.edu/cgi-bin/ipcSF?id=PIRSF018300), | | |
| **UP_KW_BIOLOGICAL_PROCESS** | [DNA replication](http://www.uniprot.org/keywords/?query=KW-0235), | | |
| **UP_KW_CELLULAR_COMPONENT** | [Nucleus](http://www.uniprot.org/keywords/?query=KW-0539), | | |
| **UP_KW_PTM** | [Phosphoprotein](http://www.uniprot.org/keywords/?query=KW-0597), | | |
| **UP_SEQ_FEATURE** | DOMAIN:DNA_pol_E_B, DOMAIN:Pol_alpha_B_N, REGION:Disordered, | | |
| **TOP1MT** | [**DNA topoisomerase I mitochondrial(TOP1MT)**](https://david.ncifcrf.gov/geneReportFull.jsp?rowids=116447) | [**Related Genes**](https://david.ncifcrf.gov/relatedGenes.jsp?id=116447) | [**Homo sapiens**](http://www.ncbi.nlm.nih.gov/Taxonomy/Browser/wwwtax.cgi?name=Homo%20sapiens) |
| **COG_ONTOLOGY** | [DNA replication, recombination, and repair](http://www.ncbi.nlm.nih.gov/COG/new/), | | |
| **GOTERM_BP_DIRECT** | [DNA replication](http://www.ebi.ac.uk/QuickGO/GTerm?id=GO:0006260), [DNA topological change](http://www.ebi.ac.uk/QuickGO/GTerm?id=GO:0006265), | | |
| **GOTERM_CC_DIRECT** | [nucleoplasm](http://www.ebi.ac.uk/QuickGO/GTerm?id=GO:0005654), [chromosome](http://www.ebi.ac.uk/QuickGO/GTerm?id=GO:0005694), [mitochondrion](http://www.ebi.ac.uk/QuickGO/GTerm?id=GO:0005739), [mitochondrial nucleoid](http://www.ebi.ac.uk/QuickGO/GTerm?id=GO:0042645), | | |
| **GOTERM_MF_DIRECT** | [DNA binding](http://www.ebi.ac.uk/QuickGO/GTerm?id=GO:0003677), [DNA topoisomerase type I activity](http://www.ebi.ac.uk/QuickGO/GTerm?id=GO:0003917), | | |
| **INTERPRO** | [DNA topoisomerase I](https://www.ebi.ac.uk/interpro/entry/InterPro/IPR001631), [DNA topoisomerase I, DNA binding, eukaryotic-type](https://www.ebi.ac.uk/interpro/entry/InterPro/IPR008336), [DNA breaking-rejoining enzyme, catalytic core](https://www.ebi.ac.uk/interpro/entry/InterPro/IPR011010), [DNA topoisomerase I, DNA binding, mixed alpha/beta motif, eukaryotic-type](https://www.ebi.ac.uk/interpro/entry/InterPro/IPR013030), [DNA topoisomerase I, domain 1](https://www.ebi.ac.uk/interpro/entry/InterPro/IPR013034), [DNA topoisomerase I, eukaryotic-type](https://www.ebi.ac.uk/interpro/entry/InterPro/IPR013499), [DNA topoisomerase I, catalytic core, eukaryotic-type](https://www.ebi.ac.uk/interpro/entry/InterPro/IPR013500), [DNA topoisomerase I, catalytic core, alpha-helical subdomain, eukaryotic-type](https://www.ebi.ac.uk/interpro/entry/InterPro/IPR014711), [DNA topoisomerase I, catalytic core, alpha/beta subdomain](https://www.ebi.ac.uk/interpro/entry/InterPro/IPR014727), [DNA topoisomerase I, active site](https://www.ebi.ac.uk/interpro/entry/InterPro/IPR018521), [Topoisomerase I C-terminal domain](https://www.ebi.ac.uk/interpro/entry/InterPro/IPR025834), | | |
| **SMART** | [TOPEUc](http://smart.embl.de/smart/do_annotation.pl?DOMAIN=SM00435), | | |
| **UP_KW_CELLULAR_COMPONENT** | [Mitochondrion](http://www.uniprot.org/keywords/?query=KW-0496), | | |
| **UP_KW_DOMAIN** | [Coiled coil](http://www.uniprot.org/keywords/?query=KW-0175), [Transit peptide](http://www.uniprot.org/keywords/?query=KW-0809), | | |
| **UP_KW_MOLECULAR_FUNCTION** | [DNA-binding](http://www.uniprot.org/keywords/?query=KW-0238), [Isomerase](http://www.uniprot.org/keywords/?query=KW-0413), [Topoisomerase](http://www.uniprot.org/keywords/?query=KW-0799), | | |
| **UP_SEQ_FEATURE** | ACT_SITE:O-(3'-phospho-DNA)-tyrosine intermediate, DOMAIN:TOPEUc, DOMAIN:Topo_C_assoc, DOMAIN:Topoisom_I_N, REGION:Disordered, REGION:Interaction with DNA, SITE:Interaction with DNA, TRANSIT:Mitochondrion, | | |
| **EFCAB2** | [**EF-hand calcium binding domain 2(EFCAB2)**](https://david.ncifcrf.gov/geneReportFull.jsp?rowids=84288) | [**Related Genes**](https://david.ncifcrf.gov/relatedGenes.jsp?id=84288) | [**Homo sapiens**](http://www.ncbi.nlm.nih.gov/Taxonomy/Browser/wwwtax.cgi?name=Homo%20sapiens) |
| **GOTERM_CC_DIRECT** | [cytoplasm](http://www.ebi.ac.uk/QuickGO/GTerm?id=GO:0005737), [cytoskeleton](http://www.ebi.ac.uk/QuickGO/GTerm?id=GO:0005856), [motile cilium](http://www.ebi.ac.uk/QuickGO/GTerm?id=GO:0031514), [sperm principal piece](http://www.ebi.ac.uk/QuickGO/GTerm?id=GO:0097228), | | |
| **GOTERM_MF_DIRECT** | [calcium ion binding](http://www.ebi.ac.uk/QuickGO/GTerm?id=GO:0005509), | | |
| **INTERPRO** | [EF-hand domain](https://www.ebi.ac.uk/interpro/entry/InterPro/IPR002048), [EF-hand-like domain](https://www.ebi.ac.uk/interpro/entry/InterPro/IPR011992), | | |
| **SMART** | [EFh](http://smart.embl.de/smart/do_annotation.pl?DOMAIN=SM00054), | | |
| **UP_KW_CELLULAR_COMPONENT** | [Cytoskeleton](http://www.uniprot.org/keywords/?query=KW-0206), [Flagellum](http://www.uniprot.org/keywords/?query=KW-0282), [Cytoplasm](http://www.uniprot.org/keywords/?query=KW-0963), [Cell projection](http://www.uniprot.org/keywords/?query=KW-0966), [Cilium](http://www.uniprot.org/keywords/?query=KW-0969), | | |
| **UP_KW_DOMAIN** | [Repeat](http://www.uniprot.org/keywords/?query=KW-0677), | | |
| **UP_SEQ_FEATURE** | COMPBIAS:Polar residues, DOMAIN:EF-hand, DOMAIN:EF-hand 1, DOMAIN:EF-hand 2, REGION:Disordered, | | |
| **EPHA4** | [**EPH receptor A4(EPHA4)**](https://david.ncifcrf.gov/geneReportFull.jsp?rowids=2043) | [**Related Genes**](https://david.ncifcrf.gov/relatedGenes.jsp?id=2043) | [**Homo sapiens**](http://www.ncbi.nlm.nih.gov/Taxonomy/Browser/wwwtax.cgi?name=Homo%20sapiens) |
| **BIOCARTA** | [Eph Kinases and ephrins support platelet aggregation](https://david.ncifcrf.gov/biocarta.jsp?path=h_ephA4Pathway$Eph%20Kinases%20and%20ephrins%20support%20platelet%20aggregation&termId=30000099&source=biocarta), | | |
| **GOTERM_BP_DIRECT** | [cell adhesion](http://www.ebi.ac.uk/QuickGO/GTerm?id=GO:0007155), [negative regulation of cell adhesion](http://www.ebi.ac.uk/QuickGO/GTerm?id=GO:0007162), [transmembrane receptor protein tyrosine kinase signaling pathway](http://www.ebi.ac.uk/QuickGO/GTerm?id=GO:0007169), [multicellular organism development](http://www.ebi.ac.uk/QuickGO/GTerm?id=GO:0007275), [axon guidance](http://www.ebi.ac.uk/QuickGO/GTerm?id=GO:0007411), [adult walking behavior](http://www.ebi.ac.uk/QuickGO/GTerm?id=GO:0007628), [motor neuron axon guidance](http://www.ebi.ac.uk/QuickGO/GTerm?id=GO:0008045), [positive regulation of cell proliferation](http://www.ebi.ac.uk/QuickGO/GTerm?id=GO:0008284), [glial cell migration](http://www.ebi.ac.uk/QuickGO/GTerm?id=GO:0008347), [negative regulation of epithelial to mesenchymal transition](http://www.ebi.ac.uk/QuickGO/GTerm?id=GO:0010719), [negative regulation of neuron projection development](http://www.ebi.ac.uk/QuickGO/GTerm?id=GO:0010977), [peptidyl-tyrosine phosphorylation](http://www.ebi.ac.uk/QuickGO/GTerm?id=GO:0018108), [corticospinal tract morphogenesis](http://www.ebi.ac.uk/QuickGO/GTerm?id=GO:0021957), [positive regulation of cell migration](http://www.ebi.ac.uk/QuickGO/GTerm?id=GO:0030335), [negative regulation of cell migration](http://www.ebi.ac.uk/QuickGO/GTerm?id=GO:0030336), [positive regulation of kinase activity](http://www.ebi.ac.uk/QuickGO/GTerm?id=GO:0033674), [adherens junction organization](http://www.ebi.ac.uk/QuickGO/GTerm?id=GO:0034332), [regulation of GTPase activity](http://www.ebi.ac.uk/QuickGO/GTerm?id=GO:0043087), [positive regulation of JUN kinase activity](http://www.ebi.ac.uk/QuickGO/GTerm?id=GO:0043507), [positive regulation of cell adhesion](http://www.ebi.ac.uk/QuickGO/GTerm?id=GO:0045785), [protein autophosphorylation](http://www.ebi.ac.uk/QuickGO/GTerm?id=GO:0046777), [ephrin receptor signaling pathway](http://www.ebi.ac.uk/QuickGO/GTerm?id=GO:0048013), [negative regulation of axon regeneration](http://www.ebi.ac.uk/QuickGO/GTerm?id=GO:0048681), [regulation of astrocyte differentiation](http://www.ebi.ac.uk/QuickGO/GTerm?id=GO:0048710), [regulation of axonogenesis](http://www.ebi.ac.uk/QuickGO/GTerm?id=GO:0050770), [positive regulation of dendrite morphogenesis](http://www.ebi.ac.uk/QuickGO/GTerm?id=GO:0050775), [protein stabilization](http://www.ebi.ac.uk/QuickGO/GTerm?id=GO:0050821), [regulation of dendritic spine morphogenesis](http://www.ebi.ac.uk/QuickGO/GTerm?id=GO:0061001), [positive regulation of protein tyrosine kinase activity](http://www.ebi.ac.uk/QuickGO/GTerm?id=GO:0061098), [negative regulation of ERK1 and ERK2 cascade](http://www.ebi.ac.uk/QuickGO/GTerm?id=GO:0070373), [nephric duct morphogenesis](http://www.ebi.ac.uk/QuickGO/GTerm?id=GO:0072178), [cochlea development](http://www.ebi.ac.uk/QuickGO/GTerm?id=GO:0090102), [fasciculation of sensory neuron axon](http://www.ebi.ac.uk/QuickGO/GTerm?id=GO:0097155), [fasciculation of motor neuron axon](http://www.ebi.ac.uk/QuickGO/GTerm?id=GO:0097156), [neuron projection guidance](http://www.ebi.ac.uk/QuickGO/GTerm?id=GO:0097485), [synapse disassembly](http://www.ebi.ac.uk/QuickGO/GTerm?id=GO:0098883), [negative regulation of cellular response to hypoxia](http://www.ebi.ac.uk/QuickGO/GTerm?id=GO:1900038), [negative regulation of long-term synaptic potentiation](http://www.ebi.ac.uk/QuickGO/GTerm?id=GO:1900272), [positive regulation of beta-amyloid formation](http://www.ebi.ac.uk/QuickGO/GTerm?id=GO:1902004), [positive regulation of aspartic-type endopeptidase activity involved in amyloid precursor protein catabolic process](http://www.ebi.ac.uk/QuickGO/GTerm?id=GO:1902961), [negative regulation of proteolysis involved in cellular protein catabolic process](http://www.ebi.ac.uk/QuickGO/GTerm?id=GO:1903051), [cellular response to beta-amyloid](http://www.ebi.ac.uk/QuickGO/GTerm?id=GO:1904646), [regulation of modification of synaptic structure](http://www.ebi.ac.uk/QuickGO/GTerm?id=GO:1905244), [positive regulation of Rho guanyl-nucleotide exchange factor activity](http://www.ebi.ac.uk/QuickGO/GTerm?id=GO:2001108), | | |
| **GOTERM_CC_DIRECT** | [cytoplasm](http://www.ebi.ac.uk/QuickGO/GTerm?id=GO:0005737), [mitochondrial outer membrane](http://www.ebi.ac.uk/QuickGO/GTerm?id=GO:0005741), [endoplasmic reticulum](http://www.ebi.ac.uk/QuickGO/GTerm?id=GO:0005783), [Golgi apparatus](http://www.ebi.ac.uk/QuickGO/GTerm?id=GO:0005794), [plasma membrane](http://www.ebi.ac.uk/QuickGO/GTerm?id=GO:0005886), [integral component of plasma membrane](http://www.ebi.ac.uk/QuickGO/GTerm?id=GO:0005887), [adherens junction](http://www.ebi.ac.uk/QuickGO/GTerm?id=GO:0005912), [cell surface](http://www.ebi.ac.uk/QuickGO/GTerm?id=GO:0009986), [postsynaptic density](http://www.ebi.ac.uk/QuickGO/GTerm?id=GO:0014069), [integral component of membrane](http://www.ebi.ac.uk/QuickGO/GTerm?id=GO:0016021), [filopodium](http://www.ebi.ac.uk/QuickGO/GTerm?id=GO:0030175), [axon](http://www.ebi.ac.uk/QuickGO/GTerm?id=GO:0030424), [dendrite](http://www.ebi.ac.uk/QuickGO/GTerm?id=GO:0030425), [neuromuscular junction](http://www.ebi.ac.uk/QuickGO/GTerm?id=GO:0031594), [early endosome membrane](http://www.ebi.ac.uk/QuickGO/GTerm?id=GO:0031901), [neuron projection](http://www.ebi.ac.uk/QuickGO/GTerm?id=GO:0043005), [dendritic spine](http://www.ebi.ac.uk/QuickGO/GTerm?id=GO:0043197), [dendritic shaft](http://www.ebi.ac.uk/QuickGO/GTerm?id=GO:0043198), [perikaryon](http://www.ebi.ac.uk/QuickGO/GTerm?id=GO:0043204), [receptor complex](http://www.ebi.ac.uk/QuickGO/GTerm?id=GO:0043235), [axon terminus](http://www.ebi.ac.uk/QuickGO/GTerm?id=GO:0043679), [axonal growth cone](http://www.ebi.ac.uk/QuickGO/GTerm?id=GO:0044295), [Schaffer collateral - CA1 synapse](http://www.ebi.ac.uk/QuickGO/GTerm?id=GO:0098685), [postsynaptic density membrane](http://www.ebi.ac.uk/QuickGO/GTerm?id=GO:0098839), [glutamatergic synapse](http://www.ebi.ac.uk/QuickGO/GTerm?id=GO:0098978), [integral component of postsynaptic membrane](http://www.ebi.ac.uk/QuickGO/GTerm?id=GO:0099055), [integral component of presynaptic membrane](http://www.ebi.ac.uk/QuickGO/GTerm?id=GO:0099056), | | |
| **GOTERM_MF_DIRECT** | [beta-amyloid binding](http://www.ebi.ac.uk/QuickGO/GTerm?id=GO:0001540), [protein kinase activity](http://www.ebi.ac.uk/QuickGO/GTerm?id=GO:0004672), [protein serine/threonine/tyrosine kinase activity](http://www.ebi.ac.uk/QuickGO/GTerm?id=GO:0004712), [transmembrane receptor protein tyrosine kinase activity](http://www.ebi.ac.uk/QuickGO/GTerm?id=GO:0004714), [ephrin receptor activity](http://www.ebi.ac.uk/QuickGO/GTerm?id=GO:0005003), [GPI-linked ephrin receptor activity](http://www.ebi.ac.uk/QuickGO/GTerm?id=GO:0005004), [transmembrane-ephrin receptor activity](http://www.ebi.ac.uk/QuickGO/GTerm?id=GO:0005005), [protein binding](http://www.ebi.ac.uk/QuickGO/GTerm?id=GO:0005515), [ATP binding](http://www.ebi.ac.uk/QuickGO/GTerm?id=GO:0005524), [kinase activity](http://www.ebi.ac.uk/QuickGO/GTerm?id=GO:0016301), [PH domain binding](http://www.ebi.ac.uk/QuickGO/GTerm?id=GO:0042731), [identical protein binding](http://www.ebi.ac.uk/QuickGO/GTerm?id=GO:0042802), [ephrin receptor binding](http://www.ebi.ac.uk/QuickGO/GTerm?id=GO:0046875), [DH domain binding](http://www.ebi.ac.uk/QuickGO/GTerm?id=GO:0097161), [protein tyrosine kinase binding](http://www.ebi.ac.uk/QuickGO/GTerm?id=GO:1990782), | | |
| **INTERPRO** | [Protein kinase, catalytic domain](https://www.ebi.ac.uk/interpro/entry/InterPro/IPR000719), [Ephrin receptor ligand binding domain](https://www.ebi.ac.uk/interpro/entry/InterPro/IPR001090), [Serine-threonine/tyrosine-protein kinase catalytic domain](https://www.ebi.ac.uk/interpro/entry/InterPro/IPR001245), [Tyrosine-protein kinase, receptor class V, conserved site](https://www.ebi.ac.uk/interpro/entry/InterPro/IPR001426), [Sterile alpha motif domain](https://www.ebi.ac.uk/interpro/entry/InterPro/IPR001660), [Fibronectin, type III](https://www.ebi.ac.uk/interpro/entry/InterPro/IPR003961), [Tyrosine-protein kinase, active site](https://www.ebi.ac.uk/interpro/entry/InterPro/IPR008266), [Galactose-binding domain-like](https://www.ebi.ac.uk/interpro/entry/InterPro/IPR008979), [Protein kinase-like domain](https://www.ebi.ac.uk/interpro/entry/InterPro/IPR011009), [Sterile alpha motif/pointed domain](https://www.ebi.ac.uk/interpro/entry/InterPro/IPR013761), [Immunoglobulin-like fold](https://www.ebi.ac.uk/interpro/entry/InterPro/IPR013783), [Tyrosine-protein kinase, ephrin receptor](https://www.ebi.ac.uk/interpro/entry/InterPro/IPR016257), [Protein kinase, ATP binding site](https://www.ebi.ac.uk/interpro/entry/InterPro/IPR017441), [Tyrosine-protein kinase, catalytic domain](https://www.ebi.ac.uk/interpro/entry/InterPro/IPR020635), | | |
| **KEGG_PATHWAY** | [Axon guidance](https://david.ncifcrf.gov/kegg.jsp?path=hsa04360$Axon%20guidance&termId=520047989&source=kegg), | | |
| **PIR_SUPERFAMILY** | [tyrosine-protein kinase, ephrin receptor type](http://pir.georgetown.edu/cgi-bin/ipcSF?id=PIRSF000666), | | |
| **SMART** | [FN3](http://smart.embl.de/smart/do_annotation.pl?DOMAIN=SM00060), [TyrKc](http://smart.embl.de/smart/do_annotation.pl?DOMAIN=SM00219), [S_TKc](http://smart.embl.de/smart/do_annotation.pl?DOMAIN=SM00220), [SAM](http://smart.embl.de/smart/do_annotation.pl?DOMAIN=SM00454), [EPH_lbd](http://smart.embl.de/smart/do_annotation.pl?DOMAIN=SM00615), | | |
| **UP_KW_BIOLOGICAL_PROCESS** | [Cell adhesion](http://www.uniprot.org/keywords/?query=KW-0130), [Neurogenesis](http://www.uniprot.org/keywords/?query=KW-0524), | | |
| **UP_KW_CELLULAR_COMPONENT** | [Membrane](http://www.uniprot.org/keywords/?query=KW-0472), [Postsynaptic cell membrane](http://www.uniprot.org/keywords/?query=KW-0628), [Synapse](http://www.uniprot.org/keywords/?query=KW-0770), [Cell junction](http://www.uniprot.org/keywords/?query=KW-0965), [Cell projection](http://www.uniprot.org/keywords/?query=KW-0966), [Endosome](http://www.uniprot.org/keywords/?query=KW-0967), [Cell membrane](http://www.uniprot.org/keywords/?query=KW-1003), | | |
| **UP_KW_DOMAIN** | [Repeat](http://www.uniprot.org/keywords/?query=KW-0677), [Signal](http://www.uniprot.org/keywords/?query=KW-0732), [Transmembrane](http://www.uniprot.org/keywords/?query=KW-0812), [Transmembrane helix](http://www.uniprot.org/keywords/?query=KW-1133), | | |
| **UP_KW_LIGAND** | [ATP-binding](http://www.uniprot.org/keywords/?query=KW-0067), [Nucleotide-binding](http://www.uniprot.org/keywords/?query=KW-0547), | | |
| **UP_KW_MOLECULAR_FUNCTION** | [Developmental protein](http://www.uniprot.org/keywords/?query=KW-0217), [Kinase](http://www.uniprot.org/keywords/?query=KW-0418), [Receptor](http://www.uniprot.org/keywords/?query=KW-0675), [Transferase](http://www.uniprot.org/keywords/?query=KW-0808), [Tyrosine-protein kinase](http://www.uniprot.org/keywords/?query=KW-0829), [Developmental protein](http://www.uniprot.org/keywords/?query=KW-9996), | | |
| **UP_KW_PTM** | [Glycoprotein](http://www.uniprot.org/keywords/?query=KW-0325), [Phosphoprotein](http://www.uniprot.org/keywords/?query=KW-0597), | | |
| **UP_SEQ_FEATURE** | ACT_SITE:Proton acceptor, BINDING:ATP, CARBOHYD:N-linked (GlcNAc...) asparagine, DOMAIN:Eph LBD, DOMAIN:Fibronectin type-III, DOMAIN:Fibronectin type-III 1, DOMAIN:Fibronectin type-III 2, DOMAIN:Protein kinase, DOMAIN:SAM, MOTIF:PDZ-binding, MUTAGEN:E->A: 10-fold reduced affinity for EFNB2; when associated with A-40., MUTAGEN:Q->A: 10-fold reduced affinity for EFNB2; when associated with A-42., NP_BIND:ATP, TOPO_DOM:Cytoplasmic, TOPO_DOM:Extracellular, TRANSMEM:Helical, | | |
| **EMC1** | [**ER membrane protein complex subunit 1(EMC1)**](https://david.ncifcrf.gov/geneReportFull.jsp?rowids=23065) | [**Related Genes**](https://david.ncifcrf.gov/relatedGenes.jsp?id=23065) | [**Homo sapiens**](http://www.ncbi.nlm.nih.gov/Taxonomy/Browser/wwwtax.cgi?name=Homo%20sapiens) |
| **COG_ONTOLOGY** | [Function unknown](http://www.ncbi.nlm.nih.gov/COG/new/), | | |
| **GOTERM_BP_DIRECT** | [protein insertion into ER membrane by stop-transfer membrane-anchor sequence](http://www.ebi.ac.uk/QuickGO/GTerm?id=GO:0045050), [tail-anchored membrane protein insertion into ER membrane](http://www.ebi.ac.uk/QuickGO/GTerm?id=GO:0071816), | | |
| **GOTERM_CC_DIRECT** | [endoplasmic reticulum membrane](http://www.ebi.ac.uk/QuickGO/GTerm?id=GO:0005789), [integral component of membrane](http://www.ebi.ac.uk/QuickGO/GTerm?id=GO:0016021), [integral component of endoplasmic reticulum membrane](http://www.ebi.ac.uk/QuickGO/GTerm?id=GO:0030176), [macromolecular complex](http://www.ebi.ac.uk/QuickGO/GTerm?id=GO:0032991), [ER membrane protein complex](http://www.ebi.ac.uk/QuickGO/GTerm?id=GO:0072546), | | |
| **GOTERM_MF_DIRECT** | [membrane insertase activity](http://www.ebi.ac.uk/QuickGO/GTerm?id=GO:0032977), | | |
| **INTERPRO** | [Pyrrolo-quinoline quinone repeat](https://www.ebi.ac.uk/interpro/entry/InterPro/IPR002372), [Quinonprotein alcohol dehydrogenase-like superfamily](https://www.ebi.ac.uk/interpro/entry/InterPro/IPR011047), [Domain of unknown function DUF1620](https://www.ebi.ac.uk/interpro/entry/InterPro/IPR011678), [ER membrane protein complex subunit 1](https://www.ebi.ac.uk/interpro/entry/InterPro/IPR026895), | | |
| **OMIM_DISEASE** | [Cerebellar atrophy, visual impairment, and psychomotor retardation](http://omim.org/entry/616875), | | |
| **UP_KW_CELLULAR_COMPONENT** | [Endoplasmic reticulum](http://www.uniprot.org/keywords/?query=KW-0256), [Membrane](http://www.uniprot.org/keywords/?query=KW-0472), | | |
| **UP_KW_DISEASE** | [Disease variant](http://www.uniprot.org/keywords/?query=KW-0225), [Neurodegeneration](http://www.uniprot.org/keywords/?query=KW-0523), | | |
| **UP_KW_DOMAIN** | [Signal](http://www.uniprot.org/keywords/?query=KW-0732), [Transmembrane](http://www.uniprot.org/keywords/?query=KW-0812), [Transmembrane helix](http://www.uniprot.org/keywords/?query=KW-1133), | | |
| **UP_KW_PTM** | [Glycoprotein](http://www.uniprot.org/keywords/?query=KW-0325), [Disulfide bond](http://www.uniprot.org/keywords/?query=KW-1015), | | |
| **UP_SEQ_FEATURE** | CARBOHYD:N-linked (GlcNAc...) asparagine, DOMAIN:EMC1_C, TOPO_DOM:Cytoplasmic, TOPO_DOM:Lumenal, TRANSMEM:Helical, | | |
| **FBXW2** | [**F-box and WD repeat domain containing 2(FBXW2)**](https://david.ncifcrf.gov/geneReportFull.jsp?rowids=26190) | [**Related Genes**](https://david.ncifcrf.gov/relatedGenes.jsp?id=26190) | [**Homo sapiens**](http://www.ncbi.nlm.nih.gov/Taxonomy/Browser/wwwtax.cgi?name=Homo%20sapiens) |
| **GOTERM_BP_DIRECT** | [protein polyubiquitination](http://www.ebi.ac.uk/QuickGO/GTerm?id=GO:0000209), [cellular protein modification process](http://www.ebi.ac.uk/QuickGO/GTerm?id=GO:0006464), [proteolysis](http://www.ebi.ac.uk/QuickGO/GTerm?id=GO:0006508), [protein ubiquitination](http://www.ebi.ac.uk/QuickGO/GTerm?id=GO:0016567), [post-translational protein modification](http://www.ebi.ac.uk/QuickGO/GTerm?id=GO:0043687), | | |
| **GOTERM_CC_DIRECT** | [cytosol](http://www.ebi.ac.uk/QuickGO/GTerm?id=GO:0005829), | | |
| **GOTERM_MF_DIRECT** | [ubiquitin-protein transferase activity](http://www.ebi.ac.uk/QuickGO/GTerm?id=GO:0004842), [protein binding](http://www.ebi.ac.uk/QuickGO/GTerm?id=GO:0005515), | | |
| **INTERPRO** | [WD40 repeat](https://www.ebi.ac.uk/interpro/entry/InterPro/IPR001680), [F-box domain, cyclin-like](https://www.ebi.ac.uk/interpro/entry/InterPro/IPR001810), [WD40/YVTN repeat-like-containing domain](https://www.ebi.ac.uk/interpro/entry/InterPro/IPR015943), [WD40-repeat-containing domain](https://www.ebi.ac.uk/interpro/entry/InterPro/IPR017986), [WD40 repeat, conserved site](https://www.ebi.ac.uk/interpro/entry/InterPro/IPR019775), [G-protein beta WD-40 repeat](https://www.ebi.ac.uk/interpro/entry/InterPro/IPR020472), | | |
| **SMART** | [FBOX](http://smart.embl.de/smart/do_annotation.pl?DOMAIN=SM00256), [WD40](http://smart.embl.de/smart/do_annotation.pl?DOMAIN=SM00320), | | |
| **UP_KW_BIOLOGICAL_PROCESS** | [Ubl conjugation pathway](http://www.uniprot.org/keywords/?query=KW-0833), | | |
| **UP_KW_DOMAIN** | [Repeat](http://www.uniprot.org/keywords/?query=KW-0677), [WD repeat](http://www.uniprot.org/keywords/?query=KW-0853), | | |
| **UP_KW_PTM** | [Acetylation](http://www.uniprot.org/keywords/?query=KW-0007), | | |
| **UP_SEQ_FEATURE** | DOMAIN:F-box, REPEAT:WD, REPEAT:WD 1, REPEAT:WD 2, REPEAT:WD 3, REPEAT:WD 4, REPEAT:WD 5, REPEAT:WD 6, REPEAT:WD 7, | | |
| **FBXW7** | [**F-box and WD repeat domain containing 7(FBXW7)**](https://david.ncifcrf.gov/geneReportFull.jsp?rowids=55294) | [**Related Genes**](https://david.ncifcrf.gov/relatedGenes.jsp?id=55294) | [**Homo sapiens**](http://www.ncbi.nlm.nih.gov/Taxonomy/Browser/wwwtax.cgi?name=Homo%20sapiens) |
| **BIOCARTA** | [Cyclin E Destruction Pathway](https://david.ncifcrf.gov/biocarta.jsp?path=h_fbw7Pathway$Cyclin%20E%20Destruction%20Pathway&termId=30000112&source=biocarta), | | |
| **GOTERM_BP_DIRECT** | [protein polyubiquitination](http://www.ebi.ac.uk/QuickGO/GTerm?id=GO:0000209), [vasculogenesis](http://www.ebi.ac.uk/QuickGO/GTerm?id=GO:0001570), [vasculature development](http://www.ebi.ac.uk/QuickGO/GTerm?id=GO:0001944), [DNA repair](http://www.ebi.ac.uk/QuickGO/GTerm?id=GO:0006281), [cellular response to DNA damage stimulus](http://www.ebi.ac.uk/QuickGO/GTerm?id=GO:0006974), [sister chromatid cohesion](http://www.ebi.ac.uk/QuickGO/GTerm?id=GO:0007062), [Notch signaling pathway](http://www.ebi.ac.uk/QuickGO/GTerm?id=GO:0007219), [negative regulation of gene expression](http://www.ebi.ac.uk/QuickGO/GTerm?id=GO:0010629), [negative regulation of triglyceride biosynthetic process](http://www.ebi.ac.uk/QuickGO/GTerm?id=GO:0010868), [regulation of lipid storage](http://www.ebi.ac.uk/QuickGO/GTerm?id=GO:0010883), [ubiquitin homeostasis](http://www.ebi.ac.uk/QuickGO/GTerm?id=GO:0010992), [viral process](http://www.ebi.ac.uk/QuickGO/GTerm?id=GO:0016032), [protein ubiquitination](http://www.ebi.ac.uk/QuickGO/GTerm?id=GO:0016567), [lung development](http://www.ebi.ac.uk/QuickGO/GTerm?id=GO:0030324), [SCF-dependent proteasomal ubiquitin-dependent protein catabolic process](http://www.ebi.ac.uk/QuickGO/GTerm?id=GO:0031146), [positive regulation of protein ubiquitination](http://www.ebi.ac.uk/QuickGO/GTerm?id=GO:0031398), [protein destabilization](http://www.ebi.ac.uk/QuickGO/GTerm?id=GO:0031648), [regulation of protein localization](http://www.ebi.ac.uk/QuickGO/GTerm?id=GO:0032880), [cellular response to UV](http://www.ebi.ac.uk/QuickGO/GTerm?id=GO:0034644), [regulation of circadian rhythm](http://www.ebi.ac.uk/QuickGO/GTerm?id=GO:0042752), [proteasome-mediated ubiquitin-dependent protein catabolic process](http://www.ebi.ac.uk/QuickGO/GTerm?id=GO:0043161), [post-translational protein modification](http://www.ebi.ac.uk/QuickGO/GTerm?id=GO:0043687), [positive regulation of epidermal growth factor-activated receptor activity](http://www.ebi.ac.uk/QuickGO/GTerm?id=GO:0045741), [negative regulation of Notch signaling pathway](http://www.ebi.ac.uk/QuickGO/GTerm?id=GO:0045746), [rhythmic process](http://www.ebi.ac.uk/QuickGO/GTerm?id=GO:0048511), [protein stabilization](http://www.ebi.ac.uk/QuickGO/GTerm?id=GO:0050821), [positive regulation of ubiquitin-protein transferase activity](http://www.ebi.ac.uk/QuickGO/GTerm?id=GO:0051443), [lipid homeostasis](http://www.ebi.ac.uk/QuickGO/GTerm?id=GO:0055088), [positive regulation of ERK1 and ERK2 cascade](http://www.ebi.ac.uk/QuickGO/GTerm?id=GO:0070374), [regulation of cell migration involved in sprouting angiogenesis](http://www.ebi.ac.uk/QuickGO/GTerm?id=GO:0090049), [positive regulation of proteasomal protein catabolic process](http://www.ebi.ac.uk/QuickGO/GTerm?id=GO:1901800), [regulation of cell cycle G1/S phase transition](http://www.ebi.ac.uk/QuickGO/GTerm?id=GO:1902806), [negative regulation of RNA polymerase II regulatory region sequence-specific DNA binding](http://www.ebi.ac.uk/QuickGO/GTerm?id=GO:1903026), [regulation of mitophagy](http://www.ebi.ac.uk/QuickGO/GTerm?id=GO:1903146), [positive regulation of oxidative stress-induced neuron intrinsic apoptotic signaling pathway](http://www.ebi.ac.uk/QuickGO/GTerm?id=GO:1903378), [positive regulation of protein targeting to mitochondrion](http://www.ebi.ac.uk/QuickGO/GTerm?id=GO:1903955), [positive regulation of protein ubiquitination involved in ubiquitin-dependent protein catabolic process](http://www.ebi.ac.uk/QuickGO/GTerm?id=GO:2000060), [negative regulation of hepatocyte proliferation](http://www.ebi.ac.uk/QuickGO/GTerm?id=GO:2000346), [negative regulation of SREBP signaling pathway](http://www.ebi.ac.uk/QuickGO/GTerm?id=GO:2000639), [negative regulation of osteoclast development](http://www.ebi.ac.uk/QuickGO/GTerm?id=GO:2001205), | | |
| **GOTERM_CC_DIRECT** | [nucleus](http://www.ebi.ac.uk/QuickGO/GTerm?id=GO:0005634), [nucleoplasm](http://www.ebi.ac.uk/QuickGO/GTerm?id=GO:0005654), [chromosome](http://www.ebi.ac.uk/QuickGO/GTerm?id=GO:0005694), [nucleolus](http://www.ebi.ac.uk/QuickGO/GTerm?id=GO:0005730), [cytoplasm](http://www.ebi.ac.uk/QuickGO/GTerm?id=GO:0005737), [mitochondrion](http://www.ebi.ac.uk/QuickGO/GTerm?id=GO:0005739), [endoplasmic reticulum](http://www.ebi.ac.uk/QuickGO/GTerm?id=GO:0005783), [Golgi apparatus](http://www.ebi.ac.uk/QuickGO/GTerm?id=GO:0005794), [cytosol](http://www.ebi.ac.uk/QuickGO/GTerm?id=GO:0005829), [SCF ubiquitin ligase complex](http://www.ebi.ac.uk/QuickGO/GTerm?id=GO:0019005), [macromolecular complex](http://www.ebi.ac.uk/QuickGO/GTerm?id=GO:0032991), [perinuclear region of cytoplasm](http://www.ebi.ac.uk/QuickGO/GTerm?id=GO:0048471), [Parkin-FBXW7-Cul1 ubiquitin ligase complex](http://www.ebi.ac.uk/QuickGO/GTerm?id=GO:1990452), | | |
| **GOTERM_MF_DIRECT** | [protein binding](http://www.ebi.ac.uk/QuickGO/GTerm?id=GO:0005515), [cyclin binding](http://www.ebi.ac.uk/QuickGO/GTerm?id=GO:0030332), [protein binding, bridging](http://www.ebi.ac.uk/QuickGO/GTerm?id=GO:0030674), [ubiquitin protein ligase binding](http://www.ebi.ac.uk/QuickGO/GTerm?id=GO:0031625), [identical protein binding](http://www.ebi.ac.uk/QuickGO/GTerm?id=GO:0042802), [ubiquitin binding](http://www.ebi.ac.uk/QuickGO/GTerm?id=GO:0043130), [phosphothreonine binding](http://www.ebi.ac.uk/QuickGO/GTerm?id=GO:0050816), [ubiquitin-protein transferase activator activity](http://www.ebi.ac.uk/QuickGO/GTerm?id=GO:0097027), | | |
| **INTERPRO** | [WD40 repeat](https://www.ebi.ac.uk/interpro/entry/InterPro/IPR001680), [F-box domain, cyclin-like](https://www.ebi.ac.uk/interpro/entry/InterPro/IPR001810), [WD40/YVTN repeat-like-containing domain](https://www.ebi.ac.uk/interpro/entry/InterPro/IPR015943), [WD40-repeat-containing domain](https://www.ebi.ac.uk/interpro/entry/InterPro/IPR017986), [WD40 repeat, conserved site](https://www.ebi.ac.uk/interpro/entry/InterPro/IPR019775), [G-protein beta WD-40 repeat](https://www.ebi.ac.uk/interpro/entry/InterPro/IPR020472), | | |
| **KEGG_PATHWAY** | [Ubiquitin mediated proteolysis](https://david.ncifcrf.gov/kegg.jsp?path=hsa04120$Ubiquitin%20mediated%20proteolysis&termId=520047961&source=kegg), | | |
| **SMART** | [FBOX](http://smart.embl.de/smart/do_annotation.pl?DOMAIN=SM00256), [WD40](http://smart.embl.de/smart/do_annotation.pl?DOMAIN=SM00320), | | |
| **UP_KW_BIOLOGICAL_PROCESS** | [Biological rhythms](http://www.uniprot.org/keywords/?query=KW-0090), [DNA damage](http://www.uniprot.org/keywords/?query=KW-0227), [DNA repair](http://www.uniprot.org/keywords/?query=KW-0234), [Ubl conjugation pathway](http://www.uniprot.org/keywords/?query=KW-0833), [Host-virus interaction](http://www.uniprot.org/keywords/?query=KW-0945), | | |
| **UP_KW_CELLULAR_COMPONENT** | [Chromosome](http://www.uniprot.org/keywords/?query=KW-0158), [Nucleus](http://www.uniprot.org/keywords/?query=KW-0539), [Cytoplasm](http://www.uniprot.org/keywords/?query=KW-0963), | | |
| **UP_KW_DOMAIN** | [Repeat](http://www.uniprot.org/keywords/?query=KW-0677), [WD repeat](http://www.uniprot.org/keywords/?query=KW-0853), | | |
| **UP_KW_PTM** | [Phosphoprotein](http://www.uniprot.org/keywords/?query=KW-0597), [Ubl conjugation](http://www.uniprot.org/keywords/?query=KW-0832), | | |
| **UP_SEQ_FEATURE** | COMPBIAS:Acidic residues, COMPBIAS:Basic and acidic residues, COMPBIAS:Polar residues, DOMAIN:F-box, MUTAGEN:ALDELI->DDDEDD: Prevents homodimerization., MUTAGEN:S->A: Abolished phosphorylation by ATM., MUTAGEN:S->A: Does not affect interaction with PIN1., MUTAGEN:S->A: Does not affect phosphorylation by ATM., MUTAGEN:T->A: Impaired interaction with PIN1., REGION:Disordered, REPEAT:WD, REPEAT:WD 1, REPEAT:WD 2, REPEAT:WD 3, REPEAT:WD 4, REPEAT:WD 5, REPEAT:WD 6, REPEAT:WD 7, | | |
| **FIG4** | [**FIG4 phosphoinositide 5-phosphatase(FIG4)**](https://david.ncifcrf.gov/geneReportFull.jsp?rowids=9896) | [**Related Genes**](https://david.ncifcrf.gov/relatedGenes.jsp?id=9896) | [**Homo sapiens**](http://www.ncbi.nlm.nih.gov/Taxonomy/Browser/wwwtax.cgi?name=Homo%20sapiens) |
| **GOTERM_BP_DIRECT** | [protein dephosphorylation](http://www.ebi.ac.uk/QuickGO/GTerm?id=GO:0006470), [phosphatidylinositol biosynthetic process](http://www.ebi.ac.uk/QuickGO/GTerm?id=GO:0006661), [vacuole organization](http://www.ebi.ac.uk/QuickGO/GTerm?id=GO:0007033), [locomotory behavior](http://www.ebi.ac.uk/QuickGO/GTerm?id=GO:0007626), [positive regulation of neuron projection development](http://www.ebi.ac.uk/QuickGO/GTerm?id=GO:0010976), [negative regulation of myelination](http://www.ebi.ac.uk/QuickGO/GTerm?id=GO:0031642), [myelin assembly](http://www.ebi.ac.uk/QuickGO/GTerm?id=GO:0032288), [phosphatidylinositol-3-phosphate biosynthetic process](http://www.ebi.ac.uk/QuickGO/GTerm?id=GO:0036092), [pigmentation](http://www.ebi.ac.uk/QuickGO/GTerm?id=GO:0043473), [phosphatidylinositol dephosphorylation](http://www.ebi.ac.uk/QuickGO/GTerm?id=GO:0046856), [neuron development](http://www.ebi.ac.uk/QuickGO/GTerm?id=GO:0048666), | | |
| **GOTERM_CC_DIRECT** | [Golgi membrane](http://www.ebi.ac.uk/QuickGO/GTerm?id=GO:0000139), [endoplasmic reticulum](http://www.ebi.ac.uk/QuickGO/GTerm?id=GO:0005783), [lipid particle](http://www.ebi.ac.uk/QuickGO/GTerm?id=GO:0005811), [endosome membrane](http://www.ebi.ac.uk/QuickGO/GTerm?id=GO:0010008), [membrane](http://www.ebi.ac.uk/QuickGO/GTerm?id=GO:0016020), [integral component of membrane](http://www.ebi.ac.uk/QuickGO/GTerm?id=GO:0016021), [early endosome membrane](http://www.ebi.ac.uk/QuickGO/GTerm?id=GO:0031901), [late endosome membrane](http://www.ebi.ac.uk/QuickGO/GTerm?id=GO:0031902), [intracellular membrane-bounded organelle](http://www.ebi.ac.uk/QuickGO/GTerm?id=GO:0043231), [recycling endosome](http://www.ebi.ac.uk/QuickGO/GTerm?id=GO:0055037), | | |
| **GOTERM_MF_DIRECT** | [phosphatidylinositol-3-phosphatase activity](http://www.ebi.ac.uk/QuickGO/GTerm?id=GO:0004438), [phosphatidylinositol-4,5-bisphosphate 5-phosphatase activity](http://www.ebi.ac.uk/QuickGO/GTerm?id=GO:0004439), [protein binding](http://www.ebi.ac.uk/QuickGO/GTerm?id=GO:0005515), [phosphatidylinositol-3,4,5-trisphosphate 5-phosphatase activity](http://www.ebi.ac.uk/QuickGO/GTerm?id=GO:0034485), [phosphatidylinositol-4-phosphate phosphatase activity](http://www.ebi.ac.uk/QuickGO/GTerm?id=GO:0043812), [phosphatidylinositol-3,5-bisphosphate 5-phosphatase activity](http://www.ebi.ac.uk/QuickGO/GTerm?id=GO:0043813), | | |
| **INTERPRO** | [Synaptojanin, N-terminal](https://www.ebi.ac.uk/interpro/entry/InterPro/IPR002013), | | |
| **KEGG_PATHWAY** | [Inositol phosphate metabolism](https://david.ncifcrf.gov/kegg.jsp?path=hsa00562$Inositol%20phosphate%20metabolism&termId=520047877&source=kegg), [Metabolic pathways](https://david.ncifcrf.gov/kegg.jsp?path=hsa01100$Metabolic%20pathways&termId=520047911&source=kegg), [Amyotrophic lateral sclerosis](https://david.ncifcrf.gov/kegg.jsp?path=hsa05014$Amyotrophic%20lateral%20sclerosis&termId=520048089&source=kegg), [Pathways of neurodegeneration - multiple diseases](https://david.ncifcrf.gov/kegg.jsp?path=hsa05022$Pathways%20of%20neurodegeneration%20-%20multiple%20diseases&termId=520048093&source=kegg), | | |
| **OMIM_DISEASE** | [Yunis-Varon syndrome](http://omim.org/entry/216340), [Charcot-Marie-Tooth disease, type 4J](http://omim.org/entry/611228), [Amyotrophic lateral sclerosis 11](http://omim.org/entry/612577), [Polymicrogyria, bilateral temporooccipital](http://omim.org/entry/612691), | | |
| **UP_KW_CELLULAR_COMPONENT** | [Membrane](http://www.uniprot.org/keywords/?query=KW-0472), [Endosome](http://www.uniprot.org/keywords/?query=KW-0967), | | |
| **UP_KW_DISEASE** | [Amyotrophic lateral sclerosis](http://www.uniprot.org/keywords/?query=KW-0036), [Charcot-Marie-Tooth disease](http://www.uniprot.org/keywords/?query=KW-0144), [Disease variant](http://www.uniprot.org/keywords/?query=KW-0225), [Neurodegeneration](http://www.uniprot.org/keywords/?query=KW-0523), [Neuropathy](http://www.uniprot.org/keywords/?query=KW-0622), | | |
| **UP_KW_DOMAIN** | [Transmembrane](http://www.uniprot.org/keywords/?query=KW-0812), [Transmembrane helix](http://www.uniprot.org/keywords/?query=KW-1133), | | |
| **UP_KW_MOLECULAR_FUNCTION** | [Hydrolase](http://www.uniprot.org/keywords/?query=KW-0378), | | |
| **UP_SEQ_FEATURE** | COMPBIAS:Polar residues, DOMAIN:SAC, MUTAGEN:C->S: Loss of phosphatase activity on PIKFYVE., MUTAGEN:D->A: Loss of activity., REGION:Disordered, TRANSMEM:Helical, | | |
| **FLII** | [**FLII actin remodeling protein(FLII)**](https://david.ncifcrf.gov/geneReportFull.jsp?rowids=2314) | [**Related Genes**](https://david.ncifcrf.gov/relatedGenes.jsp?id=2314) | [**Homo sapiens**](http://www.ncbi.nlm.nih.gov/Taxonomy/Browser/wwwtax.cgi?name=Homo%20sapiens) |
| **COG_ONTOLOGY** | [Function unknown](http://www.ncbi.nlm.nih.gov/COG/new/), | | |
| **GOTERM_BP_DIRECT** | [multicellular organism development](http://www.ebi.ac.uk/QuickGO/GTerm?id=GO:0007275), [actin polymerization or depolymerization](http://www.ebi.ac.uk/QuickGO/GTerm?id=GO:0008154), [actin cytoskeleton organization](http://www.ebi.ac.uk/QuickGO/GTerm?id=GO:0030036), [myofibril assembly](http://www.ebi.ac.uk/QuickGO/GTerm?id=GO:0030239), [actin filament severing](http://www.ebi.ac.uk/QuickGO/GTerm?id=GO:0051014), [barbed-end actin filament capping](http://www.ebi.ac.uk/QuickGO/GTerm?id=GO:0051016), | | |
| **GOTERM_CC_DIRECT** | [nucleus](http://www.ebi.ac.uk/QuickGO/GTerm?id=GO:0005634), [nucleoplasm](http://www.ebi.ac.uk/QuickGO/GTerm?id=GO:0005654), [cytoplasm](http://www.ebi.ac.uk/QuickGO/GTerm?id=GO:0005737), [cytosol](http://www.ebi.ac.uk/QuickGO/GTerm?id=GO:0005829), [brush border](http://www.ebi.ac.uk/QuickGO/GTerm?id=GO:0005903), [focal adhesion](http://www.ebi.ac.uk/QuickGO/GTerm?id=GO:0005925), [actin cytoskeleton](http://www.ebi.ac.uk/QuickGO/GTerm?id=GO:0015629), [centriolar satellite](http://www.ebi.ac.uk/QuickGO/GTerm?id=GO:0034451), | | |
| **GOTERM_MF_DIRECT** | [actin binding](http://www.ebi.ac.uk/QuickGO/GTerm?id=GO:0003779), [protein binding](http://www.ebi.ac.uk/QuickGO/GTerm?id=GO:0005515), [phosphatidylinositol-4,5-bisphosphate binding](http://www.ebi.ac.uk/QuickGO/GTerm?id=GO:0005546), [actin filament binding](http://www.ebi.ac.uk/QuickGO/GTerm?id=GO:0051015), | | |
| **INTERPRO** | [Leucine-rich repeat](https://www.ebi.ac.uk/interpro/entry/InterPro/IPR001611), [Leucine-rich repeat, typical subtype](https://www.ebi.ac.uk/interpro/entry/InterPro/IPR003591), [Villin/Gelsolin](https://www.ebi.ac.uk/interpro/entry/InterPro/IPR007122), [Gelsolin domain](https://www.ebi.ac.uk/interpro/entry/InterPro/IPR007123), | | |
| **SMART** | [GEL](http://smart.embl.de/smart/do_annotation.pl?DOMAIN=SM00262), [LRR_TYP](http://smart.embl.de/smart/do_annotation.pl?DOMAIN=SM00369), | | |
| **UP_KW_BIOLOGICAL_PROCESS** | [Transcription](http://www.uniprot.org/keywords/?query=KW-0804), [Transcription regulation](http://www.uniprot.org/keywords/?query=KW-0805), | | |
| **UP_KW_CELLULAR_COMPONENT** | [Cytoskeleton](http://www.uniprot.org/keywords/?query=KW-0206), [Nucleus](http://www.uniprot.org/keywords/?query=KW-0539), [Cytoplasm](http://www.uniprot.org/keywords/?query=KW-0963), [Cell junction](http://www.uniprot.org/keywords/?query=KW-0965), | | |
| **UP_KW_DOMAIN** | [Leucine-rich repeat](http://www.uniprot.org/keywords/?query=KW-0433), [Repeat](http://www.uniprot.org/keywords/?query=KW-0677), | | |
| **UP_KW_MOLECULAR_FUNCTION** | [Actin-binding](http://www.uniprot.org/keywords/?query=KW-0009), [Activator](http://www.uniprot.org/keywords/?query=KW-0010), [Developmental protein](http://www.uniprot.org/keywords/?query=KW-0217), [Developmental protein](http://www.uniprot.org/keywords/?query=KW-9996), | | |
| **UP_KW_PTM** | [Acetylation](http://www.uniprot.org/keywords/?query=KW-0007), [Phosphoprotein](http://www.uniprot.org/keywords/?query=KW-0597), | | |
| **UP_SEQ_FEATURE** | DOMAIN:Gelsolin-like, MUTAGEN:E->K: No change in ESR1 binding but reduced binding to ACTL6A and reduced coactivator function., MUTAGEN:G->S: No change in binding to ACTL6A or in coactivator function., REGION:Disordered, REGION:Interaction with ACTL6A, REGION:Interaction with LRRFIP1 and LRRFIP2, REPEAT:Gelsolin-like 1, REPEAT:Gelsolin-like 2, REPEAT:Gelsolin-like 3, REPEAT:Gelsolin-like 4, REPEAT:Gelsolin-like 5, REPEAT:LRR 1, REPEAT:LRR 10, REPEAT:LRR 11, REPEAT:LRR 12, REPEAT:LRR 13, REPEAT:LRR 14, REPEAT:LRR 15, REPEAT:LRR 2, REPEAT:LRR 3, REPEAT:LRR 4, REPEAT:LRR 5, REPEAT:LRR 6, REPEAT:LRR 7, REPEAT:LRR 8, REPEAT:LRR 9, | | |
| **GNB3** | [**G protein subunit beta 3(GNB3)**](https://david.ncifcrf.gov/geneReportFull.jsp?rowids=2784) | [**Related Genes**](https://david.ncifcrf.gov/relatedGenes.jsp?id=2784) | [**Homo sapiens**](http://www.ncbi.nlm.nih.gov/Taxonomy/Browser/wwwtax.cgi?name=Homo%20sapiens) |
| **GOTERM_BP_DIRECT** | [protein folding](http://www.ebi.ac.uk/QuickGO/GTerm?id=GO:0006457), [cell volume homeostasis](http://www.ebi.ac.uk/QuickGO/GTerm?id=GO:0006884), [signal transduction](http://www.ebi.ac.uk/QuickGO/GTerm?id=GO:0007165), [G-protein coupled receptor signaling pathway](http://www.ebi.ac.uk/QuickGO/GTerm?id=GO:0007186), [regulation of blood pressure](http://www.ebi.ac.uk/QuickGO/GTerm?id=GO:0008217), [regulation of gene expression](http://www.ebi.ac.uk/QuickGO/GTerm?id=GO:0010468), [regulation of glucose metabolic process](http://www.ebi.ac.uk/QuickGO/GTerm?id=GO:0010906), [regulation of hormone metabolic process](http://www.ebi.ac.uk/QuickGO/GTerm?id=GO:0032350), [regulation of fat cell differentiation](http://www.ebi.ac.uk/QuickGO/GTerm?id=GO:0045598), [regulation of cholesterol metabolic process](http://www.ebi.ac.uk/QuickGO/GTerm?id=GO:0090181), [regulation of triglyceride metabolic process](http://www.ebi.ac.uk/QuickGO/GTerm?id=GO:0090207), [regulation of phospholipid metabolic process](http://www.ebi.ac.uk/QuickGO/GTerm?id=GO:1903725), | | |
| **GOTERM_CC_DIRECT** | [cytoplasm](http://www.ebi.ac.uk/QuickGO/GTerm?id=GO:0005737), [cytosol](http://www.ebi.ac.uk/QuickGO/GTerm?id=GO:0005829), [heterotrimeric G-protein complex](http://www.ebi.ac.uk/QuickGO/GTerm?id=GO:0005834), [plasma membrane](http://www.ebi.ac.uk/QuickGO/GTerm?id=GO:0005886), [dendrite](http://www.ebi.ac.uk/QuickGO/GTerm?id=GO:0030425), [cell body](http://www.ebi.ac.uk/QuickGO/GTerm?id=GO:0044297), [extracellular exosome](http://www.ebi.ac.uk/QuickGO/GTerm?id=GO:0070062), | | |
| **GOTERM_MF_DIRECT** | [GTPase activity](http://www.ebi.ac.uk/QuickGO/GTerm?id=GO:0003924), [protein binding](http://www.ebi.ac.uk/QuickGO/GTerm?id=GO:0005515), [receptor signaling complex scaffold activity](http://www.ebi.ac.uk/QuickGO/GTerm?id=GO:0030159), [spectrin binding](http://www.ebi.ac.uk/QuickGO/GTerm?id=GO:0030507), [G-protein gamma-subunit binding](http://www.ebi.ac.uk/QuickGO/GTerm?id=GO:0031682), [GTPase binding](http://www.ebi.ac.uk/QuickGO/GTerm?id=GO:0051020), | | |
| **INTERPRO** | [G-protein, beta subunit](https://www.ebi.ac.uk/interpro/entry/InterPro/IPR001632), [WD40 repeat](https://www.ebi.ac.uk/interpro/entry/InterPro/IPR001680), [WD40/YVTN repeat-like-containing domain](https://www.ebi.ac.uk/interpro/entry/InterPro/IPR015943), [Guanine nucleotide-binding protein, beta subunit](https://www.ebi.ac.uk/interpro/entry/InterPro/IPR016346), [WD40-repeat-containing domain](https://www.ebi.ac.uk/interpro/entry/InterPro/IPR017986), [WD40 repeat, conserved site](https://www.ebi.ac.uk/interpro/entry/InterPro/IPR019775), [G-protein beta WD-40 repeat](https://www.ebi.ac.uk/interpro/entry/InterPro/IPR020472), | | |
| **KEGG_PATHWAY** | [Ras signaling pathway](https://david.ncifcrf.gov/kegg.jsp?path=hsa04014$Ras%20signaling%20pathway&termId=520047943&source=kegg), [Chemokine signaling pathway](https://david.ncifcrf.gov/kegg.jsp?path=hsa04062$Chemokine%20signaling%20pathway&termId=520047950&source=kegg), [PI3K-Akt signaling pathway](https://david.ncifcrf.gov/kegg.jsp?path=hsa04151$PI3K-Akt%20signaling%20pathway&termId=520047973&source=kegg), [Apelin signaling pathway](https://david.ncifcrf.gov/kegg.jsp?path=hsa04371$Apelin%20signaling%20pathway&termId=520047991&source=kegg), [Circadian entrainment](https://david.ncifcrf.gov/kegg.jsp?path=hsa04713$Circadian%20entrainment&termId=520048026&source=kegg), [Retrograde endocannabinoid signaling](https://david.ncifcrf.gov/kegg.jsp?path=hsa04723$Retrograde%20endocannabinoid%20signaling&termId=520048031&source=kegg), [Glutamatergic synapse](https://david.ncifcrf.gov/kegg.jsp?path=hsa04724$Glutamatergic%20synapse&termId=520048032&source=kegg), [Cholinergic synapse](https://david.ncifcrf.gov/kegg.jsp?path=hsa04725$Cholinergic%20synapse&termId=520048033&source=kegg), [Serotonergic synapse](https://david.ncifcrf.gov/kegg.jsp?path=hsa04726$Serotonergic%20synapse&termId=520048034&source=kegg), [GABAergic synapse](https://david.ncifcrf.gov/kegg.jsp?path=hsa04727$GABAergic%20synapse&termId=520048035&source=kegg), [Dopaminergic synapse](https://david.ncifcrf.gov/kegg.jsp?path=hsa04728$Dopaminergic%20synapse&termId=520048036&source=kegg), [Taste transduction](https://david.ncifcrf.gov/kegg.jsp?path=hsa04742$Taste%20transduction&termId=520048039&source=kegg), [Relaxin signaling pathway](https://david.ncifcrf.gov/kegg.jsp?path=hsa04926$Relaxin%20signaling%20pathway&termId=520048059&source=kegg), [Morphine addiction](https://david.ncifcrf.gov/kegg.jsp?path=hsa05032$Morphine%20addiction&termId=520048096&source=kegg), [Alcoholism](https://david.ncifcrf.gov/kegg.jsp?path=hsa05034$Alcoholism&termId=520048098&source=kegg), [Human cytomegalovirus infection](https://david.ncifcrf.gov/kegg.jsp?path=hsa05163$Human%20cytomegalovirus%20infection&termId=520048119&source=kegg), [Kaposi sarcoma-associated herpesvirus infection](https://david.ncifcrf.gov/kegg.jsp?path=hsa05167$Kaposi%20sarcoma-associated%20herpesvirus%20infection&termId=520048123&source=kegg), [Human immunodeficiency virus 1 infection](https://david.ncifcrf.gov/kegg.jsp?path=hsa05170$Human%20immunodeficiency%20virus%201%20infection&termId=520048126&source=kegg), [Pathways in cancer](https://david.ncifcrf.gov/kegg.jsp?path=hsa05200$Pathways%20in%20cancer&termId=520048128&source=kegg), | | |
| **OMIM_DISEASE** | [Hypertension, essential, susceptibility to](http://omim.org/entry/145500), [Night blindness, congenital stationary, type 1H](http://omim.org/entry/617024), | | |
| **SMART** | [WD40](http://smart.embl.de/smart/do_annotation.pl?DOMAIN=SM00320), | | |
| **UP_KW_DISEASE** | [Disease variant](http://www.uniprot.org/keywords/?query=KW-0225), [Congenital stationary night blindness](http://www.uniprot.org/keywords/?query=KW-1014), | | |
| **UP_KW_DOMAIN** | [Coiled coil](http://www.uniprot.org/keywords/?query=KW-0175), [Repeat](http://www.uniprot.org/keywords/?query=KW-0677), [WD repeat](http://www.uniprot.org/keywords/?query=KW-0853), | | |
| **UP_KW_MOLECULAR_FUNCTION** | [Transducer](http://www.uniprot.org/keywords/?query=KW-0807), | | |
| **UP_SEQ_FEATURE** | REPEAT:WD, REPEAT:WD 1, REPEAT:WD 2, REPEAT:WD 3, REPEAT:WD 4, REPEAT:WD 5, REPEAT:WD 6, REPEAT:WD 7, | | |
| **GARNL3** | [**GTPase activating Rap/RanGAP domain like 3(GARNL3)**](https://david.ncifcrf.gov/geneReportFull.jsp?rowids=84253) | [**Related Genes**](https://david.ncifcrf.gov/relatedGenes.jsp?id=84253) | [**Homo sapiens**](http://www.ncbi.nlm.nih.gov/Taxonomy/Browser/wwwtax.cgi?name=Homo%20sapiens) |
| **GOTERM_BP_DIRECT** | [positive regulation of GTPase activity](http://www.ebi.ac.uk/QuickGO/GTerm?id=GO:0043547), [regulation of small GTPase mediated signal transduction](http://www.ebi.ac.uk/QuickGO/GTerm?id=GO:0051056), [activation of GTPase activity](http://www.ebi.ac.uk/QuickGO/GTerm?id=GO:0090630), | | |
| **GOTERM_CC_DIRECT** | [cytoplasm](http://www.ebi.ac.uk/QuickGO/GTerm?id=GO:0005737), | | |
| **GOTERM_MF_DIRECT** | [GTPase activator activity](http://www.ebi.ac.uk/QuickGO/GTerm?id=GO:0005096), | | |
| **INTERPRO** | [Rap GTPase activating proteins domain](https://www.ebi.ac.uk/interpro/entry/InterPro/IPR000331), [Citron-like](https://www.ebi.ac.uk/interpro/entry/InterPro/IPR001180), | | |
| **SMART** | [CNH](http://smart.embl.de/smart/do_annotation.pl?DOMAIN=SM00036), | | |
| **UP_KW_MOLECULAR_FUNCTION** | [GTPase activation](http://www.uniprot.org/keywords/?query=KW-0343), | | |
| **UP_KW_PTM** | [Phosphoprotein](http://www.uniprot.org/keywords/?query=KW-0597), | | |
| **UP_SEQ_FEATURE** | COMPBIAS:Basic and acidic residues, COMPBIAS:Polar residues, DOMAIN:CNH, DOMAIN:Rap-GAP, REGION:Disordered, | | |
| **HBS1L** | [**HBS1 like translational GTPase(HBS1L)**](https://david.ncifcrf.gov/geneReportFull.jsp?rowids=10767) | [**Related Genes**](https://david.ncifcrf.gov/relatedGenes.jsp?id=10767) | [**Homo sapiens**](http://www.ncbi.nlm.nih.gov/Taxonomy/Browser/wwwtax.cgi?name=Homo%20sapiens) |
| **COG_ONTOLOGY** | [Translation, ribosomal structure and biogenesis](http://www.ncbi.nlm.nih.gov/COG/new/), | | |
| **GOTERM_BP_DIRECT** | [translation](http://www.ebi.ac.uk/QuickGO/GTerm?id=GO:0006412), [translational elongation](http://www.ebi.ac.uk/QuickGO/GTerm?id=GO:0006414), [regulation of translation](http://www.ebi.ac.uk/QuickGO/GTerm?id=GO:0006417), [signal transduction](http://www.ebi.ac.uk/QuickGO/GTerm?id=GO:0007165), [exonucleolytic nuclear-transcribed mRNA catabolic process involved in deadenylation-dependent decay](http://www.ebi.ac.uk/QuickGO/GTerm?id=GO:0043928), | | |
| **GOTERM_CC_DIRECT** | [cytosol](http://www.ebi.ac.uk/QuickGO/GTerm?id=GO:0005829), [membrane](http://www.ebi.ac.uk/QuickGO/GTerm?id=GO:0016020), [integral component of membrane](http://www.ebi.ac.uk/QuickGO/GTerm?id=GO:0016021), [extracellular exosome](http://www.ebi.ac.uk/QuickGO/GTerm?id=GO:0070062), | | |
| **GOTERM_MF_DIRECT** | [translation elongation factor activity](http://www.ebi.ac.uk/QuickGO/GTerm?id=GO:0003746), [GTPase activity](http://www.ebi.ac.uk/QuickGO/GTerm?id=GO:0003924), [protein binding](http://www.ebi.ac.uk/QuickGO/GTerm?id=GO:0005515), [GTP binding](http://www.ebi.ac.uk/QuickGO/GTerm?id=GO:0005525), | | |
| **INTERPRO** | [Elongation factor, GTP-binding domain](https://www.ebi.ac.uk/interpro/entry/InterPro/IPR000795), [Translation elongation factor EFTu/EF1A, C-terminal](https://www.ebi.ac.uk/interpro/entry/InterPro/IPR004160), [Translation elongation factor EFTu/EF1A, domain 2](https://www.ebi.ac.uk/interpro/entry/InterPro/IPR004161), [Translation elongation/initiation factor/Ribosomal, beta-barrel](https://www.ebi.ac.uk/interpro/entry/InterPro/IPR009000), [Translation elongation factor EF1A/initiation factor IF2gamma, C-terminal](https://www.ebi.ac.uk/interpro/entry/InterPro/IPR009001), [Domain of unknown function DUF1916](https://www.ebi.ac.uk/interpro/entry/InterPro/IPR015033), [P-loop containing nucleoside triphosphate hydrolase](https://www.ebi.ac.uk/interpro/entry/InterPro/IPR027417), | | |
| **KEGG_PATHWAY** | [mRNA surveillance pathway](https://david.ncifcrf.gov/kegg.jsp?path=hsa03015$mRNA%20surveillance%20pathway&termId=520047926&source=kegg), [Legionellosis](https://david.ncifcrf.gov/kegg.jsp?path=hsa05134$Legionellosis&termId=520048106&source=kegg), | | |
| **UP_KW_BIOLOGICAL_PROCESS** | [Protein biosynthesis](http://www.uniprot.org/keywords/?query=KW-0648), [Translation regulation](http://www.uniprot.org/keywords/?query=KW-0810), | | |
| **UP_KW_CELLULAR_COMPONENT** | [Membrane](http://www.uniprot.org/keywords/?query=KW-0472), [Cytoplasm](http://www.uniprot.org/keywords/?query=KW-0963), | | |
| **UP_KW_DOMAIN** | [Signal](http://www.uniprot.org/keywords/?query=KW-0732), [Transmembrane](http://www.uniprot.org/keywords/?query=KW-0812), [Transmembrane helix](http://www.uniprot.org/keywords/?query=KW-1133), | | |
| **UP_KW_LIGAND** | [GTP-binding](http://www.uniprot.org/keywords/?query=KW-0342), [Nucleotide-binding](http://www.uniprot.org/keywords/?query=KW-0547), | | |
| **UP_KW_MOLECULAR_FUNCTION** | [Elongation factor](http://www.uniprot.org/keywords/?query=KW-0251), | | |
| **UP_KW_PTM** | [Acetylation](http://www.uniprot.org/keywords/?query=KW-0007), [Phosphoprotein](http://www.uniprot.org/keywords/?query=KW-0597), | | |
| **UP_SEQ_FEATURE** | COMPBIAS:Basic and acidic residues, COMPBIAS:Polar residues, DOMAIN:HBS1_N, DOMAIN:Tr-type G, DOMAIN:tr-type G, NP_BIND:GTP, REGION:Disordered, REGION:G1, REGION:G2, REGION:G3, REGION:G4, REGION:G5, REGION:Interaction with the exosome complex, TRANSMEM:Helical, | | |
| **IQCE** | [**IQ motif containing E(IQCE)**](https://david.ncifcrf.gov/geneReportFull.jsp?rowids=23288) | [**Related Genes**](https://david.ncifcrf.gov/relatedGenes.jsp?id=23288) | [**Homo sapiens**](http://www.ncbi.nlm.nih.gov/Taxonomy/Browser/wwwtax.cgi?name=Homo%20sapiens) |
| **COG_ONTOLOGY** | [Cell division and chromosome partitioning](http://www.ncbi.nlm.nih.gov/COG/new/), | | |
| **GOTERM_BP_DIRECT** | [limb morphogenesis](http://www.ebi.ac.uk/QuickGO/GTerm?id=GO:0035108), | | |
| **GOTERM_CC_DIRECT** | [cilium](http://www.ebi.ac.uk/QuickGO/GTerm?id=GO:0005929), [ciliary membrane](http://www.ebi.ac.uk/QuickGO/GTerm?id=GO:0060170), | | |
| **GOTERM_MF_DIRECT** | [protein binding](http://www.ebi.ac.uk/QuickGO/GTerm?id=GO:0005515), | | |
| **INTERPRO** | [IQ motif, EF-hand binding site](https://www.ebi.ac.uk/interpro/entry/InterPro/IPR000048), [P-loop containing nucleoside triphosphate hydrolase](https://www.ebi.ac.uk/interpro/entry/InterPro/IPR027417), | | |
| **KEGG_PATHWAY** | [Hedgehog signaling pathway](https://david.ncifcrf.gov/kegg.jsp?path=hsa04340$Hedgehog%20signaling%20pathway&termId=520047987&source=kegg), | | |
| **OMIM_DISEASE** | [Polydactyly, postaxial, type A7](http://omim.org/entry/617642), | | |
| **SMART** | [IQ](http://smart.embl.de/smart/do_annotation.pl?DOMAIN=SM00015), | | |
| **UP_KW_CELLULAR_COMPONENT** | [Membrane](http://www.uniprot.org/keywords/?query=KW-0472), [Cell projection](http://www.uniprot.org/keywords/?query=KW-0966), [Cell membrane](http://www.uniprot.org/keywords/?query=KW-1003), | | |
| **UP_KW_DOMAIN** | [Coiled coil](http://www.uniprot.org/keywords/?query=KW-0175), [Repeat](http://www.uniprot.org/keywords/?query=KW-0677), | | |
| **UP_KW_PTM** | [Phosphoprotein](http://www.uniprot.org/keywords/?query=KW-0597), | | |
| **UP_SEQ_FEATURE** | COMPBIAS:Basic and acidic residues, COMPBIAS:Polar residues, DOMAIN:IQ 1, DOMAIN:IQ 2, REGION:Disordered, | | |
| **KIAA0513** | [**KIAA0513(KIAA0513)**](https://david.ncifcrf.gov/geneReportFull.jsp?rowids=9764) | [**Related Genes**](https://david.ncifcrf.gov/relatedGenes.jsp?id=9764) | [**Homo sapiens**](http://www.ncbi.nlm.nih.gov/Taxonomy/Browser/wwwtax.cgi?name=Homo%20sapiens) |
| **GOTERM_CC_DIRECT** | [cytoplasm](http://www.ebi.ac.uk/QuickGO/GTerm?id=GO:0005737), | | |
| **INTERPRO** | [Myotubularin protein](https://www.ebi.ac.uk/interpro/entry/InterPro/IPR022096), | | |
| **UP_KW_CELLULAR_COMPONENT** | [Cytoplasm](http://www.uniprot.org/keywords/?query=KW-0963), | | |
| **UP_KW_PTM** | [Phosphoprotein](http://www.uniprot.org/keywords/?query=KW-0597), | | |
| **UP_SEQ_FEATURE** | COMPBIAS:Basic and acidic residues, COMPBIAS:Polar residues, DOMAIN:SBF2, REGION:Disordered, | | |
| **LPP** | [**LIM domain containing preferred translocation partner in lipoma(LPP)**](https://david.ncifcrf.gov/geneReportFull.jsp?rowids=4026) | [**Related Genes**](https://david.ncifcrf.gov/relatedGenes.jsp?id=4026) | [**Homo sapiens**](http://www.ncbi.nlm.nih.gov/Taxonomy/Browser/wwwtax.cgi?name=Homo%20sapiens) |
| **GOTERM_BP_DIRECT** | [cell-cell adhesion](http://www.ebi.ac.uk/QuickGO/GTerm?id=GO:0098609), | | |
| **GOTERM_CC_DIRECT** | [stress fiber](http://www.ebi.ac.uk/QuickGO/GTerm?id=GO:0001725), [nucleus](http://www.ebi.ac.uk/QuickGO/GTerm?id=GO:0005634), [cytosol](http://www.ebi.ac.uk/QuickGO/GTerm?id=GO:0005829), [plasma membrane](http://www.ebi.ac.uk/QuickGO/GTerm?id=GO:0005886), [focal adhesion](http://www.ebi.ac.uk/QuickGO/GTerm?id=GO:0005925), | | |
| **GOTERM_MF_DIRECT** | [protein binding](http://www.ebi.ac.uk/QuickGO/GTerm?id=GO:0005515), [metal ion binding](http://www.ebi.ac.uk/QuickGO/GTerm?id=GO:0046872), | | |
| **INTERPRO** | [Zinc finger, LIM-type](https://www.ebi.ac.uk/interpro/entry/InterPro/IPR001781), | | |
| **OMIM_DISEASE** | [Leukemia, acute myeloid](http://omim.org/entry/601626), [Lipoma](http://omim.org/entry/Lipoma), | | |
| **SMART** | [LIM](http://smart.embl.de/smart/do_annotation.pl?DOMAIN=SM00132), | | |
| **UP_KW_BIOLOGICAL_PROCESS** | [Cell adhesion](http://www.uniprot.org/keywords/?query=KW-0130), | | |
| **UP_KW_CELLULAR_COMPONENT** | [Membrane](http://www.uniprot.org/keywords/?query=KW-0472), [Nucleus](http://www.uniprot.org/keywords/?query=KW-0539), [Cytoplasm](http://www.uniprot.org/keywords/?query=KW-0963), [Cell junction](http://www.uniprot.org/keywords/?query=KW-0965), [Cell membrane](http://www.uniprot.org/keywords/?query=KW-1003), | | |
| **UP_KW_DOMAIN** | [LIM domain](http://www.uniprot.org/keywords/?query=KW-0440), [Repeat](http://www.uniprot.org/keywords/?query=KW-0677), | | |
| **UP_KW_LIGAND** | [Metal-binding](http://www.uniprot.org/keywords/?query=KW-0479), [Zinc](http://www.uniprot.org/keywords/?query=KW-0862), | | |
| **UP_KW_MOLECULAR_FUNCTION** | [Activator](http://www.uniprot.org/keywords/?query=KW-0010), | | |
| **UP_KW_PTM** | [Acetylation](http://www.uniprot.org/keywords/?query=KW-0007), [Phosphoprotein](http://www.uniprot.org/keywords/?query=KW-0597), [Ubl conjugation](http://www.uniprot.org/keywords/?query=KW-0832), [Isopeptide bond](http://www.uniprot.org/keywords/?query=KW-1017), | | |
| **UP_SEQ_FEATURE** | COMPBIAS:Polar residues, COMPBIAS:Pro residues, CROSSLNK:Glycyl lysine isopeptide (Lys-Gly) (interchain with G-Cter in SUMO1), DOMAIN:LIM zinc-binding, DOMAIN:LIM zinc-binding 1, DOMAIN:LIM zinc-binding 2, DOMAIN:LIM zinc-binding 3, MUTAGEN:L->A: Abolishes binding to SCRIB., MUTAGEN:T->A: Abolishes binding to SCRIB., REGION:Disordered, SITE:Breakpoint for translocation to form HMGA2-LPP, SITE:Breakpoint for translocation to form HMGA2-LPP and KMT2A/MLL1-LPP, | | |
| **LIMS2** | [**LIM zinc finger domain containing 2(LIMS2)**](https://david.ncifcrf.gov/geneReportFull.jsp?rowids=55679) | [**Related Genes**](https://david.ncifcrf.gov/relatedGenes.jsp?id=55679) | [**Homo sapiens**](http://www.ncbi.nlm.nih.gov/Taxonomy/Browser/wwwtax.cgi?name=Homo%20sapiens) |
| **GOTERM_BP_DIRECT** | [cell junction assembly](http://www.ebi.ac.uk/QuickGO/GTerm?id=GO:0034329), [negative regulation of apoptotic process](http://www.ebi.ac.uk/QuickGO/GTerm?id=GO:0043066), [cell-cell junction organization](http://www.ebi.ac.uk/QuickGO/GTerm?id=GO:0045216), [cell-cell adhesion](http://www.ebi.ac.uk/QuickGO/GTerm?id=GO:0098609), [positive regulation of substrate adhesion-dependent cell spreading](http://www.ebi.ac.uk/QuickGO/GTerm?id=GO:1900026), [negative regulation of neural precursor cell proliferation](http://www.ebi.ac.uk/QuickGO/GTerm?id=GO:2000178), [negative regulation of hepatocyte proliferation](http://www.ebi.ac.uk/QuickGO/GTerm?id=GO:2000346), [positive regulation of integrin-mediated signaling pathway](http://www.ebi.ac.uk/QuickGO/GTerm?id=GO:2001046), | | |
| **GOTERM_CC_DIRECT** | [nucleus](http://www.ebi.ac.uk/QuickGO/GTerm?id=GO:0005634), [cytoplasm](http://www.ebi.ac.uk/QuickGO/GTerm?id=GO:0005737), [cytosol](http://www.ebi.ac.uk/QuickGO/GTerm?id=GO:0005829), [plasma membrane](http://www.ebi.ac.uk/QuickGO/GTerm?id=GO:0005886), [cell-cell junction](http://www.ebi.ac.uk/QuickGO/GTerm?id=GO:0005911), [focal adhesion](http://www.ebi.ac.uk/QuickGO/GTerm?id=GO:0005925), | | |
| **GOTERM_MF_DIRECT** | [metal ion binding](http://www.ebi.ac.uk/QuickGO/GTerm?id=GO:0046872), | | |
| **INTERPRO** | [Zinc finger, LIM-type](https://www.ebi.ac.uk/interpro/entry/InterPro/IPR001781), [PINCH](https://www.ebi.ac.uk/interpro/entry/InterPro/IPR017351), | | |
| **OMIM_DISEASE** | [Muscular dystrophy, autosomal recessive, with cardiomyopathy and triangular tongue](http://omim.org/entry/616827), | | |
| **PIR_SUPERFAMILY** | [PINCH protein](http://pir.georgetown.edu/cgi-bin/ipcSF?id=PIRSF038003), | | |
| **SMART** | [LIM](http://smart.embl.de/smart/do_annotation.pl?DOMAIN=SM00132), | | |
| **UP_KW_CELLULAR_COMPONENT** | [Membrane](http://www.uniprot.org/keywords/?query=KW-0472), [Nucleus](http://www.uniprot.org/keywords/?query=KW-0539), [Cell junction](http://www.uniprot.org/keywords/?query=KW-0965), [Cell membrane](http://www.uniprot.org/keywords/?query=KW-1003), | | |
| **UP_KW_DISEASE** | [Disease variant](http://www.uniprot.org/keywords/?query=KW-0225), [Limb-girdle muscular dystrophy](http://www.uniprot.org/keywords/?query=KW-0947), | | |
| **UP_KW_DOMAIN** | [LIM domain](http://www.uniprot.org/keywords/?query=KW-0440), [Repeat](http://www.uniprot.org/keywords/?query=KW-0677), | | |
| **UP_KW_LIGAND** | [Metal-binding](http://www.uniprot.org/keywords/?query=KW-0479), [Zinc](http://www.uniprot.org/keywords/?query=KW-0862), | | |
| **UP_KW_PTM** | [Phosphoprotein](http://www.uniprot.org/keywords/?query=KW-0597), | | |
| **UP_SEQ_FEATURE** | COMPBIAS:Polar residues, DOMAIN:LIM zinc-binding, DOMAIN:LIM zinc-binding 1, DOMAIN:LIM zinc-binding 2, DOMAIN:LIM zinc-binding 3, DOMAIN:LIM zinc-binding 4, DOMAIN:LIM zinc-binding 5, REGION:Disordered, | | |
| **MAPKAP1** | [**MAPK associated protein 1(MAPKAP1)**](https://david.ncifcrf.gov/geneReportFull.jsp?rowids=79109) | [**Related Genes**](https://david.ncifcrf.gov/relatedGenes.jsp?id=79109) | [**Homo sapiens**](http://www.ncbi.nlm.nih.gov/Taxonomy/Browser/wwwtax.cgi?name=Homo%20sapiens) |
| **GOTERM_BP_DIRECT** | [phosphorylation](http://www.ebi.ac.uk/QuickGO/GTerm?id=GO:0016310), [substantia nigra development](http://www.ebi.ac.uk/QuickGO/GTerm?id=GO:0021762), [establishment or maintenance of actin cytoskeleton polarity](http://www.ebi.ac.uk/QuickGO/GTerm?id=GO:0030950), [activation of protein kinase B activity](http://www.ebi.ac.uk/QuickGO/GTerm?id=GO:0032148), [positive regulation of peptidyl-serine phosphorylation](http://www.ebi.ac.uk/QuickGO/GTerm?id=GO:0033138), [TORC2 signaling](http://www.ebi.ac.uk/QuickGO/GTerm?id=GO:0038203), [negative regulation of Ras protein signal transduction](http://www.ebi.ac.uk/QuickGO/GTerm?id=GO:0046580), [regulation of cellular response to oxidative stress](http://www.ebi.ac.uk/QuickGO/GTerm?id=GO:1900407), [regulation of signal transduction by p53 class mediator](http://www.ebi.ac.uk/QuickGO/GTerm?id=GO:1901796), | | |
| **GOTERM_CC_DIRECT** | [nucleoplasm](http://www.ebi.ac.uk/QuickGO/GTerm?id=GO:0005654), [cytoplasm](http://www.ebi.ac.uk/QuickGO/GTerm?id=GO:0005737), [Golgi apparatus](http://www.ebi.ac.uk/QuickGO/GTerm?id=GO:0005794), [cytosol](http://www.ebi.ac.uk/QuickGO/GTerm?id=GO:0005829), [plasma membrane](http://www.ebi.ac.uk/QuickGO/GTerm?id=GO:0005886), [cytoplasmic vesicle](http://www.ebi.ac.uk/QuickGO/GTerm?id=GO:0031410), [TORC2 complex](http://www.ebi.ac.uk/QuickGO/GTerm?id=GO:0031932), | | |
| **GOTERM_MF_DIRECT** | [protein binding](http://www.ebi.ac.uk/QuickGO/GTerm?id=GO:0005515), [phosphatidylinositol-4,5-bisphosphate binding](http://www.ebi.ac.uk/QuickGO/GTerm?id=GO:0005546), [phosphatidylinositol-3,4,5-trisphosphate binding](http://www.ebi.ac.uk/QuickGO/GTerm?id=GO:0005547), [kinase activity](http://www.ebi.ac.uk/QuickGO/GTerm?id=GO:0016301), [protein kinase binding](http://www.ebi.ac.uk/QuickGO/GTerm?id=GO:0019901), [small GTPase binding](http://www.ebi.ac.uk/QuickGO/GTerm?id=GO:0031267), [phosphatidylinositol-3,4-bisphosphate binding](http://www.ebi.ac.uk/QuickGO/GTerm?id=GO:0043325), [phosphatidic acid binding](http://www.ebi.ac.uk/QuickGO/GTerm?id=GO:0070300), [phosphatidylinositol-3,5-bisphosphate binding](http://www.ebi.ac.uk/QuickGO/GTerm?id=GO:0080025), | | |
| **INTERPRO** | [Stress-activated map kinase interacting 1](https://www.ebi.ac.uk/interpro/entry/InterPro/IPR008828), [Pleckstrin homology-like domain](https://www.ebi.ac.uk/interpro/entry/InterPro/IPR011993), | | |
| **KEGG_PATHWAY** | [mTOR signaling pathway](https://david.ncifcrf.gov/kegg.jsp?path=hsa04150$mTOR%20signaling%20pathway&termId=520047972&source=kegg), | | |
| **UP_KW_BIOLOGICAL_PROCESS** | [Stress response](http://www.uniprot.org/keywords/?query=KW-0346), | | |
| **UP_KW_CELLULAR_COMPONENT** | [Membrane](http://www.uniprot.org/keywords/?query=KW-0472), [Nucleus](http://www.uniprot.org/keywords/?query=KW-0539), [Cytoplasmic vesicle](http://www.uniprot.org/keywords/?query=KW-0968), [Cell membrane](http://www.uniprot.org/keywords/?query=KW-1003), | | |
| **UP_KW_DOMAIN** | [Coiled coil](http://www.uniprot.org/keywords/?query=KW-0175), | | |
| **UP_KW_MOLECULAR_FUNCTION** | [Kinase](http://www.uniprot.org/keywords/?query=KW-0418), [Transferase](http://www.uniprot.org/keywords/?query=KW-0808), | | |
| **UP_KW_PTM** | [Acetylation](http://www.uniprot.org/keywords/?query=KW-0007), [Phosphoprotein](http://www.uniprot.org/keywords/?query=KW-0597), | | |
| **UP_SEQ_FEATURE** | DOMAIN:CRIM, DOMAIN:SIN1, DOMAIN:SIN1_PH, REGION:Interaction with ATF2, REGION:Interaction with MAP3K2, REGION:Interaction with NBN, | | |
| **MLXIP** | [**MLX interacting protein(MLXIP)**](https://david.ncifcrf.gov/geneReportFull.jsp?rowids=22877) | [**Related Genes**](https://david.ncifcrf.gov/relatedGenes.jsp?id=22877) | [**Homo sapiens**](http://www.ncbi.nlm.nih.gov/Taxonomy/Browser/wwwtax.cgi?name=Homo%20sapiens) |
| **GOTERM_BP_DIRECT** | [regulation of transcription from RNA polymerase II promoter](http://www.ebi.ac.uk/QuickGO/GTerm?id=GO:0006357), [positive regulation of transcription from RNA polymerase II promoter](http://www.ebi.ac.uk/QuickGO/GTerm?id=GO:0045944), | | |
| **GOTERM_CC_DIRECT** | [chromatin](http://www.ebi.ac.uk/QuickGO/GTerm?id=GO:0000785), [nucleus](http://www.ebi.ac.uk/QuickGO/GTerm?id=GO:0005634), [mitochondrial outer membrane](http://www.ebi.ac.uk/QuickGO/GTerm?id=GO:0005741), | | |
| **GOTERM_MF_DIRECT** | [RNA polymerase II regulatory region sequence-specific DNA binding](http://www.ebi.ac.uk/QuickGO/GTerm?id=GO:0000977), [RNA polymerase II core promoter proximal region sequence-specific DNA binding](http://www.ebi.ac.uk/QuickGO/GTerm?id=GO:0000978), [RNA polymerase II transcription factor activity, sequence-specific DNA binding](http://www.ebi.ac.uk/QuickGO/GTerm?id=GO:0000981), [transcriptional activator activity, RNA polymerase II transcription regulatory region sequence-specific binding](http://www.ebi.ac.uk/QuickGO/GTerm?id=GO:0001228), [protein dimerization activity](http://www.ebi.ac.uk/QuickGO/GTerm?id=GO:0046983), | | |
| **INTERPRO** | [Myc-type, basic helix-loop-helix (bHLH) domain](https://www.ebi.ac.uk/interpro/entry/InterPro/IPR011598), | | |
| **KEGG_PATHWAY** | [Insulin resistance](https://david.ncifcrf.gov/kegg.jsp?path=hsa04931$Insulin%20resistance&termId=520048064&source=kegg), [Non-alcoholic fatty liver disease](https://david.ncifcrf.gov/kegg.jsp?path=hsa04932$Non-alcoholic%20fatty%20liver%20disease&termId=520048065&source=kegg), | | |
| **SMART** | [HLH](http://smart.embl.de/smart/do_annotation.pl?DOMAIN=SM00353), | | |
| **UP_KW_BIOLOGICAL_PROCESS** | [Transcription](http://www.uniprot.org/keywords/?query=KW-0804), [Transcription regulation](http://www.uniprot.org/keywords/?query=KW-0805), | | |
| **UP_KW_CELLULAR_COMPONENT** | [Membrane](http://www.uniprot.org/keywords/?query=KW-0472), [Mitochondrion](http://www.uniprot.org/keywords/?query=KW-0496), [Nucleus](http://www.uniprot.org/keywords/?query=KW-0539), [Cytoplasm](http://www.uniprot.org/keywords/?query=KW-0963), [Mitochondrion outer membrane](http://www.uniprot.org/keywords/?query=KW-1000), | | |
| **UP_KW_DOMAIN** | [Coiled coil](http://www.uniprot.org/keywords/?query=KW-0175), [Signal](http://www.uniprot.org/keywords/?query=KW-0732), | | |
| **UP_KW_MOLECULAR_FUNCTION** | [Activator](http://www.uniprot.org/keywords/?query=KW-0010), [DNA-binding](http://www.uniprot.org/keywords/?query=KW-0238), | | |
| **UP_KW_PTM** | [Acetylation](http://www.uniprot.org/keywords/?query=KW-0007), [Phosphoprotein](http://www.uniprot.org/keywords/?query=KW-0597), | | |
| **UP_SEQ_FEATURE** | COMPBIAS:Polar residues, DOMAIN:BHLH, DOMAIN:bHLH, REGION:Disordered, REGION:Leucine-zipper, REGION:Mediates heterotypic interactions between MLXIP and MLX and is required for cytoplasmic localization, REGION:Required for cytoplasmic localization, REGION:Transactivation domain, | | |
| **MORC3** | [**MORC family CW-type zinc finger 3(MORC3)**](https://david.ncifcrf.gov/geneReportFull.jsp?rowids=23515) | [**Related Genes**](https://david.ncifcrf.gov/relatedGenes.jsp?id=23515) | [**Homo sapiens**](http://www.ncbi.nlm.nih.gov/Taxonomy/Browser/wwwtax.cgi?name=Homo%20sapiens) |
| **GOTERM_BP_DIRECT** | [protein phosphorylation](http://www.ebi.ac.uk/QuickGO/GTerm?id=GO:0006468), [cell aging](http://www.ebi.ac.uk/QuickGO/GTerm?id=GO:0007569), [post-embryonic development](http://www.ebi.ac.uk/QuickGO/GTerm?id=GO:0009791), [viral process](http://www.ebi.ac.uk/QuickGO/GTerm?id=GO:0016032), [peptidyl-serine phosphorylation](http://www.ebi.ac.uk/QuickGO/GTerm?id=GO:0018105), [negative regulation of fibroblast proliferation](http://www.ebi.ac.uk/QuickGO/GTerm?id=GO:0048147), [protein stabilization](http://www.ebi.ac.uk/QuickGO/GTerm?id=GO:0050821), [maintenance of protein location in nucleus](http://www.ebi.ac.uk/QuickGO/GTerm?id=GO:0051457), | | |
| **GOTERM_CC_DIRECT** | [nucleus](http://www.ebi.ac.uk/QuickGO/GTerm?id=GO:0005634), [nucleoplasm](http://www.ebi.ac.uk/QuickGO/GTerm?id=GO:0005654), [nuclear matrix](http://www.ebi.ac.uk/QuickGO/GTerm?id=GO:0016363), [PML body](http://www.ebi.ac.uk/QuickGO/GTerm?id=GO:0016605), | | |
| **GOTERM_MF_DIRECT** | [RNA binding](http://www.ebi.ac.uk/QuickGO/GTerm?id=GO:0003723), [protein binding](http://www.ebi.ac.uk/QuickGO/GTerm?id=GO:0005515), [zinc ion binding](http://www.ebi.ac.uk/QuickGO/GTerm?id=GO:0008270), [methylated histone binding](http://www.ebi.ac.uk/QuickGO/GTerm?id=GO:0035064), | | |
| **INTERPRO** | [Zinc finger, CW-type](https://www.ebi.ac.uk/interpro/entry/InterPro/IPR011124), | | |
| **UP_KW_BIOLOGICAL_PROCESS** | [Host-virus interaction](http://www.uniprot.org/keywords/?query=KW-0945), | | |
| **UP_KW_CELLULAR_COMPONENT** | [Nucleus](http://www.uniprot.org/keywords/?query=KW-0539), | | |
| **UP_KW_DOMAIN** | [Coiled coil](http://www.uniprot.org/keywords/?query=KW-0175), [Zinc-finger](http://www.uniprot.org/keywords/?query=KW-0863), | | |
| **UP_KW_LIGAND** | [Metal-binding](http://www.uniprot.org/keywords/?query=KW-0479), [Zinc](http://www.uniprot.org/keywords/?query=KW-0862), | | |
| **UP_KW_MOLECULAR_FUNCTION** | [RNA-binding](http://www.uniprot.org/keywords/?query=KW-0694), | | |
| **UP_KW_PTM** | [Phosphoprotein](http://www.uniprot.org/keywords/?query=KW-0597), [Ubl conjugation](http://www.uniprot.org/keywords/?query=KW-0832), [Isopeptide bond](http://www.uniprot.org/keywords/?query=KW-1017), | | |
| **UP_SEQ_FEATURE** | COMPBIAS:Polar residues, CROSSLNK:Glycyl lysine isopeptide (Lys-Gly) (interchain with G-Cter in SUMO1); alternate, CROSSLNK:Glycyl lysine isopeptide (Lys-Gly) (interchain with G-Cter in SUMO2), CROSSLNK:Glycyl lysine isopeptide (Lys-Gly) (interchain with G-Cter in SUMO2); alternate, DOMAIN:CW-type, METAL:Zinc, MUTAGEN:D->N: Forms nuclear bodies, but rapidly diffuses throughout the nucleus under conditions of ATP depletion., MUTAGEN:E->A: Fails to localize to PML nuclear bodies and activate TP53., MUTAGEN:G->A: Diffuse nuclear localization. Fails to form nuclear bodies in the presence of ATP., MUTAGEN:K->R: Loss of sumoylation; when associated with R-597; R-650; R-651 and R-740., MUTAGEN:K->R: Loss of sumoylation; when associated with R-597; R-650; R-651 and R-794., MUTAGEN:K->R: Loss of sumoylation; when associated with R-597; R-650; R-740 and R-794., MUTAGEN:K->R: Loss of sumoylation; when associated with R-597; R-651; R-740 and R-794., MUTAGEN:K->R: Loss of sumoylation; when associated with R-650; R-651; R-740 and R-794., MUTAGEN:W->A: Diffuse nuclear localization, possibly due to loss of DNA or nucleosome binding., REGION:Disordered, REGION:Nuclear matrix binding, REGION:RNA binding, ZN_FING:CW-type, | | |
| **NAGK** | [**N-acetylglucosamine kinase(NAGK)**](https://david.ncifcrf.gov/geneReportFull.jsp?rowids=55577) | [**Related Genes**](https://david.ncifcrf.gov/relatedGenes.jsp?id=55577) | [**Homo sapiens**](http://www.ncbi.nlm.nih.gov/Taxonomy/Browser/wwwtax.cgi?name=Homo%20sapiens) |
| **GOTERM_BP_DIRECT** | [N-acetylglucosamine metabolic process](http://www.ebi.ac.uk/QuickGO/GTerm?id=GO:0006044), [UDP-N-acetylglucosamine biosynthetic process](http://www.ebi.ac.uk/QuickGO/GTerm?id=GO:0006048), [N-acetylmannosamine metabolic process](http://www.ebi.ac.uk/QuickGO/GTerm?id=GO:0006051), [N-acetylneuraminate catabolic process](http://www.ebi.ac.uk/QuickGO/GTerm?id=GO:0019262), [carbohydrate phosphorylation](http://www.ebi.ac.uk/QuickGO/GTerm?id=GO:0046835), | | |
| **GOTERM_CC_DIRECT** | [cytosol](http://www.ebi.ac.uk/QuickGO/GTerm?id=GO:0005829), [extracellular exosome](http://www.ebi.ac.uk/QuickGO/GTerm?id=GO:0070062), | | |
| **GOTERM_MF_DIRECT** | [protein binding](http://www.ebi.ac.uk/QuickGO/GTerm?id=GO:0005515), [ATP binding](http://www.ebi.ac.uk/QuickGO/GTerm?id=GO:0005524), [N-acetylglucosamine kinase activity](http://www.ebi.ac.uk/QuickGO/GTerm?id=GO:0045127), | | |
| **INTERPRO** | [ATPase, BadF/BadG/BcrA/BcrD type](https://www.ebi.ac.uk/interpro/entry/InterPro/IPR002731), | | |
| **KEGG_PATHWAY** | [Amino sugar and nucleotide sugar metabolism](https://david.ncifcrf.gov/kegg.jsp?path=hsa00520$Amino%20sugar%20and%20nucleotide%20sugar%20metabolism&termId=520047870&source=kegg), [Metabolic pathways](https://david.ncifcrf.gov/kegg.jsp?path=hsa01100$Metabolic%20pathways&termId=520047911&source=kegg), [Biosynthesis of nucleotide sugars](https://david.ncifcrf.gov/kegg.jsp?path=hsa01250$Biosynthesis%20of%20nucleotide%20sugars&termId=520047917&source=kegg), | | |
| **UP_KW_LIGAND** | [ATP-binding](http://www.uniprot.org/keywords/?query=KW-0067), [Nucleotide-binding](http://www.uniprot.org/keywords/?query=KW-0547), | | |
| **UP_KW_MOLECULAR_FUNCTION** | [Kinase](http://www.uniprot.org/keywords/?query=KW-0418), [Transferase](http://www.uniprot.org/keywords/?query=KW-0808), | | |
| **UP_KW_PTM** | [Acetylation](http://www.uniprot.org/keywords/?query=KW-0007), [Phosphoprotein](http://www.uniprot.org/keywords/?query=KW-0597), | | |
| **UP_SEQ_FEATURE** | BINDING:ATP, BINDING:ATP; via amide nitrogen, BINDING:ATP; via carbonyl oxygen, BINDING:Substrate, DOMAIN:BcrAD_BadFG, REGION:Disordered, REGION:Substrate binding, | | |
| **NLRP3** | [**NLR family pyrin domain containing 3(NLRP3)**](https://david.ncifcrf.gov/geneReportFull.jsp?rowids=114548) | [**Related Genes**](https://david.ncifcrf.gov/relatedGenes.jsp?id=114548) | [**Homo sapiens**](http://www.ncbi.nlm.nih.gov/Taxonomy/Browser/wwwtax.cgi?name=Homo%20sapiens) |
| **GOTERM_BP_DIRECT** | [leukocyte migration involved in inflammatory response](http://www.ebi.ac.uk/QuickGO/GTerm?id=GO:0002523), [negative regulation of acute inflammatory response](http://www.ebi.ac.uk/QuickGO/GTerm?id=GO:0002674), [positive regulation of type 2 immune response](http://www.ebi.ac.uk/QuickGO/GTerm?id=GO:0002830), [apoptotic process](http://www.ebi.ac.uk/QuickGO/GTerm?id=GO:0006915), [defense response](http://www.ebi.ac.uk/QuickGO/GTerm?id=GO:0006952), [inflammatory response](http://www.ebi.ac.uk/QuickGO/GTerm?id=GO:0006954), [signal transduction](http://www.ebi.ac.uk/QuickGO/GTerm?id=GO:0007165), [detection of biotic stimulus](http://www.ebi.ac.uk/QuickGO/GTerm?id=GO:0009595), [response to organic cyclic compound](http://www.ebi.ac.uk/QuickGO/GTerm?id=GO:0014070), [protein deubiquitination](http://www.ebi.ac.uk/QuickGO/GTerm?id=GO:0016579), [negative regulation of NF-kappaB transcription factor activity](http://www.ebi.ac.uk/QuickGO/GTerm?id=GO:0032088), [negative regulation of interleukin-1 beta production](http://www.ebi.ac.uk/QuickGO/GTerm?id=GO:0032691), [positive regulation of interleukin-1 beta production](http://www.ebi.ac.uk/QuickGO/GTerm?id=GO:0032731), [positive regulation of interleukin-4 production](http://www.ebi.ac.uk/QuickGO/GTerm?id=GO:0032753), [purinergic nucleotide receptor signaling pathway](http://www.ebi.ac.uk/QuickGO/GTerm?id=GO:0035590), [positive regulation of cysteine-type endopeptidase activity involved in apoptotic process](http://www.ebi.ac.uk/QuickGO/GTerm?id=GO:0043280), [NLRP3 inflammasome complex assembly](http://www.ebi.ac.uk/QuickGO/GTerm?id=GO:0044546), [innate immune response](http://www.ebi.ac.uk/QuickGO/GTerm?id=GO:0045087), [response to ethanol](http://www.ebi.ac.uk/QuickGO/GTerm?id=GO:0045471), [positive regulation of T-helper 2 cell differentiation](http://www.ebi.ac.uk/QuickGO/GTerm?id=GO:0045630), [positive regulation of transcription from RNA polymerase II promoter](http://www.ebi.ac.uk/QuickGO/GTerm?id=GO:0045944), [negative regulation of inflammatory response](http://www.ebi.ac.uk/QuickGO/GTerm?id=GO:0050728), [positive regulation of NF-kappaB transcription factor activity](http://www.ebi.ac.uk/QuickGO/GTerm?id=GO:0051092), [cellular response to lipopolysaccharide](http://www.ebi.ac.uk/QuickGO/GTerm?id=GO:0071222), [cellular response to virus](http://www.ebi.ac.uk/QuickGO/GTerm?id=GO:0098586), [negative regulation of NIK/NF-kappaB signaling](http://www.ebi.ac.uk/QuickGO/GTerm?id=GO:1901223), [positive regulation of T-helper 2 cell cytokine production](http://www.ebi.ac.uk/QuickGO/GTerm?id=GO:2000553), | | |
| **GOTERM_CC_DIRECT** | [Golgi membrane](http://www.ebi.ac.uk/QuickGO/GTerm?id=GO:0000139), [extracellular region](http://www.ebi.ac.uk/QuickGO/GTerm?id=GO:0005576), [nucleus](http://www.ebi.ac.uk/QuickGO/GTerm?id=GO:0005634), [cytoplasm](http://www.ebi.ac.uk/QuickGO/GTerm?id=GO:0005737), [endoplasmic reticulum](http://www.ebi.ac.uk/QuickGO/GTerm?id=GO:0005783), [cytosol](http://www.ebi.ac.uk/QuickGO/GTerm?id=GO:0005829), [NLRP3 inflammasome complex](http://www.ebi.ac.uk/QuickGO/GTerm?id=GO:0072559), | | |
| **GOTERM_MF_DIRECT** | [protein binding](http://www.ebi.ac.uk/QuickGO/GTerm?id=GO:0005515), [ATP binding](http://www.ebi.ac.uk/QuickGO/GTerm?id=GO:0005524), [transcription factor binding](http://www.ebi.ac.uk/QuickGO/GTerm?id=GO:0008134), [identical protein binding](http://www.ebi.ac.uk/QuickGO/GTerm?id=GO:0042802), [peptidoglycan binding](http://www.ebi.ac.uk/QuickGO/GTerm?id=GO:0042834), [sequence-specific DNA binding](http://www.ebi.ac.uk/QuickGO/GTerm?id=GO:0043565), | | |
| **INTERPRO** | [Leucine-rich repeat](https://www.ebi.ac.uk/interpro/entry/InterPro/IPR001611), [DAPIN domain](https://www.ebi.ac.uk/interpro/entry/InterPro/IPR004020), [NACHT nucleoside triphosphatase](https://www.ebi.ac.uk/interpro/entry/InterPro/IPR007111), [Death-like domain](https://www.ebi.ac.uk/interpro/entry/InterPro/IPR011029), [P-loop containing nucleoside triphosphate hydrolase](https://www.ebi.ac.uk/interpro/entry/InterPro/IPR027417), | | |
| **KEGG_PATHWAY** | [Necroptosis](https://david.ncifcrf.gov/kegg.jsp?path=hsa04217$Necroptosis&termId=520047980&source=kegg), [NOD-like receptor signaling pathway](https://david.ncifcrf.gov/kegg.jsp?path=hsa04621$NOD-like%20receptor%20signaling%20pathway&termId=520048008&source=kegg), [C-type lectin receptor signaling pathway](https://david.ncifcrf.gov/kegg.jsp?path=hsa04625$C-type%20lectin%20receptor%20signaling%20pathway&termId=520048011&source=kegg), [Pathogenic Escherichia coli infection](https://david.ncifcrf.gov/kegg.jsp?path=hsa05130$Pathogenic%20Escherichia%20coli%20infection&termId=520048102&source=kegg), [Shigellosis](https://david.ncifcrf.gov/kegg.jsp?path=hsa05131$Shigellosis&termId=520048103&source=kegg), [Salmonella infection](https://david.ncifcrf.gov/kegg.jsp?path=hsa05132$Salmonella%20infection&termId=520048104&source=kegg), [Pertussis](https://david.ncifcrf.gov/kegg.jsp?path=hsa05133$Pertussis&termId=520048105&source=kegg), [Yersinia infection](https://david.ncifcrf.gov/kegg.jsp?path=hsa05135$Yersinia%20infection&termId=520048107&source=kegg), [Influenza A](https://david.ncifcrf.gov/kegg.jsp?path=hsa05164$Influenza%20A&termId=520048120&source=kegg), [Coronavirus disease - COVID-19](https://david.ncifcrf.gov/kegg.jsp?path=hsa05171$Coronavirus%20disease%20-%20COVID-19&termId=520048127&source=kegg), [Lipid and atherosclerosis](https://david.ncifcrf.gov/kegg.jsp?path=hsa05417$Lipid%20and%20atherosclerosis&termId=520048169&source=kegg), | | |
| **OMIM_DISEASE** | [Familial cold inflammatory syndrome 1](http://omim.org/entry/120100), [Keratoendothelitis fugax hereditaria](http://omim.org/entry/148200), [Muckle-Wells syndrome](http://omim.org/entry/191900), [CINCA syndrome](http://omim.org/entry/607115), [Deafness, autosomal dominant 34, with or without inflammation](http://omim.org/entry/617772), | | |
| **SMART** | [SM01288](http://smart.embl.de/smart/do_annotation.pl?DOMAIN=SM01288), [SM01289](http://smart.embl.de/smart/do_annotation.pl?DOMAIN=SM01289), | | |
| **UP_KW_BIOLOGICAL_PROCESS** | [Immunity](http://www.uniprot.org/keywords/?query=KW-0391), [Inflammatory response](http://www.uniprot.org/keywords/?query=KW-0395), [Innate immunity](http://www.uniprot.org/keywords/?query=KW-0399), [Transcription](http://www.uniprot.org/keywords/?query=KW-0804), [Transcription regulation](http://www.uniprot.org/keywords/?query=KW-0805), | | |
| **UP_KW_CELLULAR_COMPONENT** | [Endoplasmic reticulum](http://www.uniprot.org/keywords/?query=KW-0256), [Golgi apparatus](http://www.uniprot.org/keywords/?query=KW-0333), [Membrane](http://www.uniprot.org/keywords/?query=KW-0472), [Nucleus](http://www.uniprot.org/keywords/?query=KW-0539), [Cytoplasm](http://www.uniprot.org/keywords/?query=KW-0963), [Secreted](http://www.uniprot.org/keywords/?query=KW-0964), [Inflammasome](http://www.uniprot.org/keywords/?query=KW-1271), | | |
| **UP_KW_DISEASE** | [Deafness](http://www.uniprot.org/keywords/?query=KW-0209), [Disease variant](http://www.uniprot.org/keywords/?query=KW-0225), [Amyloidosis](http://www.uniprot.org/keywords/?query=KW-1008), [Non-syndromic deafness](http://www.uniprot.org/keywords/?query=KW-1010), | | |
| **UP_KW_DOMAIN** | [Leucine-rich repeat](http://www.uniprot.org/keywords/?query=KW-0433), [Repeat](http://www.uniprot.org/keywords/?query=KW-0677), | | |
| **UP_KW_LIGAND** | [ATP-binding](http://www.uniprot.org/keywords/?query=KW-0067), [Nucleotide-binding](http://www.uniprot.org/keywords/?query=KW-0547), | | |
| **UP_KW_MOLECULAR_FUNCTION** | [Activator](http://www.uniprot.org/keywords/?query=KW-0010), | | |
| **UP_KW_PTM** | [Ubl conjugation](http://www.uniprot.org/keywords/?query=KW-0832), [Disulfide bond](http://www.uniprot.org/keywords/?query=KW-1015), | | |
| **UP_SEQ_FEATURE** | DISULFID:Redox-active, DOMAIN:NACHT, DOMAIN:NLRC4_HD2, DOMAIN:Pyrin, MUTAGEN:D->R: Complete loss of PYCARD filament nucleation., MUTAGEN:E->R: Complete loss of PYCARD filament nucleation., MUTAGEN:K->E: Complete loss of PYCARD filament nucleation; when associated with E-23., MUTAGEN:K->E: Complete loss of PYCARD filament nucleation; when associated with E-24., MUTAGEN:LK->PA: Loss of PYCARD-binding. No effect on GBP5-binding., MUTAGEN:M->E: Complete loss of PYCARD filament nucleation., MUTAGEN:R->W: Complete loss of PYCARD filament nucleation., NP_BIND:ATP, REPEAT:LRR 1, REPEAT:LRR 2, REPEAT:LRR 3, REPEAT:LRR 4, REPEAT:LRR 5, REPEAT:LRR 6, REPEAT:LRR 7, REPEAT:LRR 8, REPEAT:LRR 9, | | |
| **NSMF** | [**NMDA receptor synaptonuclear signaling and neuronal migration factor(NSMF)**](https://david.ncifcrf.gov/geneReportFull.jsp?rowids=26012) | [**Related Genes**](https://david.ncifcrf.gov/relatedGenes.jsp?id=26012) | [**Homo sapiens**](http://www.ncbi.nlm.nih.gov/Taxonomy/Browser/wwwtax.cgi?name=Homo%20sapiens) |
| **GOTERM_BP_DIRECT** | [positive regulation of protein dephosphorylation](http://www.ebi.ac.uk/QuickGO/GTerm?id=GO:0035307), [regulation of neuron apoptotic process](http://www.ebi.ac.uk/QuickGO/GTerm?id=GO:0043523), [regulation of neuronal synaptic plasticity](http://www.ebi.ac.uk/QuickGO/GTerm?id=GO:0048168), [regulation of dendrite morphogenesis](http://www.ebi.ac.uk/QuickGO/GTerm?id=GO:0048814), [cellular response to amino acid stimulus](http://www.ebi.ac.uk/QuickGO/GTerm?id=GO:0071230), [cellular response to electrical stimulus](http://www.ebi.ac.uk/QuickGO/GTerm?id=GO:0071257), [cellular response to gonadotropin stimulus](http://www.ebi.ac.uk/QuickGO/GTerm?id=GO:0071371), [regulation of neuron migration](http://www.ebi.ac.uk/QuickGO/GTerm?id=GO:2001222), [positive regulation of neuron migration](http://www.ebi.ac.uk/QuickGO/GTerm?id=GO:2001224), | | |
| **GOTERM_CC_DIRECT** | [euchromatin](http://www.ebi.ac.uk/QuickGO/GTerm?id=GO:0000791), [nucleus](http://www.ebi.ac.uk/QuickGO/GTerm?id=GO:0005634), [nuclear envelope](http://www.ebi.ac.uk/QuickGO/GTerm?id=GO:0005635), [nucleoplasm](http://www.ebi.ac.uk/QuickGO/GTerm?id=GO:0005654), [cytoplasm](http://www.ebi.ac.uk/QuickGO/GTerm?id=GO:0005737), [plasma membrane](http://www.ebi.ac.uk/QuickGO/GTerm?id=GO:0005886), [postsynaptic density](http://www.ebi.ac.uk/QuickGO/GTerm?id=GO:0014069), [membrane](http://www.ebi.ac.uk/QuickGO/GTerm?id=GO:0016020), [nuclear matrix](http://www.ebi.ac.uk/QuickGO/GTerm?id=GO:0016363), [dendrite](http://www.ebi.ac.uk/QuickGO/GTerm?id=GO:0030425), [cortical cytoskeleton](http://www.ebi.ac.uk/QuickGO/GTerm?id=GO:0030863), [nuclear membrane](http://www.ebi.ac.uk/QuickGO/GTerm?id=GO:0031965), [neuron projection](http://www.ebi.ac.uk/QuickGO/GTerm?id=GO:0043005), [perikaryon](http://www.ebi.ac.uk/QuickGO/GTerm?id=GO:0043204), [synapse](http://www.ebi.ac.uk/QuickGO/GTerm?id=GO:0045202), [apical dendrite](http://www.ebi.ac.uk/QuickGO/GTerm?id=GO:0097440), | | |
| **GOTERM_MF_DIRECT** | [calcium-dependent protein binding](http://www.ebi.ac.uk/QuickGO/GTerm?id=GO:0048306), | | |
| **OMIM_DISEASE** | [Hypogonadotropic hypogonadism 9 with or without anosmia](http://omim.org/entry/614838), | | |
| **UP_KW_CELLULAR_COMPONENT** | [Cytoskeleton](http://www.uniprot.org/keywords/?query=KW-0206), [Membrane](http://www.uniprot.org/keywords/?query=KW-0472), [Nucleus](http://www.uniprot.org/keywords/?query=KW-0539), [Synapse](http://www.uniprot.org/keywords/?query=KW-0770), [Synaptosome](http://www.uniprot.org/keywords/?query=KW-0771), [Cytoplasm](http://www.uniprot.org/keywords/?query=KW-0963), [Cell junction](http://www.uniprot.org/keywords/?query=KW-0965), [Cell projection](http://www.uniprot.org/keywords/?query=KW-0966), [Cell membrane](http://www.uniprot.org/keywords/?query=KW-1003), | | |
| **UP_KW_DISEASE** | [Disease variant](http://www.uniprot.org/keywords/?query=KW-0225), [Kallmann syndrome](http://www.uniprot.org/keywords/?query=KW-0956), [Hypogonadotropic hypogonadism](http://www.uniprot.org/keywords/?query=KW-1016), | | |
| **UP_KW_PTM** | [Lipoprotein](http://www.uniprot.org/keywords/?query=KW-0449), [Myristate](http://www.uniprot.org/keywords/?query=KW-0519), [Phosphoprotein](http://www.uniprot.org/keywords/?query=KW-0597), | | |
| **UP_SEQ_FEATURE** | LIPID:N-myristoyl glycine, MOTIF:Nuclear localization signal, MUTAGEN:RK->AA: Localizes both in the cytoplasm and the nucleus., MUTAGEN:RRKR->AAKA: Localizes predominantly in the cytoplasm., REGION:Disordered, REGION:Necessary and sufficient to elicit dendritic processes and synaptic contacts, | | |
| **NSMCE2** | [**NSE2 (MMS21) homolog, SMC5-SMC6 complex SUMO ligase(NSMCE2)**](https://david.ncifcrf.gov/geneReportFull.jsp?rowids=286053) | [**Related Genes**](https://david.ncifcrf.gov/relatedGenes.jsp?id=286053) | [**Homo sapiens**](http://www.ncbi.nlm.nih.gov/Taxonomy/Browser/wwwtax.cgi?name=Homo%20sapiens) |
| **COG_ONTOLOGY** | [DNA replication, recombination, and repair](http://www.ncbi.nlm.nih.gov/COG/new/), | | |
| **GOTERM_BP_DIRECT** | [telomere maintenance via recombination](http://www.ebi.ac.uk/QuickGO/GTerm?id=GO:0000722), [double-strand break repair via homologous recombination](http://www.ebi.ac.uk/QuickGO/GTerm?id=GO:0000724), [cell cycle](http://www.ebi.ac.uk/QuickGO/GTerm?id=GO:0007049), [protein sumoylation](http://www.ebi.ac.uk/QuickGO/GTerm?id=GO:0016925), [positive regulation of maintenance of mitotic sister chromatid cohesion](http://www.ebi.ac.uk/QuickGO/GTerm?id=GO:0034184), [positive regulation of mitotic metaphase/anaphase transition](http://www.ebi.ac.uk/QuickGO/GTerm?id=GO:0045842), [cell division](http://www.ebi.ac.uk/QuickGO/GTerm?id=GO:0051301), [cellular senescence](http://www.ebi.ac.uk/QuickGO/GTerm?id=GO:0090398), | | |
| **GOTERM_CC_DIRECT** | [chromosome, telomeric region](http://www.ebi.ac.uk/QuickGO/GTerm?id=GO:0000781), [nucleus](http://www.ebi.ac.uk/QuickGO/GTerm?id=GO:0005634), [nucleoplasm](http://www.ebi.ac.uk/QuickGO/GTerm?id=GO:0005654), [nuclear body](http://www.ebi.ac.uk/QuickGO/GTerm?id=GO:0016604), [PML body](http://www.ebi.ac.uk/QuickGO/GTerm?id=GO:0016605), [Smc5-Smc6 complex](http://www.ebi.ac.uk/QuickGO/GTerm?id=GO:0030915), | | |
| **GOTERM_MF_DIRECT** | [protein binding](http://www.ebi.ac.uk/QuickGO/GTerm?id=GO:0005515), [zinc ion binding](http://www.ebi.ac.uk/QuickGO/GTerm?id=GO:0008270), [SUMO transferase activity](http://www.ebi.ac.uk/QuickGO/GTerm?id=GO:0019789), [SUMO ligase activity](http://www.ebi.ac.uk/QuickGO/GTerm?id=GO:0061665), | | |
| **INTERPRO** | [Zinc finger, MIZ-type](https://www.ebi.ac.uk/interpro/entry/InterPro/IPR004181), [Zinc finger, RING/FYVE/PHD-type](https://www.ebi.ac.uk/interpro/entry/InterPro/IPR013083), [E3 SUMO-protein ligase Nse2 (Mms21)](https://www.ebi.ac.uk/interpro/entry/InterPro/IPR026846), | | |
| **OMIM_DISEASE** | [Seckel syndrome 10](http://omim.org/entry/617253), | | |
| **UP_KW_BIOLOGICAL_PROCESS** | [Cell cycle](http://www.uniprot.org/keywords/?query=KW-0131), [Cell division](http://www.uniprot.org/keywords/?query=KW-0132), [DNA damage](http://www.uniprot.org/keywords/?query=KW-0227), [DNA recombination](http://www.uniprot.org/keywords/?query=KW-0233), [DNA repair](http://www.uniprot.org/keywords/?query=KW-0234), [Mitosis](http://www.uniprot.org/keywords/?query=KW-0498), [Ubl conjugation pathway](http://www.uniprot.org/keywords/?query=KW-0833), | | |
| **UP_KW_CELLULAR_COMPONENT** | [Chromosome](http://www.uniprot.org/keywords/?query=KW-0158), [Nucleus](http://www.uniprot.org/keywords/?query=KW-0539), [Telomere](http://www.uniprot.org/keywords/?query=KW-0779), | | |
| **UP_KW_DISEASE** | [Dwarfism](http://www.uniprot.org/keywords/?query=KW-0242), [Mental retardation](http://www.uniprot.org/keywords/?query=KW-0991), | | |
| **UP_KW_DOMAIN** | [Zinc-finger](http://www.uniprot.org/keywords/?query=KW-0863), | | |
| **UP_KW_LIGAND** | [Metal-binding](http://www.uniprot.org/keywords/?query=KW-0479), [Zinc](http://www.uniprot.org/keywords/?query=KW-0862), | | |
| **UP_KW_MOLECULAR_FUNCTION** | [Transferase](http://www.uniprot.org/keywords/?query=KW-0808), | | |
| **UP_KW_PTM** | [Acetylation](http://www.uniprot.org/keywords/?query=KW-0007), [Phosphoprotein](http://www.uniprot.org/keywords/?query=KW-0597), [Ubl conjugation](http://www.uniprot.org/keywords/?query=KW-0832), [Isopeptide bond](http://www.uniprot.org/keywords/?query=KW-1017), | | |
| **UP_SEQ_FEATURE** | CROSSLNK:Glycyl lysine isopeptide (Lys-Gly) (interchain with G-Cter in SUMO2), DOMAIN:SP-RING-type, METAL:Zinc, MUTAGEN:C->A: Induces a strong decrease in SUMO ligase activity., MUTAGEN:H->A: Induces a strong decrease in SUMO ligase activity., REGION:Disordered, ZN_FING:SP-RING-type, | | |
| **PHRF1** | [**PHD and ring finger domains 1(PHRF1)**](https://david.ncifcrf.gov/geneReportFull.jsp?rowids=57661) | [**Related Genes**](https://david.ncifcrf.gov/relatedGenes.jsp?id=57661) | [**Homo sapiens**](http://www.ncbi.nlm.nih.gov/Taxonomy/Browser/wwwtax.cgi?name=Homo%20sapiens) |
| **GOTERM_BP_DIRECT** | [transcription from RNA polymerase II promoter](http://www.ebi.ac.uk/QuickGO/GTerm?id=GO:0006366), [mRNA processing](http://www.ebi.ac.uk/QuickGO/GTerm?id=GO:0006397), | | |
| **GOTERM_CC_DIRECT** | [membrane](http://www.ebi.ac.uk/QuickGO/GTerm?id=GO:0016020), | | |
| **GOTERM_MF_DIRECT** | [protein domain specific binding](http://www.ebi.ac.uk/QuickGO/GTerm?id=GO:0019904), [metal ion binding](http://www.ebi.ac.uk/QuickGO/GTerm?id=GO:0046872), [RNA polymerase binding](http://www.ebi.ac.uk/QuickGO/GTerm?id=GO:0070063), | | |
| **INTERPRO** | [Zinc finger, RING-type](https://www.ebi.ac.uk/interpro/entry/InterPro/IPR001841), [Zinc finger, PHD-type](https://www.ebi.ac.uk/interpro/entry/InterPro/IPR001965), [Zinc finger, FYVE/PHD-type](https://www.ebi.ac.uk/interpro/entry/InterPro/IPR011011), [Zinc finger, RING/FYVE/PHD-type](https://www.ebi.ac.uk/interpro/entry/InterPro/IPR013083), [Zinc finger, RING-type, conserved site](https://www.ebi.ac.uk/interpro/entry/InterPro/IPR017907), [Zinc finger, PHD-type, conserved site](https://www.ebi.ac.uk/interpro/entry/InterPro/IPR019786), [Zinc finger, PHD-finger](https://www.ebi.ac.uk/interpro/entry/InterPro/IPR019787), | | |
| **SMART** | [RING](http://smart.embl.de/smart/do_annotation.pl?DOMAIN=SM00184), [PHD](http://smart.embl.de/smart/do_annotation.pl?DOMAIN=SM00249), | | |
| **UP_KW_DOMAIN** | [Coiled coil](http://www.uniprot.org/keywords/?query=KW-0175), [Zinc-finger](http://www.uniprot.org/keywords/?query=KW-0863), | | |
| **UP_KW_LIGAND** | [Metal-binding](http://www.uniprot.org/keywords/?query=KW-0479), [Zinc](http://www.uniprot.org/keywords/?query=KW-0862), | | |
| **UP_KW_PTM** | [Phosphoprotein](http://www.uniprot.org/keywords/?query=KW-0597), | | |
| **UP_SEQ_FEATURE** | COMPBIAS:Acidic residues, COMPBIAS:Basic and acidic residues, COMPBIAS:Basic residues, COMPBIAS:Polar residues, DOMAIN:PHD-type, DOMAIN:RING-type, REGION:Disordered, ZN_FING:PHD-type, ZN_FING:RING-type; degenerate, | | |
| **RAD51B** | [**RAD51 paralog B(RAD51B)**](https://david.ncifcrf.gov/geneReportFull.jsp?rowids=5890) | [**Related Genes**](https://david.ncifcrf.gov/relatedGenes.jsp?id=5890) | [**Homo sapiens**](http://www.ncbi.nlm.nih.gov/Taxonomy/Browser/wwwtax.cgi?name=Homo%20sapiens) |
| **GOTERM_BP_DIRECT** | [double-strand break repair via homologous recombination](http://www.ebi.ac.uk/QuickGO/GTerm?id=GO:0000724), [blastocyst growth](http://www.ebi.ac.uk/QuickGO/GTerm?id=GO:0001832), [DNA repair](http://www.ebi.ac.uk/QuickGO/GTerm?id=GO:0006281), [DNA recombination](http://www.ebi.ac.uk/QuickGO/GTerm?id=GO:0006310), [reciprocal meiotic recombination](http://www.ebi.ac.uk/QuickGO/GTerm?id=GO:0007131), [blood coagulation](http://www.ebi.ac.uk/QuickGO/GTerm?id=GO:0007596), [positive regulation of cell proliferation](http://www.ebi.ac.uk/QuickGO/GTerm?id=GO:0008284), [positive regulation of G2/M transition of mitotic cell cycle](http://www.ebi.ac.uk/QuickGO/GTerm?id=GO:0010971), [somite development](http://www.ebi.ac.uk/QuickGO/GTerm?id=GO:0061053), | | |
| **GOTERM_CC_DIRECT** | [nucleus](http://www.ebi.ac.uk/QuickGO/GTerm?id=GO:0005634), [nucleoplasm](http://www.ebi.ac.uk/QuickGO/GTerm?id=GO:0005654), [replication fork](http://www.ebi.ac.uk/QuickGO/GTerm?id=GO:0005657), [integral component of membrane](http://www.ebi.ac.uk/QuickGO/GTerm?id=GO:0016021), [Rad51B-Rad51C-Rad51D-XRCC2 complex](http://www.ebi.ac.uk/QuickGO/GTerm?id=GO:0033063), | | |
| **GOTERM_MF_DIRECT** | [four-way junction DNA binding](http://www.ebi.ac.uk/QuickGO/GTerm?id=GO:0000400), [DNA binding](http://www.ebi.ac.uk/QuickGO/GTerm?id=GO:0003677), [double-stranded DNA binding](http://www.ebi.ac.uk/QuickGO/GTerm?id=GO:0003690), [single-stranded DNA binding](http://www.ebi.ac.uk/QuickGO/GTerm?id=GO:0003697), [protein binding](http://www.ebi.ac.uk/QuickGO/GTerm?id=GO:0005515), [ATP binding](http://www.ebi.ac.uk/QuickGO/GTerm?id=GO:0005524), [DNA-dependent ATPase activity](http://www.ebi.ac.uk/QuickGO/GTerm?id=GO:0008094), | | |
| **INTERPRO** | [AAA+ ATPase domain](https://www.ebi.ac.uk/interpro/entry/InterPro/IPR003593), [DNA recombination and repair protein Rad51, C-terminal](https://www.ebi.ac.uk/interpro/entry/InterPro/IPR013632), [DNA recombination and repair protein, RecA-like](https://www.ebi.ac.uk/interpro/entry/InterPro/IPR016467), [DNA recombination/repair protein RecA/RadB, ATP-binding domain](https://www.ebi.ac.uk/interpro/entry/InterPro/IPR020588), [P-loop containing nucleoside triphosphate hydrolase](https://www.ebi.ac.uk/interpro/entry/InterPro/IPR027417), | | |
| **KEGG_PATHWAY** | [Homologous recombination](https://david.ncifcrf.gov/kegg.jsp?path=hsa03440$Homologous%20recombination&termId=520047938&source=kegg), | | |
| **PIR_SUPERFAMILY** | [DNA repair and recombination protein, Rad51 type](http://pir.georgetown.edu/cgi-bin/ipcSF?id=PIRSF005856), | | |
| **SMART** | [AAA](http://smart.embl.de/smart/do_annotation.pl?DOMAIN=SM00382), | | |
| **UP_KW_BIOLOGICAL_PROCESS** | [DNA damage](http://www.uniprot.org/keywords/?query=KW-0227), [DNA recombination](http://www.uniprot.org/keywords/?query=KW-0233), [DNA repair](http://www.uniprot.org/keywords/?query=KW-0234), | | |
| **UP_KW_CELLULAR_COMPONENT** | [Membrane](http://www.uniprot.org/keywords/?query=KW-0472), [Nucleus](http://www.uniprot.org/keywords/?query=KW-0539), | | |
| **UP_KW_DOMAIN** | [Transmembrane](http://www.uniprot.org/keywords/?query=KW-0812), [Transmembrane helix](http://www.uniprot.org/keywords/?query=KW-1133), | | |
| **UP_KW_LIGAND** | [ATP-binding](http://www.uniprot.org/keywords/?query=KW-0067), [Nucleotide-binding](http://www.uniprot.org/keywords/?query=KW-0547), | | |
| **UP_KW_MOLECULAR_FUNCTION** | [DNA-binding](http://www.uniprot.org/keywords/?query=KW-0238), | | |
| **UP_KW_PTM** | [Phosphoprotein](http://www.uniprot.org/keywords/?query=KW-0597), | | |
| **UP_SEQ_FEATURE** | DOMAIN:RECA_2, MUTAGEN:P->L: Abolishes interaction with BCR-ABL SH3 domain., NP_BIND:ATP, REGION:Interaction with RAD51C, SITE:Breakpoint for translocation to form HMGA2-RAD51B, TRANSMEM:Helical, | | |
| **RBFOX3** | [**RNA binding fox-1 homolog 3(RBFOX3)**](https://david.ncifcrf.gov/geneReportFull.jsp?rowids=146713) | [**Related Genes**](https://david.ncifcrf.gov/relatedGenes.jsp?id=146713) | [**Homo sapiens**](http://www.ncbi.nlm.nih.gov/Taxonomy/Browser/wwwtax.cgi?name=Homo%20sapiens) |
| **COG_ONTOLOGY** | [General function prediction only](http://www.ncbi.nlm.nih.gov/COG/new/), | | |
| **GOTERM_BP_DIRECT** | [regulation of alternative mRNA splicing, via spliceosome](http://www.ebi.ac.uk/QuickGO/GTerm?id=GO:0000381), [mRNA processing](http://www.ebi.ac.uk/QuickGO/GTerm?id=GO:0006397), [nervous system development](http://www.ebi.ac.uk/QuickGO/GTerm?id=GO:0007399), [RNA splicing](http://www.ebi.ac.uk/QuickGO/GTerm?id=GO:0008380), [regulation of RNA splicing](http://www.ebi.ac.uk/QuickGO/GTerm?id=GO:0043484), | | |
| **GOTERM_CC_DIRECT** | [nucleus](http://www.ebi.ac.uk/QuickGO/GTerm?id=GO:0005634), [cytoplasm](http://www.ebi.ac.uk/QuickGO/GTerm?id=GO:0005737), [perikaryon](http://www.ebi.ac.uk/QuickGO/GTerm?id=GO:0043204), | | |
| **GOTERM_MF_DIRECT** | [DNA binding](http://www.ebi.ac.uk/QuickGO/GTerm?id=GO:0003677), [RNA binding](http://www.ebi.ac.uk/QuickGO/GTerm?id=GO:0003723), [mRNA binding](http://www.ebi.ac.uk/QuickGO/GTerm?id=GO:0003729), | | |
| **INTERPRO** | [RNA recognition motif domain](https://www.ebi.ac.uk/interpro/entry/InterPro/IPR000504), [Nucleotide-binding, alpha-beta plait](https://www.ebi.ac.uk/interpro/entry/InterPro/IPR012677), [RNA binding protein Fox-1](https://www.ebi.ac.uk/interpro/entry/InterPro/IPR017325), [Fox-1 C-terminal domain](https://www.ebi.ac.uk/interpro/entry/InterPro/IPR025670), | | |
| **PIR_SUPERFAMILY** | [ataxin 2 binding protein, A2BP type](http://pir.georgetown.edu/cgi-bin/ipcSF?id=PIRSF037932), | | |
| **SMART** | [RRM](http://smart.embl.de/smart/do_annotation.pl?DOMAIN=SM00360), | | |
| **UP_KW_BIOLOGICAL_PROCESS** | [mRNA processing](http://www.uniprot.org/keywords/?query=KW-0507), [mRNA splicing](http://www.uniprot.org/keywords/?query=KW-0508), | | |
| **UP_KW_CELLULAR_COMPONENT** | [Nucleus](http://www.uniprot.org/keywords/?query=KW-0539), [Cytoplasm](http://www.uniprot.org/keywords/?query=KW-0963), | | |
| **UP_KW_MOLECULAR_FUNCTION** | [RNA-binding](http://www.uniprot.org/keywords/?query=KW-0694), | | |
| **UP_KW_PTM** | [Methylation](http://www.uniprot.org/keywords/?query=KW-0488), | | |
| **UP_SEQ_FEATURE** | COMPBIAS:Polar residues, COMPBIAS:Pro residues, DOMAIN:Fox-1_C, DOMAIN:RRM, REGION:Disordered, SITE:Interaction with RNA, | | |
| **RUFY4** | [**RUN and FYVE domain containing 4(RUFY4)**](https://david.ncifcrf.gov/geneReportFull.jsp?rowids=285180) | [**Related Genes**](https://david.ncifcrf.gov/relatedGenes.jsp?id=285180) | [**Homo sapiens**](http://www.ncbi.nlm.nih.gov/Taxonomy/Browser/wwwtax.cgi?name=Homo%20sapiens) |
| **GOTERM_BP_DIRECT** | [autophagosome assembly](http://www.ebi.ac.uk/QuickGO/GTerm?id=GO:0000045), [positive regulation of macroautophagy](http://www.ebi.ac.uk/QuickGO/GTerm?id=GO:0016239), [cellular response to interleukin-4](http://www.ebi.ac.uk/QuickGO/GTerm?id=GO:0071353), | | |
| **GOTERM_CC_DIRECT** | [autophagosome](http://www.ebi.ac.uk/QuickGO/GTerm?id=GO:0005776), [cytoplasmic vesicle](http://www.ebi.ac.uk/QuickGO/GTerm?id=GO:0031410), | | |
| **GOTERM_MF_DIRECT** | [protein binding](http://www.ebi.ac.uk/QuickGO/GTerm?id=GO:0005515), [phosphatidylinositol-3-phosphate binding](http://www.ebi.ac.uk/QuickGO/GTerm?id=GO:0032266), [metal ion binding](http://www.ebi.ac.uk/QuickGO/GTerm?id=GO:0046872), | | |
| **INTERPRO** | [RUN](https://www.ebi.ac.uk/interpro/entry/InterPro/IPR004012), [Zinc finger, FYVE/PHD-type](https://www.ebi.ac.uk/interpro/entry/InterPro/IPR011011), [Zinc finger, FYVE-related](https://www.ebi.ac.uk/interpro/entry/InterPro/IPR017455), | | |
| **SMART** | [RUN](http://smart.embl.de/smart/do_annotation.pl?DOMAIN=SM00593), | | |
| **UP_KW_BIOLOGICAL_PROCESS** | [Autophagy](http://www.uniprot.org/keywords/?query=KW-0072), | | |
| **UP_KW_CELLULAR_COMPONENT** | [Cytoplasmic vesicle](http://www.uniprot.org/keywords/?query=KW-0968), | | |
| **UP_KW_DOMAIN** | [Coiled coil](http://www.uniprot.org/keywords/?query=KW-0175), [Zinc-finger](http://www.uniprot.org/keywords/?query=KW-0863), | | |
| **UP_KW_LIGAND** | [Metal-binding](http://www.uniprot.org/keywords/?query=KW-0479), [Zinc](http://www.uniprot.org/keywords/?query=KW-0862), | | |
| **UP_SEQ_FEATURE** | DOMAIN:RUN, METAL:Zinc 1, METAL:Zinc 2, REGION:Disordered, ZN_FING:FYVE-type, | | |
| **RUNX2** | [**RUNX family transcription factor 2(RUNX2)**](https://david.ncifcrf.gov/geneReportFull.jsp?rowids=860) | [**Related Genes**](https://david.ncifcrf.gov/relatedGenes.jsp?id=860) | [**Homo sapiens**](http://www.ncbi.nlm.nih.gov/Taxonomy/Browser/wwwtax.cgi?name=Homo%20sapiens) |
| **GOTERM_BP_DIRECT** | [ossification](http://www.ebi.ac.uk/QuickGO/GTerm?id=GO:0001503), [osteoblast differentiation](http://www.ebi.ac.uk/QuickGO/GTerm?id=GO:0001649), [endochondral ossification](http://www.ebi.ac.uk/QuickGO/GTerm?id=GO:0001958), [osteoblast fate commitment](http://www.ebi.ac.uk/QuickGO/GTerm?id=GO:0002051), [chondrocyte differentiation](http://www.ebi.ac.uk/QuickGO/GTerm?id=GO:0002062), [chondrocyte development](http://www.ebi.ac.uk/QuickGO/GTerm?id=GO:0002063), [osteoblast development](http://www.ebi.ac.uk/QuickGO/GTerm?id=GO:0002076), [regulation of transcription, DNA-templated](http://www.ebi.ac.uk/QuickGO/GTerm?id=GO:0006355), [regulation of transcription from RNA polymerase II promoter](http://www.ebi.ac.uk/QuickGO/GTerm?id=GO:0006357), [positive regulation of cell proliferation](http://www.ebi.ac.uk/QuickGO/GTerm?id=GO:0008284), [positive regulation of gene expression](http://www.ebi.ac.uk/QuickGO/GTerm?id=GO:0010628), [hemopoiesis](http://www.ebi.ac.uk/QuickGO/GTerm?id=GO:0030097), [neuron differentiation](http://www.ebi.ac.uk/QuickGO/GTerm?id=GO:0030182), [T cell differentiation](http://www.ebi.ac.uk/QuickGO/GTerm?id=GO:0030217), [regulation of ossification](http://www.ebi.ac.uk/QuickGO/GTerm?id=GO:0030278), [BMP signaling pathway](http://www.ebi.ac.uk/QuickGO/GTerm?id=GO:0030509), [positive regulation of chondrocyte differentiation](http://www.ebi.ac.uk/QuickGO/GTerm?id=GO:0032332), [embryonic forelimb morphogenesis](http://www.ebi.ac.uk/QuickGO/GTerm?id=GO:0035115), [regulation of fibroblast growth factor receptor signaling pathway](http://www.ebi.ac.uk/QuickGO/GTerm?id=GO:0040036), [odontogenesis of dentin-containing tooth](http://www.ebi.ac.uk/QuickGO/GTerm?id=GO:0042475), [regulation of odontogenesis of dentin-containing tooth](http://www.ebi.ac.uk/QuickGO/GTerm?id=GO:0042487), [regulation of cell differentiation](http://www.ebi.ac.uk/QuickGO/GTerm?id=GO:0045595), [positive regulation of osteoblast differentiation](http://www.ebi.ac.uk/QuickGO/GTerm?id=GO:0045669), [negative regulation of smoothened signaling pathway](http://www.ebi.ac.uk/QuickGO/GTerm?id=GO:0045879), [negative regulation of transcription, DNA-templated](http://www.ebi.ac.uk/QuickGO/GTerm?id=GO:0045892), [positive regulation of transcription, DNA-templated](http://www.ebi.ac.uk/QuickGO/GTerm?id=GO:0045893), [positive regulation of transcription from RNA polymerase II promoter](http://www.ebi.ac.uk/QuickGO/GTerm?id=GO:0045944), [cell maturation](http://www.ebi.ac.uk/QuickGO/GTerm?id=GO:0048469), [embryonic cranial skeleton morphogenesis](http://www.ebi.ac.uk/QuickGO/GTerm?id=GO:0048701), [stem cell differentiation](http://www.ebi.ac.uk/QuickGO/GTerm?id=GO:0048863), [regulation of transcription initiation from RNA polymerase II promoter](http://www.ebi.ac.uk/QuickGO/GTerm?id=GO:0060260), [cellular response to BMP stimulus](http://www.ebi.ac.uk/QuickGO/GTerm?id=GO:0071773), [positive regulation of transcription from RNA polymerase II promoter involved in cellular response to chemical stimulus](http://www.ebi.ac.uk/QuickGO/GTerm?id=GO:1901522), | | |
| **GOTERM_CC_DIRECT** | [chromatin](http://www.ebi.ac.uk/QuickGO/GTerm?id=GO:0000785), [nucleus](http://www.ebi.ac.uk/QuickGO/GTerm?id=GO:0005634), [nucleoplasm](http://www.ebi.ac.uk/QuickGO/GTerm?id=GO:0005654), [transcription factor complex](http://www.ebi.ac.uk/QuickGO/GTerm?id=GO:0005667), [cytoplasm](http://www.ebi.ac.uk/QuickGO/GTerm?id=GO:0005737), [cytosol](http://www.ebi.ac.uk/QuickGO/GTerm?id=GO:0005829), | | |
| **GOTERM_MF_DIRECT** | [RNA polymerase II core promoter proximal region sequence-specific DNA binding](http://www.ebi.ac.uk/QuickGO/GTerm?id=GO:0000978), [RNA polymerase II transcription factor activity, sequence-specific DNA binding](http://www.ebi.ac.uk/QuickGO/GTerm?id=GO:0000981), [transcriptional activator activity, RNA polymerase II transcription regulatory region sequence-specific binding](http://www.ebi.ac.uk/QuickGO/GTerm?id=GO:0001228), [DNA binding](http://www.ebi.ac.uk/QuickGO/GTerm?id=GO:0003677), [chromatin binding](http://www.ebi.ac.uk/QuickGO/GTerm?id=GO:0003682), [transcription factor activity, sequence-specific DNA binding](http://www.ebi.ac.uk/QuickGO/GTerm?id=GO:0003700), [protein binding](http://www.ebi.ac.uk/QuickGO/GTerm?id=GO:0005515), [ATP binding](http://www.ebi.ac.uk/QuickGO/GTerm?id=GO:0005524), [protein domain specific binding](http://www.ebi.ac.uk/QuickGO/GTerm?id=GO:0019904), [bHLH transcription factor binding](http://www.ebi.ac.uk/QuickGO/GTerm?id=GO:0043425), [repressing transcription factor binding](http://www.ebi.ac.uk/QuickGO/GTerm?id=GO:0070491), [sequence-specific double-stranded DNA binding](http://www.ebi.ac.uk/QuickGO/GTerm?id=GO:1990837), | | |
| **INTERPRO** | [Acute myeloid leukemia 1 protein (AML1)/Runt](https://www.ebi.ac.uk/interpro/entry/InterPro/IPR000040), [p53-like transcription factor, DNA-binding](https://www.ebi.ac.uk/interpro/entry/InterPro/IPR008967), [p53/RUNT-type transcription factor, DNA-binding domain](https://www.ebi.ac.uk/interpro/entry/InterPro/IPR012346), [Runt domain](https://www.ebi.ac.uk/interpro/entry/InterPro/IPR013524), [Runx, C-terminal domain](https://www.ebi.ac.uk/interpro/entry/InterPro/IPR013711), [Runt-related transcription factor RUNX](https://www.ebi.ac.uk/interpro/entry/InterPro/IPR016554), [Runx, central domain](https://www.ebi.ac.uk/interpro/entry/InterPro/IPR027384), | | |
| **KEGG_PATHWAY** | [Parathyroid hormone synthesis, secretion and action](https://david.ncifcrf.gov/kegg.jsp?path=hsa04928$Parathyroid%20hormone%20synthesis,%20secretion%20and%20action&termId=520048061&source=kegg), [Transcriptional misregulation in cancer](https://david.ncifcrf.gov/kegg.jsp?path=hsa05202$Transcriptional%20misregulation%20in%20cancer&termId=520048129&source=kegg), | | |
| **OMIM_DISEASE** | [Cleidocranial dysplasia](http://omim.org/entry/119600), [Cleidocranial dysplasia, forme fruste, dental anomalies only](http://omim.org/entry/119600), [Cleidocranial dysplasia, forme fruste, with brachydactyly](http://omim.org/entry/119600), [Metaphyseal dysplasia with maxillary hypoplasia with or without brachydactyly](http://omim.org/entry/156510), | | |
| **PIR_SUPERFAMILY** | [runt-related transcription factor RUNX](http://pir.georgetown.edu/cgi-bin/ipcSF?id=PIRSF009374), | | |
| **UP_KW_BIOLOGICAL_PROCESS** | [Differentiation](http://www.uniprot.org/keywords/?query=KW-0221), [Transcription](http://www.uniprot.org/keywords/?query=KW-0804), [Transcription regulation](http://www.uniprot.org/keywords/?query=KW-0805), | | |
| **UP_KW_CELLULAR_COMPONENT** | [Nucleus](http://www.uniprot.org/keywords/?query=KW-0539), | | |
| **UP_KW_DISEASE** | [Disease variant](http://www.uniprot.org/keywords/?query=KW-0225), | | |
| **UP_KW_MOLECULAR_FUNCTION** | [DNA-binding](http://www.uniprot.org/keywords/?query=KW-0238), | | |
| **UP_KW_PTM** | [Methylation](http://www.uniprot.org/keywords/?query=KW-0488), [Phosphoprotein](http://www.uniprot.org/keywords/?query=KW-0597), [Ubl conjugation](http://www.uniprot.org/keywords/?query=KW-0832), [Isopeptide bond](http://www.uniprot.org/keywords/?query=KW-1017), | | |
| **UP_SEQ_FEATURE** | BINDING:Chloride 1, BINDING:Chloride 1; via amide nitrogen, BINDING:Chloride 2, BINDING:Chloride 2; via amide nitrogen, COMPBIAS:Basic and acidic residues, COMPBIAS:Polar residues, COMPBIAS:Pro residues, CROSSLNK:Glycyl lysine isopeptide (Lys-Gly) (interchain with G-Cter in SUMO2), DOMAIN:Runt, MUTAGEN:S->A: Reduced DNA-binding and impaired phosphorylation., REGION:Disordered, REGION:Interaction with KAT6A, REGION:Interaction with KAT6B, REGION:Required for interaction with FOXO1, | | |
| **RAPGEF1** | [**Rap guanine nucleotide exchange factor 1(RAPGEF1)**](https://david.ncifcrf.gov/geneReportFull.jsp?rowids=2889) | [**Related Genes**](https://david.ncifcrf.gov/relatedGenes.jsp?id=2889) | [**Homo sapiens**](http://www.ncbi.nlm.nih.gov/Taxonomy/Browser/wwwtax.cgi?name=Homo%20sapiens) |
| **BIOCARTA** | [Integrin Signaling Pathway](https://david.ncifcrf.gov/biocarta.jsp?path=h_integrinPathway$Integrin%20Signaling%20Pathway&termId=30000165&source=biocarta), [Signaling of Hepatocyte Growth Factor Receptor](https://david.ncifcrf.gov/biocarta.jsp?path=h_metPathway$Signaling%20of%20Hepatocyte%20Growth%20Factor%20Receptor&termId=30000187&source=biocarta), | | |
| **GOTERM_BP_DIRECT** | [activation of MAPKK activity](http://www.ebi.ac.uk/QuickGO/GTerm?id=GO:0000186), [blood vessel development](http://www.ebi.ac.uk/QuickGO/GTerm?id=GO:0001568), [signal transduction](http://www.ebi.ac.uk/QuickGO/GTerm?id=GO:0007165), [transmembrane receptor protein tyrosine kinase signaling pathway](http://www.ebi.ac.uk/QuickGO/GTerm?id=GO:0007169), [small GTPase mediated signal transduction](http://www.ebi.ac.uk/QuickGO/GTerm?id=GO:0007264), [Ras protein signal transduction](http://www.ebi.ac.uk/QuickGO/GTerm?id=GO:0007265), [nervous system development](http://www.ebi.ac.uk/QuickGO/GTerm?id=GO:0007399), [positive regulation of neuron projection development](http://www.ebi.ac.uk/QuickGO/GTerm?id=GO:0010976), [cytokine-mediated signaling pathway](http://www.ebi.ac.uk/QuickGO/GTerm?id=GO:0019221), [Rap protein signal transduction](http://www.ebi.ac.uk/QuickGO/GTerm?id=GO:0032486), [nerve growth factor signaling pathway](http://www.ebi.ac.uk/QuickGO/GTerm?id=GO:0038180), [positive regulation of GTPase activity](http://www.ebi.ac.uk/QuickGO/GTerm?id=GO:0043547), [regulation of JNK cascade](http://www.ebi.ac.uk/QuickGO/GTerm?id=GO:0046328), [positive regulation of Ras protein signal transduction](http://www.ebi.ac.uk/QuickGO/GTerm?id=GO:0046579), [negative regulation of Ras protein signal transduction](http://www.ebi.ac.uk/QuickGO/GTerm?id=GO:0046580), [platelet-derived growth factor receptor signaling pathway](http://www.ebi.ac.uk/QuickGO/GTerm?id=GO:0048008), [negative regulation of protein kinase B signaling](http://www.ebi.ac.uk/QuickGO/GTerm?id=GO:0051898), [establishment of endothelial barrier](http://www.ebi.ac.uk/QuickGO/GTerm?id=GO:0061028), [negative regulation of ERK1 and ERK2 cascade](http://www.ebi.ac.uk/QuickGO/GTerm?id=GO:0070373), [positive regulation of ERK1 and ERK2 cascade](http://www.ebi.ac.uk/QuickGO/GTerm?id=GO:0070374), [cellular response to cAMP](http://www.ebi.ac.uk/QuickGO/GTerm?id=GO:0071320), [negative regulation of canonical Wnt signaling pathway](http://www.ebi.ac.uk/QuickGO/GTerm?id=GO:0090090), [activation of GTPase activity](http://www.ebi.ac.uk/QuickGO/GTerm?id=GO:0090630), [cell-cell adhesion](http://www.ebi.ac.uk/QuickGO/GTerm?id=GO:0098609), [regulation of cell junction assembly](http://www.ebi.ac.uk/QuickGO/GTerm?id=GO:1901888), [positive regulation of Fc-gamma receptor signaling pathway involved in phagocytosis](http://www.ebi.ac.uk/QuickGO/GTerm?id=GO:1905451), [cellular response to nerve growth factor stimulus](http://www.ebi.ac.uk/QuickGO/GTerm?id=GO:1990090), [negative regulation of neural precursor cell proliferation](http://www.ebi.ac.uk/QuickGO/GTerm?id=GO:2000178), | | |
| **GOTERM_CC_DIRECT** | [cytoplasm](http://www.ebi.ac.uk/QuickGO/GTerm?id=GO:0005737), [early endosome](http://www.ebi.ac.uk/QuickGO/GTerm?id=GO:0005769), [cytosol](http://www.ebi.ac.uk/QuickGO/GTerm?id=GO:0005829), [plasma membrane](http://www.ebi.ac.uk/QuickGO/GTerm?id=GO:0005886), [phagocytic vesicle membrane](http://www.ebi.ac.uk/QuickGO/GTerm?id=GO:0030670), [macromolecular complex](http://www.ebi.ac.uk/QuickGO/GTerm?id=GO:0032991), [intracellular membrane-bounded organelle](http://www.ebi.ac.uk/QuickGO/GTerm?id=GO:0043231), [perinuclear region of cytoplasm](http://www.ebi.ac.uk/QuickGO/GTerm?id=GO:0048471), | | |
| **GOTERM_MF_DIRECT** | [guanyl-nucleotide exchange factor activity](http://www.ebi.ac.uk/QuickGO/GTerm?id=GO:0005085), [protein binding](http://www.ebi.ac.uk/QuickGO/GTerm?id=GO:0005515), [SH3 domain binding](http://www.ebi.ac.uk/QuickGO/GTerm?id=GO:0017124), | | |
| **INTERPRO** | [Ras-like guanine nucleotide exchange factor, N-terminal](https://www.ebi.ac.uk/interpro/entry/InterPro/IPR000651), [Guanine-nucleotide dissociation stimulator CDC25](https://www.ebi.ac.uk/interpro/entry/InterPro/IPR001895), [Ras guanine nucleotide exchange factor](https://www.ebi.ac.uk/interpro/entry/InterPro/IPR008937), [Ras guanine-nucleotide exchange factor, conserved site](https://www.ebi.ac.uk/interpro/entry/InterPro/IPR019804), [Ras guanine nucleotide exchange factor, domain](https://www.ebi.ac.uk/interpro/entry/InterPro/IPR023578), | | |
| **KEGG_PATHWAY** | [Rap1 signaling pathway](https://david.ncifcrf.gov/kegg.jsp?path=hsa04015$Rap1%20signaling%20pathway&termId=520047944&source=kegg), [Focal adhesion](https://david.ncifcrf.gov/kegg.jsp?path=hsa04510$Focal%20adhesion&termId=520047995&source=kegg), [Neurotrophin signaling pathway](https://david.ncifcrf.gov/kegg.jsp?path=hsa04722$Neurotrophin%20signaling%20pathway&termId=520048030&source=kegg), [Insulin signaling pathway](https://david.ncifcrf.gov/kegg.jsp?path=hsa04910$Insulin%20signaling%20pathway&termId=520048043&source=kegg), [Renal cell carcinoma](https://david.ncifcrf.gov/kegg.jsp?path=hsa05211$Renal%20cell%20carcinoma&termId=520048137&source=kegg), | | |
| **SMART** | [RasGEF](http://smart.embl.de/smart/do_annotation.pl?DOMAIN=SM00147), [RasGEFN](http://smart.embl.de/smart/do_annotation.pl?DOMAIN=SM00229), | | |
| **UP_KW_BIOLOGICAL_PROCESS** | [Neurogenesis](http://www.uniprot.org/keywords/?query=KW-0524), | | |
| **UP_KW_CELLULAR_COMPONENT** | [Endosome](http://www.uniprot.org/keywords/?query=KW-0967), | | |
| **UP_KW_DOMAIN** | [SH3-binding](http://www.uniprot.org/keywords/?query=KW-0729), | | |
| **UP_KW_MOLECULAR_FUNCTION** | [Guanine-nucleotide releasing factor](http://www.uniprot.org/keywords/?query=KW-0344), | | |
| **UP_KW_PTM** | [Phosphoprotein](http://www.uniprot.org/keywords/?query=KW-0597), [Ubl conjugation](http://www.uniprot.org/keywords/?query=KW-0832), [Isopeptide bond](http://www.uniprot.org/keywords/?query=KW-1017), | | |
| **UP_SEQ_FEATURE** | COMPBIAS:Polar residues, COMPBIAS:Pro residues, CROSSLNK:Glycyl lysine isopeptide (Lys-Gly) (interchain with G-Cter in SUMO2), DOMAIN:N-terminal Ras-GEF, DOMAIN:Ras-GEF, MOTIF:SH3-binding, MUTAGEN:Y->F: Abolishes phosphorylation by HCK., REGION:Disordered, | | |
| **RIN2** | [**Ras and Rab interactor 2(RIN2)**](https://david.ncifcrf.gov/geneReportFull.jsp?rowids=54453) | [**Related Genes**](https://david.ncifcrf.gov/relatedGenes.jsp?id=54453) | [**Homo sapiens**](http://www.ncbi.nlm.nih.gov/Taxonomy/Browser/wwwtax.cgi?name=Homo%20sapiens) |
| **GOTERM_BP_DIRECT** | [endocytosis](http://www.ebi.ac.uk/QuickGO/GTerm?id=GO:0006897), [signal transduction](http://www.ebi.ac.uk/QuickGO/GTerm?id=GO:0007165), [small GTPase mediated signal transduction](http://www.ebi.ac.uk/QuickGO/GTerm?id=GO:0007264), [positive regulation of endothelial cell migration](http://www.ebi.ac.uk/QuickGO/GTerm?id=GO:0010595), [positive regulation of GTPase activity](http://www.ebi.ac.uk/QuickGO/GTerm?id=GO:0043547), [regulation of catalytic activity](http://www.ebi.ac.uk/QuickGO/GTerm?id=GO:0050790), [positive regulation of endothelial cell-matrix adhesion via fibronectin](http://www.ebi.ac.uk/QuickGO/GTerm?id=GO:1904906), [positive regulation of vasculogenesis](http://www.ebi.ac.uk/QuickGO/GTerm?id=GO:2001214), | | |
| **GOTERM_CC_DIRECT** | [cytosol](http://www.ebi.ac.uk/QuickGO/GTerm?id=GO:0005829), [endocytic vesicle](http://www.ebi.ac.uk/QuickGO/GTerm?id=GO:0030139), | | |
| **GOTERM_MF_DIRECT** | [guanyl-nucleotide exchange factor activity](http://www.ebi.ac.uk/QuickGO/GTerm?id=GO:0005085), [GTPase activator activity](http://www.ebi.ac.uk/QuickGO/GTerm?id=GO:0005096), [GTPase regulator activity](http://www.ebi.ac.uk/QuickGO/GTerm?id=GO:0030695), [small GTPase binding](http://www.ebi.ac.uk/QuickGO/GTerm?id=GO:0031267), | | |
| **INTERPRO** | [Ras-association](https://www.ebi.ac.uk/interpro/entry/InterPro/IPR000159), [SH2 domain](https://www.ebi.ac.uk/interpro/entry/InterPro/IPR000980), [Vacuolar sorting protein 9](https://www.ebi.ac.uk/interpro/entry/InterPro/IPR003123), | | |
| **OMIM_DISEASE** | [Macrocephaly, alopecia, cutis laxa, and scoliosis](http://omim.org/entry/613075), | | |
| **SMART** | [VPS9](http://smart.embl.de/smart/do_annotation.pl?DOMAIN=SM00167), [RA](http://smart.embl.de/smart/do_annotation.pl?DOMAIN=SM00314), | | |
| **UP_KW_BIOLOGICAL_PROCESS** | [Endocytosis](http://www.uniprot.org/keywords/?query=KW-0254), | | |
| **UP_KW_CELLULAR_COMPONENT** | [Cytoplasm](http://www.uniprot.org/keywords/?query=KW-0963), | | |
| **UP_KW_DOMAIN** | [SH2 domain](http://www.uniprot.org/keywords/?query=KW-0727), | | |
| **UP_KW_MOLECULAR_FUNCTION** | [GTPase activation](http://www.uniprot.org/keywords/?query=KW-0343), | | |
| **UP_KW_PTM** | [Phosphoprotein](http://www.uniprot.org/keywords/?query=KW-0597), | | |
| **UP_SEQ_FEATURE** | COMPBIAS:Polar residues, DOMAIN:Ras-associating, DOMAIN:SH2, DOMAIN:VPS9, REGION:Disordered, | | |
| **ARHGAP22** | [**Rho GTPase activating protein 22(ARHGAP22)**](https://david.ncifcrf.gov/geneReportFull.jsp?rowids=58504) | [**Related Genes**](https://david.ncifcrf.gov/relatedGenes.jsp?id=58504) | [**Homo sapiens**](http://www.ncbi.nlm.nih.gov/Taxonomy/Browser/wwwtax.cgi?name=Homo%20sapiens) |
| **GOTERM_BP_DIRECT** | [angiogenesis](http://www.ebi.ac.uk/QuickGO/GTerm?id=GO:0001525), [signal transduction](http://www.ebi.ac.uk/QuickGO/GTerm?id=GO:0007165), [cell differentiation](http://www.ebi.ac.uk/QuickGO/GTerm?id=GO:0030154), [positive regulation of GTPase activity](http://www.ebi.ac.uk/QuickGO/GTerm?id=GO:0043547), [regulation of small GTPase mediated signal transduction](http://www.ebi.ac.uk/QuickGO/GTerm?id=GO:0051056), [activation of GTPase activity](http://www.ebi.ac.uk/QuickGO/GTerm?id=GO:0090630), | | |
| **GOTERM_CC_DIRECT** | [nucleus](http://www.ebi.ac.uk/QuickGO/GTerm?id=GO:0005634), [cytosol](http://www.ebi.ac.uk/QuickGO/GTerm?id=GO:0005829), [focal adhesion](http://www.ebi.ac.uk/QuickGO/GTerm?id=GO:0005925), [glutamatergic synapse](http://www.ebi.ac.uk/QuickGO/GTerm?id=GO:0098978), | | |
| **GOTERM_MF_DIRECT** | [GTPase activator activity](http://www.ebi.ac.uk/QuickGO/GTerm?id=GO:0005096), [protein binding](http://www.ebi.ac.uk/QuickGO/GTerm?id=GO:0005515), | | |
| **INTERPRO** | [Rho GTPase-activating protein domain](https://www.ebi.ac.uk/interpro/entry/InterPro/IPR000198), [Pleckstrin homology domain](https://www.ebi.ac.uk/interpro/entry/InterPro/IPR001849), [Rho GTPase activation protein](https://www.ebi.ac.uk/interpro/entry/InterPro/IPR008936), [Pleckstrin homology-like domain](https://www.ebi.ac.uk/interpro/entry/InterPro/IPR011993), | | |
| **SMART** | [PH](http://smart.embl.de/smart/do_annotation.pl?DOMAIN=SM00233), [RhoGAP](http://smart.embl.de/smart/do_annotation.pl?DOMAIN=SM00324), | | |
| **UP_KW_BIOLOGICAL_PROCESS** | [Angiogenesis](http://www.uniprot.org/keywords/?query=KW-0037), [Differentiation](http://www.uniprot.org/keywords/?query=KW-0221), [Transcription](http://www.uniprot.org/keywords/?query=KW-0804), [Transcription regulation](http://www.uniprot.org/keywords/?query=KW-0805), | | |
| **UP_KW_CELLULAR_COMPONENT** | [Nucleus](http://www.uniprot.org/keywords/?query=KW-0539), [Cytoplasm](http://www.uniprot.org/keywords/?query=KW-0963), | | |
| **UP_KW_DOMAIN** | [Coiled coil](http://www.uniprot.org/keywords/?query=KW-0175), | | |
| **UP_KW_MOLECULAR_FUNCTION** | [Developmental protein](http://www.uniprot.org/keywords/?query=KW-0217), [GTPase activation](http://www.uniprot.org/keywords/?query=KW-0343), [Developmental protein](http://www.uniprot.org/keywords/?query=KW-9996), | | |
| **UP_KW_PTM** | [Phosphoprotein](http://www.uniprot.org/keywords/?query=KW-0597), | | |
| **UP_SEQ_FEATURE** | COMPBIAS:Polar residues, DOMAIN:PH, DOMAIN:Rho-GAP, REGION:Disordered, | | |
| **ARHGAP24** | [**Rho GTPase activating protein 24(ARHGAP24)**](https://david.ncifcrf.gov/geneReportFull.jsp?rowids=83478) | [**Related Genes**](https://david.ncifcrf.gov/relatedGenes.jsp?id=83478) | [**Homo sapiens**](http://www.ncbi.nlm.nih.gov/Taxonomy/Browser/wwwtax.cgi?name=Homo%20sapiens) |
| **GOTERM_BP_DIRECT** | [angiogenesis](http://www.ebi.ac.uk/QuickGO/GTerm?id=GO:0001525), [signal transduction](http://www.ebi.ac.uk/QuickGO/GTerm?id=GO:0007165), [cell differentiation](http://www.ebi.ac.uk/QuickGO/GTerm?id=GO:0030154), [negative regulation of Rac protein signal transduction](http://www.ebi.ac.uk/QuickGO/GTerm?id=GO:0035021), [wound healing, spreading of epidermal cells](http://www.ebi.ac.uk/QuickGO/GTerm?id=GO:0035313), [positive regulation of GTPase activity](http://www.ebi.ac.uk/QuickGO/GTerm?id=GO:0043547), [regulation of small GTPase mediated signal transduction](http://www.ebi.ac.uk/QuickGO/GTerm?id=GO:0051056), [activation of GTPase activity](http://www.ebi.ac.uk/QuickGO/GTerm?id=GO:0090630), [negative regulation of ruffle assembly](http://www.ebi.ac.uk/QuickGO/GTerm?id=GO:1900028), | | |
| **GOTERM_CC_DIRECT** | [cytoplasm](http://www.ebi.ac.uk/QuickGO/GTerm?id=GO:0005737), [cytosol](http://www.ebi.ac.uk/QuickGO/GTerm?id=GO:0005829), [cytoskeleton](http://www.ebi.ac.uk/QuickGO/GTerm?id=GO:0005856), [adherens junction](http://www.ebi.ac.uk/QuickGO/GTerm?id=GO:0005912), [focal adhesion](http://www.ebi.ac.uk/QuickGO/GTerm?id=GO:0005925), [cell projection](http://www.ebi.ac.uk/QuickGO/GTerm?id=GO:0042995), | | |
| **GOTERM_MF_DIRECT** | [GTPase activator activity](http://www.ebi.ac.uk/QuickGO/GTerm?id=GO:0005096), [protein binding](http://www.ebi.ac.uk/QuickGO/GTerm?id=GO:0005515), | | |
| **INTERPRO** | [Rho GTPase-activating protein domain](https://www.ebi.ac.uk/interpro/entry/InterPro/IPR000198), [Pleckstrin homology domain](https://www.ebi.ac.uk/interpro/entry/InterPro/IPR001849), [Rho GTPase activation protein](https://www.ebi.ac.uk/interpro/entry/InterPro/IPR008936), [Pleckstrin homology-like domain](https://www.ebi.ac.uk/interpro/entry/InterPro/IPR011993), | | |
| **SMART** | [PH](http://smart.embl.de/smart/do_annotation.pl?DOMAIN=SM00233), [RhoGAP](http://smart.embl.de/smart/do_annotation.pl?DOMAIN=SM00324), | | |
| **UP_KW_BIOLOGICAL_PROCESS** | [Angiogenesis](http://www.uniprot.org/keywords/?query=KW-0037), [Differentiation](http://www.uniprot.org/keywords/?query=KW-0221), | | |
| **UP_KW_CELLULAR_COMPONENT** | [Cytoskeleton](http://www.uniprot.org/keywords/?query=KW-0206), [Cytoplasm](http://www.uniprot.org/keywords/?query=KW-0963), [Cell junction](http://www.uniprot.org/keywords/?query=KW-0965), [Cell projection](http://www.uniprot.org/keywords/?query=KW-0966), | | |
| **UP_KW_DOMAIN** | [Coiled coil](http://www.uniprot.org/keywords/?query=KW-0175), | | |
| **UP_KW_MOLECULAR_FUNCTION** | [Developmental protein](http://www.uniprot.org/keywords/?query=KW-0217), [GTPase activation](http://www.uniprot.org/keywords/?query=KW-0343), [Developmental protein](http://www.uniprot.org/keywords/?query=KW-9996), | | |
| **UP_KW_PTM** | [Phosphoprotein](http://www.uniprot.org/keywords/?query=KW-0597), | | |
| **UP_SEQ_FEATURE** | COMPBIAS:Basic and acidic residues, COMPBIAS:Polar residues, DOMAIN:PH, DOMAIN:Rho-GAP, MUTAGEN:R->A: Loss of function., MUTAGEN:R->K: Does not abolish the effect on actin stress fibers but moderates its capability to induce membrane protrusions., REGION:Disordered, | | |
| **ARHGEF10L** | [**Rho guanine nucleotide exchange factor 10 like(ARHGEF10L)**](https://david.ncifcrf.gov/geneReportFull.jsp?rowids=55160) | [**Related Genes**](https://david.ncifcrf.gov/relatedGenes.jsp?id=55160) | [**Homo sapiens**](http://www.ncbi.nlm.nih.gov/Taxonomy/Browser/wwwtax.cgi?name=Homo%20sapiens) |
| **GOTERM_BP_DIRECT** | [actin cytoskeleton organization](http://www.ebi.ac.uk/QuickGO/GTerm?id=GO:0030036), [SREBP signaling pathway](http://www.ebi.ac.uk/QuickGO/GTerm?id=GO:0032933), [regulation of catalytic activity](http://www.ebi.ac.uk/QuickGO/GTerm?id=GO:0050790), [regulation of small GTPase mediated signal transduction](http://www.ebi.ac.uk/QuickGO/GTerm?id=GO:0051056), [positive regulation of stress fiber assembly](http://www.ebi.ac.uk/QuickGO/GTerm?id=GO:0051496), | | |
| **GOTERM_CC_DIRECT** | [cytosol](http://www.ebi.ac.uk/QuickGO/GTerm?id=GO:0005829), | | |
| **GOTERM_MF_DIRECT** | [guanyl-nucleotide exchange factor activity](http://www.ebi.ac.uk/QuickGO/GTerm?id=GO:0005085), [GTPase activator activity](http://www.ebi.ac.uk/QuickGO/GTerm?id=GO:0005096), | | |
| **INTERPRO** | [Dbl homology (DH) domain](https://www.ebi.ac.uk/interpro/entry/InterPro/IPR000219), [Pleckstrin homology-like domain](https://www.ebi.ac.uk/interpro/entry/InterPro/IPR011993), [WD40/YVTN repeat-like-containing domain](https://www.ebi.ac.uk/interpro/entry/InterPro/IPR015943), | | |
| **SMART** | [RhoGEF](http://smart.embl.de/smart/do_annotation.pl?DOMAIN=SM00325), | | |
| **UP_KW_CELLULAR_COMPONENT** | [Cytoplasm](http://www.uniprot.org/keywords/?query=KW-0963), | | |
| **UP_KW_DOMAIN** | [Coiled coil](http://www.uniprot.org/keywords/?query=KW-0175), | | |
| **UP_KW_MOLECULAR_FUNCTION** | [Guanine-nucleotide releasing factor](http://www.uniprot.org/keywords/?query=KW-0344), | | |
| **UP_KW_PTM** | [Phosphoprotein](http://www.uniprot.org/keywords/?query=KW-0597), | | |
| **UP_SEQ_FEATURE** | COMPBIAS:Acidic residues, COMPBIAS:Basic and acidic residues, COMPBIAS:Polar residues, DOMAIN:DH, REGION:Disordered, | | |
| **SAMHD1** | [**SAM and HD domain containing deoxynucleoside triphosphate triphosphohydrolase 1(SAMHD1)**](https://david.ncifcrf.gov/geneReportFull.jsp?rowids=25939) | [**Related Genes**](https://david.ncifcrf.gov/relatedGenes.jsp?id=25939) | [**Homo sapiens**](http://www.ncbi.nlm.nih.gov/Taxonomy/Browser/wwwtax.cgi?name=Homo%20sapiens) |
| **GOTERM_BP_DIRECT** | [double-strand break repair via homologous recombination](http://www.ebi.ac.uk/QuickGO/GTerm?id=GO:0000724), [dGTP catabolic process](http://www.ebi.ac.uk/QuickGO/GTerm?id=GO:0006203), [DNA replication](http://www.ebi.ac.uk/QuickGO/GTerm?id=GO:0006260), [DNA repair](http://www.ebi.ac.uk/QuickGO/GTerm?id=GO:0006281), [immune response](http://www.ebi.ac.uk/QuickGO/GTerm?id=GO:0006955), [cellular response to DNA damage stimulus](http://www.ebi.ac.uk/QuickGO/GTerm?id=GO:0006974), [deoxyribonucleotide catabolic process](http://www.ebi.ac.uk/QuickGO/GTerm?id=GO:0009264), [viral process](http://www.ebi.ac.uk/QuickGO/GTerm?id=GO:0016032), [somatic hypermutation of immunoglobulin genes](http://www.ebi.ac.uk/QuickGO/GTerm?id=GO:0016446), [innate immune response](http://www.ebi.ac.uk/QuickGO/GTerm?id=GO:0045087), [regulation of innate immune response](http://www.ebi.ac.uk/QuickGO/GTerm?id=GO:0045088), [dATP catabolic process](http://www.ebi.ac.uk/QuickGO/GTerm?id=GO:0046061), [protein homotetramerization](http://www.ebi.ac.uk/QuickGO/GTerm?id=GO:0051289), [defense response to virus](http://www.ebi.ac.uk/QuickGO/GTerm?id=GO:0051607), [type I interferon signaling pathway](http://www.ebi.ac.uk/QuickGO/GTerm?id=GO:0060337), [negative regulation of type I interferon-mediated signaling pathway](http://www.ebi.ac.uk/QuickGO/GTerm?id=GO:0060339), [RNA phosphodiester bond hydrolysis](http://www.ebi.ac.uk/QuickGO/GTerm?id=GO:0090501), | | |
| **GOTERM_CC_DIRECT** | [nucleus](http://www.ebi.ac.uk/QuickGO/GTerm?id=GO:0005634), [nucleoplasm](http://www.ebi.ac.uk/QuickGO/GTerm?id=GO:0005654), [chromosome](http://www.ebi.ac.uk/QuickGO/GTerm?id=GO:0005694), [plasma membrane](http://www.ebi.ac.uk/QuickGO/GTerm?id=GO:0005886), [site of double-strand break](http://www.ebi.ac.uk/QuickGO/GTerm?id=GO:0035861), [tetraspanin-enriched microdomain](http://www.ebi.ac.uk/QuickGO/GTerm?id=GO:0097197), | | |
| **GOTERM_MF_DIRECT** | [nucleic acid binding](http://www.ebi.ac.uk/QuickGO/GTerm?id=GO:0003676), [single-stranded DNA binding](http://www.ebi.ac.uk/QuickGO/GTerm?id=GO:0003697), [RNA binding](http://www.ebi.ac.uk/QuickGO/GTerm?id=GO:0003723), [catalytic activity](http://www.ebi.ac.uk/QuickGO/GTerm?id=GO:0003824), [ribonuclease activity](http://www.ebi.ac.uk/QuickGO/GTerm?id=GO:0004540), [protein binding](http://www.ebi.ac.uk/QuickGO/GTerm?id=GO:0005515), [GTP binding](http://www.ebi.ac.uk/QuickGO/GTerm?id=GO:0005525), [zinc ion binding](http://www.ebi.ac.uk/QuickGO/GTerm?id=GO:0008270), [dGTPase activity](http://www.ebi.ac.uk/QuickGO/GTerm?id=GO:0008832), [triphosphoric monoester hydrolase activity](http://www.ebi.ac.uk/QuickGO/GTerm?id=GO:0016793), [dGTP binding](http://www.ebi.ac.uk/QuickGO/GTerm?id=GO:0032567), [identical protein binding](http://www.ebi.ac.uk/QuickGO/GTerm?id=GO:0042802), | | |
| **INTERPRO** | [Sterile alpha motif domain](https://www.ebi.ac.uk/interpro/entry/InterPro/IPR001660), [HD/PDEase domain](https://www.ebi.ac.uk/interpro/entry/InterPro/IPR003607), [HD domain](https://www.ebi.ac.uk/interpro/entry/InterPro/IPR006674), [Sterile alpha motif/pointed domain](https://www.ebi.ac.uk/interpro/entry/InterPro/IPR013761), | | |
| **KEGG_PATHWAY** | [Human immunodeficiency virus 1 infection](https://david.ncifcrf.gov/kegg.jsp?path=hsa05170$Human%20immunodeficiency%20virus%201%20infection&termId=520048126&source=kegg), | | |
| **OMIM_DISEASE** | [Aicardi-Goutieres syndrome 5](http://omim.org/entry/612952), [Chilblain lupus 2](http://omim.org/entry/614415), | | |
| **SMART** | [SAM](http://smart.embl.de/smart/do_annotation.pl?DOMAIN=SM00454), [HDc](http://smart.embl.de/smart/do_annotation.pl?DOMAIN=SM00471), | | |
| **UP_KW_BIOLOGICAL_PROCESS** | [Antiviral defense](http://www.uniprot.org/keywords/?query=KW-0051), [DNA damage](http://www.uniprot.org/keywords/?query=KW-0227), [DNA repair](http://www.uniprot.org/keywords/?query=KW-0234), [DNA replication](http://www.uniprot.org/keywords/?query=KW-0235), [Immunity](http://www.uniprot.org/keywords/?query=KW-0391), [Innate immunity](http://www.uniprot.org/keywords/?query=KW-0399), [Host-virus interaction](http://www.uniprot.org/keywords/?query=KW-0945), | | |
| **UP_KW_CELLULAR_COMPONENT** | [Chromosome](http://www.uniprot.org/keywords/?query=KW-0158), [Nucleus](http://www.uniprot.org/keywords/?query=KW-0539), | | |
| **UP_KW_DISEASE** | [Disease variant](http://www.uniprot.org/keywords/?query=KW-0225), [Aicardi-Goutieres syndrome](http://www.uniprot.org/keywords/?query=KW-0948), | | |
| **UP_KW_LIGAND** | [GTP-binding](http://www.uniprot.org/keywords/?query=KW-0342), [Metal-binding](http://www.uniprot.org/keywords/?query=KW-0479), [Nucleotide-binding](http://www.uniprot.org/keywords/?query=KW-0547), [Zinc](http://www.uniprot.org/keywords/?query=KW-0862), | | |
| **UP_KW_MOLECULAR_FUNCTION** | [Allosteric enzyme](http://www.uniprot.org/keywords/?query=KW-0021), [Hydrolase](http://www.uniprot.org/keywords/?query=KW-0378), | | |
| **UP_KW_PTM** | [Acetylation](http://www.uniprot.org/keywords/?query=KW-0007), [Phosphoprotein](http://www.uniprot.org/keywords/?query=KW-0597), [Ubl conjugation](http://www.uniprot.org/keywords/?query=KW-0832), [Isopeptide bond](http://www.uniprot.org/keywords/?query=KW-1017), | | |
| **UP_SEQ_FEATURE** | BINDING:GTP, BINDING:GTP; shared with neighboring subunit, BINDING:Substrate, BINDING:dNTP, BINDING:dNTP; shared with neighboring subunit, BINDING:dNTP; via amide nitrogen; shared with neighboring subunit, COMPBIAS:Basic and acidic residues, COMPBIAS:Polar residues, CROSSLNK:Glycyl lysine isopeptide (Lys-Gly) (interchain with G-Cter in SUMO2), DOMAIN:HD, DOMAIN:SAM, METAL:Zinc, METAL:Zinc; via tele nitrogen, MUTAGEN:C->F: Increased stability of the tetramer and increased deoxynucleoside triphosphate (dNTPase) activity; when associated with F-77 and R-111., MUTAGEN:D->A: Abolished ability to restrict infection by viruses., MUTAGEN:D->A: Abolishes dNTPase activity; when associated with A-149., MUTAGEN:D->A: Impaired homotetramerization and slightly reduced dNTPase activity. Impaired homotetramerization and reduced dNTPase activity; when associated with A-358., MUTAGEN:D->A: Impairs homotetramerization and nearly abolishes dNTPase activity., MUTAGEN:D->A: Loss of function in defense response to virus. Loss of dNTPase activity. Does not affect oligomerization., MUTAGEN:D->N,A: Loss of dNTPase activity., MUTAGEN:D->R: Impairs homotetramerization and nearly abolishes dNTPase activity; when associated with K-364., MUTAGEN:H->A: Abolished ability to restrict infection by viruses., MUTAGEN:H->A: Abolished dNTPase activity without affecting homotetramerization., MUTAGEN:H->A: Abolished dNTPase activity without affecting homotetramerization. Abolished ability to restrict infection by viruses., MUTAGEN:H->A: Abolishes dNTPase activity; when associated with G-374., MUTAGEN:H->A: Impairs homotetramerization and abolishes dNTPase activity; when associated with A-352 and A-377., MUTAGEN:H->K: Impairs homotetramerization and nearly abolishes dNTPase activity; when associated with R-361., MUTAGEN:H->R: Increased stability of the tetramer and increased deoxynucleoside triphosphate (dNTPase) activity; when associated with F-77 and F-80., MUTAGEN:HD->RN: Abolishes zinc binding and dNTPase activity. Does not affect ability to promote DNA end resection at stalled replication forks., MUTAGEN:K->A,E: Abolishes proteasomal degradation triggered by the viral accessory protein vpx., MUTAGEN:K->A: Abolishes dNTPase activity; when associated with A-315 and A-366. Does not affect ability to promote DNA end resection at stalled replication forks; when associated with A-315., MUTAGEN:K->A: Impairs homotetramerization and abolishes dNTPase activity; when associated with A-352 and A-376., MUTAGEN:K->A: Impairs homotetramerization and abolishes dNTPase activity; when associated with A-537 and D-540., MUTAGEN:L->D: Impairs homotetramerization and abolishes dNTPase activity; when associated with A-537 and A-534., MUTAGEN:L->F: Increased stability of the tetramer and increased deoxynucleoside triphosphate (dNTPase) activity; when associated with F-77 and F-80 and R-111., MUTAGEN:N->A: Impaired homotetramerization and slightly reduced dNTPase activity. Impaired homotetramerization and reduced dNTPase activity A-330., MUTAGEN:P->A: Promotes ability to restrict infection by viruses., MUTAGEN:Q->A: Abolished dNTPase activity without affecting homotetramerization., MUTAGEN:Q->A: Abolished dNTPase activity without affecting homotetramerization. Abolished dNTPase activity; when associated with A-319., MUTAGEN:Q->A: Loss of function in defense response to virus. Does not affect oligomerization. Retains dNTPase activity., MUTAGEN:Q->E,A: Impairs homotetramerization and nearly abolishes dNTPase activity; when associated with K-145., MUTAGEN:R->A,E: Abolishes proteasomal degradation triggered by the viral accessory protein vpx., MUTAGEN:R->A: Abolished ability to restrict infection by viruses., MUTAGEN:R->A: Abolishes dNTPase activity; when associated with A-312 and A-315., MUTAGEN:R->A: Impairs homotetramerization and abolishes dNTPase activity; when associated with A-376 and A-377., MUTAGEN:R->A: Impairs homotetramerization and nearly abolishes dNTPase activity. Abolished ability to restrict infection by viruses., MUTAGEN:R->D: Abolished homotetramerization and dNTPase activity., MUTAGEN:R->E: Decreases dNTPase activity. Impairs homotetramerization and nearly abolishes dNTPase activity; when associated with E-451., MUTAGEN:R->E: Impairs homotetramerization and abolishes dNTPase activity., MUTAGEN:R->G: Loss of function in defense response to virus., MUTAGEN:R->K: Impairs homotetramerization and nearly abolishes dNTPase activity; when associated with E-145., MUTAGEN:T->A,V: Impaired ability to promote DNA end resection at stalled replication forks. Promotes dNTPase activity and ability to restrict infection by viruses., MUTAGEN:T->A: Loss of phosphorylation by human cytomegalovirus/HCMV kinase UL97., MUTAGEN:T->E: Mimicks phosphorylation state, retains ability to promote DNA end resection at stalled replication forks. Induces large conformational changes that impair homotetramerization, leading to reduced dNTPase activity and decreased ability to restrict infection by viruses., MUTAGEN:V->A: Impairs homotetramerization and abolishes dNTPase activity; when associated with A-534 and D-540., MUTAGEN:Y->A: Abolished ability to restrict infection by viruses. Abolishes dNTPase activity; when associated with A-312 and A-366. Does not affect ability to promote DNA end resection at stalled replication forks; when associated with A-312., MUTAGEN:Y->G: Abolishes dNTPase activity; when associated with A-370., NP_BIND:GTP, NP_BIND:dNTP, REGION:Disordered, REGION:Substrate binding, | | |
| **SETD2** | [**SET domain containing 2, histone lysine methyltransferase(SETD2)**](https://david.ncifcrf.gov/geneReportFull.jsp?rowids=29072) | [**Related Genes**](https://david.ncifcrf.gov/relatedGenes.jsp?id=29072) | [**Homo sapiens**](http://www.ncbi.nlm.nih.gov/Taxonomy/Browser/wwwtax.cgi?name=Homo%20sapiens) |
| **GOTERM_BP_DIRECT** | [angiogenesis](http://www.ebi.ac.uk/QuickGO/GTerm?id=GO:0001525), [morphogenesis of a branching structure](http://www.ebi.ac.uk/QuickGO/GTerm?id=GO:0001763), [neural tube closure](http://www.ebi.ac.uk/QuickGO/GTerm?id=GO:0001843), [mismatch repair](http://www.ebi.ac.uk/QuickGO/GTerm?id=GO:0006298), [regulation of transcription, DNA-templated](http://www.ebi.ac.uk/QuickGO/GTerm?id=GO:0006355), [transcription elongation from RNA polymerase II promoter](http://www.ebi.ac.uk/QuickGO/GTerm?id=GO:0006368), [regulation of double-strand break repair via homologous recombination](http://www.ebi.ac.uk/QuickGO/GTerm?id=GO:0010569), [regulation of mRNA export from nucleus](http://www.ebi.ac.uk/QuickGO/GTerm?id=GO:0010793), [viral process](http://www.ebi.ac.uk/QuickGO/GTerm?id=GO:0016032), [peptidyl-lysine trimethylation](http://www.ebi.ac.uk/QuickGO/GTerm?id=GO:0018023), [peptidyl-lysine monomethylation](http://www.ebi.ac.uk/QuickGO/GTerm?id=GO:0018026), [forebrain development](http://www.ebi.ac.uk/QuickGO/GTerm?id=GO:0030900), [regulation of cytokinesis](http://www.ebi.ac.uk/QuickGO/GTerm?id=GO:0032465), [positive regulation of interferon-alpha production](http://www.ebi.ac.uk/QuickGO/GTerm?id=GO:0032727), [response to type I interferon](http://www.ebi.ac.uk/QuickGO/GTerm?id=GO:0034340), [nucleosome organization](http://www.ebi.ac.uk/QuickGO/GTerm?id=GO:0034728), [cell migration involved in vasculogenesis](http://www.ebi.ac.uk/QuickGO/GTerm?id=GO:0035441), [endodermal cell differentiation](http://www.ebi.ac.uk/QuickGO/GTerm?id=GO:0035987), [mesoderm morphogenesis](http://www.ebi.ac.uk/QuickGO/GTerm?id=GO:0048332), [embryonic cranial skeleton morphogenesis](http://www.ebi.ac.uk/QuickGO/GTerm?id=GO:0048701), [stem cell differentiation](http://www.ebi.ac.uk/QuickGO/GTerm?id=GO:0048863), [stem cell development](http://www.ebi.ac.uk/QuickGO/GTerm?id=GO:0048864), [defense response to virus](http://www.ebi.ac.uk/QuickGO/GTerm?id=GO:0051607), [pericardium development](http://www.ebi.ac.uk/QuickGO/GTerm?id=GO:0060039), [embryonic placenta morphogenesis](http://www.ebi.ac.uk/QuickGO/GTerm?id=GO:0060669), [coronary vasculature morphogenesis](http://www.ebi.ac.uk/QuickGO/GTerm?id=GO:0060977), [histone H3-K36 trimethylation](http://www.ebi.ac.uk/QuickGO/GTerm?id=GO:0097198), [histone H3-K36 dimethylation](http://www.ebi.ac.uk/QuickGO/GTerm?id=GO:0097676), [microtubule cytoskeleton organization involved in mitosis](http://www.ebi.ac.uk/QuickGO/GTerm?id=GO:1902850), [regulation of protein localization to chromatin](http://www.ebi.ac.uk/QuickGO/GTerm?id=GO:1905634), | | |
| **GOTERM_CC_DIRECT** | [nucleus](http://www.ebi.ac.uk/QuickGO/GTerm?id=GO:0005634), [nucleoplasm](http://www.ebi.ac.uk/QuickGO/GTerm?id=GO:0005654), [chromosome](http://www.ebi.ac.uk/QuickGO/GTerm?id=GO:0005694), | | |
| **GOTERM_MF_DIRECT** | [protein binding](http://www.ebi.ac.uk/QuickGO/GTerm?id=GO:0005515), [protein-lysine N-methyltransferase activity](http://www.ebi.ac.uk/QuickGO/GTerm?id=GO:0016279), [histone-lysine N-methyltransferase activity](http://www.ebi.ac.uk/QuickGO/GTerm?id=GO:0018024), [alpha-tubulin binding](http://www.ebi.ac.uk/QuickGO/GTerm?id=GO:0043014), [metal ion binding](http://www.ebi.ac.uk/QuickGO/GTerm?id=GO:0046872), [histone methyltransferase activity (H3-K36 specific)](http://www.ebi.ac.uk/QuickGO/GTerm?id=GO:0046975), | | |
| **INTERPRO** | [WW domain](https://www.ebi.ac.uk/interpro/entry/InterPro/IPR001202), [SET domain](https://www.ebi.ac.uk/interpro/entry/InterPro/IPR001214), [Post-SET domain](https://www.ebi.ac.uk/interpro/entry/InterPro/IPR003616), [AWS](https://www.ebi.ac.uk/interpro/entry/InterPro/IPR006560), [Ferritin-like superfamily](https://www.ebi.ac.uk/interpro/entry/InterPro/IPR009078), [SRI, Set2 Rpb1 interacting](https://www.ebi.ac.uk/interpro/entry/InterPro/IPR013257), | | |
| **KEGG_PATHWAY** | [Lysine degradation](https://david.ncifcrf.gov/kegg.jsp?path=hsa00310$Lysine%20degradation&termId=520047850&source=kegg), [Metabolic pathways](https://david.ncifcrf.gov/kegg.jsp?path=hsa01100$Metabolic%20pathways&termId=520047911&source=kegg), | | |
| **OMIM_DISEASE** | [Luscan-Lumish syndrome](http://omim.org/entry/616831), | | |
| **SMART** | [SET](http://smart.embl.de/smart/do_annotation.pl?DOMAIN=SM00317), [WW](http://smart.embl.de/smart/do_annotation.pl?DOMAIN=SM00456), [PostSET](http://smart.embl.de/smart/do_annotation.pl?DOMAIN=SM00508), [AWS](http://smart.embl.de/smart/do_annotation.pl?DOMAIN=SM00570), | | |
| **UP_KW_BIOLOGICAL_PROCESS** | [Antiviral defense](http://www.uniprot.org/keywords/?query=KW-0051), [Differentiation](http://www.uniprot.org/keywords/?query=KW-0221), [DNA damage](http://www.uniprot.org/keywords/?query=KW-0227), [DNA repair](http://www.uniprot.org/keywords/?query=KW-0234), [Immunity](http://www.uniprot.org/keywords/?query=KW-0391), [Innate immunity](http://www.uniprot.org/keywords/?query=KW-0399), [Transcription](http://www.uniprot.org/keywords/?query=KW-0804), [Transcription regulation](http://www.uniprot.org/keywords/?query=KW-0805), [Host-virus interaction](http://www.uniprot.org/keywords/?query=KW-0945), | | |
| **UP_KW_CELLULAR_COMPONENT** | [Chromosome](http://www.uniprot.org/keywords/?query=KW-0158), [Nucleus](http://www.uniprot.org/keywords/?query=KW-0539), | | |
| **UP_KW_DISEASE** | [Tumor suppressor](http://www.uniprot.org/keywords/?query=KW-0043), [Disease variant](http://www.uniprot.org/keywords/?query=KW-0225), [Mental retardation](http://www.uniprot.org/keywords/?query=KW-0991), [Autism spectrum disorder](http://www.uniprot.org/keywords/?query=KW-1268), | | |
| **UP_KW_DOMAIN** | [Coiled coil](http://www.uniprot.org/keywords/?query=KW-0175), | | |
| **UP_KW_LIGAND** | [Metal-binding](http://www.uniprot.org/keywords/?query=KW-0479), [Zinc](http://www.uniprot.org/keywords/?query=KW-0862), [S-adenosyl-L-methionine](http://www.uniprot.org/keywords/?query=KW-0949), | | |
| **UP_KW_MOLECULAR_FUNCTION** | [Activator](http://www.uniprot.org/keywords/?query=KW-0010), [Chromatin regulator](http://www.uniprot.org/keywords/?query=KW-0156), [Developmental protein](http://www.uniprot.org/keywords/?query=KW-0217), [Methyltransferase](http://www.uniprot.org/keywords/?query=KW-0489), [Transferase](http://www.uniprot.org/keywords/?query=KW-0808), [Developmental protein](http://www.uniprot.org/keywords/?query=KW-9996), | | |
| **UP_KW_PTM** | [Phosphoprotein](http://www.uniprot.org/keywords/?query=KW-0597), [Ubl conjugation](http://www.uniprot.org/keywords/?query=KW-0832), [Isopeptide bond](http://www.uniprot.org/keywords/?query=KW-1017), | | |
| **UP_SEQ_FEATURE** | BINDING:N-propyl sinefungin; inhibitor, BINDING:N-propyl sinefungin; inhibitor; via amide nitrogen, BINDING:S-adenosyl-L-methionine, BINDING:S-adenosyl-L-methionine; via amide nitrogen, COMPBIAS:Basic and acidic residues, COMPBIAS:Polar residues, COMPBIAS:Pro residues, CROSSLNK:Glycyl lysine isopeptide (Lys-Gly) (interchain with G-Cter in SUMO2), DOMAIN:AWS, DOMAIN:Post-SET, DOMAIN:SET, DOMAIN:WW, METAL:Zinc 1, METAL:Zinc 2, METAL:Zinc 3, MUTAGEN:C->A: Does not affect methyltransferase activity., MUTAGEN:E->A: Increased methyltransferase activity., MUTAGEN:E->A: Increases interaction with hyperphosphorylated POLR2A; when associated with A-2528., MUTAGEN:E->A: Increases interaction with hyperphosphorylated POLR2A; when associated with A-2531., MUTAGEN:F->A: Does not affect interaction with hyperphosphorylated POLR2A., MUTAGEN:F->A: Strongly reduced methyltransferase activity., MUTAGEN:G->A,T: Does not affect interaction with hyperphosphorylated POLR2A., MUTAGEN:H->A: Impairs interaction with hyperphosphorylated POLR2A., MUTAGEN:K->A: Does not affect interaction with hyperphosphorylated POLR2A., MUTAGEN:K->A: Impairs interaction with hyperphosphorylated POLR2A., MUTAGEN:Q->A: Does not affect interaction with hyperphosphorylated POLR2A., MUTAGEN:Q->A: Loss of methyltransferase activity., MUTAGEN:R->A,V,L,I,F: Impaired methyltransferase activity., MUTAGEN:R->A: Does not affect interaction with hyperphosphorylated POLR2A., MUTAGEN:R->A: Impairs interaction with hyperphosphorylated POLR2A., MUTAGEN:R->H,G: Loss of methyltransferase activity. Abolishes ability to monomethylate STAT1., MUTAGEN:R->P,W,K,Q: Loss of methyltransferase activity., MUTAGEN:T->A: Increased methyltransferase activity., MUTAGEN:V->A: Impairs interaction with hyperphosphorylated POLR2A., MUTAGEN:Y->A: Increased methyltransferase activity., MUTAGEN:Y->A: Strongly reduced methyltransferase activity., REGION:Disordered, REGION:Interaction with POLR2A, REGION:Interaction with TUBA1A, REGION:Low charge region, REGION:N-propyl sinefungin binding; inhibitor, REGION:S-adenosyl-L-methionine binding, | | |
| **SHANK2** | [**SH3 and multiple ankyrin repeat domains 2(SHANK2)**](https://david.ncifcrf.gov/geneReportFull.jsp?rowids=22941) | [**Related Genes**](https://david.ncifcrf.gov/relatedGenes.jsp?id=22941) | [**Homo sapiens**](http://www.ncbi.nlm.nih.gov/Taxonomy/Browser/wwwtax.cgi?name=Homo%20sapiens) |
| **GOTERM_BP_DIRECT** | [synapse assembly](http://www.ebi.ac.uk/QuickGO/GTerm?id=GO:0007416), [brain development](http://www.ebi.ac.uk/QuickGO/GTerm?id=GO:0007420), [response to nutrient](http://www.ebi.ac.uk/QuickGO/GTerm?id=GO:0007584), [learning](http://www.ebi.ac.uk/QuickGO/GTerm?id=GO:0007612), [positive regulation of cell proliferation](http://www.ebi.ac.uk/QuickGO/GTerm?id=GO:0008284), [associative learning](http://www.ebi.ac.uk/QuickGO/GTerm?id=GO:0008306), [adult locomotory behavior](http://www.ebi.ac.uk/QuickGO/GTerm?id=GO:0008344), [response to xenobiotic stimulus](http://www.ebi.ac.uk/QuickGO/GTerm?id=GO:0009410), [adult behavior](http://www.ebi.ac.uk/QuickGO/GTerm?id=GO:0030534), [social behavior](http://www.ebi.ac.uk/QuickGO/GTerm?id=GO:0035176), [negative regulation of hippo signaling](http://www.ebi.ac.uk/QuickGO/GTerm?id=GO:0035331), [brain morphogenesis](http://www.ebi.ac.uk/QuickGO/GTerm?id=GO:0048854), [regulation of synapse organization](http://www.ebi.ac.uk/QuickGO/GTerm?id=GO:0050807), [synaptic growth at neuromuscular junction](http://www.ebi.ac.uk/QuickGO/GTerm?id=GO:0051124), [positive regulation of synaptic transmission, glutamatergic](http://www.ebi.ac.uk/QuickGO/GTerm?id=GO:0051968), [long-term synaptic potentiation](http://www.ebi.ac.uk/QuickGO/GTerm?id=GO:0060291), [long term synaptic depression](http://www.ebi.ac.uk/QuickGO/GTerm?id=GO:0060292), [dendritic spine morphogenesis](http://www.ebi.ac.uk/QuickGO/GTerm?id=GO:0060997), [positive regulation of dendritic spine development](http://www.ebi.ac.uk/QuickGO/GTerm?id=GO:0060999), [vocalization behavior](http://www.ebi.ac.uk/QuickGO/GTerm?id=GO:0071625), [postsynaptic density assembly](http://www.ebi.ac.uk/QuickGO/GTerm?id=GO:0097107), [maintenance of postsynaptic density structure](http://www.ebi.ac.uk/QuickGO/GTerm?id=GO:0099562), [regulation of AMPA receptor activity](http://www.ebi.ac.uk/QuickGO/GTerm?id=GO:2000311), [positive regulation of excitatory postsynaptic potential](http://www.ebi.ac.uk/QuickGO/GTerm?id=GO:2000463), | | |
| **GOTERM_CC_DIRECT** | [photoreceptor outer segment](http://www.ebi.ac.uk/QuickGO/GTerm?id=GO:0001750), [photoreceptor inner segment](http://www.ebi.ac.uk/QuickGO/GTerm?id=GO:0001917), [cytosol](http://www.ebi.ac.uk/QuickGO/GTerm?id=GO:0005829), [neurofilament](http://www.ebi.ac.uk/QuickGO/GTerm?id=GO:0005883), [plasma membrane](http://www.ebi.ac.uk/QuickGO/GTerm?id=GO:0005886), [postsynaptic density](http://www.ebi.ac.uk/QuickGO/GTerm?id=GO:0014069), [integral component of membrane](http://www.ebi.ac.uk/QuickGO/GTerm?id=GO:0016021), [apical plasma membrane](http://www.ebi.ac.uk/QuickGO/GTerm?id=GO:0016324), [growth cone](http://www.ebi.ac.uk/QuickGO/GTerm?id=GO:0030426), [brush border membrane](http://www.ebi.ac.uk/QuickGO/GTerm?id=GO:0031526), [neuron projection](http://www.ebi.ac.uk/QuickGO/GTerm?id=GO:0043005), [neuronal cell body](http://www.ebi.ac.uk/QuickGO/GTerm?id=GO:0043025), [dendritic spine](http://www.ebi.ac.uk/QuickGO/GTerm?id=GO:0043197), [postsynaptic membrane](http://www.ebi.ac.uk/QuickGO/GTerm?id=GO:0045211), [presynaptic active zone](http://www.ebi.ac.uk/QuickGO/GTerm?id=GO:0048786), [ciliary membrane](http://www.ebi.ac.uk/QuickGO/GTerm?id=GO:0060170), [hippocampal mossy fiber to CA3 synapse](http://www.ebi.ac.uk/QuickGO/GTerm?id=GO:0098686), [glutamatergic synapse](http://www.ebi.ac.uk/QuickGO/GTerm?id=GO:0098978), | | |
| **GOTERM_MF_DIRECT** | [protein binding](http://www.ebi.ac.uk/QuickGO/GTerm?id=GO:0005515), [protein C-terminus binding](http://www.ebi.ac.uk/QuickGO/GTerm?id=GO:0008022), [SH3 domain binding](http://www.ebi.ac.uk/QuickGO/GTerm?id=GO:0017124), [GKAP/Homer scaffold activity](http://www.ebi.ac.uk/QuickGO/GTerm?id=GO:0030160), [ionotropic glutamate receptor binding](http://www.ebi.ac.uk/QuickGO/GTerm?id=GO:0035255), [structural constituent of postsynaptic density](http://www.ebi.ac.uk/QuickGO/GTerm?id=GO:0098919), | | |
| **INTERPRO** | [Src homology-3 domain](https://www.ebi.ac.uk/interpro/entry/InterPro/IPR001452), [PDZ domain](https://www.ebi.ac.uk/interpro/entry/InterPro/IPR001478), [Sterile alpha motif domain](https://www.ebi.ac.uk/interpro/entry/InterPro/IPR001660), [Sterile alpha motif/pointed domain](https://www.ebi.ac.uk/interpro/entry/InterPro/IPR013761), [Ankyrin repeat-containing domain](https://www.ebi.ac.uk/interpro/entry/InterPro/IPR020683), | | |
| **KEGG_PATHWAY** | [Glutamatergic synapse](https://david.ncifcrf.gov/kegg.jsp?path=hsa04724$Glutamatergic%20synapse&termId=520048032&source=kegg), | | |
| **OMIM_DISEASE** | [Autism susceptibility 17](http://omim.org/entry/613436), | | |
| **SMART** | [PDZ](http://smart.embl.de/smart/do_annotation.pl?DOMAIN=SM00228), [SH3](http://smart.embl.de/smart/do_annotation.pl?DOMAIN=SM00326), [SAM](http://smart.embl.de/smart/do_annotation.pl?DOMAIN=SM00454), | | |
| **UP_KW_CELLULAR_COMPONENT** | [Membrane](http://www.uniprot.org/keywords/?query=KW-0472), [Synapse](http://www.uniprot.org/keywords/?query=KW-0770), [Cytoplasm](http://www.uniprot.org/keywords/?query=KW-0963), [Cell junction](http://www.uniprot.org/keywords/?query=KW-0965), [Cell projection](http://www.uniprot.org/keywords/?query=KW-0966), [Cell membrane](http://www.uniprot.org/keywords/?query=KW-1003), | | |
| **UP_KW_DISEASE** | [Autism spectrum disorder](http://www.uniprot.org/keywords/?query=KW-1268), [Autism](http://www.uniprot.org/keywords/?query=KW-1269), | | |
| **UP_KW_DOMAIN** | [SH3 domain](http://www.uniprot.org/keywords/?query=KW-0728), [SH3-binding](http://www.uniprot.org/keywords/?query=KW-0729), [Transmembrane](http://www.uniprot.org/keywords/?query=KW-0812), [Transmembrane helix](http://www.uniprot.org/keywords/?query=KW-1133), | | |
| **UP_KW_PTM** | [Glycoprotein](http://www.uniprot.org/keywords/?query=KW-0325), [Phosphoprotein](http://www.uniprot.org/keywords/?query=KW-0597), | | |
| **UP_SEQ_FEATURE** | CARBOHYD:O-linked (GlcNAc) threonine, COMPBIAS:Basic and acidic residues, COMPBIAS:Polar residues, COMPBIAS:Pro residues, DOMAIN:PDZ, DOMAIN:SAM, DOMAIN:SH3, MOTIF:SH3-binding, REGION:Disordered, TRANSMEM:Helical, | | |
| **SH3BP5** | [**SH3 domain binding protein 5(SH3BP5)**](https://david.ncifcrf.gov/geneReportFull.jsp?rowids=9467) | [**Related Genes**](https://david.ncifcrf.gov/relatedGenes.jsp?id=9467) | [**Homo sapiens**](http://www.ncbi.nlm.nih.gov/Taxonomy/Browser/wwwtax.cgi?name=Homo%20sapiens) |
| **GOTERM_BP_DIRECT** | [signal transduction](http://www.ebi.ac.uk/QuickGO/GTerm?id=GO:0007165), [intracellular signal transduction](http://www.ebi.ac.uk/QuickGO/GTerm?id=GO:0035556), [negative regulation of protein tyrosine kinase activity](http://www.ebi.ac.uk/QuickGO/GTerm?id=GO:0061099), | | |
| **GOTERM_CC_DIRECT** | [nucleoplasm](http://www.ebi.ac.uk/QuickGO/GTerm?id=GO:0005654), [cytoplasm](http://www.ebi.ac.uk/QuickGO/GTerm?id=GO:0005737), [mitochondrion](http://www.ebi.ac.uk/QuickGO/GTerm?id=GO:0005739), [nuclear body](http://www.ebi.ac.uk/QuickGO/GTerm?id=GO:0016604), [cytoplasmic vesicle membrane](http://www.ebi.ac.uk/QuickGO/GTerm?id=GO:0030659), | | |
| **GOTERM_MF_DIRECT** | [protein kinase inhibitor activity](http://www.ebi.ac.uk/QuickGO/GTerm?id=GO:0004860), [guanyl-nucleotide exchange factor activity](http://www.ebi.ac.uk/QuickGO/GTerm?id=GO:0005085), [protein binding](http://www.ebi.ac.uk/QuickGO/GTerm?id=GO:0005515), [SH3 domain binding](http://www.ebi.ac.uk/QuickGO/GTerm?id=GO:0017124), | | |
| **INTERPRO** | [SH3-binding 5](https://www.ebi.ac.uk/interpro/entry/InterPro/IPR007940), | | |
| **UP_KW_CELLULAR_COMPONENT** | [Membrane](http://www.uniprot.org/keywords/?query=KW-0472), [Mitochondrion](http://www.uniprot.org/keywords/?query=KW-0496), [Cytoplasmic vesicle](http://www.uniprot.org/keywords/?query=KW-0968), | | |
| **UP_KW_DOMAIN** | [Coiled coil](http://www.uniprot.org/keywords/?query=KW-0175), [SH3-binding](http://www.uniprot.org/keywords/?query=KW-0729), | | |
| **UP_KW_MOLECULAR_FUNCTION** | [Guanine-nucleotide releasing factor](http://www.uniprot.org/keywords/?query=KW-0344), | | |
| **UP_KW_PTM** | [Phosphoprotein](http://www.uniprot.org/keywords/?query=KW-0597), | | |
| **UP_SEQ_FEATURE** | COMPBIAS:Acidic residues, COMPBIAS:Basic and acidic residues, COMPBIAS:Polar residues, MUTAGEN:L->A: Loss of phosphorylation and binding by phospho-JNK; when associated with A-347., MUTAGEN:L->A: Loss of phosphorylation and binding by phospho-JNK; when associated with A-349., MUTAGEN:L->A: No change of phosphorylation or binding by phospho-JNK; when associated with A-434., MUTAGEN:L->A: No change of phosphorylation or binding by phospho-JNK; when associated with A-436., MUTAGEN:LE->AK: Loss of guanine nucleotide exchange factor activity., MUTAGEN:LNQ->AAA: Loss of guanine nucleotide exchange factor activity., REGION:Disordered, REGION:Sufficient for interaction with RAB11A and for guanine nucleotide exchange activity, | | |
| **SLAIN1** | [**SLAIN motif family member 1(SLAIN1)**](https://david.ncifcrf.gov/geneReportFull.jsp?rowids=122060) | [**Related Genes**](https://david.ncifcrf.gov/relatedGenes.jsp?id=122060) | [**Homo sapiens**](http://www.ncbi.nlm.nih.gov/Taxonomy/Browser/wwwtax.cgi?name=Homo%20sapiens) |
| **GOTERM_BP_DIRECT** | [microtubule nucleation](http://www.ebi.ac.uk/QuickGO/GTerm?id=GO:0007020), [positive regulation of microtubule polymerization](http://www.ebi.ac.uk/QuickGO/GTerm?id=GO:0031116), [cytoplasmic microtubule organization](http://www.ebi.ac.uk/QuickGO/GTerm?id=GO:0031122), | | |
| **GOTERM_CC_DIRECT** | [cytoplasm](http://www.ebi.ac.uk/QuickGO/GTerm?id=GO:0005737), [cytoskeleton](http://www.ebi.ac.uk/QuickGO/GTerm?id=GO:0005856), [microtubule plus-end](http://www.ebi.ac.uk/QuickGO/GTerm?id=GO:0035371), | | |
| **GOTERM_MF_DIRECT** | [protein binding](http://www.ebi.ac.uk/QuickGO/GTerm?id=GO:0005515), | | |
| **INTERPRO** | [SLAIN motif-containing protein](https://www.ebi.ac.uk/interpro/entry/InterPro/IPR026179), | | |
| **UP_KW_CELLULAR_COMPONENT** | [Cytoskeleton](http://www.uniprot.org/keywords/?query=KW-0206), [Cytoplasm](http://www.uniprot.org/keywords/?query=KW-0963), | | |
| **UP_KW_DOMAIN** | [Coiled coil](http://www.uniprot.org/keywords/?query=KW-0175), | | |
| **UP_KW_PTM** | [Methylation](http://www.uniprot.org/keywords/?query=KW-0488), [Phosphoprotein](http://www.uniprot.org/keywords/?query=KW-0597), | | |
| **UP_SEQ_FEATURE** | COMPBIAS:Polar residues, COMPBIAS:Pro residues, REGION:Disordered, | | |
| **SP110** | [**SP110 nuclear body protein(SP110)**](https://david.ncifcrf.gov/geneReportFull.jsp?rowids=3431) | [**Related Genes**](https://david.ncifcrf.gov/relatedGenes.jsp?id=3431) | [**Homo sapiens**](http://www.ncbi.nlm.nih.gov/Taxonomy/Browser/wwwtax.cgi?name=Homo%20sapiens) |
| **GOTERM_BP_DIRECT** | [regulation of transcription from RNA polymerase II promoter](http://www.ebi.ac.uk/QuickGO/GTerm?id=GO:0006357), [viral process](http://www.ebi.ac.uk/QuickGO/GTerm?id=GO:0016032), | | |
| **GOTERM_CC_DIRECT** | [nucleus](http://www.ebi.ac.uk/QuickGO/GTerm?id=GO:0005634), [nucleoplasm](http://www.ebi.ac.uk/QuickGO/GTerm?id=GO:0005654), | | |
| **GOTERM_MF_DIRECT** | [RNA polymerase II transcription factor activity, sequence-specific DNA binding](http://www.ebi.ac.uk/QuickGO/GTerm?id=GO:0000981), [DNA binding](http://www.ebi.ac.uk/QuickGO/GTerm?id=GO:0003677), [metal ion binding](http://www.ebi.ac.uk/QuickGO/GTerm?id=GO:0046872), | | |
| **INTERPRO** | [SAND domain](https://www.ebi.ac.uk/interpro/entry/InterPro/IPR000770), [Bromodomain](https://www.ebi.ac.uk/interpro/entry/InterPro/IPR001487), [Zinc finger, PHD-type](https://www.ebi.ac.uk/interpro/entry/InterPro/IPR001965), [Sp100](https://www.ebi.ac.uk/interpro/entry/InterPro/IPR004865), [SAND domain-like](https://www.ebi.ac.uk/interpro/entry/InterPro/IPR010919), [Zinc finger, FYVE/PHD-type](https://www.ebi.ac.uk/interpro/entry/InterPro/IPR011011), [Zinc finger, PHD-type, conserved site](https://www.ebi.ac.uk/interpro/entry/InterPro/IPR019786), [Zinc finger, PHD-finger](https://www.ebi.ac.uk/interpro/entry/InterPro/IPR019787), | | |
| **OMIM_DISEASE** | [Hepatic venoocclusive disease with immunodeficiency](http://omim.org/entry/235550), [Mycobacterium tuberculosis, susceptibility to](http://omim.org/entry/607948), | | |
| **SMART** | [PHD](http://smart.embl.de/smart/do_annotation.pl?DOMAIN=SM00249), [SAND](http://smart.embl.de/smart/do_annotation.pl?DOMAIN=SM00258), [BROMO](http://smart.embl.de/smart/do_annotation.pl?DOMAIN=SM00297), | | |
| **UP_KW_BIOLOGICAL_PROCESS** | [Transcription](http://www.uniprot.org/keywords/?query=KW-0804), [Transcription regulation](http://www.uniprot.org/keywords/?query=KW-0805), [Host-virus interaction](http://www.uniprot.org/keywords/?query=KW-0945), | | |
| **UP_KW_CELLULAR_COMPONENT** | [Nucleus](http://www.uniprot.org/keywords/?query=KW-0539), | | |
| **UP_KW_DOMAIN** | [Bromodomain](http://www.uniprot.org/keywords/?query=KW-0103), [Zinc-finger](http://www.uniprot.org/keywords/?query=KW-0863), | | |
| **UP_KW_LIGAND** | [Metal-binding](http://www.uniprot.org/keywords/?query=KW-0479), [Zinc](http://www.uniprot.org/keywords/?query=KW-0862), | | |
| **UP_KW_MOLECULAR_FUNCTION** | [DNA-binding](http://www.uniprot.org/keywords/?query=KW-0238), | | |
| **UP_KW_PTM** | [Phosphoprotein](http://www.uniprot.org/keywords/?query=KW-0597), | | |
| **UP_SEQ_FEATURE** | COMPBIAS:Basic and acidic residues, COMPBIAS:Basic residues, COMPBIAS:Polar residues, COMPBIAS:Pro residues, DOMAIN:Bromo, DOMAIN:HSR, DOMAIN:SAND, MOTIF:Nuclear localization signal, REGION:Disordered, REGION:Nuclear hormone receptor interaction, ZN_FING:PHD-type, | | |
| **SRC** | [**SRC proto-oncogene, non-receptor tyrosine kinase(SRC)**](https://david.ncifcrf.gov/geneReportFull.jsp?rowids=6714) | [**Related Genes**](https://david.ncifcrf.gov/relatedGenes.jsp?id=6714) | [**Homo sapiens**](http://www.ncbi.nlm.nih.gov/Taxonomy/Browser/wwwtax.cgi?name=Homo%20sapiens) |
| **BBID** | 85.Nuc_Rec-Coact_Complex, | | |
| **BIOCARTA** | [Role of nicotinic acetylcholine receptors in the regulation of apoptosis](https://david.ncifcrf.gov/biocarta.jsp?path=h_achPathway$Role%20of%20nicotinic%20acetylcholine%20receptors%20in%20the%20regulation%20of%20apoptosis&termId=30000005&source=biocarta), [Agrin in Postsynaptic Differentiation](https://david.ncifcrf.gov/biocarta.jsp?path=h_agrPathway$Agrin%20in%20Postsynaptic%20Differentiation&termId=30000009&source=biocarta), [Angiotensin II mediated activation of JNK Pathway via Pyk2 dependent signaling](https://david.ncifcrf.gov/biocarta.jsp?path=h_at1rPathway$Angiotensin%20II%20mediated%20activation%20of%20JNK%20Pathway%20via%20Pyk2%20dependent%20signaling&termId=30000028&source=biocarta), [Roles of ?-arrestin-dependent Recruitment of Src Kinases in GPCR Signaling](https://david.ncifcrf.gov/biocarta.jsp?path=h_bArrestin-srcPathway$Roles%20of%20?-arrestin-dependent%20Recruitment%20of%20Src%20Kinases%20in%20GPCR%20Signaling&termId=30000034&source=biocarta), [CBL mediated ligand-induced downregulation of EGF receptors](https://david.ncifcrf.gov/biocarta.jsp?path=h_cblPathway$CBL%20mediated%20ligand-induced%20downregulation%20of%20EGF%20receptors&termId=30000051&source=biocarta), [Cell to Cell Adhesion Signaling](https://david.ncifcrf.gov/biocarta.jsp?path=h_cell2cellPathway$Cell%20to%20Cell%20Adhesion%20Signaling&termId=30000059&source=biocarta), [Erk and PI-3 Kinase Are Necessary for Collagen Binding in Corneal Epithelia](https://david.ncifcrf.gov/biocarta.jsp?path=h_ecmPathway$Erk%20and%20PI-3%20Kinase%20Are%20Necessary%20for%20Collagen%20Binding%20in%20Corneal%20Epithelia&termId=30000088&source=biocarta), [Phospholipids as signalling intermediaries](https://david.ncifcrf.gov/biocarta.jsp?path=h_edg1Pathway$Phospholipids%20as%20signalling%20intermediaries&termId=30000089&source=biocarta), [Erk1/Erk2 Mapk Signaling pathway](https://david.ncifcrf.gov/biocarta.jsp?path=h_erkPathway$Erk1/Erk2%20Mapk%20Signaling%20pathway&termId=30000106&source=biocarta), [Gamma-aminobutyric Acid Receptor Life Cycle](https://david.ncifcrf.gov/biocarta.jsp?path=h_gabaPathway$Gamma-aminobutyric%20Acid%20Receptor%20Life%20Cycle&termId=30000123&source=biocarta), [Calcium Signaling by HBx of Hepatitis B virus](https://david.ncifcrf.gov/biocarta.jsp?path=h_hbxPathway$Calcium%20Signaling%20by%20HBx%20of%20Hepatitis%20B%20virus&termId=30000134&source=biocarta), [Integrin Signaling Pathway](https://david.ncifcrf.gov/biocarta.jsp?path=h_integrinPathway$Integrin%20Signaling%20Pathway&termId=30000165&source=biocarta), [Signaling of Hepatocyte Growth Factor Receptor](https://david.ncifcrf.gov/biocarta.jsp?path=h_metPathway$Signaling%20of%20Hepatocyte%20Growth%20Factor%20Receptor&termId=30000187&source=biocarta), [How Progesterone Initiates the Oocyte Maturation](https://david.ncifcrf.gov/biocarta.jsp?path=h_mPRPathway$How%20Progesterone%20Initiates%20the%20Oocyte%20Maturation&termId=30000192&source=biocarta), [Pelp1 Modulation of Estrogen Receptor Activity](https://david.ncifcrf.gov/biocarta.jsp?path=h_pelp1Pathway$Pelp1%20Modulation%20of%20Estrogen%20Receptor%20Activity&termId=30000225&source=biocarta), [Links between Pyk2 and Map Kinases](https://david.ncifcrf.gov/biocarta.jsp?path=h_pyk2Pathway$Links%20between%20Pyk2%20and%20Map%20Kinases&termId=30000245&source=biocarta), [Rho cell motility signaling pathway](https://david.ncifcrf.gov/biocarta.jsp?path=h_rhoPathway$Rho%20cell%20motility%20signaling%20pathway&termId=30000262&source=biocarta), [Regulation of Splicing through Sam68](https://david.ncifcrf.gov/biocarta.jsp?path=h_sam68Pathway$Regulation%20of%20Splicing%20through%20Sam68&termId=30000267&source=biocarta), [Aspirin Blocks Signaling Pathway Involved in Platelet Activation](https://david.ncifcrf.gov/biocarta.jsp?path=h_sppaPathway$Aspirin%20Blocks%20Signaling%20Pathway%20Involved%20in%20Platelet%20Activation&termId=30000276&source=biocarta), [Sprouty regulation of tyrosine kinase signals](https://david.ncifcrf.gov/biocarta.jsp?path=h_spryPathway$Sprouty%20regulation%20of%20tyrosine%20kinase%20signals&termId=30000277&source=biocarta), [Activation of Src by Protein-tyrosine phosphatase alpha](https://david.ncifcrf.gov/biocarta.jsp?path=h_srcRPTPPathway$Activation%20of%20Src%20by%20Protein-tyrosine%20phosphatase%20alpha&termId=30000278&source=biocarta), [uCalpain and friends in Cell spread](https://david.ncifcrf.gov/biocarta.jsp?path=h_uCalpainPathway$uCalpain%20and%20friends%20in%20Cell%20spread&termId=30000306&source=biocarta), | | |
| **GOTERM_BP_DIRECT** | [primary ovarian follicle growth](http://www.ebi.ac.uk/QuickGO/GTerm?id=GO:0001545), [positive regulation of cytokine production](http://www.ebi.ac.uk/QuickGO/GTerm?id=GO:0001819), [stimulatory C-type lectin receptor signaling pathway](http://www.ebi.ac.uk/QuickGO/GTerm?id=GO:0002223), [negative regulation of inflammatory response to antigenic stimulus](http://www.ebi.ac.uk/QuickGO/GTerm?id=GO:0002862), [cell cycle](http://www.ebi.ac.uk/QuickGO/GTerm?id=GO:0007049), [cell adhesion](http://www.ebi.ac.uk/QuickGO/GTerm?id=GO:0007155), [signal transduction](http://www.ebi.ac.uk/QuickGO/GTerm?id=GO:0007165), [transmembrane receptor protein tyrosine kinase signaling pathway](http://www.ebi.ac.uk/QuickGO/GTerm?id=GO:0007169), [signal complex assembly](http://www.ebi.ac.uk/QuickGO/GTerm?id=GO:0007172), [epidermal growth factor receptor signaling pathway](http://www.ebi.ac.uk/QuickGO/GTerm?id=GO:0007173), [transforming growth factor beta receptor signaling pathway](http://www.ebi.ac.uk/QuickGO/GTerm?id=GO:0007179), [G-protein coupled receptor signaling pathway](http://www.ebi.ac.uk/QuickGO/GTerm?id=GO:0007186), [integrin-mediated signaling pathway](http://www.ebi.ac.uk/QuickGO/GTerm?id=GO:0007229), [axon guidance](http://www.ebi.ac.uk/QuickGO/GTerm?id=GO:0007411), [cell proliferation](http://www.ebi.ac.uk/QuickGO/GTerm?id=GO:0008283), [response to xenobiotic stimulus](http://www.ebi.ac.uk/QuickGO/GTerm?id=GO:0009410), [response to mechanical stimulus](http://www.ebi.ac.uk/QuickGO/GTerm?id=GO:0009612), [response to virus](http://www.ebi.ac.uk/QuickGO/GTerm?id=GO:0009615), [response to acidic pH](http://www.ebi.ac.uk/QuickGO/GTerm?id=GO:0010447), [regulation of epithelial cell migration](http://www.ebi.ac.uk/QuickGO/GTerm?id=GO:0010632), [positive regulation of epithelial cell migration](http://www.ebi.ac.uk/QuickGO/GTerm?id=GO:0010634), [positive regulation of glucose metabolic process](http://www.ebi.ac.uk/QuickGO/GTerm?id=GO:0010907), [positive regulation of protein processing](http://www.ebi.ac.uk/QuickGO/GTerm?id=GO:0010954), [positive regulation of phosphatidylinositol 3-kinase signaling](http://www.ebi.ac.uk/QuickGO/GTerm?id=GO:0014068), [positive regulation of smooth muscle cell migration](http://www.ebi.ac.uk/QuickGO/GTerm?id=GO:0014911), [viral process](http://www.ebi.ac.uk/QuickGO/GTerm?id=GO:0016032), [macroautophagy](http://www.ebi.ac.uk/QuickGO/GTerm?id=GO:0016236), [peptidyl-serine phosphorylation](http://www.ebi.ac.uk/QuickGO/GTerm?id=GO:0018105), [peptidyl-tyrosine phosphorylation](http://www.ebi.ac.uk/QuickGO/GTerm?id=GO:0018108), [regulation of cell-cell adhesion](http://www.ebi.ac.uk/QuickGO/GTerm?id=GO:0022407), [cell differentiation](http://www.ebi.ac.uk/QuickGO/GTerm?id=GO:0030154), [platelet activation](http://www.ebi.ac.uk/QuickGO/GTerm?id=GO:0030168), [forebrain development](http://www.ebi.ac.uk/QuickGO/GTerm?id=GO:0030900), [T cell costimulation](http://www.ebi.ac.uk/QuickGO/GTerm?id=GO:0031295), [negative regulation of protein complex assembly](http://www.ebi.ac.uk/QuickGO/GTerm?id=GO:0031333), [protein destabilization](http://www.ebi.ac.uk/QuickGO/GTerm?id=GO:0031648), [response to nutrient levels](http://www.ebi.ac.uk/QuickGO/GTerm?id=GO:0031667), [positive regulation of protein autophosphorylation](http://www.ebi.ac.uk/QuickGO/GTerm?id=GO:0031954), [activation of protein kinase B activity](http://www.ebi.ac.uk/QuickGO/GTerm?id=GO:0032148), [negative regulation of telomere maintenance via telomerase](http://www.ebi.ac.uk/QuickGO/GTerm?id=GO:0032211), [cellular response to insulin stimulus](http://www.ebi.ac.uk/QuickGO/GTerm?id=GO:0032869), [regulation of intracellular estrogen receptor signaling pathway](http://www.ebi.ac.uk/QuickGO/GTerm?id=GO:0033146), [positive regulation of integrin activation](http://www.ebi.ac.uk/QuickGO/GTerm?id=GO:0033625), [adherens junction organization](http://www.ebi.ac.uk/QuickGO/GTerm?id=GO:0034332), [substrate adhesion-dependent cell spreading](http://www.ebi.ac.uk/QuickGO/GTerm?id=GO:0034446), [positive regulation of dephosphorylation](http://www.ebi.ac.uk/QuickGO/GTerm?id=GO:0035306), [intracellular signal transduction](http://www.ebi.ac.uk/QuickGO/GTerm?id=GO:0035556), [entry of bacterium into host cell](http://www.ebi.ac.uk/QuickGO/GTerm?id=GO:0035635), [osteoclast development](http://www.ebi.ac.uk/QuickGO/GTerm?id=GO:0036035), [cellular response to platelet-derived growth factor stimulus](http://www.ebi.ac.uk/QuickGO/GTerm?id=GO:0036120), [peptidyl-tyrosine autophosphorylation](http://www.ebi.ac.uk/QuickGO/GTerm?id=GO:0038083), [Fc-gamma receptor signaling pathway involved in phagocytosis](http://www.ebi.ac.uk/QuickGO/GTerm?id=GO:0038096), [ERBB2 signaling pathway](http://www.ebi.ac.uk/QuickGO/GTerm?id=GO:0038128), [odontogenesis](http://www.ebi.ac.uk/QuickGO/GTerm?id=GO:0042476), [response to drug](http://www.ebi.ac.uk/QuickGO/GTerm?id=GO:0042493), [positive regulation of apoptotic process](http://www.ebi.ac.uk/QuickGO/GTerm?id=GO:0043065), [negative regulation of apoptotic process](http://www.ebi.ac.uk/QuickGO/GTerm?id=GO:0043066), [regulation of vascular permeability](http://www.ebi.ac.uk/QuickGO/GTerm?id=GO:0043114), [stress fiber assembly](http://www.ebi.ac.uk/QuickGO/GTerm?id=GO:0043149), [negative regulation of cysteine-type endopeptidase activity involved in apoptotic process](http://www.ebi.ac.uk/QuickGO/GTerm?id=GO:0043154), [regulation of protein binding](http://www.ebi.ac.uk/QuickGO/GTerm?id=GO:0043393), [positive regulation of MAP kinase activity](http://www.ebi.ac.uk/QuickGO/GTerm?id=GO:0043406), [positive regulation of phosphatidylinositol 3-kinase activity](http://www.ebi.ac.uk/QuickGO/GTerm?id=GO:0043552), [transcytosis](http://www.ebi.ac.uk/QuickGO/GTerm?id=GO:0045056), [innate immune response](http://www.ebi.ac.uk/QuickGO/GTerm?id=GO:0045087), [regulation of bone resorption](http://www.ebi.ac.uk/QuickGO/GTerm?id=GO:0045124), [bone resorption](http://www.ebi.ac.uk/QuickGO/GTerm?id=GO:0045453), [positive regulation of cyclin-dependent protein serine/threonine kinase activity](http://www.ebi.ac.uk/QuickGO/GTerm?id=GO:0045737), [positive regulation of Notch signaling pathway](http://www.ebi.ac.uk/QuickGO/GTerm?id=GO:0045747), [negative regulation of transcription, DNA-templated](http://www.ebi.ac.uk/QuickGO/GTerm?id=GO:0045892), [positive regulation of transcription, DNA-templated](http://www.ebi.ac.uk/QuickGO/GTerm?id=GO:0045893), [positive regulation of insulin receptor signaling pathway](http://www.ebi.ac.uk/QuickGO/GTerm?id=GO:0046628), [protein autophosphorylation](http://www.ebi.ac.uk/QuickGO/GTerm?id=GO:0046777), [vascular endothelial growth factor receptor signaling pathway](http://www.ebi.ac.uk/QuickGO/GTerm?id=GO:0048010), [neurotrophin TRK receptor signaling pathway](http://www.ebi.ac.uk/QuickGO/GTerm?id=GO:0048011), [ephrin receptor signaling pathway](http://www.ebi.ac.uk/QuickGO/GTerm?id=GO:0048013), [focal adhesion assembly](http://www.ebi.ac.uk/QuickGO/GTerm?id=GO:0048041), [oogenesis](http://www.ebi.ac.uk/QuickGO/GTerm?id=GO:0048477), [positive regulation of peptidyl-tyrosine phosphorylation](http://www.ebi.ac.uk/QuickGO/GTerm?id=GO:0050731), [progesterone receptor signaling pathway](http://www.ebi.ac.uk/QuickGO/GTerm?id=GO:0050847), [leukocyte migration](http://www.ebi.ac.uk/QuickGO/GTerm?id=GO:0050900), [positive regulation of small GTPase mediated signal transduction](http://www.ebi.ac.uk/QuickGO/GTerm?id=GO:0051057), [positive regulation of protein transport](http://www.ebi.ac.uk/QuickGO/GTerm?id=GO:0051222), [response to mineralocorticoid](http://www.ebi.ac.uk/QuickGO/GTerm?id=GO:0051385), [response to electrical stimulus](http://www.ebi.ac.uk/QuickGO/GTerm?id=GO:0051602), [negative regulation of focal adhesion assembly](http://www.ebi.ac.uk/QuickGO/GTerm?id=GO:0051895), [positive regulation of protein kinase B signaling](http://www.ebi.ac.uk/QuickGO/GTerm?id=GO:0051897), [negative regulation of mitochondrial depolarization](http://www.ebi.ac.uk/QuickGO/GTerm?id=GO:0051902), [negative regulation of telomerase activity](http://www.ebi.ac.uk/QuickGO/GTerm?id=GO:0051974), [uterus development](http://www.ebi.ac.uk/QuickGO/GTerm?id=GO:0060065), [branching involved in mammary gland duct morphogenesis](http://www.ebi.ac.uk/QuickGO/GTerm?id=GO:0060444), [regulation of cell projection assembly](http://www.ebi.ac.uk/QuickGO/GTerm?id=GO:0060491), [intestinal epithelial cell development](http://www.ebi.ac.uk/QuickGO/GTerm?id=GO:0060576), [interleukin-6-mediated signaling pathway](http://www.ebi.ac.uk/QuickGO/GTerm?id=GO:0070102), [cellular response to hydrogen peroxide](http://www.ebi.ac.uk/QuickGO/GTerm?id=GO:0070301), [positive regulation of ERK1 and ERK2 cascade](http://www.ebi.ac.uk/QuickGO/GTerm?id=GO:0070374), [response to interleukin-1](http://www.ebi.ac.uk/QuickGO/GTerm?id=GO:0070555), [cellular response to lipopolysaccharide](http://www.ebi.ac.uk/QuickGO/GTerm?id=GO:0071222), [cellular response to peptide hormone stimulus](http://www.ebi.ac.uk/QuickGO/GTerm?id=GO:0071375), [cellular response to progesterone stimulus](http://www.ebi.ac.uk/QuickGO/GTerm?id=GO:0071393), [cellular response to fatty acid](http://www.ebi.ac.uk/QuickGO/GTerm?id=GO:0071398), [cellular response to hypoxia](http://www.ebi.ac.uk/QuickGO/GTerm?id=GO:0071456), [cellular response to fluid shear stress](http://www.ebi.ac.uk/QuickGO/GTerm?id=GO:0071498), [positive regulation of podosome assembly](http://www.ebi.ac.uk/QuickGO/GTerm?id=GO:0071803), [positive regulation of protein serine/threonine kinase activity](http://www.ebi.ac.uk/QuickGO/GTerm?id=GO:0071902), [angiotensin-activated signaling pathway involved in heart process](http://www.ebi.ac.uk/QuickGO/GTerm?id=GO:0086098), [positive regulation of canonical Wnt signaling pathway](http://www.ebi.ac.uk/QuickGO/GTerm?id=GO:0090263), [cell-cell adhesion](http://www.ebi.ac.uk/QuickGO/GTerm?id=GO:0098609), [regulation of postsynaptic neurotransmitter receptor activity](http://www.ebi.ac.uk/QuickGO/GTerm?id=GO:0098962), [positive regulation of protein localization to nucleus](http://www.ebi.ac.uk/QuickGO/GTerm?id=GO:1900182), [positive regulation of non-membrane spanning protein tyrosine kinase activity](http://www.ebi.ac.uk/QuickGO/GTerm?id=GO:1903997), [positive regulation of ovarian follicle development](http://www.ebi.ac.uk/QuickGO/GTerm?id=GO:2000386), [positive regulation of lamellipodium morphogenesis](http://www.ebi.ac.uk/QuickGO/GTerm?id=GO:2000394), [positive regulation of DNA biosynthetic process](http://www.ebi.ac.uk/QuickGO/GTerm?id=GO:2000573), [positive regulation of platelet-derived growth factor receptor-beta signaling pathway](http://www.ebi.ac.uk/QuickGO/GTerm?id=GO:2000588), [regulation of early endosome to late endosome transport](http://www.ebi.ac.uk/QuickGO/GTerm?id=GO:2000641), [negative regulation of anoikis](http://www.ebi.ac.uk/QuickGO/GTerm?id=GO:2000811), [negative regulation of extrinsic apoptotic signaling pathway](http://www.ebi.ac.uk/QuickGO/GTerm?id=GO:2001237), [negative regulation of intrinsic apoptotic signaling pathway](http://www.ebi.ac.uk/QuickGO/GTerm?id=GO:2001243), [regulation of caveolin-mediated endocytosis](http://www.ebi.ac.uk/QuickGO/GTerm?id=GO:2001286), | | |
| **GOTERM_CC_DIRECT** | [podosome](http://www.ebi.ac.uk/QuickGO/GTerm?id=GO:0002102), [nucleoplasm](http://www.ebi.ac.uk/QuickGO/GTerm?id=GO:0005654), [cytoplasm](http://www.ebi.ac.uk/QuickGO/GTerm?id=GO:0005737), [mitochondrion](http://www.ebi.ac.uk/QuickGO/GTerm?id=GO:0005739), [mitochondrial inner membrane](http://www.ebi.ac.uk/QuickGO/GTerm?id=GO:0005743), [lysosome](http://www.ebi.ac.uk/QuickGO/GTerm?id=GO:0005764), [late endosome](http://www.ebi.ac.uk/QuickGO/GTerm?id=GO:0005770), [cytosol](http://www.ebi.ac.uk/QuickGO/GTerm?id=GO:0005829), [actin filament](http://www.ebi.ac.uk/QuickGO/GTerm?id=GO:0005884), [plasma membrane](http://www.ebi.ac.uk/QuickGO/GTerm?id=GO:0005886), [caveola](http://www.ebi.ac.uk/QuickGO/GTerm?id=GO:0005901), [focal adhesion](http://www.ebi.ac.uk/QuickGO/GTerm?id=GO:0005925), [postsynaptic density](http://www.ebi.ac.uk/QuickGO/GTerm?id=GO:0014069), [cell junction](http://www.ebi.ac.uk/QuickGO/GTerm?id=GO:0030054), [extrinsic component of cytoplasmic side of plasma membrane](http://www.ebi.ac.uk/QuickGO/GTerm?id=GO:0031234), [ruffle membrane](http://www.ebi.ac.uk/QuickGO/GTerm?id=GO:0032587), [neuron projection](http://www.ebi.ac.uk/QuickGO/GTerm?id=GO:0043005), [membrane raft](http://www.ebi.ac.uk/QuickGO/GTerm?id=GO:0045121), [perinuclear region of cytoplasm](http://www.ebi.ac.uk/QuickGO/GTerm?id=GO:0048471), [extracellular exosome](http://www.ebi.ac.uk/QuickGO/GTerm?id=GO:0070062), [glutamatergic synapse](http://www.ebi.ac.uk/QuickGO/GTerm?id=GO:0098978), [postsynaptic specialization, intracellular component](http://www.ebi.ac.uk/QuickGO/GTerm?id=GO:0099091), | | |
| **GOTERM_MF_DIRECT** | [protein kinase activity](http://www.ebi.ac.uk/QuickGO/GTerm?id=GO:0004672), [protein serine/threonine/tyrosine kinase activity](http://www.ebi.ac.uk/QuickGO/GTerm?id=GO:0004712), [protein tyrosine kinase activity](http://www.ebi.ac.uk/QuickGO/GTerm?id=GO:0004713), [transmembrane receptor protein tyrosine kinase activity](http://www.ebi.ac.uk/QuickGO/GTerm?id=GO:0004714), [non-membrane spanning protein tyrosine kinase activity](http://www.ebi.ac.uk/QuickGO/GTerm?id=GO:0004715), [protein kinase C binding](http://www.ebi.ac.uk/QuickGO/GTerm?id=GO:0005080), [receptor binding](http://www.ebi.ac.uk/QuickGO/GTerm?id=GO:0005102), [insulin receptor binding](http://www.ebi.ac.uk/QuickGO/GTerm?id=GO:0005158), [integrin binding](http://www.ebi.ac.uk/QuickGO/GTerm?id=GO:0005178), [protein binding](http://www.ebi.ac.uk/QuickGO/GTerm?id=GO:0005515), [ATP binding](http://www.ebi.ac.uk/QuickGO/GTerm?id=GO:0005524), [protein C-terminus binding](http://www.ebi.ac.uk/QuickGO/GTerm?id=GO:0008022), [phospholipase activator activity](http://www.ebi.ac.uk/QuickGO/GTerm?id=GO:0016004), [enzyme binding](http://www.ebi.ac.uk/QuickGO/GTerm?id=GO:0019899), [kinase binding](http://www.ebi.ac.uk/QuickGO/GTerm?id=GO:0019900), [heme binding](http://www.ebi.ac.uk/QuickGO/GTerm?id=GO:0020037), [estrogen receptor binding](http://www.ebi.ac.uk/QuickGO/GTerm?id=GO:0030331), [ubiquitin protein ligase binding](http://www.ebi.ac.uk/QuickGO/GTerm?id=GO:0031625), [SH2 domain binding](http://www.ebi.ac.uk/QuickGO/GTerm?id=GO:0042169), [phospholipase binding](http://www.ebi.ac.uk/QuickGO/GTerm?id=GO:0043274), [ion channel binding](http://www.ebi.ac.uk/QuickGO/GTerm?id=GO:0044325), [cadherin binding](http://www.ebi.ac.uk/QuickGO/GTerm?id=GO:0045296), [ephrin receptor binding](http://www.ebi.ac.uk/QuickGO/GTerm?id=GO:0046875), [ATPase binding](http://www.ebi.ac.uk/QuickGO/GTerm?id=GO:0051117), [phosphoprotein binding](http://www.ebi.ac.uk/QuickGO/GTerm?id=GO:0051219), [BMP receptor binding](http://www.ebi.ac.uk/QuickGO/GTerm?id=GO:0070700), [growth factor receptor binding](http://www.ebi.ac.uk/QuickGO/GTerm?id=GO:0070851), [connexin binding](http://www.ebi.ac.uk/QuickGO/GTerm?id=GO:0071253), [scaffold protein binding](http://www.ebi.ac.uk/QuickGO/GTerm?id=GO:0097110), | | |
| **INTERPRO** | [Protein kinase, catalytic domain](https://www.ebi.ac.uk/interpro/entry/InterPro/IPR000719), [SH2 domain](https://www.ebi.ac.uk/interpro/entry/InterPro/IPR000980), [Serine-threonine/tyrosine-protein kinase catalytic domain](https://www.ebi.ac.uk/interpro/entry/InterPro/IPR001245), [Src homology-3 domain](https://www.ebi.ac.uk/interpro/entry/InterPro/IPR001452), [Tyrosine-protein kinase, active site](https://www.ebi.ac.uk/interpro/entry/InterPro/IPR008266), [Protein kinase-like domain](https://www.ebi.ac.uk/interpro/entry/InterPro/IPR011009), [Protein kinase, ATP binding site](https://www.ebi.ac.uk/interpro/entry/InterPro/IPR017441), [Tyrosine-protein kinase, catalytic domain](https://www.ebi.ac.uk/interpro/entry/InterPro/IPR020635), | | |
| **KEGG_PATHWAY** | [EGFR tyrosine kinase inhibitor resistance](https://david.ncifcrf.gov/kegg.jsp?path=hsa01521$EGFR%20tyrosine%20kinase%20inhibitor%20resistance&termId=520047918&source=kegg), [Endocrine resistance](https://david.ncifcrf.gov/kegg.jsp?path=hsa01522$Endocrine%20resistance&termId=520047919&source=kegg), [ErbB signaling pathway](https://david.ncifcrf.gov/kegg.jsp?path=hsa04012$ErbB%20signaling%20pathway&termId=520047942&source=kegg), [Rap1 signaling pathway](https://david.ncifcrf.gov/kegg.jsp?path=hsa04015$Rap1%20signaling%20pathway&termId=520047944&source=kegg), [Chemokine signaling pathway](https://david.ncifcrf.gov/kegg.jsp?path=hsa04062$Chemokine%20signaling%20pathway&termId=520047950&source=kegg), [Mitophagy - animal](https://david.ncifcrf.gov/kegg.jsp?path=hsa04137$Mitophagy%20-%20animal&termId=520047965&source=kegg), [Endocytosis](https://david.ncifcrf.gov/kegg.jsp?path=hsa04144$Endocytosis&termId=520047969&source=kegg), [Axon guidance](https://david.ncifcrf.gov/kegg.jsp?path=hsa04360$Axon%20guidance&termId=520047989&source=kegg), [VEGF signaling pathway](https://david.ncifcrf.gov/kegg.jsp?path=hsa04370$VEGF%20signaling%20pathway&termId=520047990&source=kegg), [Focal adhesion](https://david.ncifcrf.gov/kegg.jsp?path=hsa04510$Focal%20adhesion&termId=520047995&source=kegg), [Adherens junction](https://david.ncifcrf.gov/kegg.jsp?path=hsa04520$Adherens%20junction&termId=520047998&source=kegg), [Tight junction](https://david.ncifcrf.gov/kegg.jsp?path=hsa04530$Tight%20junction&termId=520047999&source=kegg), [Gap junction](https://david.ncifcrf.gov/kegg.jsp?path=hsa04540$Gap%20junction&termId=520048000&source=kegg), [Platelet activation](https://david.ncifcrf.gov/kegg.jsp?path=hsa04611$Platelet%20activation&termId=520048003&source=kegg), [Neutrophil extracellular trap formation](https://david.ncifcrf.gov/kegg.jsp?path=hsa04613$Neutrophil%20extracellular%20trap%20formation&termId=520048005&source=kegg), [C-type lectin receptor signaling pathway](https://david.ncifcrf.gov/kegg.jsp?path=hsa04625$C-type%20lectin%20receptor%20signaling%20pathway&termId=520048011&source=kegg), [GABAergic synapse](https://david.ncifcrf.gov/kegg.jsp?path=hsa04727$GABAergic%20synapse&termId=520048035&source=kegg), [Inflammatory mediator regulation of TRP channels](https://david.ncifcrf.gov/kegg.jsp?path=hsa04750$Inflammatory%20mediator%20regulation%20of%20TRP%20channels&termId=520048041&source=kegg), [Regulation of actin cytoskeleton](https://david.ncifcrf.gov/kegg.jsp?path=hsa04810$Regulation%20of%20actin%20cytoskeleton&termId=520048042&source=kegg), [GnRH signaling pathway](https://david.ncifcrf.gov/kegg.jsp?path=hsa04912$GnRH%20signaling%20pathway&termId=520048045&source=kegg), [Estrogen signaling pathway](https://david.ncifcrf.gov/kegg.jsp?path=hsa04915$Estrogen%20signaling%20pathway&termId=520048048&source=kegg), [Prolactin signaling pathway](https://david.ncifcrf.gov/kegg.jsp?path=hsa04917$Prolactin%20signaling%20pathway&termId=520048050&source=kegg), [Thyroid hormone signaling pathway](https://david.ncifcrf.gov/kegg.jsp?path=hsa04919$Thyroid%20hormone%20signaling%20pathway&termId=520048052&source=kegg), [Oxytocin signaling pathway](https://david.ncifcrf.gov/kegg.jsp?path=hsa04921$Oxytocin%20signaling%20pathway&termId=520048054&source=kegg), [Relaxin signaling pathway](https://david.ncifcrf.gov/kegg.jsp?path=hsa04926$Relaxin%20signaling%20pathway&termId=520048059&source=kegg), [Bacterial invasion of epithelial cells](https://david.ncifcrf.gov/kegg.jsp?path=hsa05100$Bacterial%20invasion%20of%20epithelial%20cells&termId=520048099&source=kegg), [Epithelial cell signaling in Helicobacter pylori infection](https://david.ncifcrf.gov/kegg.jsp?path=hsa05120$Epithelial%20cell%20signaling%20in%20Helicobacter%20pylori%20infection&termId=520048101&source=kegg), [Pathogenic Escherichia coli infection](https://david.ncifcrf.gov/kegg.jsp?path=hsa05130$Pathogenic%20Escherichia%20coli%20infection&termId=520048102&source=kegg), [Shigellosis](https://david.ncifcrf.gov/kegg.jsp?path=hsa05131$Shigellosis&termId=520048103&source=kegg), [Yersinia infection](https://david.ncifcrf.gov/kegg.jsp?path=hsa05135$Yersinia%20infection&termId=520048107&source=kegg), [Tuberculosis](https://david.ncifcrf.gov/kegg.jsp?path=hsa05152$Tuberculosis&termId=520048115&source=kegg), [Hepatitis B](https://david.ncifcrf.gov/kegg.jsp?path=hsa05161$Hepatitis%20B&termId=520048117&source=kegg), [Human cytomegalovirus infection](https://david.ncifcrf.gov/kegg.jsp?path=hsa05163$Human%20cytomegalovirus%20infection&termId=520048119&source=kegg), [Kaposi sarcoma-associated herpesvirus infection](https://david.ncifcrf.gov/kegg.jsp?path=hsa05167$Kaposi%20sarcoma-associated%20herpesvirus%20infection&termId=520048123&source=kegg), [Herpes simplex virus 1 infection](https://david.ncifcrf.gov/kegg.jsp?path=hsa05168$Herpes%20simplex%20virus%201%20infection&termId=520048124&source=kegg), [Viral carcinogenesis](https://david.ncifcrf.gov/kegg.jsp?path=hsa05203$Viral%20carcinogenesis&termId=520048130&source=kegg), [Proteoglycans in cancer](https://david.ncifcrf.gov/kegg.jsp?path=hsa05205$Proteoglycans%20in%20cancer&termId=520048132&source=kegg), [Chemical carcinogenesis - receptor activation](https://david.ncifcrf.gov/kegg.jsp?path=hsa05207$Chemical%20carcinogenesis%20-%20receptor%20activation&termId=520048134&source=kegg), [Chemical carcinogenesis - reactive oxygen species](https://david.ncifcrf.gov/kegg.jsp?path=hsa05208$Chemical%20carcinogenesis%20-%20reactive%20oxygen%20species&termId=520048135&source=kegg), [Bladder cancer](https://david.ncifcrf.gov/kegg.jsp?path=hsa05219$Bladder%20cancer&termId=520048145&source=kegg), [Lipid and atherosclerosis](https://david.ncifcrf.gov/kegg.jsp?path=hsa05417$Lipid%20and%20atherosclerosis&termId=520048169&source=kegg), [Fluid shear stress and atherosclerosis](https://david.ncifcrf.gov/kegg.jsp?path=hsa05418$Fluid%20shear%20stress%20and%20atherosclerosis&termId=520048170&source=kegg), | | |
| **OMIM_DISEASE** | [Colon cancer, advanced, somatic](http://omim.org/entry/114500), [Thrombocytopenia 6](http://omim.org/entry/616937), | | |
| **SMART** | [TyrKc](http://smart.embl.de/smart/do_annotation.pl?DOMAIN=SM00219), [SH2](http://smart.embl.de/smart/do_annotation.pl?DOMAIN=SM00252), [SH3](http://smart.embl.de/smart/do_annotation.pl?DOMAIN=SM00326), | | |
| **UP_KW_BIOLOGICAL_PROCESS** | [Cell adhesion](http://www.uniprot.org/keywords/?query=KW-0130), [Cell cycle](http://www.uniprot.org/keywords/?query=KW-0131), [Immunity](http://www.uniprot.org/keywords/?query=KW-0391), [Host-virus interaction](http://www.uniprot.org/keywords/?query=KW-0945), | | |
| **UP_KW_CELLULAR_COMPONENT** | [Cytoskeleton](http://www.uniprot.org/keywords/?query=KW-0206), [Membrane](http://www.uniprot.org/keywords/?query=KW-0472), [Mitochondrion](http://www.uniprot.org/keywords/?query=KW-0496), [Nucleus](http://www.uniprot.org/keywords/?query=KW-0539), [Cytoplasm](http://www.uniprot.org/keywords/?query=KW-0963), [Cell junction](http://www.uniprot.org/keywords/?query=KW-0965), [Mitochondrion inner membrane](http://www.uniprot.org/keywords/?query=KW-0999), [Cell membrane](http://www.uniprot.org/keywords/?query=KW-1003), | | |
| **UP_KW_DISEASE** | [Disease variant](http://www.uniprot.org/keywords/?query=KW-0225), [Proto-oncogene](http://www.uniprot.org/keywords/?query=KW-0656), | | |
| **UP_KW_DOMAIN** | [SH2 domain](http://www.uniprot.org/keywords/?query=KW-0727), [SH3 domain](http://www.uniprot.org/keywords/?query=KW-0728), | | |
| **UP_KW_LIGAND** | [ATP-binding](http://www.uniprot.org/keywords/?query=KW-0067), [Nucleotide-binding](http://www.uniprot.org/keywords/?query=KW-0547), | | |
| **UP_KW_MOLECULAR_FUNCTION** | [Kinase](http://www.uniprot.org/keywords/?query=KW-0418), [Transferase](http://www.uniprot.org/keywords/?query=KW-0808), [Tyrosine-protein kinase](http://www.uniprot.org/keywords/?query=KW-0829), | | |
| **UP_KW_PTM** | [Lipoprotein](http://www.uniprot.org/keywords/?query=KW-0449), [Myristate](http://www.uniprot.org/keywords/?query=KW-0519), [Phosphoprotein](http://www.uniprot.org/keywords/?query=KW-0597), [Ubl conjugation](http://www.uniprot.org/keywords/?query=KW-0832), | | |
| **UP_SEQ_FEATURE** | ACT_SITE:Proton acceptor, BINDING:ATP, DOMAIN:PK_Tyr_Ser-Thr, DOMAIN:Protein kinase, DOMAIN:SH2, DOMAIN:SH3, LIPID:N-myristoyl glycine, MUTAGEN:K->M: Kinase inactive. Abolishes ubiquitination promoted by CBLC., MUTAGEN:P->E: Kinase active. Interacts with PDLIM4; when associated with E-302 and F-419., MUTAGEN:P->E: Kinase active. Interacts with PDLIM4; when associated with E-307 and F-419., MUTAGEN:Y->F: Loss of kinase activity. Loss of interaction with PDLIM4., NP_BIND:ATP, REGION:Disordered, | | |
| **SUFU** | [**SUFU negative regulator of hedgehog signaling(SUFU)**](https://david.ncifcrf.gov/geneReportFull.jsp?rowids=51684) | [**Related Genes**](https://david.ncifcrf.gov/relatedGenes.jsp?id=51684) | [**Homo sapiens**](http://www.ncbi.nlm.nih.gov/Taxonomy/Browser/wwwtax.cgi?name=Homo%20sapiens) |
| **BIOCARTA** | [Sonic Hedgehog (Shh) Pathway](https://david.ncifcrf.gov/biocarta.jsp?path=h_shhPathway$Sonic%20Hedgehog%20(Shh)%20Pathway&termId=30000270&source=biocarta), | | |
| **GOTERM_BP_DIRECT** | [negative regulation of transcription from RNA polymerase II promoter](http://www.ebi.ac.uk/QuickGO/GTerm?id=GO:0000122), [skeletal system development](http://www.ebi.ac.uk/QuickGO/GTerm?id=GO:0001501), [neural tube closure](http://www.ebi.ac.uk/QuickGO/GTerm?id=GO:0001843), [heart looping](http://www.ebi.ac.uk/QuickGO/GTerm?id=GO:0001947), [ventricular septum development](http://www.ebi.ac.uk/QuickGO/GTerm?id=GO:0003281), [regulation of transcription, DNA-templated](http://www.ebi.ac.uk/QuickGO/GTerm?id=GO:0006355), [proteolysis](http://www.ebi.ac.uk/QuickGO/GTerm?id=GO:0006508), [signal transduction](http://www.ebi.ac.uk/QuickGO/GTerm?id=GO:0007165), [multicellular organism development](http://www.ebi.ac.uk/QuickGO/GTerm?id=GO:0007275), [smoothened signaling pathway involved in ventral spinal cord interneuron specification](http://www.ebi.ac.uk/QuickGO/GTerm?id=GO:0021775), [smoothened signaling pathway involved in spinal cord motor neuron cell fate specification](http://www.ebi.ac.uk/QuickGO/GTerm?id=GO:0021776), [aorta development](http://www.ebi.ac.uk/QuickGO/GTerm?id=GO:0035904), [negative regulation of protein import into nucleus](http://www.ebi.ac.uk/QuickGO/GTerm?id=GO:0042308), [cytoplasmic sequestering of transcription factor](http://www.ebi.ac.uk/QuickGO/GTerm?id=GO:0042994), [negative regulation of sequence-specific DNA binding transcription factor activity](http://www.ebi.ac.uk/QuickGO/GTerm?id=GO:0043433), [skin development](http://www.ebi.ac.uk/QuickGO/GTerm?id=GO:0043588), [negative regulation of osteoblast differentiation](http://www.ebi.ac.uk/QuickGO/GTerm?id=GO:0045668), [negative regulation of smoothened signaling pathway](http://www.ebi.ac.uk/QuickGO/GTerm?id=GO:0045879), [coronary vasculature development](http://www.ebi.ac.uk/QuickGO/GTerm?id=GO:0060976), [negative regulation of smoothened signaling pathway involved in dorsal/ventral neural tube patterning](http://www.ebi.ac.uk/QuickGO/GTerm?id=GO:1901621), [negative regulation of hh target transcription factor activity](http://www.ebi.ac.uk/QuickGO/GTerm?id=GO:1990787), [negative regulation of protein ubiquitination involved in ubiquitin-dependent protein catabolic process](http://www.ebi.ac.uk/QuickGO/GTerm?id=GO:2000059), | | |
| **GOTERM_CC_DIRECT** | [nucleus](http://www.ebi.ac.uk/QuickGO/GTerm?id=GO:0005634), [nucleoplasm](http://www.ebi.ac.uk/QuickGO/GTerm?id=GO:0005654), [cytoplasm](http://www.ebi.ac.uk/QuickGO/GTerm?id=GO:0005737), [cytosol](http://www.ebi.ac.uk/QuickGO/GTerm?id=GO:0005829), [ciliary tip](http://www.ebi.ac.uk/QuickGO/GTerm?id=GO:0097542), [ciliary base](http://www.ebi.ac.uk/QuickGO/GTerm?id=GO:0097546), [GLI-SUFU complex](http://www.ebi.ac.uk/QuickGO/GTerm?id=GO:1990788), | | |
| **GOTERM_MF_DIRECT** | [transcription corepressor activity](http://www.ebi.ac.uk/QuickGO/GTerm?id=GO:0003714), [protein binding](http://www.ebi.ac.uk/QuickGO/GTerm?id=GO:0005515), [beta-catenin binding](http://www.ebi.ac.uk/QuickGO/GTerm?id=GO:0008013), [transcription factor binding](http://www.ebi.ac.uk/QuickGO/GTerm?id=GO:0008134), [protein kinase binding](http://www.ebi.ac.uk/QuickGO/GTerm?id=GO:0019901), | | |
| **INTERPRO** | [Suppressor of fused-like](https://www.ebi.ac.uk/interpro/entry/InterPro/IPR007768), [Suppressor of fused protein](https://www.ebi.ac.uk/interpro/entry/InterPro/IPR016591), [Suppressor of fused domain](https://www.ebi.ac.uk/interpro/entry/InterPro/IPR020941), [Suppressor of fused C-terminal](https://www.ebi.ac.uk/interpro/entry/InterPro/IPR024314), | | |
| **KEGG_PATHWAY** | [Hedgehog signaling pathway](https://david.ncifcrf.gov/kegg.jsp?path=hsa04340$Hedgehog%20signaling%20pathway&termId=520047987&source=kegg), [Pathways in cancer](https://david.ncifcrf.gov/kegg.jsp?path=hsa05200$Pathways%20in%20cancer&termId=520048128&source=kegg), [Basal cell carcinoma](https://david.ncifcrf.gov/kegg.jsp?path=hsa05217$Basal%20cell%20carcinoma&termId=520048143&source=kegg), | | |
| **OMIM_DISEASE** | [Basal cell nevus syndrome](http://omim.org/entry/109400), [Medulloblastoma, desmoplastic](http://omim.org/entry/155255), [Meningioma, familial, susceptibility to](http://omim.org/entry/607174), [Joubert syndrome 32](http://omim.org/entry/617757), | | |
| **PIR_SUPERFAMILY** | [suppressor of fused protein](http://pir.georgetown.edu/cgi-bin/ipcSF?id=PIRSF011844), | | |
| **UP_KW_CELLULAR_COMPONENT** | [Nucleus](http://www.uniprot.org/keywords/?query=KW-0539), [Cytoplasm](http://www.uniprot.org/keywords/?query=KW-0963), | | |
| **UP_KW_DISEASE** | [Tumor suppressor](http://www.uniprot.org/keywords/?query=KW-0043), [Disease variant](http://www.uniprot.org/keywords/?query=KW-0225), [Joubert syndrome](http://www.uniprot.org/keywords/?query=KW-0979), [Ciliopathy](http://www.uniprot.org/keywords/?query=KW-1186), | | |
| **UP_KW_MOLECULAR_FUNCTION** | [Developmental protein](http://www.uniprot.org/keywords/?query=KW-0217), [Developmental protein](http://www.uniprot.org/keywords/?query=KW-9996), | | |
| **UP_KW_PTM** | [Acetylation](http://www.uniprot.org/keywords/?query=KW-0007), [Phosphoprotein](http://www.uniprot.org/keywords/?query=KW-0597), [Ubl conjugation](http://www.uniprot.org/keywords/?query=KW-0832), [Isopeptide bond](http://www.uniprot.org/keywords/?query=KW-1017), | | |
| **UP_SEQ_FEATURE** | COMPBIAS:Pro residues, CROSSLNK:Glycyl lysine isopeptide (Lys-Gly) (interchain with G-Cter in SUMO2), CROSSLNK:Glycyl lysine isopeptide (Lys-Gly) (interchain with G-Cter in ubiquitin), DOMAIN:SUFU, DOMAIN:SUFU_C, MUTAGEN:D->A: Abolishes down-regulation of GLI1 activity. Has only slight effect on GLI1 binding., MUTAGEN:D->A: No effect on down-regulation of GLI1 activity., MUTAGEN:D->R: Impairs interaction with GLI1 and GLI2. Abolishes interaction with GLI1 and GLI2; when associated with R-147 and R-380., MUTAGEN:E->A: No effect on down-regulation of GLI1 activity., MUTAGEN:K->R: Abolishes ubiquitination by the SCF(FBXL17) complex., MUTAGEN:L->R: Impairs interaction with GLI1 and GLI2. Abolishes interaction with GLI1 and GLI2; when associated with R-147 and R-159., MUTAGEN:SRKDS->ARKDA: Increased interaction with FBXL17 and ubiquitination by the SCF(FBXL17) complex., MUTAGEN:SRKDS->DRKDD: Phosphomimetic mutant; decreased interaction with FBXL17 and ubiquitination by the SCF(FBXL17) complex., MUTAGEN:T->A,D: No effect on down-regulation of GLI1 activity., MUTAGEN:Y->R: Impairs interaction with GLI1 and GLI2. Abolishes interaction with GLI1 and GLI2; when associated with R-159 and R-380., REGION:Disordered, | | |
| **SMARCC1** | [**SWI/SNF related, matrix associated, actin dependent regulator of chromatin subfamily c member 1(SMARCC1)**](https://david.ncifcrf.gov/geneReportFull.jsp?rowids=6599) | [**Related Genes**](https://david.ncifcrf.gov/relatedGenes.jsp?id=6599) | [**Homo sapiens**](http://www.ncbi.nlm.nih.gov/Taxonomy/Browser/wwwtax.cgi?name=Homo%20sapiens) |
| **BIOCARTA** | [Chromatin Remodeling by hSWI/SNF ATP-dependent Complexes](https://david.ncifcrf.gov/biocarta.jsp?path=h_hSWI-SNFpathway$Chromatin%20Remodeling%20by%20hSWI/SNF%20ATP-dependent%20Complexes&termId=30000143&source=biocarta), [Control of Gene Expression by Vitamin D Receptor](https://david.ncifcrf.gov/biocarta.jsp?path=h_vdrPathway$Control%20of%20Gene%20Expression%20by%20Vitamin%20D%20Receptor&termId=30000307&source=biocarta), | | |
| **COG_ONTOLOGY** | [Chromatin structure and dynamics / Transcription](http://www.ncbi.nlm.nih.gov/COG/new/), [General function prediction only](http://www.ncbi.nlm.nih.gov/COG/new/), | | |
| **GOTERM_BP_DIRECT** | [nucleosome disassembly](http://www.ebi.ac.uk/QuickGO/GTerm?id=GO:0006337), [chromatin remodeling](http://www.ebi.ac.uk/QuickGO/GTerm?id=GO:0006338), [regulation of transcription from RNA polymerase II promoter](http://www.ebi.ac.uk/QuickGO/GTerm?id=GO:0006357), [nervous system development](http://www.ebi.ac.uk/QuickGO/GTerm?id=GO:0007399), [insulin receptor signaling pathway](http://www.ebi.ac.uk/QuickGO/GTerm?id=GO:0008286), [animal organ morphogenesis](http://www.ebi.ac.uk/QuickGO/GTerm?id=GO:0009887), [prostate gland development](http://www.ebi.ac.uk/QuickGO/GTerm?id=GO:0030850), [negative regulation of proteasomal ubiquitin-dependent protein catabolic process](http://www.ebi.ac.uk/QuickGO/GTerm?id=GO:0032435), [ATP-dependent chromatin remodeling](http://www.ebi.ac.uk/QuickGO/GTerm?id=GO:0043044), [positive regulation of transcription, DNA-templated](http://www.ebi.ac.uk/QuickGO/GTerm?id=GO:0045893), [positive regulation of transcription from RNA polymerase II promoter](http://www.ebi.ac.uk/QuickGO/GTerm?id=GO:0045944), | | |
| **GOTERM_CC_DIRECT** | [chromatin](http://www.ebi.ac.uk/QuickGO/GTerm?id=GO:0000785), [XY body](http://www.ebi.ac.uk/QuickGO/GTerm?id=GO:0001741), [nucleus](http://www.ebi.ac.uk/QuickGO/GTerm?id=GO:0005634), [nucleoplasm](http://www.ebi.ac.uk/QuickGO/GTerm?id=GO:0005654), [cytoplasm](http://www.ebi.ac.uk/QuickGO/GTerm?id=GO:0005737), [SWI/SNF complex](http://www.ebi.ac.uk/QuickGO/GTerm?id=GO:0016514), [macromolecular complex](http://www.ebi.ac.uk/QuickGO/GTerm?id=GO:0032991), [npBAF complex](http://www.ebi.ac.uk/QuickGO/GTerm?id=GO:0071564), [nBAF complex](http://www.ebi.ac.uk/QuickGO/GTerm?id=GO:0071565), | | |
| **GOTERM_MF_DIRECT** | [chromatin binding](http://www.ebi.ac.uk/QuickGO/GTerm?id=GO:0003682), [transcription coactivator activity](http://www.ebi.ac.uk/QuickGO/GTerm?id=GO:0003713), [protein binding](http://www.ebi.ac.uk/QuickGO/GTerm?id=GO:0005515), [nucleosomal DNA binding](http://www.ebi.ac.uk/QuickGO/GTerm?id=GO:0031492), [histone binding](http://www.ebi.ac.uk/QuickGO/GTerm?id=GO:0042393), [protein N-terminus binding](http://www.ebi.ac.uk/QuickGO/GTerm?id=GO:0047485), | | |
| **INTERPRO** | [Chromo domain/shadow](https://www.ebi.ac.uk/interpro/entry/InterPro/IPR000953), [SANT/Myb domain](https://www.ebi.ac.uk/interpro/entry/InterPro/IPR001005), [SWIRM domain](https://www.ebi.ac.uk/interpro/entry/InterPro/IPR007526), [Homeodomain-like](https://www.ebi.ac.uk/interpro/entry/InterPro/IPR009057), [SANT domain](https://www.ebi.ac.uk/interpro/entry/InterPro/IPR017884), | | |
| **KEGG_PATHWAY** | [Thermogenesis](https://david.ncifcrf.gov/kegg.jsp?path=hsa04714$Thermogenesis&termId=520048027&source=kegg), [Hepatocellular carcinoma](https://david.ncifcrf.gov/kegg.jsp?path=hsa05225$Hepatocellular%20carcinoma&termId=520048151&source=kegg), | | |
| **SMART** | [CHROMO](http://smart.embl.de/smart/do_annotation.pl?DOMAIN=SM00298), [SANT](http://smart.embl.de/smart/do_annotation.pl?DOMAIN=SM00717), | | |
| **UP_KW_BIOLOGICAL_PROCESS** | [Neurogenesis](http://www.uniprot.org/keywords/?query=KW-0524), [Transcription](http://www.uniprot.org/keywords/?query=KW-0804), [Transcription regulation](http://www.uniprot.org/keywords/?query=KW-0805), | | |
| **UP_KW_CELLULAR_COMPONENT** | [Nucleus](http://www.uniprot.org/keywords/?query=KW-0539), [Cytoplasm](http://www.uniprot.org/keywords/?query=KW-0963), | | |
| **UP_KW_DOMAIN** | [Coiled coil](http://www.uniprot.org/keywords/?query=KW-0175), | | |
| **UP_KW_MOLECULAR_FUNCTION** | [Chromatin regulator](http://www.uniprot.org/keywords/?query=KW-0156), | | |
| **UP_KW_PTM** | [Acetylation](http://www.uniprot.org/keywords/?query=KW-0007), [Methylation](http://www.uniprot.org/keywords/?query=KW-0488), [Phosphoprotein](http://www.uniprot.org/keywords/?query=KW-0597), [Ubl conjugation](http://www.uniprot.org/keywords/?query=KW-0832), [Isopeptide bond](http://www.uniprot.org/keywords/?query=KW-1017), | | |
| **UP_SEQ_FEATURE** | COMPBIAS:Basic and acidic residues, COMPBIAS:Polar residues, COMPBIAS:Pro residues, CROSSLNK:Glycyl lysine isopeptide (Lys-Gly) (interchain with G-Cter in SUMO2), CROSSLNK:Glycyl lysine isopeptide (Lys-Gly) (interchain with G-Cter in SUMO2); alternate, DOMAIN:Myb-like, DOMAIN:SANT, DOMAIN:SWIRM, DOMAIN:SWIRM-assoc_2, REGION:Disordered, | | |
| **SHF** | [**Src homology 2 domain containing F(SHF)**](https://david.ncifcrf.gov/geneReportFull.jsp?rowids=90525) | [**Related Genes**](https://david.ncifcrf.gov/relatedGenes.jsp?id=90525) | [**Homo sapiens**](http://www.ncbi.nlm.nih.gov/Taxonomy/Browser/wwwtax.cgi?name=Homo%20sapiens) |
| **GOTERM_BP_DIRECT** | [apoptotic process](http://www.ebi.ac.uk/QuickGO/GTerm?id=GO:0006915), | | |
| **GOTERM_MF_DIRECT** | [phosphotyrosine binding](http://www.ebi.ac.uk/QuickGO/GTerm?id=GO:0001784), | | |
| **INTERPRO** | [SH2 domain](https://www.ebi.ac.uk/interpro/entry/InterPro/IPR000980), | | |
| **SMART** | [SH2](http://smart.embl.de/smart/do_annotation.pl?DOMAIN=SM00252), | | |
| **UP_KW_BIOLOGICAL_PROCESS** | [Apoptosis](http://www.uniprot.org/keywords/?query=KW-0053), | | |
| **UP_KW_DOMAIN** | [SH2 domain](http://www.uniprot.org/keywords/?query=KW-0727), | | |
| **UP_KW_PTM** | [Phosphoprotein](http://www.uniprot.org/keywords/?query=KW-0597), | | |
| **UP_SEQ_FEATURE** | COMPBIAS:Basic and acidic residues, COMPBIAS:Polar residues, COMPBIAS:Pro residues, DOMAIN:SH2, REGION:Disordered, | | |
| **TSC2** | [**TSC complex subunit 2(TSC2)**](https://david.ncifcrf.gov/geneReportFull.jsp?rowids=7249) | [**Related Genes**](https://david.ncifcrf.gov/relatedGenes.jsp?id=7249) | [**Homo sapiens**](http://www.ncbi.nlm.nih.gov/Taxonomy/Browser/wwwtax.cgi?name=Homo%20sapiens) |
| **BIOCARTA** | [mTOR Signaling Pathway](https://david.ncifcrf.gov/biocarta.jsp?path=h_mtorPathway$mTOR%20Signaling%20Pathway&termId=30000196&source=biocarta), [Control of Gene Expression by Vitamin D Receptor](https://david.ncifcrf.gov/biocarta.jsp?path=h_vdrPathway$Control%20of%20Gene%20Expression%20by%20Vitamin%20D%20Receptor&termId=30000307&source=biocarta), | | |
| **GOTERM_BP_DIRECT** | [neural tube closure](http://www.ebi.ac.uk/QuickGO/GTerm?id=GO:0001843), [negative regulation of protein kinase activity](http://www.ebi.ac.uk/QuickGO/GTerm?id=GO:0006469), [protein import into nucleus](http://www.ebi.ac.uk/QuickGO/GTerm?id=GO:0006606), [endocytosis](http://www.ebi.ac.uk/QuickGO/GTerm?id=GO:0006897), [heart development](http://www.ebi.ac.uk/QuickGO/GTerm?id=GO:0007507), [protein localization](http://www.ebi.ac.uk/QuickGO/GTerm?id=GO:0008104), [negative regulation of cell proliferation](http://www.ebi.ac.uk/QuickGO/GTerm?id=GO:0008285), [negative regulation of phosphatidylinositol 3-kinase signaling](http://www.ebi.ac.uk/QuickGO/GTerm?id=GO:0014067), [viral process](http://www.ebi.ac.uk/QuickGO/GTerm?id=GO:0016032), [vesicle-mediated transport](http://www.ebi.ac.uk/QuickGO/GTerm?id=GO:0016192), [positive regulation of macroautophagy](http://www.ebi.ac.uk/QuickGO/GTerm?id=GO:0016239), [regulation of endocytosis](http://www.ebi.ac.uk/QuickGO/GTerm?id=GO:0030100), [negative regulation of Wnt signaling pathway](http://www.ebi.ac.uk/QuickGO/GTerm?id=GO:0030178), [negative regulation of TOR signaling](http://www.ebi.ac.uk/QuickGO/GTerm?id=GO:0032007), [anoikis](http://www.ebi.ac.uk/QuickGO/GTerm?id=GO:0043276), [protein kinase B signaling](http://www.ebi.ac.uk/QuickGO/GTerm?id=GO:0043491), [positive regulation of GTPase activity](http://www.ebi.ac.uk/QuickGO/GTerm?id=GO:0043547), [regulation of insulin receptor signaling pathway](http://www.ebi.ac.uk/QuickGO/GTerm?id=GO:0046626), [negative regulation of insulin receptor signaling pathway](http://www.ebi.ac.uk/QuickGO/GTerm?id=GO:0046627), [insulin-like growth factor receptor signaling pathway](http://www.ebi.ac.uk/QuickGO/GTerm?id=GO:0048009), [positive chemotaxis](http://www.ebi.ac.uk/QuickGO/GTerm?id=GO:0050918), [regulation of small GTPase mediated signal transduction](http://www.ebi.ac.uk/QuickGO/GTerm?id=GO:0051056), [regulation of cell cycle](http://www.ebi.ac.uk/QuickGO/GTerm?id=GO:0051726), [negative regulation of protein kinase B signaling](http://www.ebi.ac.uk/QuickGO/GTerm?id=GO:0051898), [negative regulation of macromitophagy](http://www.ebi.ac.uk/QuickGO/GTerm?id=GO:1901525), | | |
| **GOTERM_CC_DIRECT** | [nucleus](http://www.ebi.ac.uk/QuickGO/GTerm?id=GO:0005634), [cytoplasm](http://www.ebi.ac.uk/QuickGO/GTerm?id=GO:0005737), [lysosome](http://www.ebi.ac.uk/QuickGO/GTerm?id=GO:0005764), [Golgi apparatus](http://www.ebi.ac.uk/QuickGO/GTerm?id=GO:0005794), [cytosol](http://www.ebi.ac.uk/QuickGO/GTerm?id=GO:0005829), [postsynaptic density](http://www.ebi.ac.uk/QuickGO/GTerm?id=GO:0014069), [membrane](http://www.ebi.ac.uk/QuickGO/GTerm?id=GO:0016020), [TSC1-TSC2 complex](http://www.ebi.ac.uk/QuickGO/GTerm?id=GO:0033596), [perinuclear region of cytoplasm](http://www.ebi.ac.uk/QuickGO/GTerm?id=GO:0048471), | | |
| **GOTERM_MF_DIRECT** | [GTPase activator activity](http://www.ebi.ac.uk/QuickGO/GTerm?id=GO:0005096), [protein binding](http://www.ebi.ac.uk/QuickGO/GTerm?id=GO:0005515), [phosphatase binding](http://www.ebi.ac.uk/QuickGO/GTerm?id=GO:0019902), [small GTPase binding](http://www.ebi.ac.uk/QuickGO/GTerm?id=GO:0031267), [protein homodimerization activity](http://www.ebi.ac.uk/QuickGO/GTerm?id=GO:0042803), [Hsp90 protein binding](http://www.ebi.ac.uk/QuickGO/GTerm?id=GO:0051879), | | |
| **INTERPRO** | [Rap GTPase activating proteins domain](https://www.ebi.ac.uk/interpro/entry/InterPro/IPR000331), [Tuberin](https://www.ebi.ac.uk/interpro/entry/InterPro/IPR003913), [Armadillo-type fold](https://www.ebi.ac.uk/interpro/entry/InterPro/IPR016024), [Tuberin-type domain](https://www.ebi.ac.uk/interpro/entry/InterPro/IPR018515), [Tuberin, N-terminal](https://www.ebi.ac.uk/interpro/entry/InterPro/IPR024584), [Tuberin/Ral GTPase-activating protein subunit alpha](https://www.ebi.ac.uk/interpro/entry/InterPro/IPR027107), | | |
| **KEGG_PATHWAY** | [Phospholipase D signaling pathway](https://david.ncifcrf.gov/kegg.jsp?path=hsa04072$Phospholipase%20D%20signaling%20pathway&termId=520047956&source=kegg), [p53 signaling pathway](https://david.ncifcrf.gov/kegg.jsp?path=hsa04115$p53%20signaling%20pathway&termId=520047960&source=kegg), [Autophagy - animal](https://david.ncifcrf.gov/kegg.jsp?path=hsa04140$Autophagy%20-%20animal&termId=520047966&source=kegg), [mTOR signaling pathway](https://david.ncifcrf.gov/kegg.jsp?path=hsa04150$mTOR%20signaling%20pathway&termId=520047972&source=kegg), [PI3K-Akt signaling pathway](https://david.ncifcrf.gov/kegg.jsp?path=hsa04151$PI3K-Akt%20signaling%20pathway&termId=520047973&source=kegg), [AMPK signaling pathway](https://david.ncifcrf.gov/kegg.jsp?path=hsa04152$AMPK%20signaling%20pathway&termId=520047974&source=kegg), [Longevity regulating pathway](https://david.ncifcrf.gov/kegg.jsp?path=hsa04211$Longevity%20regulating%20pathway&termId=520047976&source=kegg), [Cellular senescence](https://david.ncifcrf.gov/kegg.jsp?path=hsa04218$Cellular%20senescence&termId=520047981&source=kegg), [Thermogenesis](https://david.ncifcrf.gov/kegg.jsp?path=hsa04714$Thermogenesis&termId=520048027&source=kegg), [Insulin signaling pathway](https://david.ncifcrf.gov/kegg.jsp?path=hsa04910$Insulin%20signaling%20pathway&termId=520048043&source=kegg), [Thyroid hormone signaling pathway](https://david.ncifcrf.gov/kegg.jsp?path=hsa04919$Thyroid%20hormone%20signaling%20pathway&termId=520048052&source=kegg), [Human cytomegalovirus infection](https://david.ncifcrf.gov/kegg.jsp?path=hsa05163$Human%20cytomegalovirus%20infection&termId=520048119&source=kegg), [Human papillomavirus infection](https://david.ncifcrf.gov/kegg.jsp?path=hsa05165$Human%20papillomavirus%20infection&termId=520048121&source=kegg), [Herpes simplex virus 1 infection](https://david.ncifcrf.gov/kegg.jsp?path=hsa05168$Herpes%20simplex%20virus%201%20infection&termId=520048124&source=kegg), [Choline metabolism in cancer](https://david.ncifcrf.gov/kegg.jsp?path=hsa05231$Choline%20metabolism%20in%20cancer&termId=520048154&source=kegg), | | |
| **OMIM_DISEASE** | [Lymphangioleiomyomatosis, somatic](http://omim.org/entry/606690), [Focal cortical dysplasia, type II, somatic](http://omim.org/entry/607341), [Tuberous sclerosis-2](http://omim.org/entry/613254), | | |
| **UP_KW_BIOLOGICAL_PROCESS** | [Host-virus interaction](http://www.uniprot.org/keywords/?query=KW-0945), | | |
| **UP_KW_CELLULAR_COMPONENT** | [Membrane](http://www.uniprot.org/keywords/?query=KW-0472), [Cytoplasm](http://www.uniprot.org/keywords/?query=KW-0963), | | |
| **UP_KW_DISEASE** | [Tumor suppressor](http://www.uniprot.org/keywords/?query=KW-0043), [Disease variant](http://www.uniprot.org/keywords/?query=KW-0225), [Epilepsy](http://www.uniprot.org/keywords/?query=KW-0887), | | |
| **UP_KW_MOLECULAR_FUNCTION** | [GTPase activation](http://www.uniprot.org/keywords/?query=KW-0343), | | |
| **UP_KW_PTM** | [Phosphoprotein](http://www.uniprot.org/keywords/?query=KW-0597), [Ubl conjugation](http://www.uniprot.org/keywords/?query=KW-0832), | | |
| **UP_SEQ_FEATURE** | COMPBIAS:Basic and acidic residues, COMPBIAS:Polar residues, DOMAIN:DUF3384, DOMAIN:Rap-GAP, DOMAIN:Tuberin, MUTAGEN:KKR->QQQ: Abolishes GAP activity., MUTAGEN:R->Q: Abolishes GAP activity., MUTAGEN:RLR->QLQ: No effect., MUTAGEN:S->A: Abolishes AMPK-mediated phosphorylation; when associated with A-1271., MUTAGEN:S->A: Inhibits insulin-stimulated phosphorylation and activation of S6K1; when associated with A-1462., MUTAGEN:T->A: Abolishes AMPK-mediated phosphorylation; when associated with A-1387., MUTAGEN:T->A: Inhibits insulin-stimulated phosphorylation and activation of S6K1; when associated with A-939., REGION:Disordered, REGION:Required for interaction with TSC1, | | |
| **UBXN11** | [**UBX domain protein 11(UBXN11)**](https://david.ncifcrf.gov/geneReportFull.jsp?rowids=91544) | [**Related Genes**](https://david.ncifcrf.gov/relatedGenes.jsp?id=91544) | [**Homo sapiens**](http://www.ncbi.nlm.nih.gov/Taxonomy/Browser/wwwtax.cgi?name=Homo%20sapiens) |
| **GOTERM_BP_DIRECT** | [proteasome-mediated ubiquitin-dependent protein catabolic process](http://www.ebi.ac.uk/QuickGO/GTerm?id=GO:0043161), | | |
| **GOTERM_CC_DIRECT** | [cytoplasm](http://www.ebi.ac.uk/QuickGO/GTerm?id=GO:0005737), [cytosol](http://www.ebi.ac.uk/QuickGO/GTerm?id=GO:0005829), [cytoskeleton](http://www.ebi.ac.uk/QuickGO/GTerm?id=GO:0005856), | | |
| **GOTERM_MF_DIRECT** | [protein binding](http://www.ebi.ac.uk/QuickGO/GTerm?id=GO:0005515), [ubiquitin binding](http://www.ebi.ac.uk/QuickGO/GTerm?id=GO:0043130), | | |
| **INTERPRO** | [UBX](https://www.ebi.ac.uk/interpro/entry/InterPro/IPR001012), [SEP domain](https://www.ebi.ac.uk/interpro/entry/InterPro/IPR012989), | | |
| **UP_KW_CELLULAR_COMPONENT** | [Cytoskeleton](http://www.uniprot.org/keywords/?query=KW-0206), [Cytoplasm](http://www.uniprot.org/keywords/?query=KW-0963), | | |
| **UP_KW_DOMAIN** | [Coiled coil](http://www.uniprot.org/keywords/?query=KW-0175), [Repeat](http://www.uniprot.org/keywords/?query=KW-0677), | | |
| **UP_SEQ_FEATURE** | COMPBIAS:Pro residues, DOMAIN:SEP, DOMAIN:UBX, REGION:3 X 8 AA tandem repeats of P-G-P-G-P-G-P-S, REGION:Disordered, REPEAT:1, REPEAT:2, REPEAT:3, | | |
| **WRAP73** | [**WD repeat containing, antisense to TP73(WRAP73)**](https://david.ncifcrf.gov/geneReportFull.jsp?rowids=49856) | [**Related Genes**](https://david.ncifcrf.gov/relatedGenes.jsp?id=49856) | [**Homo sapiens**](http://www.ncbi.nlm.nih.gov/Taxonomy/Browser/wwwtax.cgi?name=Homo%20sapiens) |
| **GOTERM_BP_DIRECT** | [mitotic sister chromatid segregation](http://www.ebi.ac.uk/QuickGO/GTerm?id=GO:0000070), [cell projection organization](http://www.ebi.ac.uk/QuickGO/GTerm?id=GO:0030030), [mitotic spindle assembly](http://www.ebi.ac.uk/QuickGO/GTerm?id=GO:0090307), [protein localization to mitotic spindle pole body](http://www.ebi.ac.uk/QuickGO/GTerm?id=GO:1902440), [positive regulation of non-motile cilium assembly](http://www.ebi.ac.uk/QuickGO/GTerm?id=GO:1902857), | | |
| **GOTERM_CC_DIRECT** | [cytoplasm](http://www.ebi.ac.uk/QuickGO/GTerm?id=GO:0005737), [centrosome](http://www.ebi.ac.uk/QuickGO/GTerm?id=GO:0005813), [centriole](http://www.ebi.ac.uk/QuickGO/GTerm?id=GO:0005814), [microtubule organizing center](http://www.ebi.ac.uk/QuickGO/GTerm?id=GO:0005815), [ciliary basal body](http://www.ebi.ac.uk/QuickGO/GTerm?id=GO:0036064), [mitotic spindle](http://www.ebi.ac.uk/QuickGO/GTerm?id=GO:0072686), [Msd1-Wdr8-Pkl1 complex](http://www.ebi.ac.uk/QuickGO/GTerm?id=GO:1990811), | | |
| **GOTERM_MF_DIRECT** | [protein binding](http://www.ebi.ac.uk/QuickGO/GTerm?id=GO:0005515), | | |
| **INTERPRO** | [WD40 repeat](https://www.ebi.ac.uk/interpro/entry/InterPro/IPR001680), [WD40/YVTN repeat-like-containing domain](https://www.ebi.ac.uk/interpro/entry/InterPro/IPR015943), | | |
| **SMART** | [WD40](http://smart.embl.de/smart/do_annotation.pl?DOMAIN=SM00320), | | |
| **UP_KW_BIOLOGICAL_PROCESS** | [Cilium biogenesis/degradation](http://www.uniprot.org/keywords/?query=KW-0970), | | |
| **UP_KW_CELLULAR_COMPONENT** | [Cytoskeleton](http://www.uniprot.org/keywords/?query=KW-0206), [Cytoplasm](http://www.uniprot.org/keywords/?query=KW-0963), | | |
| **UP_KW_DOMAIN** | [Repeat](http://www.uniprot.org/keywords/?query=KW-0677), [WD repeat](http://www.uniprot.org/keywords/?query=KW-0853), | | |
| **UP_KW_PTM** | [Phosphoprotein](http://www.uniprot.org/keywords/?query=KW-0597), | | |
| **UP_SEQ_FEATURE** | REPEAT:WD 1, REPEAT:WD 2, REPEAT:WD 3, REPEAT:WD 4, REPEAT:WD 5, REPEAT:WD 6, | | |
| **ACSM6** | [**acyl-CoA synthetase medium chain family member 6(ACSM6)**](https://david.ncifcrf.gov/geneReportFull.jsp?rowids=142827) | [**Related Genes**](https://david.ncifcrf.gov/relatedGenes.jsp?id=142827) | [**Homo sapiens**](http://www.ncbi.nlm.nih.gov/Taxonomy/Browser/wwwtax.cgi?name=Homo%20sapiens) |
| **GOTERM_BP_DIRECT** | [fatty acid biosynthetic process](http://www.ebi.ac.uk/QuickGO/GTerm?id=GO:0006633), [acyl-CoA metabolic process](http://www.ebi.ac.uk/QuickGO/GTerm?id=GO:0006637), | | |
| **GOTERM_CC_DIRECT** | [mitochondrial matrix](http://www.ebi.ac.uk/QuickGO/GTerm?id=GO:0005759), | | |
| **GOTERM_MF_DIRECT** | [fatty-acyl-CoA synthase activity](http://www.ebi.ac.uk/QuickGO/GTerm?id=GO:0004321), [ATP binding](http://www.ebi.ac.uk/QuickGO/GTerm?id=GO:0005524), [GTP binding](http://www.ebi.ac.uk/QuickGO/GTerm?id=GO:0005525), [fatty acid ligase activity](http://www.ebi.ac.uk/QuickGO/GTerm?id=GO:0015645), [medium-chain fatty acid-CoA ligase activity](http://www.ebi.ac.uk/QuickGO/GTerm?id=GO:0031956), [metal ion binding](http://www.ebi.ac.uk/QuickGO/GTerm?id=GO:0046872), [butyrate-CoA ligase activity](http://www.ebi.ac.uk/QuickGO/GTerm?id=GO:0047760), | | |
| **INTERPRO** | [AMP-dependent synthetase/ligase](https://www.ebi.ac.uk/interpro/entry/InterPro/IPR000873), | | |
| **KEGG_PATHWAY** | [Butanoate metabolism](https://david.ncifcrf.gov/kegg.jsp?path=hsa00650$Butanoate%20metabolism&termId=520047891&source=kegg), [Metabolic pathways](https://david.ncifcrf.gov/kegg.jsp?path=hsa01100$Metabolic%20pathways&termId=520047911&source=kegg), | | |
| **UP_KW_BIOLOGICAL_PROCESS** | [Fatty acid metabolism](http://www.uniprot.org/keywords/?query=KW-0276), [Lipid metabolism](http://www.uniprot.org/keywords/?query=KW-0443), | | |
| **UP_KW_CELLULAR_COMPONENT** | [Mitochondrion](http://www.uniprot.org/keywords/?query=KW-0496), | | |
| **UP_KW_DOMAIN** | [Transit peptide](http://www.uniprot.org/keywords/?query=KW-0809), | | |
| **UP_KW_LIGAND** | [ATP-binding](http://www.uniprot.org/keywords/?query=KW-0067), [GTP-binding](http://www.uniprot.org/keywords/?query=KW-0342), [Magnesium](http://www.uniprot.org/keywords/?query=KW-0460), [Metal-binding](http://www.uniprot.org/keywords/?query=KW-0479), [Nucleotide-binding](http://www.uniprot.org/keywords/?query=KW-0547), | | |
| **UP_KW_MOLECULAR_FUNCTION** | [Ligase](http://www.uniprot.org/keywords/?query=KW-0436), | | |
| **UP_SEQ_FEATURE** | BINDING:ATP, NP_BIND:ATP, TRANSIT:Mitochondrion, | | |
| **ADAR** | [**adenosine deaminase RNA specific(ADAR)**](https://david.ncifcrf.gov/geneReportFull.jsp?rowids=103) | [**Related Genes**](https://david.ncifcrf.gov/relatedGenes.jsp?id=103) | [**Homo sapiens**](http://www.ncbi.nlm.nih.gov/Taxonomy/Browser/wwwtax.cgi?name=Homo%20sapiens) |
| **BIOCARTA** | [RNA polymerase III transcription](https://david.ncifcrf.gov/biocarta.jsp?path=h_antisensePathway$RNA%20polymerase%20III%20transcription&termId=30000021&source=biocarta), | | |
| **GOTERM_BP_DIRECT** | [osteoblast differentiation](http://www.ebi.ac.uk/QuickGO/GTerm?id=GO:0001649), [hematopoietic progenitor cell differentiation](http://www.ebi.ac.uk/QuickGO/GTerm?id=GO:0002244), [somatic diversification of immune receptors via somatic mutation](http://www.ebi.ac.uk/QuickGO/GTerm?id=GO:0002566), [adenosine to inosine editing](http://www.ebi.ac.uk/QuickGO/GTerm?id=GO:0006382), [RNA processing](http://www.ebi.ac.uk/QuickGO/GTerm?id=GO:0006396), [mRNA processing](http://www.ebi.ac.uk/QuickGO/GTerm?id=GO:0006397), [protein import into nucleus](http://www.ebi.ac.uk/QuickGO/GTerm?id=GO:0006606), [protein export from nucleus](http://www.ebi.ac.uk/QuickGO/GTerm?id=GO:0006611), [response to virus](http://www.ebi.ac.uk/QuickGO/GTerm?id=GO:0009615), [base conversion or substitution editing](http://www.ebi.ac.uk/QuickGO/GTerm?id=GO:0016553), [erythrocyte differentiation](http://www.ebi.ac.uk/QuickGO/GTerm?id=GO:0030218), [pre-miRNA processing](http://www.ebi.ac.uk/QuickGO/GTerm?id=GO:0031054), [miRNA loading onto RISC involved in gene silencing by miRNA](http://www.ebi.ac.uk/QuickGO/GTerm?id=GO:0035280), [response to interferon-alpha](http://www.ebi.ac.uk/QuickGO/GTerm?id=GO:0035455), [negative regulation of apoptotic process](http://www.ebi.ac.uk/QuickGO/GTerm?id=GO:0043066), [negative regulation of protein kinase activity by regulation of protein phosphorylation](http://www.ebi.ac.uk/QuickGO/GTerm?id=GO:0044387), [positive regulation of viral genome replication](http://www.ebi.ac.uk/QuickGO/GTerm?id=GO:0045070), [innate immune response](http://www.ebi.ac.uk/QuickGO/GTerm?id=GO:0045087), [defense response to virus](http://www.ebi.ac.uk/QuickGO/GTerm?id=GO:0051607), [definitive hemopoiesis](http://www.ebi.ac.uk/QuickGO/GTerm?id=GO:0060216), [type I interferon signaling pathway](http://www.ebi.ac.uk/QuickGO/GTerm?id=GO:0060337), [negative regulation of type I interferon-mediated signaling pathway](http://www.ebi.ac.uk/QuickGO/GTerm?id=GO:0060339), [hematopoietic stem cell homeostasis](http://www.ebi.ac.uk/QuickGO/GTerm?id=GO:0061484), [cellular response to virus](http://www.ebi.ac.uk/QuickGO/GTerm?id=GO:0098586), [negative regulation of RNA interference](http://www.ebi.ac.uk/QuickGO/GTerm?id=GO:1900369), | | |
| **GOTERM_CC_DIRECT** | [nucleus](http://www.ebi.ac.uk/QuickGO/GTerm?id=GO:0005634), [nucleoplasm](http://www.ebi.ac.uk/QuickGO/GTerm?id=GO:0005654), [nucleolus](http://www.ebi.ac.uk/QuickGO/GTerm?id=GO:0005730), [cytoplasm](http://www.ebi.ac.uk/QuickGO/GTerm?id=GO:0005737), [membrane](http://www.ebi.ac.uk/QuickGO/GTerm?id=GO:0016020), [supraspliceosomal complex](http://www.ebi.ac.uk/QuickGO/GTerm?id=GO:0044530), | | |
| **GOTERM_MF_DIRECT** | [DNA binding](http://www.ebi.ac.uk/QuickGO/GTerm?id=GO:0003677), [RNA binding](http://www.ebi.ac.uk/QuickGO/GTerm?id=GO:0003723), [double-stranded RNA binding](http://www.ebi.ac.uk/QuickGO/GTerm?id=GO:0003725), [double-stranded RNA adenosine deaminase activity](http://www.ebi.ac.uk/QuickGO/GTerm?id=GO:0003726), [adenosine deaminase activity](http://www.ebi.ac.uk/QuickGO/GTerm?id=GO:0004000), [protein binding](http://www.ebi.ac.uk/QuickGO/GTerm?id=GO:0005515), [tRNA-specific adenosine deaminase activity](http://www.ebi.ac.uk/QuickGO/GTerm?id=GO:0008251), [metal ion binding](http://www.ebi.ac.uk/QuickGO/GTerm?id=GO:0046872), | | |
| **INTERPRO** | [Double-stranded RNA-specific adenosine deaminase (DRADA)](https://www.ebi.ac.uk/interpro/entry/InterPro/IPR000607), [Adenosine deaminase/editase](https://www.ebi.ac.uk/interpro/entry/InterPro/IPR002466), [Double-stranded RNA-binding-like domain](https://www.ebi.ac.uk/interpro/entry/InterPro/IPR014720), | | |
| **KEGG_PATHWAY** | [Cytosolic DNA-sensing pathway](https://david.ncifcrf.gov/kegg.jsp?path=hsa04623$Cytosolic%20DNA-sensing%20pathway&termId=520048010&source=kegg), [Measles](https://david.ncifcrf.gov/kegg.jsp?path=hsa05162$Measles&termId=520048118&source=kegg), [Influenza A](https://david.ncifcrf.gov/kegg.jsp?path=hsa05164$Influenza%20A&termId=520048120&source=kegg), [Coronavirus disease - COVID-19](https://david.ncifcrf.gov/kegg.jsp?path=hsa05171$Coronavirus%20disease%20-%20COVID-19&termId=520048127&source=kegg), | | |
| **OMIM_DISEASE** | [Dyschromatosis symmetrica hereditaria](http://omim.org/entry/127400), [Aicardi-Goutieres syndrome 6](http://omim.org/entry/615010), | | |
| **SMART** | [DSRM](http://smart.embl.de/smart/do_annotation.pl?DOMAIN=SM00358), [Zalpha](http://smart.embl.de/smart/do_annotation.pl?DOMAIN=SM00550), [ADEAMc](http://smart.embl.de/smart/do_annotation.pl?DOMAIN=SM00552), | | |
| **UP_KW_BIOLOGICAL_PROCESS** | [Antiviral defense](http://www.uniprot.org/keywords/?query=KW-0051), [Immunity](http://www.uniprot.org/keywords/?query=KW-0391), [Innate immunity](http://www.uniprot.org/keywords/?query=KW-0399), [mRNA processing](http://www.uniprot.org/keywords/?query=KW-0507), [RNA-mediated gene silencing](http://www.uniprot.org/keywords/?query=KW-0943), | | |
| **UP_KW_CELLULAR_COMPONENT** | [Nucleus](http://www.uniprot.org/keywords/?query=KW-0539), [Cytoplasm](http://www.uniprot.org/keywords/?query=KW-0963), | | |
| **UP_KW_DISEASE** | [Disease variant](http://www.uniprot.org/keywords/?query=KW-0225), [Aicardi-Goutieres syndrome](http://www.uniprot.org/keywords/?query=KW-0948), | | |
| **UP_KW_DOMAIN** | [Repeat](http://www.uniprot.org/keywords/?query=KW-0677), | | |
| **UP_KW_LIGAND** | [Metal-binding](http://www.uniprot.org/keywords/?query=KW-0479), [Zinc](http://www.uniprot.org/keywords/?query=KW-0862), | | |
| **UP_KW_MOLECULAR_FUNCTION** | [DNA-binding](http://www.uniprot.org/keywords/?query=KW-0238), [Hydrolase](http://www.uniprot.org/keywords/?query=KW-0378), [RNA-binding](http://www.uniprot.org/keywords/?query=KW-0694), | | |
| **UP_KW_PTM** | [Methylation](http://www.uniprot.org/keywords/?query=KW-0488), [Phosphoprotein](http://www.uniprot.org/keywords/?query=KW-0597), [Ubl conjugation](http://www.uniprot.org/keywords/?query=KW-0832), [Isopeptide bond](http://www.uniprot.org/keywords/?query=KW-1017), | | |
| **UP_SEQ_FEATURE** | ACT_SITE:Proton donor, COMPBIAS:Basic and acidic residues, COMPBIAS:Polar residues, CROSSLNK:Glycyl lysine isopeptide (Lys-Gly) (interchain with G-Cter in SUMO); alternate, CROSSLNK:Glycyl lysine isopeptide (Lys-Gly) (interchain with G-Cter in SUMO1); alternate, CROSSLNK:Glycyl lysine isopeptide (Lys-Gly) (interchain with G-Cter in SUMO2), CROSSLNK:Glycyl lysine isopeptide (Lys-Gly) (interchain with G-Cter in SUMO2); alternate, DOMAIN:A to I editase, DOMAIN:DRBM, DOMAIN:DRBM 1, DOMAIN:DRBM 2, DOMAIN:DRBM 3, DOMAIN:Z-binding, DOMAIN:Z-binding 1, DOMAIN:Z-binding 2, METAL:Zinc, MUTAGEN:E->A: No effect on nuclear location; when associated with A-721 and A-724., MUTAGEN:I->N: Disrupts the bi-partite nuclear localization signal and abolishes nuclear location; when associated with S-719 and N-723., MUTAGEN:K->R: Abolishes sumoylation., MUTAGEN:KK->AA: Strongly impaired RNA binding. No effect on nuclear location., MUTAGEN:KVRK->AVAA: No effect on nuclear location. No effect on RNA binding., MUTAGEN:L->N: Disrupts the bi-partite nuclear localization signal and abolishes nuclear location; when associated with N-716 and S-719., MUTAGEN:L->S: Disrupts the bi-partite nuclear localization signal and abolishes nuclear location; when associated with N-716 and N-723., MUTAGEN:MMP->AMA: Decreased nuclear and partially cytoplasmic location., MUTAGEN:Missing: Abolishes nuclear location., MUTAGEN:Missing: Disrupts nuclear localization signal. No effect on RNA binding., MUTAGEN:Missing: Disrupts the bi-partite nuclear localization signal and abolishes nuclear location., MUTAGEN:N->A: No effect on nuclear location; when associated with A-718 and A-721., MUTAGEN:R->A: Abolishes interaction with TNPO1, TNPO1-mediated nuclear import and nuclear location., MUTAGEN:R->A: No effect on nuclear location; when associated with A-721 and A-724., REGION:C-terminal extension of DRBM 3 and constituent of a bi-partite nuclear localization signal, REGION:Disordered, REGION:Interaction with Z-DNA, REGION:N-terminal extension of DRBM 3 and constituent of a bi-partite nuclear localization signal, | | |
| **ADCY7** | [**adenylate cyclase 7(ADCY7)**](https://david.ncifcrf.gov/geneReportFull.jsp?rowids=113) | [**Related Genes**](https://david.ncifcrf.gov/relatedGenes.jsp?id=113) | [**Homo sapiens**](http://www.ncbi.nlm.nih.gov/Taxonomy/Browser/wwwtax.cgi?name=Homo%20sapiens) |
| **GOTERM_BP_DIRECT** | [regulation of adaptive immune response](http://www.ebi.ac.uk/QuickGO/GTerm?id=GO:0002819), [negative regulation of inflammatory response to antigenic stimulus](http://www.ebi.ac.uk/QuickGO/GTerm?id=GO:0002862), [renal water homeostasis](http://www.ebi.ac.uk/QuickGO/GTerm?id=GO:0003091), [cAMP biosynthetic process](http://www.ebi.ac.uk/QuickGO/GTerm?id=GO:0006171), [G-protein coupled receptor signaling pathway](http://www.ebi.ac.uk/QuickGO/GTerm?id=GO:0007186), [adenylate cyclase-activating G-protein coupled receptor signaling pathway](http://www.ebi.ac.uk/QuickGO/GTerm?id=GO:0007189), [activation of adenylate cyclase activity](http://www.ebi.ac.uk/QuickGO/GTerm?id=GO:0007190), [adenylate cyclase-inhibiting G-protein coupled receptor signaling pathway](http://www.ebi.ac.uk/QuickGO/GTerm?id=GO:0007193), [cyclic nucleotide biosynthetic process](http://www.ebi.ac.uk/QuickGO/GTerm?id=GO:0009190), [activation of protein kinase A activity](http://www.ebi.ac.uk/QuickGO/GTerm?id=GO:0034199), [intracellular signal transduction](http://www.ebi.ac.uk/QuickGO/GTerm?id=GO:0035556), [maternal process involved in female pregnancy](http://www.ebi.ac.uk/QuickGO/GTerm?id=GO:0060135), [cellular response to lithium ion](http://www.ebi.ac.uk/QuickGO/GTerm?id=GO:0071285), [cellular response to ethanol](http://www.ebi.ac.uk/QuickGO/GTerm?id=GO:0071361), [cellular response to glucagon stimulus](http://www.ebi.ac.uk/QuickGO/GTerm?id=GO:0071377), [negative regulation of cytokine production involved in inflammatory response](http://www.ebi.ac.uk/QuickGO/GTerm?id=GO:1900016), | | |
| **GOTERM_CC_DIRECT** | [plasma membrane](http://www.ebi.ac.uk/QuickGO/GTerm?id=GO:0005886), [integral component of plasma membrane](http://www.ebi.ac.uk/QuickGO/GTerm?id=GO:0005887), [integral component of membrane](http://www.ebi.ac.uk/QuickGO/GTerm?id=GO:0016021), | | |
| **GOTERM_MF_DIRECT** | [adenylate cyclase activity](http://www.ebi.ac.uk/QuickGO/GTerm?id=GO:0004016), [ATP binding](http://www.ebi.ac.uk/QuickGO/GTerm?id=GO:0005524), [metal ion binding](http://www.ebi.ac.uk/QuickGO/GTerm?id=GO:0046872), | | |
| **INTERPRO** | [Adenylyl cyclase class-3/4/guanylyl cyclase](https://www.ebi.ac.uk/interpro/entry/InterPro/IPR001054), [Adenylate cyclase-like](https://www.ebi.ac.uk/interpro/entry/InterPro/IPR009398), [Adenylyl cyclase class-3/4/guanylyl cyclase, conserved site](https://www.ebi.ac.uk/interpro/entry/InterPro/IPR018297), | | |
| **KEGG_PATHWAY** | [Purine metabolism](https://david.ncifcrf.gov/kegg.jsp?path=hsa00230$Purine%20metabolism&termId=520047842&source=kegg), [Metabolic pathways](https://david.ncifcrf.gov/kegg.jsp?path=hsa01100$Metabolic%20pathways&termId=520047911&source=kegg), [Endocrine resistance](https://david.ncifcrf.gov/kegg.jsp?path=hsa01522$Endocrine%20resistance&termId=520047919&source=kegg), [Rap1 signaling pathway](https://david.ncifcrf.gov/kegg.jsp?path=hsa04015$Rap1%20signaling%20pathway&termId=520047944&source=kegg), [Calcium signaling pathway](https://david.ncifcrf.gov/kegg.jsp?path=hsa04020$Calcium%20signaling%20pathway&termId=520047945&source=kegg), [cGMP-PKG signaling pathway](https://david.ncifcrf.gov/kegg.jsp?path=hsa04022$cGMP-PKG%20signaling%20pathway&termId=520047946&source=kegg), [cAMP signaling pathway](https://david.ncifcrf.gov/kegg.jsp?path=hsa04024$cAMP%20signaling%20pathway&termId=520047947&source=kegg), [Chemokine signaling pathway](https://david.ncifcrf.gov/kegg.jsp?path=hsa04062$Chemokine%20signaling%20pathway&termId=520047950&source=kegg), [Phospholipase D signaling pathway](https://david.ncifcrf.gov/kegg.jsp?path=hsa04072$Phospholipase%20D%20signaling%20pathway&termId=520047956&source=kegg), [Oocyte meiosis](https://david.ncifcrf.gov/kegg.jsp?path=hsa04114$Oocyte%20meiosis&termId=520047959&source=kegg), [Longevity regulating pathway](https://david.ncifcrf.gov/kegg.jsp?path=hsa04211$Longevity%20regulating%20pathway&termId=520047976&source=kegg), [Longevity regulating pathway - multiple species](https://david.ncifcrf.gov/kegg.jsp?path=hsa04213$Longevity%20regulating%20pathway%20-%20multiple%20species&termId=520047977&source=kegg), [Adrenergic signaling in cardiomyocytes](https://david.ncifcrf.gov/kegg.jsp?path=hsa04261$Adrenergic%20signaling%20in%20cardiomyocytes&termId=520047983&source=kegg), [Vascular smooth muscle contraction](https://david.ncifcrf.gov/kegg.jsp?path=hsa04270$Vascular%20smooth%20muscle%20contraction&termId=520047984&source=kegg), [Apelin signaling pathway](https://david.ncifcrf.gov/kegg.jsp?path=hsa04371$Apelin%20signaling%20pathway&termId=520047991&source=kegg), [Gap junction](https://david.ncifcrf.gov/kegg.jsp?path=hsa04540$Gap%20junction&termId=520048000&source=kegg), [Platelet activation](https://david.ncifcrf.gov/kegg.jsp?path=hsa04611$Platelet%20activation&termId=520048003&source=kegg), [Circadian entrainment](https://david.ncifcrf.gov/kegg.jsp?path=hsa04713$Circadian%20entrainment&termId=520048026&source=kegg), [Thermogenesis](https://david.ncifcrf.gov/kegg.jsp?path=hsa04714$Thermogenesis&termId=520048027&source=kegg), [Retrograde endocannabinoid signaling](https://david.ncifcrf.gov/kegg.jsp?path=hsa04723$Retrograde%20endocannabinoid%20signaling&termId=520048031&source=kegg), [Glutamatergic synapse](https://david.ncifcrf.gov/kegg.jsp?path=hsa04724$Glutamatergic%20synapse&termId=520048032&source=kegg), [Cholinergic synapse](https://david.ncifcrf.gov/kegg.jsp?path=hsa04725$Cholinergic%20synapse&termId=520048033&source=kegg), [GABAergic synapse](https://david.ncifcrf.gov/kegg.jsp?path=hsa04727$GABAergic%20synapse&termId=520048035&source=kegg), [Inflammatory mediator regulation of TRP channels](https://david.ncifcrf.gov/kegg.jsp?path=hsa04750$Inflammatory%20mediator%20regulation%20of%20TRP%20channels&termId=520048041&source=kegg), [Insulin secretion](https://david.ncifcrf.gov/kegg.jsp?path=hsa04911$Insulin%20secretion&termId=520048044&source=kegg), [GnRH signaling pathway](https://david.ncifcrf.gov/kegg.jsp?path=hsa04912$GnRH%20signaling%20pathway&termId=520048045&source=kegg), [Ovarian steroidogenesis](https://david.ncifcrf.gov/kegg.jsp?path=hsa04913$Ovarian%20steroidogenesis&termId=520048046&source=kegg), [Progesterone-mediated oocyte maturation](https://david.ncifcrf.gov/kegg.jsp?path=hsa04914$Progesterone-mediated%20oocyte%20maturation&termId=520048047&source=kegg), [Estrogen signaling pathway](https://david.ncifcrf.gov/kegg.jsp?path=hsa04915$Estrogen%20signaling%20pathway&termId=520048048&source=kegg), [Melanogenesis](https://david.ncifcrf.gov/kegg.jsp?path=hsa04916$Melanogenesis&termId=520048049&source=kegg), [Thyroid hormone synthesis](https://david.ncifcrf.gov/kegg.jsp?path=hsa04918$Thyroid%20hormone%20synthesis&termId=520048051&source=kegg), [Oxytocin signaling pathway](https://david.ncifcrf.gov/kegg.jsp?path=hsa04921$Oxytocin%20signaling%20pathway&termId=520048054&source=kegg), [Regulation of lipolysis in adipocytes](https://david.ncifcrf.gov/kegg.jsp?path=hsa04923$Regulation%20of%20lipolysis%20in%20adipocytes&termId=520048056&source=kegg), [Aldosterone synthesis and secretion](https://david.ncifcrf.gov/kegg.jsp?path=hsa04925$Aldosterone%20synthesis%20and%20secretion&termId=520048058&source=kegg), [Relaxin signaling pathway](https://david.ncifcrf.gov/kegg.jsp?path=hsa04926$Relaxin%20signaling%20pathway&termId=520048059&source=kegg), [Cortisol synthesis and secretion](https://david.ncifcrf.gov/kegg.jsp?path=hsa04927$Cortisol%20synthesis%20and%20secretion&termId=520048060&source=kegg), [Parathyroid hormone synthesis, secretion and action](https://david.ncifcrf.gov/kegg.jsp?path=hsa04928$Parathyroid%20hormone%20synthesis,%20secretion%20and%20action&termId=520048061&source=kegg), [Cushing syndrome](https://david.ncifcrf.gov/kegg.jsp?path=hsa04934$Cushing%20syndrome&termId=520048067&source=kegg), [Growth hormone synthesis, secretion and action](https://david.ncifcrf.gov/kegg.jsp?path=hsa04935$Growth%20hormone%20synthesis,%20secretion%20and%20action&termId=520048068&source=kegg), [Salivary secretion](https://david.ncifcrf.gov/kegg.jsp?path=hsa04970$Salivary%20secretion&termId=520048077&source=kegg), [Gastric acid secretion](https://david.ncifcrf.gov/kegg.jsp?path=hsa04971$Gastric%20acid%20secretion&termId=520048078&source=kegg), [Pancreatic secretion](https://david.ncifcrf.gov/kegg.jsp?path=hsa04972$Pancreatic%20secretion&termId=520048079&source=kegg), [Bile secretion](https://david.ncifcrf.gov/kegg.jsp?path=hsa04976$Bile%20secretion&termId=520048083&source=kegg), [Morphine addiction](https://david.ncifcrf.gov/kegg.jsp?path=hsa05032$Morphine%20addiction&termId=520048096&source=kegg), [Human cytomegalovirus infection](https://david.ncifcrf.gov/kegg.jsp?path=hsa05163$Human%20cytomegalovirus%20infection&termId=520048119&source=kegg), [Human T-cell leukemia virus 1 infection](https://david.ncifcrf.gov/kegg.jsp?path=hsa05166$Human%20T-cell%20leukemia%20virus%201%20infection&termId=520048122&source=kegg), [Pathways in cancer](https://david.ncifcrf.gov/kegg.jsp?path=hsa05200$Pathways%20in%20cancer&termId=520048128&source=kegg), [Chemical carcinogenesis - receptor activation](https://david.ncifcrf.gov/kegg.jsp?path=hsa05207$Chemical%20carcinogenesis%20-%20receptor%20activation&termId=520048134&source=kegg), [Dilated cardiomyopathy](https://david.ncifcrf.gov/kegg.jsp?path=hsa05414$Dilated%20cardiomyopathy&termId=520048166&source=kegg), | | |
| **SMART** | [CYCc](http://smart.embl.de/smart/do_annotation.pl?DOMAIN=SM00044), | | |
| **UP_KW_BIOLOGICAL_PROCESS** | [cAMP biosynthesis](http://www.uniprot.org/keywords/?query=KW-0115), | | |
| **UP_KW_CELLULAR_COMPONENT** | [Membrane](http://www.uniprot.org/keywords/?query=KW-0472), | | |
| **UP_KW_DOMAIN** | [Repeat](http://www.uniprot.org/keywords/?query=KW-0677), [Transmembrane](http://www.uniprot.org/keywords/?query=KW-0812), [Transmembrane helix](http://www.uniprot.org/keywords/?query=KW-1133), | | |
| **UP_KW_LIGAND** | [ATP-binding](http://www.uniprot.org/keywords/?query=KW-0067), [Magnesium](http://www.uniprot.org/keywords/?query=KW-0460), [Manganese](http://www.uniprot.org/keywords/?query=KW-0464), [Metal-binding](http://www.uniprot.org/keywords/?query=KW-0479), [Nucleotide-binding](http://www.uniprot.org/keywords/?query=KW-0547), | | |
| **UP_KW_MOLECULAR_FUNCTION** | [Lyase](http://www.uniprot.org/keywords/?query=KW-0456), | | |
| **UP_KW_PTM** | [Glycoprotein](http://www.uniprot.org/keywords/?query=KW-0325), | | |
| **UP_SEQ_FEATURE** | BINDING:ATP, CARBOHYD:N-linked (GlcNAc...) asparagine, COMPBIAS:Basic and acidic residues, DOMAIN:Guanylate cyclase, DOMAIN:Guanylate cyclase 1, DOMAIN:Guanylate cyclase 2, METAL:Magnesium 1; catalytic, METAL:Magnesium 2; catalytic, METAL:Magnesium 2; via carbonyl oxygen; catalytic, MUTAGEN:ARPFAH->NAAIRS: Does not affect cAMP biosynthetic process in response to C5 alpha chain stimulation. Reduces by 40?60% cAMP biosynthetic process in response to sphingosine 1-phosphate stimulation., MUTAGEN:KMRASV->NAAIRS: Does not affect cAMP biosynthetic process in response to sphingosine 1-phosphate stimulation. Reduces by 40% cAMP biosynthetic process in response to C5 alpha chain stimulation., MUTAGEN:SKSDDF->NAAIRS: Reduces cAMP biosynthetic process in response to C5 alpha chain stimulation and more severely in response to sphingosine 1-phosphate stimulation., MUTAGEN:TRYLE->NAAIR: Reduces cAMP biosynthetic process in response to C5 alpha chain stimulation and more severely in response to sphingosine 1-phosphate stimulation., MUTAGEN:WGAARP->NAAIRS: Does not affect cAMP biosynthetic process in response to C5 alpha chain stimulation. Reduces by 40?60% cAMP biosynthetic process in response to sphingosine 1-phosphate stimulation., NP_BIND:ATP, REGION:Disordered, REGION:Mediates regulation of adenylate cyclase activity by C5 alpha-induced G- beta and gamma pathway, REGION:Mediates regulation of adenylate cyclase activity by sphingosine 1-phosphate-induced G alpha 13 pathway, REGION:Modulates adenylate cyclase activity by modulating the binding of G(s)alpha to the high-affinity G(s)alpha binding site in 7C1a/7C2, TOPO_DOM:Cytoplasmic, TRANSMEM:Helical, | | |
| **ADGRD1** | [**adhesion G protein-coupled receptor D1(ADGRD1)**](https://david.ncifcrf.gov/geneReportFull.jsp?rowids=283383) | [**Related Genes**](https://david.ncifcrf.gov/relatedGenes.jsp?id=283383) | [**Homo sapiens**](http://www.ncbi.nlm.nih.gov/Taxonomy/Browser/wwwtax.cgi?name=Homo%20sapiens) |
| **GOTERM_BP_DIRECT** | [cell surface receptor signaling pathway](http://www.ebi.ac.uk/QuickGO/GTerm?id=GO:0007166), [G-protein coupled receptor signaling pathway](http://www.ebi.ac.uk/QuickGO/GTerm?id=GO:0007186), [adenylate cyclase-activating G-protein coupled receptor signaling pathway](http://www.ebi.ac.uk/QuickGO/GTerm?id=GO:0007189), | | |
| **GOTERM_CC_DIRECT** | [nucleoplasm](http://www.ebi.ac.uk/QuickGO/GTerm?id=GO:0005654), [plasma membrane](http://www.ebi.ac.uk/QuickGO/GTerm?id=GO:0005886), [integral component of plasma membrane](http://www.ebi.ac.uk/QuickGO/GTerm?id=GO:0005887), [integral component of membrane](http://www.ebi.ac.uk/QuickGO/GTerm?id=GO:0016021), | | |
| **GOTERM_MF_DIRECT** | [G-protein coupled receptor activity](http://www.ebi.ac.uk/QuickGO/GTerm?id=GO:0004930), [protein binding](http://www.ebi.ac.uk/QuickGO/GTerm?id=GO:0005515), | | |
| **INTERPRO** | [GPS domain](https://www.ebi.ac.uk/interpro/entry/InterPro/IPR000203), [GPCR, family 2, secretin-like](https://www.ebi.ac.uk/interpro/entry/InterPro/IPR000832), [Pentaxin](https://www.ebi.ac.uk/interpro/entry/InterPro/IPR001759), [Concanavalin A-like lectin/glucanase, subgroup](https://www.ebi.ac.uk/interpro/entry/InterPro/IPR013320), [GPCR, family 2-like](https://www.ebi.ac.uk/interpro/entry/InterPro/IPR017981), [GPCR, family 2, secretin-like, conserved site](https://www.ebi.ac.uk/interpro/entry/InterPro/IPR017983), | | |
| **SMART** | [GPS](http://smart.embl.de/smart/do_annotation.pl?DOMAIN=SM00303), | | |
| **UP_KW_CELLULAR_COMPONENT** | [Membrane](http://www.uniprot.org/keywords/?query=KW-0472), [Cell membrane](http://www.uniprot.org/keywords/?query=KW-1003), | | |
| **UP_KW_DOMAIN** | [Signal](http://www.uniprot.org/keywords/?query=KW-0732), [Transmembrane](http://www.uniprot.org/keywords/?query=KW-0812), [Transmembrane helix](http://www.uniprot.org/keywords/?query=KW-1133), | | |
| **UP_KW_MOLECULAR_FUNCTION** | [G-protein coupled receptor](http://www.uniprot.org/keywords/?query=KW-0297), [Receptor](http://www.uniprot.org/keywords/?query=KW-0675), [Transducer](http://www.uniprot.org/keywords/?query=KW-0807), | | |
| **UP_KW_PTM** | [Glycoprotein](http://www.uniprot.org/keywords/?query=KW-0325), | | |
| **UP_SEQ_FEATURE** | CARBOHYD:N-linked (GlcNAc...) asparagine, DOMAIN:GPS, DOMAIN:G_PROTEIN_RECEP_F2_4, DOMAIN:Pentraxin (PTX), MOTIF:Stachel, MUTAGEN:H->R: Does not affect membrane trafficking and basal activity., MUTAGEN:L->A: Abolishes G-protein coupled receptor activity; does not affect subcellular location., MUTAGEN:L->T: Does not affect membrane trafficking. Abolishes receptor activity., MUTAGEN:M->A: Abolishes G-protein coupled receptor activity; does not affect subcellular location., MUTAGEN:T->A: No effect on G-protein coupled receptor activity; does not affect subcellular location., MUTAGEN:V->A: Abolishes G-protein coupled receptor activity; does not affect subcellular location., REGION:Disordered, TOPO_DOM:Cytoplasmic, TOPO_DOM:Extracellular, TRANSMEM:Helical, TRANSMEM:Helical; Name=1, TRANSMEM:Helical; Name=2, TRANSMEM:Helical; Name=3, TRANSMEM:Helical; Name=4, TRANSMEM:Helical; Name=5, TRANSMEM:Helical; Name=6, TRANSMEM:Helical; Name=7, | | |
| **ADGRE3** | [**adhesion G protein-coupled receptor E3(ADGRE3)**](https://david.ncifcrf.gov/geneReportFull.jsp?rowids=84658) | [**Related Genes**](https://david.ncifcrf.gov/relatedGenes.jsp?id=84658) | [**Homo sapiens**](http://www.ncbi.nlm.nih.gov/Taxonomy/Browser/wwwtax.cgi?name=Homo%20sapiens) |
| **GOTERM_BP_DIRECT** | [cell surface receptor signaling pathway](http://www.ebi.ac.uk/QuickGO/GTerm?id=GO:0007166), [G-protein coupled receptor signaling pathway](http://www.ebi.ac.uk/QuickGO/GTerm?id=GO:0007186), [adenylate cyclase-activating G-protein coupled receptor signaling pathway](http://www.ebi.ac.uk/QuickGO/GTerm?id=GO:0007189), [neutrophil degranulation](http://www.ebi.ac.uk/QuickGO/GTerm?id=GO:0043312), | | |
| **GOTERM_CC_DIRECT** | [extracellular region](http://www.ebi.ac.uk/QuickGO/GTerm?id=GO:0005576), [plasma membrane](http://www.ebi.ac.uk/QuickGO/GTerm?id=GO:0005886), [integral component of plasma membrane](http://www.ebi.ac.uk/QuickGO/GTerm?id=GO:0005887), [integral component of membrane](http://www.ebi.ac.uk/QuickGO/GTerm?id=GO:0016021), [secretory granule membrane](http://www.ebi.ac.uk/QuickGO/GTerm?id=GO:0030667), [ficolin-1-rich granule membrane](http://www.ebi.ac.uk/QuickGO/GTerm?id=GO:0101003), | | |
| **GOTERM_MF_DIRECT** | [G-protein coupled receptor activity](http://www.ebi.ac.uk/QuickGO/GTerm?id=GO:0004930), [calcium ion binding](http://www.ebi.ac.uk/QuickGO/GTerm?id=GO:0005509), | | |
| **INTERPRO** | [EGF-type aspartate/asparagine hydroxylation site](https://www.ebi.ac.uk/interpro/entry/InterPro/IPR000152), [GPS domain](https://www.ebi.ac.uk/interpro/entry/InterPro/IPR000203), [Epidermal growth factor-like domain](https://www.ebi.ac.uk/interpro/entry/InterPro/IPR000742), [GPCR, family 2, secretin-like](https://www.ebi.ac.uk/interpro/entry/InterPro/IPR000832), [GPCR, family 2, EMR1 hormone receptor](https://www.ebi.ac.uk/interpro/entry/InterPro/IPR001740), [EGF-like calcium-binding](https://www.ebi.ac.uk/interpro/entry/InterPro/IPR001881), [GPCR, family 2, CD97 antigen](https://www.ebi.ac.uk/interpro/entry/InterPro/IPR003056), [Insulin-like growth factor binding protein, N-terminal](https://www.ebi.ac.uk/interpro/entry/InterPro/IPR009030), [GPCR, family 2-like](https://www.ebi.ac.uk/interpro/entry/InterPro/IPR017981), [GPCR, family 2, secretin-like, conserved site](https://www.ebi.ac.uk/interpro/entry/InterPro/IPR017983), [EGF-like calcium-binding, conserved site](https://www.ebi.ac.uk/interpro/entry/InterPro/IPR018097), | | |
| **SMART** | [EGF_CA](http://smart.embl.de/smart/do_annotation.pl?DOMAIN=SM00179), [EGF](http://smart.embl.de/smart/do_annotation.pl?DOMAIN=SM00181), [GPS](http://smart.embl.de/smart/do_annotation.pl?DOMAIN=SM00303), | | |
| **UP_KW_CELLULAR_COMPONENT** | [Membrane](http://www.uniprot.org/keywords/?query=KW-0472), [Secreted](http://www.uniprot.org/keywords/?query=KW-0964), [Cell membrane](http://www.uniprot.org/keywords/?query=KW-1003), | | |
| **UP_KW_DOMAIN** | [EGF-like domain](http://www.uniprot.org/keywords/?query=KW-0245), [Repeat](http://www.uniprot.org/keywords/?query=KW-0677), [Signal](http://www.uniprot.org/keywords/?query=KW-0732), [Transmembrane](http://www.uniprot.org/keywords/?query=KW-0812), [Transmembrane helix](http://www.uniprot.org/keywords/?query=KW-1133), | | |
| **UP_KW_LIGAND** | [Calcium](http://www.uniprot.org/keywords/?query=KW-0106), | | |
| **UP_KW_MOLECULAR_FUNCTION** | [G-protein coupled receptor](http://www.uniprot.org/keywords/?query=KW-0297), [Receptor](http://www.uniprot.org/keywords/?query=KW-0675), [Transducer](http://www.uniprot.org/keywords/?query=KW-0807), | | |
| **UP_KW_PTM** | [Glycoprotein](http://www.uniprot.org/keywords/?query=KW-0325), [Disulfide bond](http://www.uniprot.org/keywords/?query=KW-1015), | | |
| **UP_SEQ_FEATURE** | CARBOHYD:N-linked (GlcNAc...) asparagine, DOMAIN:EGF-like, DOMAIN:EGF-like 1, DOMAIN:EGF-like 2; calcium-binding, DOMAIN:GPS, DOMAIN:G_PROTEIN_RECEP_F2_4, REGION:Disordered, SITE:Cleavage, TOPO_DOM:Cytoplasmic, TOPO_DOM:Extracellular, TRANSMEM:Helical, TRANSMEM:Helical; Name=1, TRANSMEM:Helical; Name=2, TRANSMEM:Helical; Name=3, TRANSMEM:Helical; Name=4, TRANSMEM:Helical; Name=5, TRANSMEM:Helical; Name=6, TRANSMEM:Helical; Name=7, | | |
| **ADGRV1** | [**adhesion G protein-coupled receptor V1(ADGRV1)**](https://david.ncifcrf.gov/geneReportFull.jsp?rowids=84059) | [**Related Genes**](https://david.ncifcrf.gov/relatedGenes.jsp?id=84059) | [**Homo sapiens**](http://www.ncbi.nlm.nih.gov/Taxonomy/Browser/wwwtax.cgi?name=Homo%20sapiens) |
| **GOTERM_BP_DIRECT** | [cell communication](http://www.ebi.ac.uk/QuickGO/GTerm?id=GO:0007154), [cell surface receptor signaling pathway](http://www.ebi.ac.uk/QuickGO/GTerm?id=GO:0007166), [G-protein coupled receptor signaling pathway](http://www.ebi.ac.uk/QuickGO/GTerm?id=GO:0007186), [negative regulation of adenylate cyclase activity](http://www.ebi.ac.uk/QuickGO/GTerm?id=GO:0007194), [nervous system development](http://www.ebi.ac.uk/QuickGO/GTerm?id=GO:0007399), [visual perception](http://www.ebi.ac.uk/QuickGO/GTerm?id=GO:0007601), [sensory perception of sound](http://www.ebi.ac.uk/QuickGO/GTerm?id=GO:0007605), [positive regulation of protein kinase A signaling](http://www.ebi.ac.uk/QuickGO/GTerm?id=GO:0010739), [positive regulation of bone mineralization](http://www.ebi.ac.uk/QuickGO/GTerm?id=GO:0030501), [regulation of protein stability](http://www.ebi.ac.uk/QuickGO/GTerm?id=GO:0031647), [establishment of protein localization](http://www.ebi.ac.uk/QuickGO/GTerm?id=GO:0045184), [photoreceptor cell maintenance](http://www.ebi.ac.uk/QuickGO/GTerm?id=GO:0045494), [maintenance of animal organ identity](http://www.ebi.ac.uk/QuickGO/GTerm?id=GO:0048496), [inner ear development](http://www.ebi.ac.uk/QuickGO/GTerm?id=GO:0048839), [neurological system process](http://www.ebi.ac.uk/QuickGO/GTerm?id=GO:0050877), [detection of mechanical stimulus involved in sensory perception of sound](http://www.ebi.ac.uk/QuickGO/GTerm?id=GO:0050910), [sensory perception of light stimulus](http://www.ebi.ac.uk/QuickGO/GTerm?id=GO:0050953), [inner ear receptor stereocilium organization](http://www.ebi.ac.uk/QuickGO/GTerm?id=GO:0060122), [cellular response to calcium ion](http://www.ebi.ac.uk/QuickGO/GTerm?id=GO:0071277), [positive regulation of protein kinase C signaling](http://www.ebi.ac.uk/QuickGO/GTerm?id=GO:0090037), [self proteolysis](http://www.ebi.ac.uk/QuickGO/GTerm?id=GO:0097264), [cell-cell adhesion](http://www.ebi.ac.uk/QuickGO/GTerm?id=GO:0098609), | | |
| **GOTERM_CC_DIRECT** | [photoreceptor inner segment](http://www.ebi.ac.uk/QuickGO/GTerm?id=GO:0001917), [stereocilia ankle link](http://www.ebi.ac.uk/QuickGO/GTerm?id=GO:0002141), [stereocilia ankle link complex](http://www.ebi.ac.uk/QuickGO/GTerm?id=GO:0002142), [cytoplasm](http://www.ebi.ac.uk/QuickGO/GTerm?id=GO:0005737), [plasma membrane](http://www.ebi.ac.uk/QuickGO/GTerm?id=GO:0005886), [cell surface](http://www.ebi.ac.uk/QuickGO/GTerm?id=GO:0009986), [membrane](http://www.ebi.ac.uk/QuickGO/GTerm?id=GO:0016020), [integral component of membrane](http://www.ebi.ac.uk/QuickGO/GTerm?id=GO:0016021), [receptor complex](http://www.ebi.ac.uk/QuickGO/GTerm?id=GO:0043235), [synapse](http://www.ebi.ac.uk/QuickGO/GTerm?id=GO:0045202), [stereocilium membrane](http://www.ebi.ac.uk/QuickGO/GTerm?id=GO:0060171), [extracellular exosome](http://www.ebi.ac.uk/QuickGO/GTerm?id=GO:0070062), [periciliary membrane compartment](http://www.ebi.ac.uk/QuickGO/GTerm?id=GO:1990075), [USH2 complex](http://www.ebi.ac.uk/QuickGO/GTerm?id=GO:1990696), | | |
| **GOTERM_MF_DIRECT** | [G-protein alpha-subunit binding](http://www.ebi.ac.uk/QuickGO/GTerm?id=GO:0001965), [G-protein coupled receptor activity](http://www.ebi.ac.uk/QuickGO/GTerm?id=GO:0004930), [calcium ion binding](http://www.ebi.ac.uk/QuickGO/GTerm?id=GO:0005509), [protein binding](http://www.ebi.ac.uk/QuickGO/GTerm?id=GO:0005515), [adenylate cyclase inhibitor activity](http://www.ebi.ac.uk/QuickGO/GTerm?id=GO:0010855), [hydrolase activity](http://www.ebi.ac.uk/QuickGO/GTerm?id=GO:0016787), | | |
| **INTERPRO** | [GPS domain](https://www.ebi.ac.uk/interpro/entry/InterPro/IPR000203), [GPCR, family 2, secretin-like](https://www.ebi.ac.uk/interpro/entry/InterPro/IPR000832), [Na-Ca exchanger/integrin-beta4](https://www.ebi.ac.uk/interpro/entry/InterPro/IPR003644), [Leucine-rich glioma-inactivated , EPTP repeat](https://www.ebi.ac.uk/interpro/entry/InterPro/IPR005492), [EAR](https://www.ebi.ac.uk/interpro/entry/InterPro/IPR009039), [Concanavalin A-like lectin/glucanase, subgroup](https://www.ebi.ac.uk/interpro/entry/InterPro/IPR013320), [GPCR, family 2-like](https://www.ebi.ac.uk/interpro/entry/InterPro/IPR017981), [G protein-coupled receptor 98](https://www.ebi.ac.uk/interpro/entry/InterPro/IPR026919), | | |
| **OMIM_DISEASE** | [Febrile seizures, familial, 4](http://omim.org/entry/604352), [Usher syndrome, type 2C](http://omim.org/entry/605472), [Usher syndrome, type 2C, GPR98/PDZD7 digenic](http://omim.org/entry/605472), | | |
| **SMART** | [Calx_beta](http://smart.embl.de/smart/do_annotation.pl?DOMAIN=SM00237), | | |
| **UP_KW_BIOLOGICAL_PROCESS** | [Sensory transduction](http://www.uniprot.org/keywords/?query=KW-0716), [Vision](http://www.uniprot.org/keywords/?query=KW-0844), | | |
| **UP_KW_CELLULAR_COMPONENT** | [Membrane](http://www.uniprot.org/keywords/?query=KW-0472), [Cell projection](http://www.uniprot.org/keywords/?query=KW-0966), [Cell membrane](http://www.uniprot.org/keywords/?query=KW-1003), | | |
| **UP_KW_DISEASE** | [Deafness](http://www.uniprot.org/keywords/?query=KW-0209), [Retinitis pigmentosa](http://www.uniprot.org/keywords/?query=KW-0682), [Usher syndrome](http://www.uniprot.org/keywords/?query=KW-0836), | | |
| **UP_KW_DOMAIN** | [Repeat](http://www.uniprot.org/keywords/?query=KW-0677), [Signal](http://www.uniprot.org/keywords/?query=KW-0732), [Transmembrane](http://www.uniprot.org/keywords/?query=KW-0812), [Transmembrane helix](http://www.uniprot.org/keywords/?query=KW-1133), | | |
| **UP_KW_LIGAND** | [Calcium](http://www.uniprot.org/keywords/?query=KW-0106), | | |
| **UP_KW_MOLECULAR_FUNCTION** | [G-protein coupled receptor](http://www.uniprot.org/keywords/?query=KW-0297), [Hydrolase](http://www.uniprot.org/keywords/?query=KW-0378), [Receptor](http://www.uniprot.org/keywords/?query=KW-0675), [Transducer](http://www.uniprot.org/keywords/?query=KW-0807), | | |
| **UP_SEQ_FEATURE** | COMPBIAS:Polar residues, DOMAIN:Calx-beta, DOMAIN:Calx-beta 1, DOMAIN:Calx-beta 10, DOMAIN:Calx-beta 11, DOMAIN:Calx-beta 12, DOMAIN:Calx-beta 13, DOMAIN:Calx-beta 14, DOMAIN:Calx-beta 15, DOMAIN:Calx-beta 16, DOMAIN:Calx-beta 17, DOMAIN:Calx-beta 18, DOMAIN:Calx-beta 19, DOMAIN:Calx-beta 2, DOMAIN:Calx-beta 20, DOMAIN:Calx-beta 21, DOMAIN:Calx-beta 22, DOMAIN:Calx-beta 23, DOMAIN:Calx-beta 24, DOMAIN:Calx-beta 25, DOMAIN:Calx-beta 26, DOMAIN:Calx-beta 27, DOMAIN:Calx-beta 28, DOMAIN:Calx-beta 29, DOMAIN:Calx-beta 3, DOMAIN:Calx-beta 30, DOMAIN:Calx-beta 31, DOMAIN:Calx-beta 32, DOMAIN:Calx-beta 33, DOMAIN:Calx-beta 34, DOMAIN:Calx-beta 35, DOMAIN:Calx-beta 4, DOMAIN:Calx-beta 5, DOMAIN:Calx-beta 6, DOMAIN:Calx-beta 7, DOMAIN:Calx-beta 8, DOMAIN:Calx-beta 9, DOMAIN:GPS, DOMAIN:G_PROTEIN_RECEP_F2_4, REGION:Disordered, REPEAT:EAR 1, REPEAT:EAR 2, REPEAT:EAR 3, REPEAT:EAR 4, REPEAT:EAR 5, REPEAT:EAR 6, SITE:Cleavage, TOPO_DOM:Cytoplasmic, TOPO_DOM:Extracellular, TRANSMEM:Helical, | | |
| **ADIPOR2** | [**adiponectin receptor 2(ADIPOR2)**](https://david.ncifcrf.gov/geneReportFull.jsp?rowids=79602) | [**Related Genes**](https://david.ncifcrf.gov/relatedGenes.jsp?id=79602) | [**Homo sapiens**](http://www.ncbi.nlm.nih.gov/Taxonomy/Browser/wwwtax.cgi?name=Homo%20sapiens) |
| **GOTERM_BP_DIRECT** | [positive regulation of protein phosphorylation](http://www.ebi.ac.uk/QuickGO/GTerm?id=GO:0001934), [heart development](http://www.ebi.ac.uk/QuickGO/GTerm?id=GO:0007507), [female pregnancy](http://www.ebi.ac.uk/QuickGO/GTerm?id=GO:0007565), [response to nutrient](http://www.ebi.ac.uk/QuickGO/GTerm?id=GO:0007584), [response to fructose](http://www.ebi.ac.uk/QuickGO/GTerm?id=GO:0009750), [hormone-mediated signaling pathway](http://www.ebi.ac.uk/QuickGO/GTerm?id=GO:0009755), [negative regulation of gene expression](http://www.ebi.ac.uk/QuickGO/GTerm?id=GO:0010629), [response to amine](http://www.ebi.ac.uk/QuickGO/GTerm?id=GO:0014075), [fatty acid oxidation](http://www.ebi.ac.uk/QuickGO/GTerm?id=GO:0019395), [negative regulation of cell growth](http://www.ebi.ac.uk/QuickGO/GTerm?id=GO:0030308), [response to lipopolysaccharide](http://www.ebi.ac.uk/QuickGO/GTerm?id=GO:0032496), [adiponectin-activated signaling pathway](http://www.ebi.ac.uk/QuickGO/GTerm?id=GO:0033211), [regulation of fatty acid biosynthetic process](http://www.ebi.ac.uk/QuickGO/GTerm?id=GO:0042304), [response to drug](http://www.ebi.ac.uk/QuickGO/GTerm?id=GO:0042493), [glucose homeostasis](http://www.ebi.ac.uk/QuickGO/GTerm?id=GO:0042593), [response to ethanol](http://www.ebi.ac.uk/QuickGO/GTerm?id=GO:0045471), [positive regulation of glucose import](http://www.ebi.ac.uk/QuickGO/GTerm?id=GO:0046326), [vascular wound healing](http://www.ebi.ac.uk/QuickGO/GTerm?id=GO:0061042), [cellular response to fatty acid](http://www.ebi.ac.uk/QuickGO/GTerm?id=GO:0071398), | | |
| **GOTERM_CC_DIRECT** | [plasma membrane](http://www.ebi.ac.uk/QuickGO/GTerm?id=GO:0005886), [integral component of membrane](http://www.ebi.ac.uk/QuickGO/GTerm?id=GO:0016021), [intrinsic component of plasma membrane](http://www.ebi.ac.uk/QuickGO/GTerm?id=GO:0031226), | | |
| **GOTERM_MF_DIRECT** | [protein binding](http://www.ebi.ac.uk/QuickGO/GTerm?id=GO:0005515), [signaling receptor activity](http://www.ebi.ac.uk/QuickGO/GTerm?id=GO:0038023), [identical protein binding](http://www.ebi.ac.uk/QuickGO/GTerm?id=GO:0042802), [metal ion binding](http://www.ebi.ac.uk/QuickGO/GTerm?id=GO:0046872), [adiponectin binding](http://www.ebi.ac.uk/QuickGO/GTerm?id=GO:0055100), [adipokinetic hormone receptor activity](http://www.ebi.ac.uk/QuickGO/GTerm?id=GO:0097003), | | |
| **INTERPRO** | [Hly-III-related](https://www.ebi.ac.uk/interpro/entry/InterPro/IPR004254), | | |
| **KEGG_PATHWAY** | [AMPK signaling pathway](https://david.ncifcrf.gov/kegg.jsp?path=hsa04152$AMPK%20signaling%20pathway&termId=520047974&source=kegg), [Longevity regulating pathway](https://david.ncifcrf.gov/kegg.jsp?path=hsa04211$Longevity%20regulating%20pathway&termId=520047976&source=kegg), [Adipocytokine signaling pathway](https://david.ncifcrf.gov/kegg.jsp?path=hsa04920$Adipocytokine%20signaling%20pathway&termId=520048053&source=kegg), [Non-alcoholic fatty liver disease](https://david.ncifcrf.gov/kegg.jsp?path=hsa04932$Non-alcoholic%20fatty%20liver%20disease&termId=520048065&source=kegg), [Alcoholic liver disease](https://david.ncifcrf.gov/kegg.jsp?path=hsa04936$Alcoholic%20liver%20disease&termId=520048069&source=kegg), | | |
| **UP_KW_BIOLOGICAL_PROCESS** | [Fatty acid metabolism](http://www.uniprot.org/keywords/?query=KW-0276), [Lipid metabolism](http://www.uniprot.org/keywords/?query=KW-0443), | | |
| **UP_KW_CELLULAR_COMPONENT** | [Membrane](http://www.uniprot.org/keywords/?query=KW-0472), [Cell membrane](http://www.uniprot.org/keywords/?query=KW-1003), | | |
| **UP_KW_DOMAIN** | [Transmembrane](http://www.uniprot.org/keywords/?query=KW-0812), [Transmembrane helix](http://www.uniprot.org/keywords/?query=KW-1133), | | |
| **UP_KW_LIGAND** | [Metal-binding](http://www.uniprot.org/keywords/?query=KW-0479), [Zinc](http://www.uniprot.org/keywords/?query=KW-0862), | | |
| **UP_KW_MOLECULAR_FUNCTION** | [Receptor](http://www.uniprot.org/keywords/?query=KW-0675), | | |
| **UP_SEQ_FEATURE** | COMPBIAS:Basic and acidic residues, METAL:Zinc, MUTAGEN:D->A: Impairs response to ADIPOQ binding. Abolishes response to ADIPOQ binding; when associated with A-202; A-348 and A-352., MUTAGEN:H->A: Abolishes response to ADIPOQ binding; when associated with A-202; A-219 and A-348., MUTAGEN:H->A: Abolishes response to ADIPOQ binding; when associated with A-219; A-348 and A-352., MUTAGEN:H->A: Impairs response to ADIPOQ binding. Abolishes response to ADIPOQ binding; when associated with A-202; A-219 and A-352., REGION:Disordered, TOPO_DOM:Cytoplasmic, TOPO_DOM:Extracellular, TRANSMEM:Helical; Name=1, TRANSMEM:Helical; Name=2, TRANSMEM:Helical; Name=3, TRANSMEM:Helical; Name=4, TRANSMEM:Helical; Name=5, TRANSMEM:Helical; Name=6, TRANSMEM:Helical; Name=7, | | |
| **MGAT5** | [**alpha-1,6-mannosylglycoprotein 6-beta-N-acetylglucosaminyltransferase(MGAT5)**](https://david.ncifcrf.gov/geneReportFull.jsp?rowids=4249) | [**Related Genes**](https://david.ncifcrf.gov/relatedGenes.jsp?id=4249) | [**Homo sapiens**](http://www.ncbi.nlm.nih.gov/Taxonomy/Browser/wwwtax.cgi?name=Homo%20sapiens) |
| **GOTERM_BP_DIRECT** | [protein N-linked glycosylation](http://www.ebi.ac.uk/QuickGO/GTerm?id=GO:0006487), [protein N-linked glycosylation via asparagine](http://www.ebi.ac.uk/QuickGO/GTerm?id=GO:0018279), [viral protein processing](http://www.ebi.ac.uk/QuickGO/GTerm?id=GO:0019082), [positive regulation of cell migration](http://www.ebi.ac.uk/QuickGO/GTerm?id=GO:0030335), [negative regulation of protein tyrosine phosphatase activity](http://www.ebi.ac.uk/QuickGO/GTerm?id=GO:1903614), [positive regulation of STAT cascade](http://www.ebi.ac.uk/QuickGO/GTerm?id=GO:1904894), | | |
| **GOTERM_CC_DIRECT** | [Golgi membrane](http://www.ebi.ac.uk/QuickGO/GTerm?id=GO:0000139), [extracellular region](http://www.ebi.ac.uk/QuickGO/GTerm?id=GO:0005576), [Golgi apparatus](http://www.ebi.ac.uk/QuickGO/GTerm?id=GO:0005794), [membrane](http://www.ebi.ac.uk/QuickGO/GTerm?id=GO:0016020), [integral component of membrane](http://www.ebi.ac.uk/QuickGO/GTerm?id=GO:0016021), [extracellular exosome](http://www.ebi.ac.uk/QuickGO/GTerm?id=GO:0070062), | | |
| **GOTERM_MF_DIRECT** | [protein phosphatase inhibitor activity](http://www.ebi.ac.uk/QuickGO/GTerm?id=GO:0004864), [alpha-1,6-mannosylglycoprotein 6-beta-N-acetylglucosaminyltransferase activity](http://www.ebi.ac.uk/QuickGO/GTerm?id=GO:0030144), [manganese ion binding](http://www.ebi.ac.uk/QuickGO/GTerm?id=GO:0030145), | | |
| **INTERPRO** | [Glycosyltransferase family 18](https://www.ebi.ac.uk/interpro/entry/InterPro/IPR026116), | | |
| **KEGG_PATHWAY** | [N-Glycan biosynthesis](https://david.ncifcrf.gov/kegg.jsp?path=hsa00510$N-Glycan%20biosynthesis&termId=520047864&source=kegg), [Metabolic pathways](https://david.ncifcrf.gov/kegg.jsp?path=hsa01100$Metabolic%20pathways&termId=520047911&source=kegg), | | |
| **UP_KW_CELLULAR_COMPONENT** | [Golgi apparatus](http://www.uniprot.org/keywords/?query=KW-0333), [Membrane](http://www.uniprot.org/keywords/?query=KW-0472), [Secreted](http://www.uniprot.org/keywords/?query=KW-0964), | | |
| **UP_KW_DOMAIN** | [Coiled coil](http://www.uniprot.org/keywords/?query=KW-0175), [Signal-anchor](http://www.uniprot.org/keywords/?query=KW-0735), [Transmembrane](http://www.uniprot.org/keywords/?query=KW-0812), [Transmembrane helix](http://www.uniprot.org/keywords/?query=KW-1133), | | |
| **UP_KW_MOLECULAR_FUNCTION** | [Glycosyltransferase](http://www.uniprot.org/keywords/?query=KW-0328), [Transferase](http://www.uniprot.org/keywords/?query=KW-0808), | | |
| **UP_KW_PTM** | [Glycoprotein](http://www.uniprot.org/keywords/?query=KW-0325), [Disulfide bond](http://www.uniprot.org/keywords/?query=KW-1015), | | |
| **UP_SEQ_FEATURE** | BINDING:Substrate, BINDING:UDP-GlcNAc, CARBOHYD:N-linked (GlcNAc...) asparagine, MUTAGEN:E->A: Decreased catalytic activity., MUTAGEN:E->A: Loss of catalytic activity., MUTAGEN:H->A: No effect on the biosynthesis of the secreted form., MUTAGEN:L->D: No effect on the biosynthesis of the secreted form., REGION:Important for activity in FGF2 release, REGION:Substrate binding, REGION:Sufficient for catalytic activity, TOPO_DOM:Cytoplasmic, TOPO_DOM:Lumenal, TRANSMEM:Helical, TRANSMEM:Helical; Signal-anchor for type II membrane protein, | | |
| **APBB1** | [**amyloid beta precursor protein binding family B member 1(APBB1)**](https://david.ncifcrf.gov/geneReportFull.jsp?rowids=322) | [**Related Genes**](https://david.ncifcrf.gov/relatedGenes.jsp?id=322) | [**Homo sapiens**](http://www.ncbi.nlm.nih.gov/Taxonomy/Browser/wwwtax.cgi?name=Homo%20sapiens) |
| **GOTERM_BP_DIRECT** | [negative regulation of transcription from RNA polymerase II promoter](http://www.ebi.ac.uk/QuickGO/GTerm?id=GO:0000122), [double-strand break repair](http://www.ebi.ac.uk/QuickGO/GTerm?id=GO:0006302), [chromatin organization](http://www.ebi.ac.uk/QuickGO/GTerm?id=GO:0006325), [regulation of transcription, DNA-templated](http://www.ebi.ac.uk/QuickGO/GTerm?id=GO:0006355), [apoptotic process](http://www.ebi.ac.uk/QuickGO/GTerm?id=GO:0006915), [smooth muscle contraction](http://www.ebi.ac.uk/QuickGO/GTerm?id=GO:0006939), [cellular response to DNA damage stimulus](http://www.ebi.ac.uk/QuickGO/GTerm?id=GO:0006974), [cell cycle arrest](http://www.ebi.ac.uk/QuickGO/GTerm?id=GO:0007050), [signal transduction](http://www.ebi.ac.uk/QuickGO/GTerm?id=GO:0007165), [axonogenesis](http://www.ebi.ac.uk/QuickGO/GTerm?id=GO:0007409), [response to iron ion](http://www.ebi.ac.uk/QuickGO/GTerm?id=GO:0010039), [positive regulation of neuron projection development](http://www.ebi.ac.uk/QuickGO/GTerm?id=GO:0010976), [negative regulation of cell growth](http://www.ebi.ac.uk/QuickGO/GTerm?id=GO:0030308), [positive regulation of apoptotic process](http://www.ebi.ac.uk/QuickGO/GTerm?id=GO:0043065), [histone H4 acetylation](http://www.ebi.ac.uk/QuickGO/GTerm?id=GO:0043967), [positive regulation of DNA repair](http://www.ebi.ac.uk/QuickGO/GTerm?id=GO:0045739), [positive regulation of transcription, DNA-templated](http://www.ebi.ac.uk/QuickGO/GTerm?id=GO:0045893), [positive regulation of transcription from RNA polymerase II promoter](http://www.ebi.ac.uk/QuickGO/GTerm?id=GO:0045944), [positive regulation of protein secretion](http://www.ebi.ac.uk/QuickGO/GTerm?id=GO:0050714), [negative regulation of cell cycle G1/S phase transition](http://www.ebi.ac.uk/QuickGO/GTerm?id=GO:1902807), | | |
| **GOTERM_CC_DIRECT** | [nucleus](http://www.ebi.ac.uk/QuickGO/GTerm?id=GO:0005634), [nucleoplasm](http://www.ebi.ac.uk/QuickGO/GTerm?id=GO:0005654), [cytoplasm](http://www.ebi.ac.uk/QuickGO/GTerm?id=GO:0005737), [endoplasmic reticulum](http://www.ebi.ac.uk/QuickGO/GTerm?id=GO:0005783), [plasma membrane](http://www.ebi.ac.uk/QuickGO/GTerm?id=GO:0005886), [nuclear speck](http://www.ebi.ac.uk/QuickGO/GTerm?id=GO:0016607), [lamellipodium](http://www.ebi.ac.uk/QuickGO/GTerm?id=GO:0030027), [growth cone](http://www.ebi.ac.uk/QuickGO/GTerm?id=GO:0030426), [macromolecular complex](http://www.ebi.ac.uk/QuickGO/GTerm?id=GO:0032991), [presynaptic membrane](http://www.ebi.ac.uk/QuickGO/GTerm?id=GO:0042734), [neuronal cell body](http://www.ebi.ac.uk/QuickGO/GTerm?id=GO:0043025), [dendritic spine](http://www.ebi.ac.uk/QuickGO/GTerm?id=GO:0043197), [main axon](http://www.ebi.ac.uk/QuickGO/GTerm?id=GO:0044304), [synapse](http://www.ebi.ac.uk/QuickGO/GTerm?id=GO:0045202), [postsynaptic membrane](http://www.ebi.ac.uk/QuickGO/GTerm?id=GO:0045211), [perinuclear region of cytoplasm](http://www.ebi.ac.uk/QuickGO/GTerm?id=GO:0048471), [growth cone lamellipodium](http://www.ebi.ac.uk/QuickGO/GTerm?id=GO:1990761), [growth cone filopodium](http://www.ebi.ac.uk/QuickGO/GTerm?id=GO:1990812), | | |
| **GOTERM_MF_DIRECT** | [beta-amyloid binding](http://www.ebi.ac.uk/QuickGO/GTerm?id=GO:0001540), [chromatin binding](http://www.ebi.ac.uk/QuickGO/GTerm?id=GO:0003682), [transcription coactivator activity](http://www.ebi.ac.uk/QuickGO/GTerm?id=GO:0003713), [protein binding](http://www.ebi.ac.uk/QuickGO/GTerm?id=GO:0005515), [transcription factor binding](http://www.ebi.ac.uk/QuickGO/GTerm?id=GO:0008134), [ubiquitin protein ligase binding](http://www.ebi.ac.uk/QuickGO/GTerm?id=GO:0031625), [histone binding](http://www.ebi.ac.uk/QuickGO/GTerm?id=GO:0042393), [macromolecular complex binding](http://www.ebi.ac.uk/QuickGO/GTerm?id=GO:0044877), [tau protein binding](http://www.ebi.ac.uk/QuickGO/GTerm?id=GO:0048156), [low-density lipoprotein particle receptor binding](http://www.ebi.ac.uk/QuickGO/GTerm?id=GO:0050750), [proline-rich region binding](http://www.ebi.ac.uk/QuickGO/GTerm?id=GO:0070064), | | |
| **INTERPRO** | [WW domain](https://www.ebi.ac.uk/interpro/entry/InterPro/IPR001202), [Phosphotyrosine interaction domain](https://www.ebi.ac.uk/interpro/entry/InterPro/IPR006020), [Pleckstrin homology-like domain](https://www.ebi.ac.uk/interpro/entry/InterPro/IPR011993), | | |
| **KEGG_PATHWAY** | [Alzheimer disease](https://david.ncifcrf.gov/kegg.jsp?path=hsa05010$Alzheimer%20disease&termId=520048087&source=kegg), | | |
| **SMART** | [WW](http://smart.embl.de/smart/do_annotation.pl?DOMAIN=SM00456), [PTB](http://smart.embl.de/smart/do_annotation.pl?DOMAIN=SM00462), | | |
| **UP_KW_BIOLOGICAL_PROCESS** | [Apoptosis](http://www.uniprot.org/keywords/?query=KW-0053), [DNA damage](http://www.uniprot.org/keywords/?query=KW-0227), [Transcription](http://www.uniprot.org/keywords/?query=KW-0804), [Transcription regulation](http://www.uniprot.org/keywords/?query=KW-0805), | | |
| **UP_KW_CELLULAR_COMPONENT** | [Membrane](http://www.uniprot.org/keywords/?query=KW-0472), [Nucleus](http://www.uniprot.org/keywords/?query=KW-0539), [Cytoplasm](http://www.uniprot.org/keywords/?query=KW-0963), [Cell projection](http://www.uniprot.org/keywords/?query=KW-0966), [Cell membrane](http://www.uniprot.org/keywords/?query=KW-1003), | | |
| **UP_KW_DOMAIN** | [Repeat](http://www.uniprot.org/keywords/?query=KW-0677), | | |
| **UP_KW_MOLECULAR_FUNCTION** | [Activator](http://www.uniprot.org/keywords/?query=KW-0010), [Chromatin regulator](http://www.uniprot.org/keywords/?query=KW-0156), [Repressor](http://www.uniprot.org/keywords/?query=KW-0678), | | |
| **UP_KW_PTM** | [Phosphoprotein](http://www.uniprot.org/keywords/?query=KW-0597), [Ubl conjugation](http://www.uniprot.org/keywords/?query=KW-0832), | | |
| **UP_SEQ_FEATURE** | COMPBIAS:Acidic residues, COMPBIAS:Basic and acidic residues, COMPBIAS:Polar residues, DOMAIN:PID, DOMAIN:PID 1, DOMAIN:PID 2, DOMAIN:WW, MUTAGEN:Y->F: Abrogates phosphorylation and stimulation of transcription by ABL1, and increases the interaction with RASD1/DEXRAS1., MUTAGEN:Y->F: No effect on phosphorylation by ABL1., MUTAGEN:YYW->AAA: Impairs transcriptional activation and inhibits binding to ABL1., REGION:Disordered, | | |
| **ANAPC16** | [**anaphase promoting complex subunit 16(ANAPC16)**](https://david.ncifcrf.gov/geneReportFull.jsp?rowids=119504) | [**Related Genes**](https://david.ncifcrf.gov/relatedGenes.jsp?id=119504) | [**Homo sapiens**](http://www.ncbi.nlm.nih.gov/Taxonomy/Browser/wwwtax.cgi?name=Homo%20sapiens) |
| **GOTERM_BP_DIRECT** | [ubiquitin-dependent protein catabolic process](http://www.ebi.ac.uk/QuickGO/GTerm?id=GO:0006511), [cell cycle](http://www.ebi.ac.uk/QuickGO/GTerm?id=GO:0007049), [regulation of exit from mitosis](http://www.ebi.ac.uk/QuickGO/GTerm?id=GO:0007096), [protein ubiquitination](http://www.ebi.ac.uk/QuickGO/GTerm?id=GO:0016567), [anaphase-promoting complex-dependent catabolic process](http://www.ebi.ac.uk/QuickGO/GTerm?id=GO:0031145), [cell division](http://www.ebi.ac.uk/QuickGO/GTerm?id=GO:0051301), [regulation of mitotic cell cycle phase transition](http://www.ebi.ac.uk/QuickGO/GTerm?id=GO:1901990), | | |
| **GOTERM_CC_DIRECT** | [kinetochore](http://www.ebi.ac.uk/QuickGO/GTerm?id=GO:0000776), [condensed chromosome kinetochore](http://www.ebi.ac.uk/QuickGO/GTerm?id=GO:0000777), [nucleoplasm](http://www.ebi.ac.uk/QuickGO/GTerm?id=GO:0005654), [anaphase-promoting complex](http://www.ebi.ac.uk/QuickGO/GTerm?id=GO:0005680), [cytoplasm](http://www.ebi.ac.uk/QuickGO/GTerm?id=GO:0005737), [cytosol](http://www.ebi.ac.uk/QuickGO/GTerm?id=GO:0005829), | | |
| **GOTERM_MF_DIRECT** | [protein binding](http://www.ebi.ac.uk/QuickGO/GTerm?id=GO:0005515), | | |
| **KEGG_PATHWAY** | [Cell cycle](https://david.ncifcrf.gov/kegg.jsp?path=hsa04110$Cell%20cycle&termId=520047958&source=kegg), [Oocyte meiosis](https://david.ncifcrf.gov/kegg.jsp?path=hsa04114$Oocyte%20meiosis&termId=520047959&source=kegg), [Ubiquitin mediated proteolysis](https://david.ncifcrf.gov/kegg.jsp?path=hsa04120$Ubiquitin%20mediated%20proteolysis&termId=520047961&source=kegg), [Progesterone-mediated oocyte maturation](https://david.ncifcrf.gov/kegg.jsp?path=hsa04914$Progesterone-mediated%20oocyte%20maturation&termId=520048047&source=kegg), [Human T-cell leukemia virus 1 infection](https://david.ncifcrf.gov/kegg.jsp?path=hsa05166$Human%20T-cell%20leukemia%20virus%201%20infection&termId=520048122&source=kegg), | | |
| **UP_KW_BIOLOGICAL_PROCESS** | [Cell cycle](http://www.uniprot.org/keywords/?query=KW-0131), [Cell division](http://www.uniprot.org/keywords/?query=KW-0132), [Mitosis](http://www.uniprot.org/keywords/?query=KW-0498), [Ubl conjugation pathway](http://www.uniprot.org/keywords/?query=KW-0833), | | |
| **UP_KW_CELLULAR_COMPONENT** | [Centromere](http://www.uniprot.org/keywords/?query=KW-0137), [Chromosome](http://www.uniprot.org/keywords/?query=KW-0158), [Nucleus](http://www.uniprot.org/keywords/?query=KW-0539), [Cytoplasm](http://www.uniprot.org/keywords/?query=KW-0963), [Kinetochore](http://www.uniprot.org/keywords/?query=KW-0995), | | |
| **UP_KW_DOMAIN** | [Coiled coil](http://www.uniprot.org/keywords/?query=KW-0175), | | |
| **UP_KW_PTM** | [Acetylation](http://www.uniprot.org/keywords/?query=KW-0007), | | |
| **UP_SEQ_FEATURE** | COMPBIAS:Polar residues, REGION:Disordered, | | |
| **ANKMY2** | [**ankyrin repeat and MYND domain containing 2(ANKMY2)**](https://david.ncifcrf.gov/geneReportFull.jsp?rowids=57037) | [**Related Genes**](https://david.ncifcrf.gov/relatedGenes.jsp?id=57037) | [**Homo sapiens**](http://www.ncbi.nlm.nih.gov/Taxonomy/Browser/wwwtax.cgi?name=Homo%20sapiens) |
| **GOTERM_CC_DIRECT** | [cilium](http://www.ebi.ac.uk/QuickGO/GTerm?id=GO:0005929), | | |
| **GOTERM_MF_DIRECT** | [protein binding](http://www.ebi.ac.uk/QuickGO/GTerm?id=GO:0005515), [enzyme binding](http://www.ebi.ac.uk/QuickGO/GTerm?id=GO:0019899), [metal ion binding](http://www.ebi.ac.uk/QuickGO/GTerm?id=GO:0046872), | | |
| **INTERPRO** | [Ankyrin repeat](https://www.ebi.ac.uk/interpro/entry/InterPro/IPR002110), [Zinc finger, MYND-type](https://www.ebi.ac.uk/interpro/entry/InterPro/IPR002893), [Ankyrin repeat-containing domain](https://www.ebi.ac.uk/interpro/entry/InterPro/IPR020683), | | |
| **SMART** | [ANK](http://smart.embl.de/smart/do_annotation.pl?DOMAIN=SM00248), | | |
| **UP_KW_CELLULAR_COMPONENT** | [Cell projection](http://www.uniprot.org/keywords/?query=KW-0966), [Cilium](http://www.uniprot.org/keywords/?query=KW-0969), | | |
| **UP_KW_DOMAIN** | [ANK repeat](http://www.uniprot.org/keywords/?query=KW-0040), [Repeat](http://www.uniprot.org/keywords/?query=KW-0677), [Zinc-finger](http://www.uniprot.org/keywords/?query=KW-0863), | | |
| **UP_KW_LIGAND** | [Metal-binding](http://www.uniprot.org/keywords/?query=KW-0479), [Zinc](http://www.uniprot.org/keywords/?query=KW-0862), | | |
| **UP_SEQ_FEATURE** | COMPBIAS:Basic and acidic residues, COMPBIAS:Polar residues, DOMAIN:Ankyrin_rpt-contain_dom, DOMAIN:MYND-type, METAL:Zinc 1, METAL:Zinc 2, REGION:Disordered, REPEAT:ANK, REPEAT:ANK 1, REPEAT:ANK 2, REPEAT:ANK 3, ZN_FING:MYND-type, | | |
| **ANKS3** | [**ankyrin repeat and sterile alpha motif domain containing 3(ANKS3)**](https://david.ncifcrf.gov/geneReportFull.jsp?rowids=124401) | [**Related Genes**](https://david.ncifcrf.gov/relatedGenes.jsp?id=124401) | [**Homo sapiens**](http://www.ncbi.nlm.nih.gov/Taxonomy/Browser/wwwtax.cgi?name=Homo%20sapiens) |
| **GOTERM_CC_DIRECT** | [cytoplasm](http://www.ebi.ac.uk/QuickGO/GTerm?id=GO:0005737), [cilium](http://www.ebi.ac.uk/QuickGO/GTerm?id=GO:0005929), | | |
| **GOTERM_MF_DIRECT** | [protein binding](http://www.ebi.ac.uk/QuickGO/GTerm?id=GO:0005515), | | |
| **INTERPRO** | [Sterile alpha motif domain](https://www.ebi.ac.uk/interpro/entry/InterPro/IPR001660), [Ankyrin repeat](https://www.ebi.ac.uk/interpro/entry/InterPro/IPR002110), [Sterile alpha motif/pointed domain](https://www.ebi.ac.uk/interpro/entry/InterPro/IPR013761), [Ankyrin repeat-containing domain](https://www.ebi.ac.uk/interpro/entry/InterPro/IPR020683), | | |
| **SMART** | [ANK](http://smart.embl.de/smart/do_annotation.pl?DOMAIN=SM00248), [SAM](http://smart.embl.de/smart/do_annotation.pl?DOMAIN=SM00454), | | |
| **UP_KW_CELLULAR_COMPONENT** | [Cytoplasm](http://www.uniprot.org/keywords/?query=KW-0963), [Cell projection](http://www.uniprot.org/keywords/?query=KW-0966), | | |
| **UP_KW_DOMAIN** | [ANK repeat](http://www.uniprot.org/keywords/?query=KW-0040), [Coiled coil](http://www.uniprot.org/keywords/?query=KW-0175), [Repeat](http://www.uniprot.org/keywords/?query=KW-0677), | | |
| **UP_KW_PTM** | [Hydroxylation](http://www.uniprot.org/keywords/?query=KW-0379), [Phosphoprotein](http://www.uniprot.org/keywords/?query=KW-0597), [Ubl conjugation](http://www.uniprot.org/keywords/?query=KW-0832), | | |
| **UP_SEQ_FEATURE** | COMPBIAS:Polar residues, DOMAIN:Ankyrin_rpt-contain_dom, DOMAIN:SAM, MUTAGEN:D->K: Decreased homooligomerization. No effect on interaction with ANKS6., MUTAGEN:E->K: Decreased homooligomerization. No effect on interaction with ANKS6., MUTAGEN:F->E: Decreased homooligomerization. Decreased interaction with ANKS6., MUTAGEN:I->E: Decreased homooligomerization. No effect on interaction with ANKS6., MUTAGEN:K->E: Decreased homooligomerization. Decreased interaction with ANKS6., MUTAGEN:L->E: Decreased homooligomerization. Decreased interaction with ANKS6., REGION:Disordered, REGION:Interaction with NEK7, REPEAT:ANK, REPEAT:ANK 1, REPEAT:ANK 2, REPEAT:ANK 3, REPEAT:ANK 4, REPEAT:ANK 5, REPEAT:ANK 6, | | |
| **ANKRD44** | [**ankyrin repeat domain 44(ANKRD44)**](https://david.ncifcrf.gov/geneReportFull.jsp?rowids=91526) | [**Related Genes**](https://david.ncifcrf.gov/relatedGenes.jsp?id=91526) | [**Homo sapiens**](http://www.ncbi.nlm.nih.gov/Taxonomy/Browser/wwwtax.cgi?name=Homo%20sapiens) |
| **GOTERM_MF_DIRECT** | [protein binding](http://www.ebi.ac.uk/QuickGO/GTerm?id=GO:0005515), | | |
| **INTERPRO** | [Ankyrin repeat](https://www.ebi.ac.uk/interpro/entry/InterPro/IPR002110), [Ankyrin repeat-containing domain](https://www.ebi.ac.uk/interpro/entry/InterPro/IPR020683), | | |
| **SMART** | [ANK](http://smart.embl.de/smart/do_annotation.pl?DOMAIN=SM00248), | | |
| **UP_KW_DOMAIN** | [ANK repeat](http://www.uniprot.org/keywords/?query=KW-0040), [Coiled coil](http://www.uniprot.org/keywords/?query=KW-0175), [Repeat](http://www.uniprot.org/keywords/?query=KW-0677), | | |
| **UP_SEQ_FEATURE** | DOMAIN:Ankyrin_rpt-contain_dom, REPEAT:ANK, REPEAT:ANK 1, REPEAT:ANK 10, REPEAT:ANK 11, REPEAT:ANK 12, REPEAT:ANK 13, REPEAT:ANK 14, REPEAT:ANK 15, REPEAT:ANK 16, REPEAT:ANK 17, REPEAT:ANK 18, REPEAT:ANK 19, REPEAT:ANK 2, REPEAT:ANK 20, REPEAT:ANK 21, REPEAT:ANK 22, REPEAT:ANK 23, REPEAT:ANK 24, REPEAT:ANK 25, REPEAT:ANK 26, REPEAT:ANK 27, REPEAT:ANK 28, REPEAT:ANK 3, REPEAT:ANK 4, REPEAT:ANK 5, REPEAT:ANK 6, REPEAT:ANK 7, REPEAT:ANK 8, REPEAT:ANK 9, | | |
| **ANO3** | [**anoctamin 3(ANO3)**](https://david.ncifcrf.gov/geneReportFull.jsp?rowids=63982) | [**Related Genes**](https://david.ncifcrf.gov/relatedGenes.jsp?id=63982) | [**Homo sapiens**](http://www.ncbi.nlm.nih.gov/Taxonomy/Browser/wwwtax.cgi?name=Homo%20sapiens) |
| **GOTERM_BP_DIRECT** | [detection of temperature stimulus](http://www.ebi.ac.uk/QuickGO/GTerm?id=GO:0016048), [ion transmembrane transport](http://www.ebi.ac.uk/QuickGO/GTerm?id=GO:0034220), [detection of mechanical stimulus](http://www.ebi.ac.uk/QuickGO/GTerm?id=GO:0050982), [transmembrane transport](http://www.ebi.ac.uk/QuickGO/GTerm?id=GO:0055085), [calcium activated phospholipid scrambling](http://www.ebi.ac.uk/QuickGO/GTerm?id=GO:0061588), [calcium activated phosphatidylcholine scrambling](http://www.ebi.ac.uk/QuickGO/GTerm?id=GO:0061590), [calcium activated galactosylceramide scrambling](http://www.ebi.ac.uk/QuickGO/GTerm?id=GO:0061591), [chloride transmembrane transport](http://www.ebi.ac.uk/QuickGO/GTerm?id=GO:1902476), | | |
| **GOTERM_CC_DIRECT** | [plasma membrane](http://www.ebi.ac.uk/QuickGO/GTerm?id=GO:0005886), [integral component of membrane](http://www.ebi.ac.uk/QuickGO/GTerm?id=GO:0016021), | | |
| **GOTERM_MF_DIRECT** | [intracellular calcium activated chloride channel activity](http://www.ebi.ac.uk/QuickGO/GTerm?id=GO:0005229), [chloride channel activity](http://www.ebi.ac.uk/QuickGO/GTerm?id=GO:0005254), [phospholipid scramblase activity](http://www.ebi.ac.uk/QuickGO/GTerm?id=GO:0017128), [protein dimerization activity](http://www.ebi.ac.uk/QuickGO/GTerm?id=GO:0046983), | | |
| **INTERPRO** | [Anoctamin/TMEM 16](https://www.ebi.ac.uk/interpro/entry/InterPro/IPR007632), | | |
| **OMIM_DISEASE** | [Dystonia 24](http://omim.org/entry/615034), | | |
| **UP_KW_BIOLOGICAL_PROCESS** | [Lipid transport](http://www.uniprot.org/keywords/?query=KW-0445), [Transport](http://www.uniprot.org/keywords/?query=KW-0813), | | |
| **UP_KW_CELLULAR_COMPONENT** | [Membrane](http://www.uniprot.org/keywords/?query=KW-0472), [Cell membrane](http://www.uniprot.org/keywords/?query=KW-1003), | | |
| **UP_KW_DISEASE** | [Disease variant](http://www.uniprot.org/keywords/?query=KW-0225), [Dystonia](http://www.uniprot.org/keywords/?query=KW-1023), | | |
| **UP_KW_DOMAIN** | [Coiled coil](http://www.uniprot.org/keywords/?query=KW-0175), [Transmembrane](http://www.uniprot.org/keywords/?query=KW-0812), [Transmembrane helix](http://www.uniprot.org/keywords/?query=KW-1133), | | |
| **UP_KW_PTM** | [Glycoprotein](http://www.uniprot.org/keywords/?query=KW-0325), | | |
| **UP_SEQ_FEATURE** | CARBOHYD:N-linked (GlcNAc...) asparagine, DOMAIN:Anoct_dimer, REGION:Disordered, TOPO_DOM:Cytoplasmic, TOPO_DOM:Extracellular, TRANSMEM:Helical, | | |
| **APOBEC3H** | [**apolipoprotein B mRNA editing enzyme catalytic subunit 3H(APOBEC3H)**](https://david.ncifcrf.gov/geneReportFull.jsp?rowids=164668) | [**Related Genes**](https://david.ncifcrf.gov/relatedGenes.jsp?id=164668) | [**Homo sapiens**](http://www.ncbi.nlm.nih.gov/Taxonomy/Browser/wwwtax.cgi?name=Homo%20sapiens) |
| **GOTERM_BP_DIRECT** | [cytidine deamination](http://www.ebi.ac.uk/QuickGO/GTerm?id=GO:0009972), [negative regulation of transposition](http://www.ebi.ac.uk/QuickGO/GTerm?id=GO:0010529), [cytidine to uridine editing](http://www.ebi.ac.uk/QuickGO/GTerm?id=GO:0016554), [innate immune response](http://www.ebi.ac.uk/QuickGO/GTerm?id=GO:0045087), [negative regulation of single stranded viral RNA replication via double stranded DNA intermediate](http://www.ebi.ac.uk/QuickGO/GTerm?id=GO:0045869), [negative regulation of viral process](http://www.ebi.ac.uk/QuickGO/GTerm?id=GO:0048525), [defense response to virus](http://www.ebi.ac.uk/QuickGO/GTerm?id=GO:0051607), [DNA cytosine deamination](http://www.ebi.ac.uk/QuickGO/GTerm?id=GO:0070383), [DNA demethylation](http://www.ebi.ac.uk/QuickGO/GTerm?id=GO:0080111), | | |
| **GOTERM_CC_DIRECT** | [P-body](http://www.ebi.ac.uk/QuickGO/GTerm?id=GO:0000932), [nucleus](http://www.ebi.ac.uk/QuickGO/GTerm?id=GO:0005634), [nucleoplasm](http://www.ebi.ac.uk/QuickGO/GTerm?id=GO:0005654), [cytoplasm](http://www.ebi.ac.uk/QuickGO/GTerm?id=GO:0005737), [cytosol](http://www.ebi.ac.uk/QuickGO/GTerm?id=GO:0005829), | | |
| **GOTERM_MF_DIRECT** | [RNA binding](http://www.ebi.ac.uk/QuickGO/GTerm?id=GO:0003723), [cytidine deaminase activity](http://www.ebi.ac.uk/QuickGO/GTerm?id=GO:0004126), [protein binding](http://www.ebi.ac.uk/QuickGO/GTerm?id=GO:0005515), [zinc ion binding](http://www.ebi.ac.uk/QuickGO/GTerm?id=GO:0008270), [hydrolase activity](http://www.ebi.ac.uk/QuickGO/GTerm?id=GO:0016787), [deoxycytidine deaminase activity](http://www.ebi.ac.uk/QuickGO/GTerm?id=GO:0047844), | | |
| **INTERPRO** | [CMP/dCMP deaminase, zinc-binding](https://www.ebi.ac.uk/interpro/entry/InterPro/IPR002125), [APOBEC/CMP deaminase, zinc-binding](https://www.ebi.ac.uk/interpro/entry/InterPro/IPR016192), [Cytidine deaminase-like](https://www.ebi.ac.uk/interpro/entry/InterPro/IPR016193), | | |
| **KEGG_PATHWAY** | [Human immunodeficiency virus 1 infection](https://david.ncifcrf.gov/kegg.jsp?path=hsa05170$Human%20immunodeficiency%20virus%201%20infection&termId=520048126&source=kegg), | | |
| **UP_KW_BIOLOGICAL_PROCESS** | [Antiviral defense](http://www.uniprot.org/keywords/?query=KW-0051), [Immunity](http://www.uniprot.org/keywords/?query=KW-0391), [Innate immunity](http://www.uniprot.org/keywords/?query=KW-0399), | | |
| **UP_KW_CELLULAR_COMPONENT** | [Nucleus](http://www.uniprot.org/keywords/?query=KW-0539), [Cytoplasm](http://www.uniprot.org/keywords/?query=KW-0963), | | |
| **UP_KW_DOMAIN** | [Coiled coil](http://www.uniprot.org/keywords/?query=KW-0175), | | |
| **UP_KW_LIGAND** | [Metal-binding](http://www.uniprot.org/keywords/?query=KW-0479), [Zinc](http://www.uniprot.org/keywords/?query=KW-0862), | | |
| **UP_KW_MOLECULAR_FUNCTION** | [Hydrolase](http://www.uniprot.org/keywords/?query=KW-0378), | | |
| **UP_KW_PTM** | [Lipoprotein](http://www.uniprot.org/keywords/?query=KW-0449), | | |
| **UP_SEQ_FEATURE** | ACT_SITE:Proton donor, DOMAIN:CMP/dCMP-type deaminase, METAL:Zinc, MUTAGEN:E->Q: Reduces the ability to inhibit the retrotransposition of LINE-1 elements., | | |
| **RSRC2** | [**arginine and serine rich coiled-coil 2(RSRC2)**](https://david.ncifcrf.gov/geneReportFull.jsp?rowids=65117) | [**Related Genes**](https://david.ncifcrf.gov/relatedGenes.jsp?id=65117) | [**Homo sapiens**](http://www.ncbi.nlm.nih.gov/Taxonomy/Browser/wwwtax.cgi?name=Homo%20sapiens) |
| **GOTERM_MF_DIRECT** | [RNA binding](http://www.ebi.ac.uk/QuickGO/GTerm?id=GO:0003723), [protein binding](http://www.ebi.ac.uk/QuickGO/GTerm?id=GO:0005515), | | |
| **UP_KW_DOMAIN** | [Coiled coil](http://www.uniprot.org/keywords/?query=KW-0175), | | |
| **UP_KW_PTM** | [Acetylation](http://www.uniprot.org/keywords/?query=KW-0007), [Phosphoprotein](http://www.uniprot.org/keywords/?query=KW-0597), [Ubl conjugation](http://www.uniprot.org/keywords/?query=KW-0832), [Isopeptide bond](http://www.uniprot.org/keywords/?query=KW-1017), | | |
| **UP_SEQ_FEATURE** | COMPBIAS:Basic and acidic residues, COMPBIAS:Basic residues, CROSSLNK:Glycyl lysine isopeptide (Lys-Gly) (interchain with G-Cter in SUMO1); alternate, CROSSLNK:Glycyl lysine isopeptide (Lys-Gly) (interchain with G-Cter in SUMO2); alternate, DOMAIN:SMAP, REGION:Disordered, | | |
| **AGO2** | [**argonaute RISC catalytic component 2(AGO2)**](https://david.ncifcrf.gov/geneReportFull.jsp?rowids=27161) | [**Related Genes**](https://david.ncifcrf.gov/relatedGenes.jsp?id=27161) | [**Homo sapiens**](http://www.ncbi.nlm.nih.gov/Taxonomy/Browser/wwwtax.cgi?name=Homo%20sapiens) |
| **BIOCARTA** | [Dicer Pathway](https://david.ncifcrf.gov/biocarta.jsp?path=h_dicerPathway$Dicer%20Pathway&termId=30000084&source=biocarta), | | |
| **GOTERM_BP_DIRECT** | [translation](http://www.ebi.ac.uk/QuickGO/GTerm?id=GO:0006412), [translational initiation](http://www.ebi.ac.uk/QuickGO/GTerm?id=GO:0006413), [Wnt signaling pathway, calcium modulating pathway](http://www.ebi.ac.uk/QuickGO/GTerm?id=GO:0007223), [post-embryonic development](http://www.ebi.ac.uk/QuickGO/GTerm?id=GO:0009791), [RNA secondary structure unwinding](http://www.ebi.ac.uk/QuickGO/GTerm?id=GO:0010501), [miRNA metabolic process](http://www.ebi.ac.uk/QuickGO/GTerm?id=GO:0010586), [positive regulation of gene expression](http://www.ebi.ac.uk/QuickGO/GTerm?id=GO:0010628), [negative regulation of gene expression](http://www.ebi.ac.uk/QuickGO/GTerm?id=GO:0010629), [RNA interference](http://www.ebi.ac.uk/QuickGO/GTerm?id=GO:0016246), [production of siRNA involved in RNA interference](http://www.ebi.ac.uk/QuickGO/GTerm?id=GO:0030422), [intracellular receptor signaling pathway](http://www.ebi.ac.uk/QuickGO/GTerm?id=GO:0030522), [gene silencing by RNA](http://www.ebi.ac.uk/QuickGO/GTerm?id=GO:0031047), [pre-miRNA processing](http://www.ebi.ac.uk/QuickGO/GTerm?id=GO:0031054), [siRNA loading onto RISC involved in RNA interference](http://www.ebi.ac.uk/QuickGO/GTerm?id=GO:0035087), [posttranscriptional gene silencing by RNA](http://www.ebi.ac.uk/QuickGO/GTerm?id=GO:0035194), [production of miRNAs involved in gene silencing by miRNA](http://www.ebi.ac.uk/QuickGO/GTerm?id=GO:0035196), [miRNA mediated inhibition of translation](http://www.ebi.ac.uk/QuickGO/GTerm?id=GO:0035278), [mRNA cleavage involved in gene silencing by miRNA](http://www.ebi.ac.uk/QuickGO/GTerm?id=GO:0035279), [miRNA loading onto RISC involved in gene silencing by miRNA](http://www.ebi.ac.uk/QuickGO/GTerm?id=GO:0035280), [negative regulation of amyloid precursor protein biosynthetic process](http://www.ebi.ac.uk/QuickGO/GTerm?id=GO:0042985), [positive regulation of angiogenesis](http://www.ebi.ac.uk/QuickGO/GTerm?id=GO:0045766), [positive regulation of transcription from RNA polymerase II promoter](http://www.ebi.ac.uk/QuickGO/GTerm?id=GO:0045944), [negative regulation of translational initiation](http://www.ebi.ac.uk/QuickGO/GTerm?id=GO:0045947), [positive regulation of translation, ncRNA-mediated](http://www.ebi.ac.uk/QuickGO/GTerm?id=GO:0045975), [positive regulation of nuclear-transcribed mRNA poly(A) tail shortening](http://www.ebi.ac.uk/QuickGO/GTerm?id=GO:0060213), [regulation of gene silencing by miRNA](http://www.ebi.ac.uk/QuickGO/GTerm?id=GO:0060964), [RNA phosphodiester bond hydrolysis, endonucleolytic](http://www.ebi.ac.uk/QuickGO/GTerm?id=GO:0090502), [mRNA cleavage involved in gene silencing by siRNA](http://www.ebi.ac.uk/QuickGO/GTerm?id=GO:0090625), [positive regulation of nuclear-transcribed mRNA catabolic process, deadenylation-dependent decay](http://www.ebi.ac.uk/QuickGO/GTerm?id=GO:1900153), [positive regulation of trophoblast cell migration](http://www.ebi.ac.uk/QuickGO/GTerm?id=GO:1901165), [positive regulation of miRNA mediated inhibition of translation](http://www.ebi.ac.uk/QuickGO/GTerm?id=GO:1905618), | | |
| **GOTERM_CC_DIRECT** | [P-body](http://www.ebi.ac.uk/QuickGO/GTerm?id=GO:0000932), [nucleus](http://www.ebi.ac.uk/QuickGO/GTerm?id=GO:0005634), [nucleoplasm](http://www.ebi.ac.uk/QuickGO/GTerm?id=GO:0005654), [cytoplasm](http://www.ebi.ac.uk/QuickGO/GTerm?id=GO:0005737), [cytosol](http://www.ebi.ac.uk/QuickGO/GTerm?id=GO:0005829), [polysome](http://www.ebi.ac.uk/QuickGO/GTerm?id=GO:0005844), [mRNA cap binding complex](http://www.ebi.ac.uk/QuickGO/GTerm?id=GO:0005845), [membrane](http://www.ebi.ac.uk/QuickGO/GTerm?id=GO:0016020), [RISC complex](http://www.ebi.ac.uk/QuickGO/GTerm?id=GO:0016442), [dendrite](http://www.ebi.ac.uk/QuickGO/GTerm?id=GO:0030425), [cytoplasmic ribonucleoprotein granule](http://www.ebi.ac.uk/QuickGO/GTerm?id=GO:0036464), [extracellular exosome](http://www.ebi.ac.uk/QuickGO/GTerm?id=GO:0070062), [RISC-loading complex](http://www.ebi.ac.uk/QuickGO/GTerm?id=GO:0070578), [ribonucleoprotein complex](http://www.ebi.ac.uk/QuickGO/GTerm?id=GO:1990904), | | |
| **GOTERM_MF_DIRECT** | [RNA 7-methylguanosine cap binding](http://www.ebi.ac.uk/QuickGO/GTerm?id=GO:0000340), [RNA polymerase II core binding](http://www.ebi.ac.uk/QuickGO/GTerm?id=GO:0000993), [core promoter sequence-specific DNA binding](http://www.ebi.ac.uk/QuickGO/GTerm?id=GO:0001046), [RNA binding](http://www.ebi.ac.uk/QuickGO/GTerm?id=GO:0003723), [double-stranded RNA binding](http://www.ebi.ac.uk/QuickGO/GTerm?id=GO:0003725), [single-stranded RNA binding](http://www.ebi.ac.uk/QuickGO/GTerm?id=GO:0003727), [translation initiation factor activity](http://www.ebi.ac.uk/QuickGO/GTerm?id=GO:0003743), [endoribonuclease activity](http://www.ebi.ac.uk/QuickGO/GTerm?id=GO:0004521), [protein binding](http://www.ebi.ac.uk/QuickGO/GTerm?id=GO:0005515), [protein C-terminus binding](http://www.ebi.ac.uk/QuickGO/GTerm?id=GO:0008022), [siRNA binding](http://www.ebi.ac.uk/QuickGO/GTerm?id=GO:0035197), [miRNA binding](http://www.ebi.ac.uk/QuickGO/GTerm?id=GO:0035198), [metal ion binding](http://www.ebi.ac.uk/QuickGO/GTerm?id=GO:0046872), [endoribonuclease activity, cleaving siRNA-paired mRNA](http://www.ebi.ac.uk/QuickGO/GTerm?id=GO:0070551), [endoribonuclease activity, cleaving miRNA-paired mRNA](http://www.ebi.ac.uk/QuickGO/GTerm?id=GO:0090624), [mRNA cap binding](http://www.ebi.ac.uk/QuickGO/GTerm?id=GO:0098808), | | |
| **INTERPRO** | [Argonaute/Dicer protein, PAZ](https://www.ebi.ac.uk/interpro/entry/InterPro/IPR003100), [Stem cell self-renewal protein Piwi](https://www.ebi.ac.uk/interpro/entry/InterPro/IPR003165), [Ribonuclease H-like domain](https://www.ebi.ac.uk/interpro/entry/InterPro/IPR012337), [Domain of unknown function DUF1785](https://www.ebi.ac.uk/interpro/entry/InterPro/IPR014811), | | |
| **OMIM_DISEASE** | [Lessel-Kreienkamp syndrome](http://omim.org/entry/619149), | | |
| **SMART** | [SM00949](http://smart.embl.de/smart/do_annotation.pl?DOMAIN=SM00949), [SM00950](http://smart.embl.de/smart/do_annotation.pl?DOMAIN=SM00950), [SM01163](http://smart.embl.de/smart/do_annotation.pl?DOMAIN=SM01163), | | |
| **UP_KW_BIOLOGICAL_PROCESS** | [Transcription](http://www.uniprot.org/keywords/?query=KW-0804), [Transcription regulation](http://www.uniprot.org/keywords/?query=KW-0805), [Translation regulation](http://www.uniprot.org/keywords/?query=KW-0810), [RNA-mediated gene silencing](http://www.uniprot.org/keywords/?query=KW-0943), | | |
| **UP_KW_CELLULAR_COMPONENT** | [Nucleus](http://www.uniprot.org/keywords/?query=KW-0539), [Cytoplasm](http://www.uniprot.org/keywords/?query=KW-0963), | | |
| **UP_KW_LIGAND** | [Magnesium](http://www.uniprot.org/keywords/?query=KW-0460), [Manganese](http://www.uniprot.org/keywords/?query=KW-0464), [Metal-binding](http://www.uniprot.org/keywords/?query=KW-0479), | | |
| **UP_KW_MOLECULAR_FUNCTION** | [Endonuclease](http://www.uniprot.org/keywords/?query=KW-0255), [Hydrolase](http://www.uniprot.org/keywords/?query=KW-0378), [Nuclease](http://www.uniprot.org/keywords/?query=KW-0540), [Repressor](http://www.uniprot.org/keywords/?query=KW-0678), [Ribonucleoprotein](http://www.uniprot.org/keywords/?query=KW-0687), [RNA-binding](http://www.uniprot.org/keywords/?query=KW-0694), | | |
| **UP_KW_PTM** | [Hydroxylation](http://www.uniprot.org/keywords/?query=KW-0379), [Phosphoprotein](http://www.uniprot.org/keywords/?query=KW-0597), [Ubl conjugation](http://www.uniprot.org/keywords/?query=KW-0832), [Nitration](http://www.uniprot.org/keywords/?query=KW-0944), | | |
| **UP_SEQ_FEATURE** | COMPBIAS:Pro residues, DOMAIN:PAZ, DOMAIN:Piwi, METAL:Divalent metal cation, MUTAGEN:D->A: Abrogates RNA cleavage but does not affect binding to siRNA or translational repression., MUTAGEN:D->A: Abrogates RNA cleavage but does not affect binding to siRNA., MUTAGEN:E->A: Impairs RNA cleavage., MUTAGEN:E->G: No effect on RNA cleavage., MUTAGEN:F->A,I,M,R,Y: Impairs RNA cleavage., MUTAGEN:F->V: Abrogates RNA cleavage., MUTAGEN:F->V: No effect on miRNA-binding or target mRNA cleavage. Abrogates binding to the 7-methylguanosine cap of mRNA and prevents inhibition of translation and abolishes interaction with TNRC6C; when associated with V-470., MUTAGEN:F->V: No effect on miRNA-binding or target mRNA cleavage. Abrogates binding to the 7-methylguanosine cap of mRNA and prevents inhibition of translation. Abolishes interaction with TNRC6C; when associated with V-505., MUTAGEN:F->W: No effect on binding to the 7-methylguanosine cap of mRNA or inhibition of translation., MUTAGEN:F->Y: No effect., MUTAGEN:H->A,R: Abrogates RNA cleavage., MUTAGEN:H->P,A: Abrogates RNA cleavage. Binds siRNA., MUTAGEN:H->Y: No effect., MUTAGEN:K->A: Impairs RNA cleavage., MUTAGEN:L->W: No effect., MUTAGEN:P->A: Reduced protein stability., MUTAGEN:Q->A: Impairs RNA cleavage., MUTAGEN:Q->A: No effect., MUTAGEN:Q->R: Abrogates RNA cleavage. Binds siRNA., MUTAGEN:T->Y: No effect., REGION:Disordered, REGION:Interaction with GW182 family members, REGION:Interaction with guide RNA, | | |
| **AMBRA1** | [**autophagy and beclin 1 regulator 1(AMBRA1)**](https://david.ncifcrf.gov/geneReportFull.jsp?rowids=55626) | [**Related Genes**](https://david.ncifcrf.gov/relatedGenes.jsp?id=55626) | [**Homo sapiens**](http://www.ncbi.nlm.nih.gov/Taxonomy/Browser/wwwtax.cgi?name=Homo%20sapiens) |
| **GOTERM_BP_DIRECT** | [autophagosome assembly](http://www.ebi.ac.uk/QuickGO/GTerm?id=GO:0000045), [protein polyubiquitination](http://www.ebi.ac.uk/QuickGO/GTerm?id=GO:0000209), [mitophagy](http://www.ebi.ac.uk/QuickGO/GTerm?id=GO:0000422), [macromitophagy](http://www.ebi.ac.uk/QuickGO/GTerm?id=GO:0000423), [autophagy](http://www.ebi.ac.uk/QuickGO/GTerm?id=GO:0006914), [cell cycle](http://www.ebi.ac.uk/QuickGO/GTerm?id=GO:0007049), [nervous system development](http://www.ebi.ac.uk/QuickGO/GTerm?id=GO:0007399), [negative regulation of cell proliferation](http://www.ebi.ac.uk/QuickGO/GTerm?id=GO:0008285), [cellular response to starvation](http://www.ebi.ac.uk/QuickGO/GTerm?id=GO:0009267), [positive regulation of autophagy](http://www.ebi.ac.uk/QuickGO/GTerm?id=GO:0010508), [negative regulation of cardiac muscle cell apoptotic process](http://www.ebi.ac.uk/QuickGO/GTerm?id=GO:0010667), [macroautophagy](http://www.ebi.ac.uk/QuickGO/GTerm?id=GO:0016236), [neural tube development](http://www.ebi.ac.uk/QuickGO/GTerm?id=GO:0021915), [cell differentiation](http://www.ebi.ac.uk/QuickGO/GTerm?id=GO:0030154), [positive regulation of protein dephosphorylation](http://www.ebi.ac.uk/QuickGO/GTerm?id=GO:0035307), [negative regulation of neuron apoptotic process](http://www.ebi.ac.uk/QuickGO/GTerm?id=GO:0043524), [positive regulation of phosphatidylinositol 3-kinase activity](http://www.ebi.ac.uk/QuickGO/GTerm?id=GO:0043552), [positive regulation of regulatory T cell differentiation](http://www.ebi.ac.uk/QuickGO/GTerm?id=GO:0045591), [response to mitochondrial depolarisation](http://www.ebi.ac.uk/QuickGO/GTerm?id=GO:0098780), [positive regulation of macromitophagy](http://www.ebi.ac.uk/QuickGO/GTerm?id=GO:1901526), [positive regulation of free ubiquitin chain polymerization](http://www.ebi.ac.uk/QuickGO/GTerm?id=GO:1904544), [regulation of G1/S transition of mitotic cell cycle](http://www.ebi.ac.uk/QuickGO/GTerm?id=GO:2000045), | | |
| **GOTERM_CC_DIRECT** | [nucleus](http://www.ebi.ac.uk/QuickGO/GTerm?id=GO:0005634), [cytoplasm](http://www.ebi.ac.uk/QuickGO/GTerm?id=GO:0005737), [mitochondrion](http://www.ebi.ac.uk/QuickGO/GTerm?id=GO:0005739), [mitochondrial outer membrane](http://www.ebi.ac.uk/QuickGO/GTerm?id=GO:0005741), [autophagosome](http://www.ebi.ac.uk/QuickGO/GTerm?id=GO:0005776), [endoplasmic reticulum](http://www.ebi.ac.uk/QuickGO/GTerm?id=GO:0005783), [cytosol](http://www.ebi.ac.uk/QuickGO/GTerm?id=GO:0005829), [cytoskeleton](http://www.ebi.ac.uk/QuickGO/GTerm?id=GO:0005856), [focal adhesion](http://www.ebi.ac.uk/QuickGO/GTerm?id=GO:0005925), [axoneme](http://www.ebi.ac.uk/QuickGO/GTerm?id=GO:0005930), [cytoplasmic vesicle](http://www.ebi.ac.uk/QuickGO/GTerm?id=GO:0031410), [intracellular membrane-bounded organelle](http://www.ebi.ac.uk/QuickGO/GTerm?id=GO:0043231), [phagocytic vesicle](http://www.ebi.ac.uk/QuickGO/GTerm?id=GO:0045335), [perinuclear region of cytoplasm](http://www.ebi.ac.uk/QuickGO/GTerm?id=GO:0048471), [Cul4-RING E3 ubiquitin ligase complex](http://www.ebi.ac.uk/QuickGO/GTerm?id=GO:0080008), | | |
| **GOTERM_MF_DIRECT** | [protein binding](http://www.ebi.ac.uk/QuickGO/GTerm?id=GO:0005515), [protein phosphatase binding](http://www.ebi.ac.uk/QuickGO/GTerm?id=GO:0019903), [ubiquitin protein ligase binding](http://www.ebi.ac.uk/QuickGO/GTerm?id=GO:0031625), [GTPase binding](http://www.ebi.ac.uk/QuickGO/GTerm?id=GO:0051020), [protein binding, bridging involved in substrate recognition for ubiquitination](http://www.ebi.ac.uk/QuickGO/GTerm?id=GO:1990756), | | |
[truncated: 1,730,806 more chars]
