# Supplementary material for: Role of epigenetics in the clinical evolution of COVID-19 disease. Epigenome-wide association study identifies markers of severe outcome
Source: Eur J Med Res. 2023 Feb 17;28:81. doi: 10.1186/s40001-023-01032-7 (PMC9936487; doi:10.1186/s40001-023-01032-7)
Supplement: Supplementary file 4 — Additional file 4: Extended results. [file 40001_2023_1032_MOESM4_ESM.docx]

***European Journal of Medical Research***

**Additional Results**

**Summary of extended results:**

**1) Average DNA Methylation evaluation**

**2)** **Epigenetic Clock**

*Cellular Component Estimation*

*Chronological Age*

*Chronological Age vs Biological Age (Horvath epigenetic clock)*

**3) Sample Group Analysis**

*Exploratory analyses: quality control and batch effect evaluation*

*Differential methylation analysis at regional-level:*

*Genes*

*Promoters*

**4) Validation of the 21 CpG sites' signature**

*a) Validation in a cohort of 75 covid negative (Covid-) subjects*

*By using GEO datasets:*

*b) GSE168739*

*c) GSE167202*

*d) GSE174818*

**6) Stochastic Epigenetic Mutations (SEMs)**

*Evaluation of hyper- and hypo-methylated SEMs for:*

*This study (Covid+Severe vs Covid+Mild)*

*GSE167202*

*GSE174818*

**1) Average DNA Methylation**

Differences in the average DNA methylation level between groups (Covid+Mild vs Covid+Severe) were evaluated by comparing the distribution of beta values of a subset of 3773 loci classified as “Random Loci”: these probes were chosen by consortium members during the array design process as a representative of the genome-wide status. As shown in the next figure (**Figure S1**), β-value distributions are almost completely overlapping, so no significant differences were found (Kolmogorov-Smirnov test: p=0.9181).


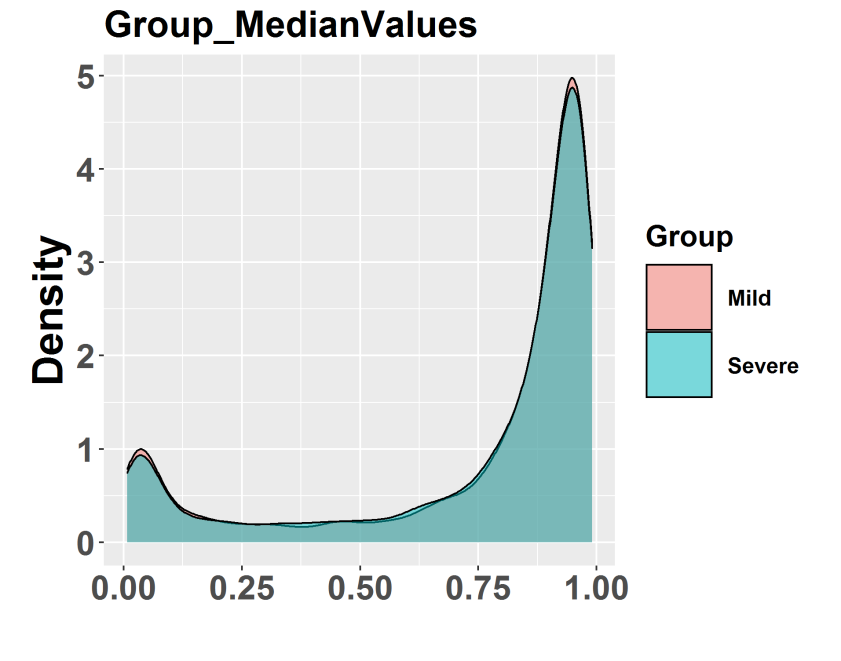


**Figure S1**: Density plot showing the distribution of beta-values of 3773 loci classified as “Random Loci in the two cohorts.

**2) Epigenetic Clock**

*Cellular Component Estimation*

Steve Horvath’s Epigenetic Clock was used to check for differences in blood cell composition in the two groups (Mann-Whitney tests). Results are shown in the figures as boxplots (**Figure S2**). We found the strongest differences for CD4T, Gran, PlasmaBlast, and CD8pCD28nCD45Ran cells.


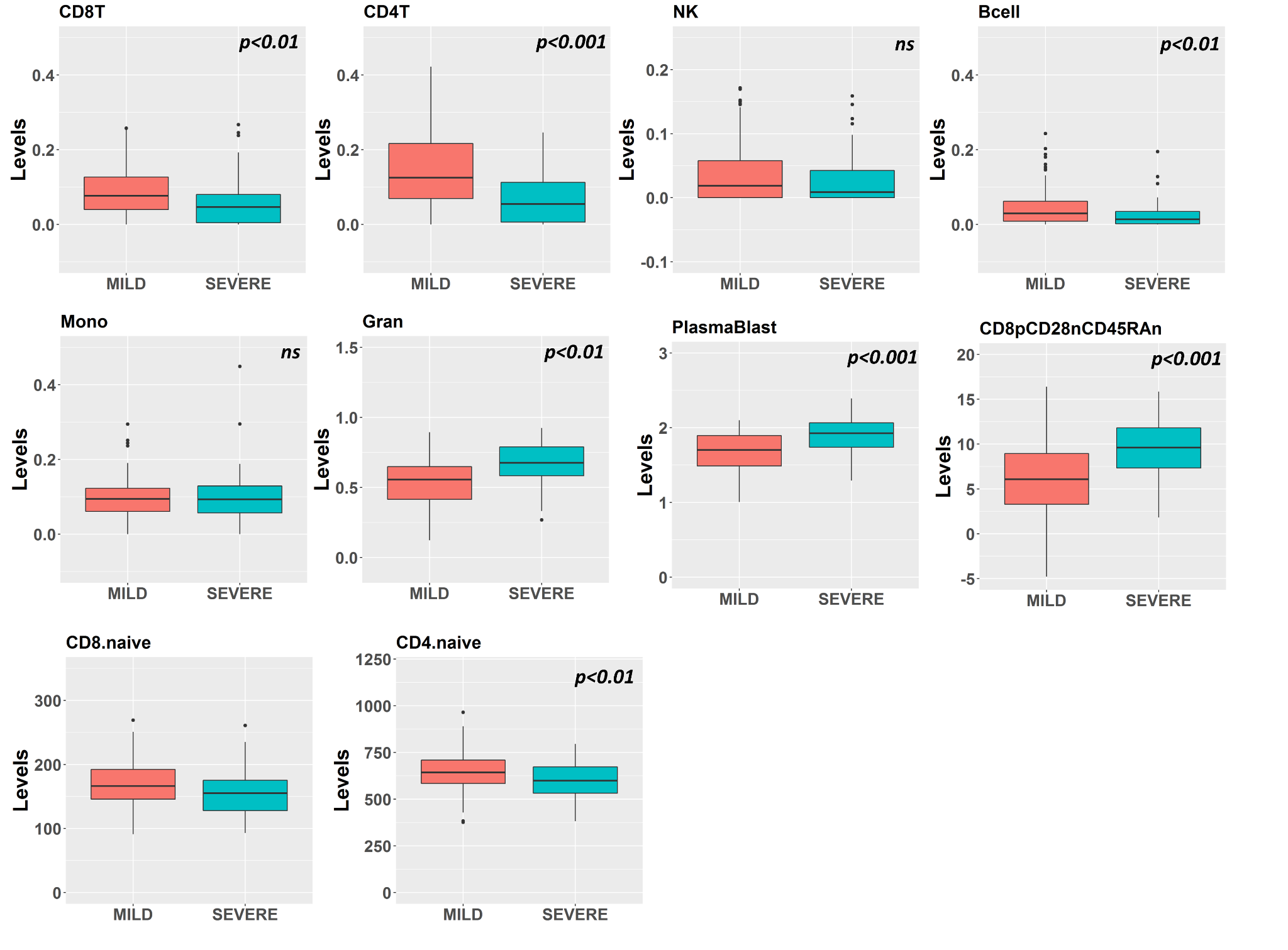


**Figure S2**: Boxplot showing the estimates of the cellular composition (Steve Horvath’s Epigenetic Clock) between the tho groups. The thick horizontal line in the box represents the median of the distribution while the box represents the interquartile range. Whiskers are set as the default option for the “ggplot” boxplot function and extend to the most extreme data point, which is no more than 1.5 times the interquartile range from the box. Dots represent outliers (single values exceeding 1.5 interquartile ranges).

The degree of association/relationship of some cellular components with the outcome (Sample_Group) is also visualized through the next correlogram (**Figure S3**).


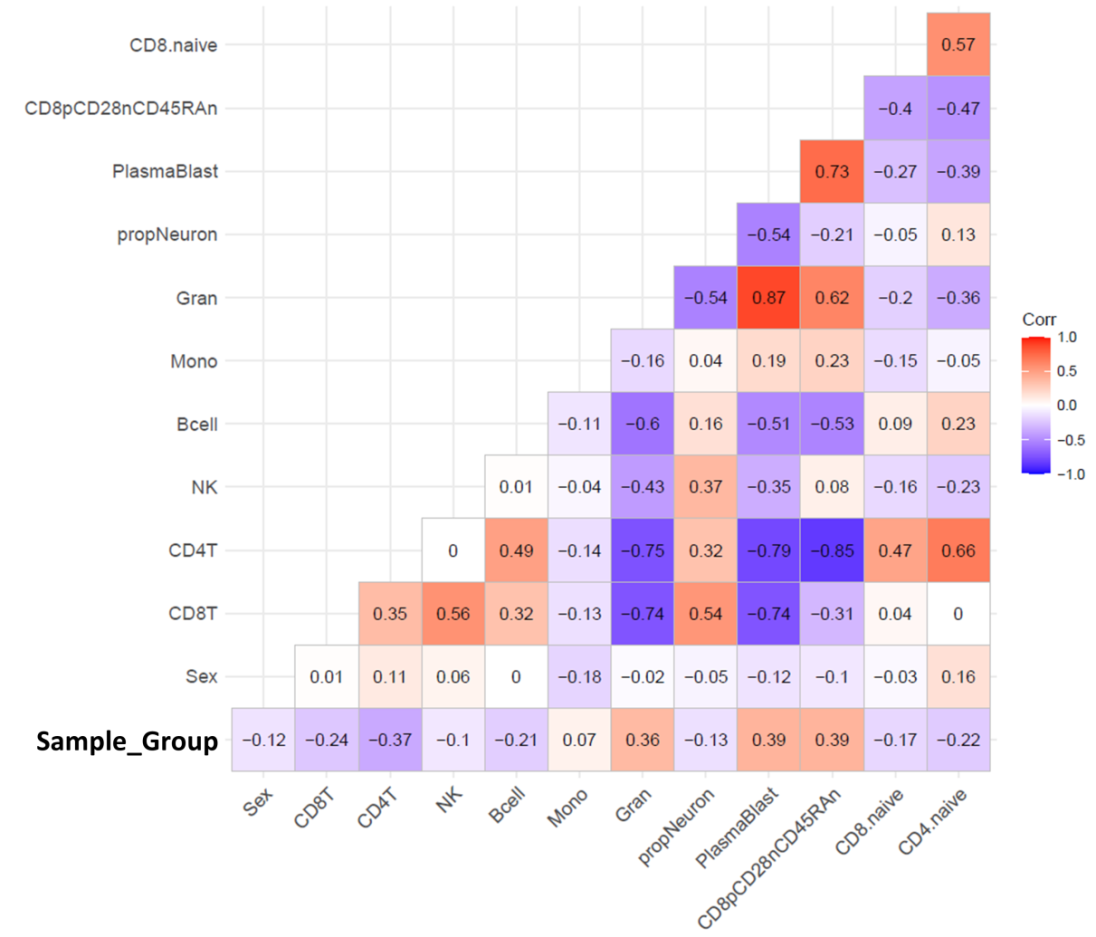


**Figure S3**: correlogram showing the degree of the correlation of cellular components ad the variable Sample Group

*Chronological Age*

No significant differences related to age are observed between the two cohorts (Mann-Whitney test, p=0.2) (**Figure S4**).


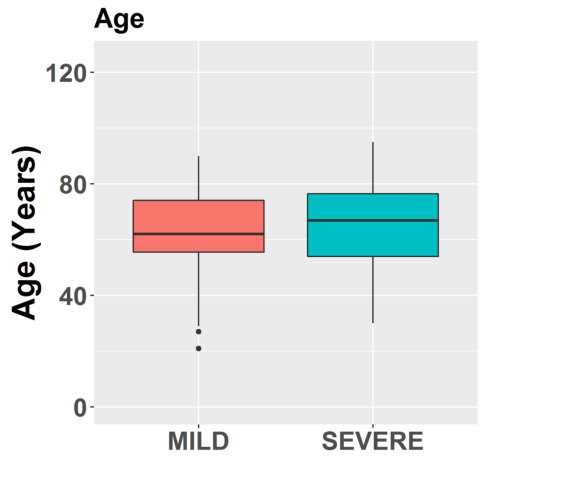


**Figure S4**: Boxplot showing the distribution of chronological age in mild (controls) and severe patients.

The comparison estimates of the cellular composition (Steve Horvath’s Epigenetic Clock) between the two groups. The thick horizontal line in the box represents the median of the distribution while the box represents the interquartile range. Whiskers are set as the default option for the “ggplot” boxplot function and extend to the most extreme data point, which is no more than 1.5 times the interquartile range from the box. Dots represent outliers (single values exceeding 1.5 interquartile ranges).

*Chronological Age vs Biological Age (Horvath epigenetic clock)*

We took advantage of Steve Horvath’s Epigenetic Clock to measure biological age: the two cohorts did not show appreciable differences (Chronological Age vs Biological Age (DNAmAge) (Mann-Whitney test, Mild cohort: p= 0.071; Severe cohort: p= 0.327) (**Figures S5 and S6**).

Mild Cohort:


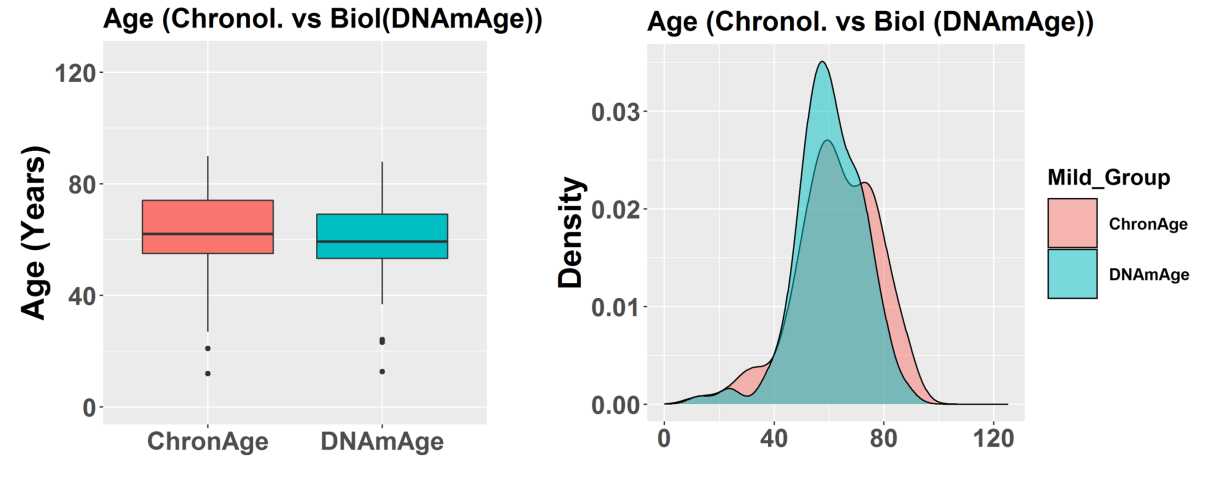


**Figure S5:** boxplots and density plots of age variables (chronological vs biological (Horvath DNAmAge))

Severe Cohort:


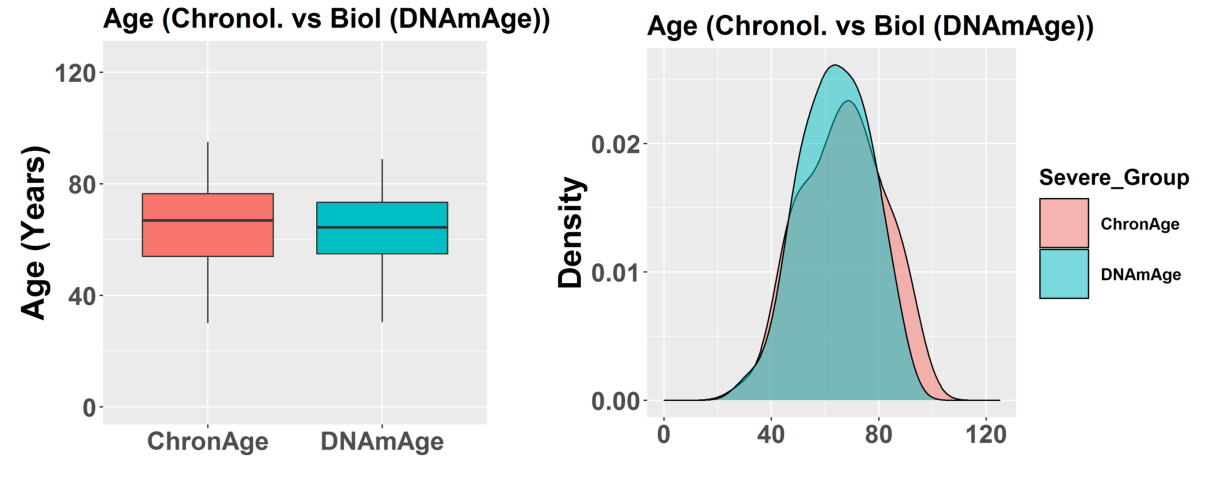


**Figure S6:** boxplots and density plots of age variables (chronological vs biological (Horvath DNAmAge))

**3) Sample group analysis (Covid+ Mild vs Covid+ Severe)**

*1.Exploratory analyses: quality control and batch effect evaluation*

Sample group-level analysis was carried out by using RnBeads. An explorative principal component analysis (PCA) was then used to reduce data complexity and explore the methylation profiles of the 190 COVID-19 patients. As shown in the next PCA scatter plot (**Figure S7**), three samples (two severe and 1 mild) resulted non-eligible for analysis (data points excessively separated from the whole group of patients).


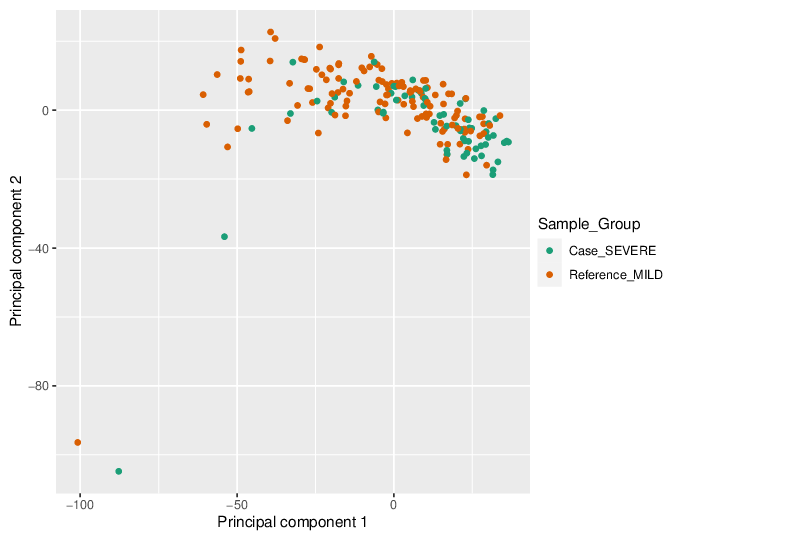


**Figure S7:** Scatter plot distribution of samples along with the first two principal components at site level before quality control.

After the exclusion of 3 patients, the final cohort was constituted of 187 methylation profiles. SNP-enriched probes (139,721 sites), cross-reactive probes (34,264), unreliable measurements (4,369 sites), context-specific (1,180 sites), and on sex chromosomes probes (16,347sites), probes with many missing values (141 sites) were filtered out in the pre-processing step. As an outcome of the filtering procedures, 670,873 CpG sites and 187 samples were retained.

Since the samples were processed in two consecutive batches, potential batch effects were, at first, evaluated: as shown in the next figure (**Figure S8**), we have ruled out batch effect as a covariate, as the samples, differently colored according to the processing batch (A and B), are evenly distributed along with the two-dimensional panel.


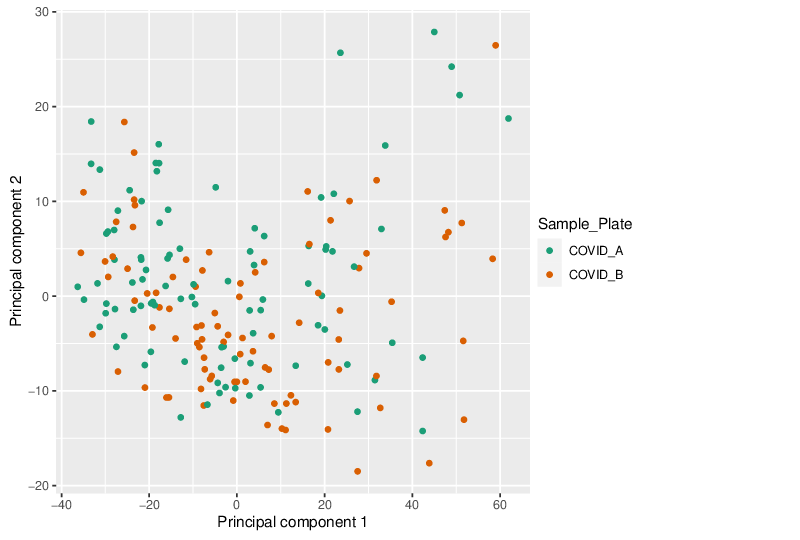


Figure S8: Scatter plot distribution of samples along with the first two principal components at the site level after quality control.

*2. Differential methylation analysis at regional-level*

The differential analysis was also performed at the region level (genes, promoters, CpG Islands, and tiling).

**Genes:**

Concerning gene classification, the comparison did not substantially produce any strong statistical result (adj.FDR.pvalue ≤ 0.05) (Figure S9).


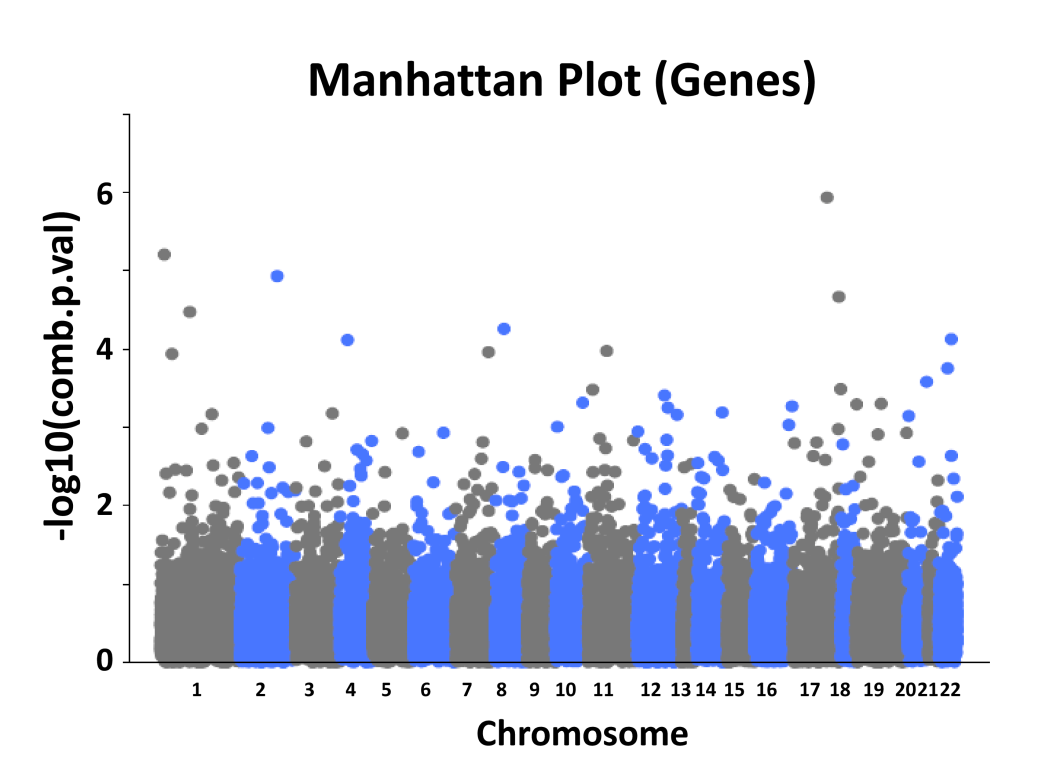


**Figure S9:** Manhattan plot showing the distribution of p-values of differentially methylated genes. The ordinate axis represents the negative log10 of the unadjusted p-value of methylation mean differences between “severe” and control “mild” groups while the abscissa axis is the location of differentially methylated points in relation to chromosomes.

Alternatively to adjusted p-value thresholds, was also assigned each region a combined rank value based on three metrics such as i) the mean difference in means across all sites in a region of the two groups, ii) the mean of quotients in mean methylation and, iii) a combined p-value calculated from all site p-values in the region. The smaller the combined rank for a region, the more evidence for epigenetic differences it exhibits. We then performed a gene ontology (GO) enrichment analysis through the relative RnBeads module by considering the top 100 ranked genes (hypermethylated and hypomethylated, separately).

To test the connection between these genes, we conducted a gene ontology analysis by considering these two lists separately. The results of the analyses are reported below (**Tables S1 and S2**).

**Table S1- Hyper-methylated genes**

| **GOMFID** | **Pvalue** | **OddsRatio** | **ExpCount** | **Count** | **Size** | **Term** |
| --- | --- | --- | --- | --- | --- | --- |
| GO:0002376 | 0 | 13.0598 | 1.2348 | 9 | 970 | immune system process |
| GO:0050863 | 0 | 15.2375 | 0.5061 | 6 | 305 | regulation of T cell activation |
| GO:0046640 | 0 | 75.0833 | 0.0498 | 3 | 30 | regulation of alpha-beta T cell proliferation |
| GO:0032819 | 1.00E-04 | 259.84 | 0.0116 | 2 | 7 | positive regulation of natural killer cell proliferation |
| GO:0045058 | 1.00E-04 | 44.019 | 0.0813 | 3 | 49 | T cell selection |
| GO:0045071 | 1.00E-04 | 39.6912 | 0.0896 | 3 | 54 | negative regulation of viral genome replication |
| GO:0043207 | 1.00E-04 | 6.061 | 2.0691 | 9 | 1247 | response to external biotic stimulus |
| GO:0046635 | 1.00E-04 | 35.5 | 0.0996 | 3 | 60 | positive regulation of alpha-beta T cell activation |
| GO:0002694 | 1.00E-04 | 9.1099 | 0.8296 | 6 | 500 | regulation of leukocyte activation |
| GO:1903555 | 2.00E-04 | 17.053 | 0.2788 | 4 | 168 | regulation of tumor necrosis factor superfamily cytokine production |
| GO:0050867 | 2.00E-04 | 11.3829 | 0.536 | 5 | 323 | positive regulation of cell activation |
| GO:0002517 | 2.00E-04 | 129.88 | 0.0199 | 2 | 12 | T cell tolerance induction |
| GO:0032729 | 2.00E-04 | 31.6035 | 0.1112 | 3 | 67 | positive regulation of interferon-gamma production |
| GO:0045059 | 2.00E-04 | 108.22 | 0.0232 | 2 | 14 | positive thymic T cell selection |
| GO:0050829 | 2.00E-04 | 28.4754 | 0.1228 | 3 | 74 | defense response to Gram-negative bacterium |
| GO:0009617 | 3.00E-04 | 7.6484 | 0.9806 | 6 | 591 | response to bacterium |
| GO:0016032 | 4.00E-04 | 9.3129 | 0.6504 | 5 | 392 | viral process |
| GO:0002250 | 4.00E-04 | 9.2395 | 0.6554 | 5 | 395 | adaptive immune response |
| GO:1903039 | 4.00E-04 | 12.9058 | 0.365 | 4 | 220 | positive regulation of leukocyte cell-cell adhesion |
| GO:0042102 | 5.00E-04 | 22.691 | 0.1527 | 3 | 92 | positive regulation of T cell proliferation |
| GO:0035455 | 5.00E-04 | 72.12 | 0.0332 | 2 | 20 | response to interferon-alpha |
| GO:0046597 | 5.00E-04 | 72.12 | 0.0332 | 2 | 20 | negative regulation of viral entry into host cell |
| GO:0098542 | 5.00E-04 | 5.9535 | 1.5083 | 7 | 909 | defense response to other organism |
| GO:0032760 | 6.00E-04 | 21.25 | 0.1626 | 3 | 98 | positive regulation of tumor necrosis factor production |
| GO:0042221 | 6.00E-04 | 3.8228 | 6.667 | 15 | 4018 | response to chemical |
| GO:0032753 | 6.00E-04 | 64.9 | 0.0365 | 2 | 22 | positive regulation of interleukin-4 production |
| GO:0051607 | 6.00E-04 | 11.6967 | 0.4015 | 4 | 242 | defense response to virus |
| GO:0001916 | 8.00E-04 | 56.4243 | 0.0415 | 2 | 25 | positive regulation of T cell mediated cytotoxicity |
| GO:0051251 | 9.00E-04 | 10.5684 | 0.443 | 4 | 267 | positive regulation of lymphocyte activation |
| GO:0001816 | 0.001 | 6.1877 | 1.1997 | 6 | 723 | cytokine production |
| GO:0035456 | 0.0011 | 46.3343 | 0.0498 | 2 | 30 | response to interferon-beta |
| GO:0032946 | 0.0012 | 15.9911 | 0.214 | 3 | 129 | positive regulation of mononuclear cell proliferation |
| GO:0032814 | 0.0014 | 41.8426 | 0.0548 | 2 | 33 | regulation of natural killer cell activation |
| GO:1903900 | 0.0016 | 14.5897 | 0.234 | 3 | 141 | regulation of viral life cycle |
| GO:0001815 | 0.0017 | Inf | 0.0017 | 1 | 1 | positive regulation of antibody-dependent cellular cytotoxicity |
| GO:0021509 | 0.0017 | Inf | 0.0017 | 1 | 1 | roof plate formation |
| GO:1901835 | 0.0017 | Inf | 0.0017 | 1 | 1 | positive regulation of deadenylation-independent decapping of nuclear-transcribed mRNA |
| GO:1904246 | 0.0017 | Inf | 0.0017 | 1 | 1 | negative regulation of polynucleotide adenylyltransferase activity |
| GO:2001051 | 0.0017 | Inf | 0.0017 | 1 | 1 | positive regulation of tendon cell differentiation |
| GO:0031295 | 0.0018 | 36.02 | 0.0631 | 2 | 38 | T cell costimulation |
| GO:0051716 | 0.0019 | 3.4381 | 11.0442 | 19 | 6656 | cellular response to stimulus |
| GO:0048534 | 0.002 | 5.3471 | 1.3772 | 6 | 830 | hematopoietic or lymphoid organ development |
| GO:0002520 | 0.0024 | 31.1113 | 0.0734 | 2 | 54 | immune system development |
| GO:0030098 | 0.0025 | 7.9679 | 0.5824 | 4 | 351 | lymphocyte differentiation |
| GO:0052126 | 0.0026 | 12.1818 | 0.2788 | 3 | 168 | movement in host environment |
| GO:0052372 | 0.0027 | 28.8 | 0.078 | 2 | 47 | modulation by symbiont of entry into host |
| GO:0030855 | 0.0028 | 5.9467 | 1.0006 | 5 | 603 | epithelial cell differentiation |
| GO:0060337 | 0.0029 | 28.1722 | 0.0796 | 2 | 48 | type I interferon signaling pathway |
| GO:0023052 | 0.0031 | 3.1247 | 9.526 | 17 | 5741 | signaling |
| GO:0002894 | 0.0033 | 624.7692 | 0.0033 | 1 | 2 | positive regulation of type II hypersensitivity |
| GO:0030573 | 0.0033 | 624.7692 | 0.0033 | 1 | 2 | bile acid catabolic process |
| GO:0030887 | 0.0033 | 624.7692 | 0.0033 | 1 | 2 | positive regulation of myeloid dendritic cell activation |
| GO:0032759 | 0.0033 | 624.7692 | 0.0033 | 1 | 2 | positive regulation of TRAIL production |
| GO:2000566 | 0.0033 | 624.7692 | 0.0033 | 1 | 2 | positive regulation of CD8-positive, alpha-beta T cell proliferation |
| GO:0007154 | 0.0034 | 3.0837 | 9.6073 | 17 | 5790 | cell communication |
| GO:0034340 | 0.0039 | 23.9867 | 0.0929 | 2 | 56 | response to type I interferon |
| GO:0031343 | 0.004 | 23.5491 | 0.0946 | 2 | 57 | positive regulation of cell killing |
| GO:0032663 | 0.0041 | 23.1271 | 0.0962 | 2 | 58 | regulation of interleukin-2 production |
| GO:0045785 | 0.0046 | 6.7169 | 0.6869 | 4 | 414 | positive regulation of cell adhesion |
| GO:0022407 | 0.0047 | 6.6503 | 0.6936 | 4 | 418 | regulation of cell-cell adhesion |
| GO:0050670 | 0.0049 | 9.7324 | 0.3468 | 3 | 209 | regulation of lymphocyte proliferation |
| GO:0001796 | 0.005 | 312.3654 | 0.005 | 1 | 3 | regulation of type IIa hypersensitivity |
| GO:0002477 | 0.005 | 312.3654 | 0.005 | 1 | 3 | antigen processing and presentation of exogenous peptide antigen via MHC class Ib |
| GO:0002519 | 0.005 | 312.3654 | 0.005 | 1 | 3 | natural killer cell tolerance induction |
| GO:0003245 | 0.005 | 312.3654 | 0.005 | 1 | 3 | cardiac muscle tissue growth involved in heart morphogenesis |
| GO:0035992 | 0.005 | 312.3654 | 0.005 | 1 | 3 | tendon formation |
| GO:0071409 | 0.005 | 312.3654 | 0.005 | 1 | 3 | cellular response to cycloheximide |
| GO:0097533 | 0.005 | 312.3654 | 0.005 | 1 | 3 | cellular stress response to acid chemical |
| GO:1904582 | 0.005 | 312.3654 | 0.005 | 1 | 3 | positive regulation of intracellular mRNA localization |
| GO:0071356 | 0.005 | 9.6376 | 0.3501 | 3 | 211 | cellular response to tumor necrosis factor |
| GO:0007186 | 0.0052 | 4.3744 | 1.6626 | 6 | 1002 | G protein-coupled receptor signaling pathway |
| GO:0044419 | 0.0052 | 4.4473 | 1.6799 | 6 | 1120 | biological process involved in interspecies interaction between organisms |
| GO:0006950 | 0.0062 | 2.9399 | 5.786 | 12 | 3487 | response to stress |
| GO:0070663 | 0.0063 | 8.8601 | 0.38 | 3 | 229 | regulation of leukocyte proliferation |
| GO:0010628 | 0.0066 | 4.1516 | 1.7456 | 6 | 1052 | positive regulation of gene expression |
| GO:0001766 | 0.0066 | 208.2308 | 0.0066 | 1 | 4 | membrane raft polarization |
| GO:0002669 | 0.0066 | 208.2308 | 0.0066 | 1 | 4 | positive regulation of T cell anergy |
| GO:0051142 | 0.0066 | 208.2308 | 0.0066 | 1 | 4 | positive regulation of NK T cell proliferation |
| GO:2000330 | 0.0066 | 208.2308 | 0.0066 | 1 | 4 | positive regulation of T-helper 17 cell lineage commitment |
| GO:0033077 | 0.0067 | 17.97 | 0.1228 | 2 | 74 | T cell differentiation in thymus |
| GO:0022610 | 0.0069 | 3.6968 | 2.3429 | 7 | 1412 | biological adhesion |
| GO:0001910 | 0.007 | 17.4822 | 0.1261 | 2 | 76 | regulation of leukocyte mediated cytotoxicity |
| GO:0002709 | 0.007 | 17.4822 | 0.1261 | 2 | 76 | regulation of T cell mediated immunity |
| GO:0097305 | 0.0071 | 8.443 | 0.3982 | 3 | 240 | response to alcohol |
| GO:0050911 | 0.0073 | 8.3713 | 0.4015 | 3 | 242 | detection of chemical stimulus involved in sensory perception of smell |
| GO:0002684 | 0.0077 | 4.6307 | 1.2694 | 5 | 765 | positive regulation of immune system process |
| GO:0002476 | 0.0083 | 156.1635 | 0.0083 | 1 | 5 | antigen processing and presentation of endogenous peptide antigen via MHC class Ib |
| GO:0002486 | 0.0083 | 156.1635 | 0.0083 | 1 | 5 | antigen processing and presentation of endogenous peptide antigen via MHC class I via ER pathway, TAP-independent |
| GO:0010593 | 0.0083 | 156.1635 | 0.0083 | 1 | 5 | negative regulation of lamellipodium assembly |
| GO:0042270 | 0.0083 | 156.1635 | 0.0083 | 1 | 5 | protection from natural killer cell mediated cytotoxicity |
| GO:0048386 | 0.0083 | 156.1635 | 0.0083 | 1 | 5 | positive regulation of retinoic acid receptor signaling pathway |
| GO:0060509 | 0.0083 | 156.1635 | 0.0083 | 1 | 5 | type I pneumocyte differentiation |
| GO:0034103 | 0.0083 | 15.9644 | 0.1377 | 2 | 83 | regulation of tissue remodeling |
| GO:0048872 | 0.0083 | 7.9651 | 0.4215 | 3 | 254 | homeostasis of number of cells |
| GO:0097581 | 0.0087 | 15.5778 | 0.141 | 2 | 85 | lamellipodium organization |
| GO:0050830 | 0.0089 | 15.3914 | 0.1427 | 2 | 86 | defense response to Gram-positive bacterium |
| GO:0002521 | 0.0096 | 5.3875 | 0.8496 | 4 | 512 | leukocyte differentiation |
| GO:0002866 | 0.0099 | 124.9231 | 0.01 | 1 | 6 | positive regulation of acute inflammatory response to antigenic stimulus |
| GO:0010536 | 0.0099 | 124.9231 | 0.01 | 1 | 6 | positive regulation of activation of Janus kinase activity |
| GO:0033634 | 0.0099 | 124.9231 | 0.01 | 1 | 6 | positive regulation of cell-cell adhesion mediated by integrin |
| GO:1902172 | 0.0099 | 124.9231 | 0.01 | 1 | 6 | regulation of keratinocyte apoptotic process |
| GO:0044403 | 0.0099 | 7.452 | 0.4497 | 3 | 271 | biological process involved in symbiotic interaction |

**Table S2 - Hypo-methylated genes**

| **GOMFID** | **Pvalue** | **OddsRatio** | **ExpCount** | **Count** | **Size** | **Term** |
| --- | --- | --- | --- | --- | --- | --- |
| GO:0002523 | 2.00E-04 | 119.3897 | 0.021 | 2 | 19 | leukocyte migration involved in inflammatory response |
| GO:0010920 | 0.0011 | Inf | 0.0011 | 1 | 1 | negative regulation of inositol phosphate biosynthetic process |
| GO:0010925 | 0.0011 | Inf | 0.0011 | 1 | 1 | positive regulation of inositol-polyphosphate 5-phosphatase activity |
| GO:0030845 | 0.0011 | Inf | 0.0011 | 1 | 1 | phospholipase C-inhibiting G protein-coupled receptor signaling pathway |
| GO:0060305 | 0.0011 | Inf | 0.0011 | 1 | 1 | regulation of cell diameter |
| GO:0035606 | 0.0022 | 956.0588 | 0.0022 | 1 | 2 | peptidyl-cysteine S-trans-nitrosylation |
| GO:0036316 | 0.0022 | 956.0588 | 0.0022 | 1 | 2 | SREBP-SCAP complex retention in endoplasmic reticulum |
| GO:0070488 | 0.0022 | 956.0588 | 0.0022 | 1 | 2 | neutrophil aggregation |
| GO:0070560 | 0.0022 | 956.0588 | 0.0022 | 1 | 2 | protein secretion by platelet |
| GO:0045113 | 0.0033 | 478 | 0.0033 | 1 | 3 | regulation of integrin biosynthetic process |
| GO:0062014 | 0.0048 | 21.9592 | 0.104 | 2 | 94 | negative regulation of small molecule metabolic process |
| GO:0032496 | 0.0052 | 9.8644 | 0.3606 | 3 | 326 | response to lipopolysaccharide |
| GO:0010572 | 0.0055 | 238.9706 | 0.0055 | 1 | 5 | positive regulation of platelet activation |
| GO:0010706 | 0.0055 | 238.9706 | 0.0055 | 1 | 5 | ganglioside biosynthetic process via lactosylceramide |
| GO:0050727 | 0.0066 | 9.0352 | 0.3927 | 3 | 355 | regulation of inflammatory response |
| GO:0021782 | 0.0069 | 18.0156 | 0.1261 | 2 | 114 | glial cell development |
| GO:0045630 | 0.0077 | 159.2941 | 0.0077 | 1 | 7 | positive regulation of T-helper 2 cell differentiation |
| GO:2000638 | 0.0077 | 159.2941 | 0.0077 | 1 | 7 | regulation of SREBP signaling pathway |
| GO:0043280 | 0.008 | 16.6663 | 0.1361 | 2 | 123 | positive regulation of cysteine-type endopeptidase activity involved in apoptotic process |
| GO:0030311 | 0.0088 | 136.5294 | 0.0088 | 1 | 8 | poly-N-acetyllactosamine biosynthetic process |
| GO:0032119 | 0.0088 | 136.5294 | 0.0088 | 1 | 8 | sequestering of zinc ion |
| GO:0035425 | 0.0088 | 136.5294 | 0.0088 | 1 | 8 | autocrine signaling |
| GO:0051584 | 0.0088 | 136.5294 | 0.0088 | 1 | 8 | regulation of dopamine uptake involved in synaptic transmission |
| GO:0060363 | 0.0088 | 136.5294 | 0.0088 | 1 | 8 | cranial suture morphogenesis |
| GO:2000553 | 0.0088 | 136.5294 | 0.0088 | 1 | 8 | positive regulation of T-helper 2 cell cytokine production |
| GO:0046165 | 0.009 | 15.625 | 0.1449 | 2 | 131 | alcohol biosynthetic process |
| GO:0035973 | 0.0099 | 119.4559 | 0.01 | 1 | 9 | aggrephagy |
| GO:1900244 | 0.0099 | 119.4559 | 0.01 | 1 | 9 | positive regulation of synaptic vesicle endocytosis |

The enrichment is summarized/classified as Gene Ontology tree-maps (Revigo) for hyper- (**Figure S10**) and hypo-methylated (**Figure S11**) GO terms. The clustering of GO biological processes was visualized using a tool such as ReviGO (<http://revigo.irb.hr/>) (**Supek et al., 2011**) setting the parameter “Allowed similarity” to Medium (0.7) and referring to the UniProt-to-GO mapping file "goa_uniprot_gcrp.gaf.gz" dated 15 March 2017. Clustering semantically divided GO-terms enriched in hyper- and hypo-methylated gene regions into a few main categories.


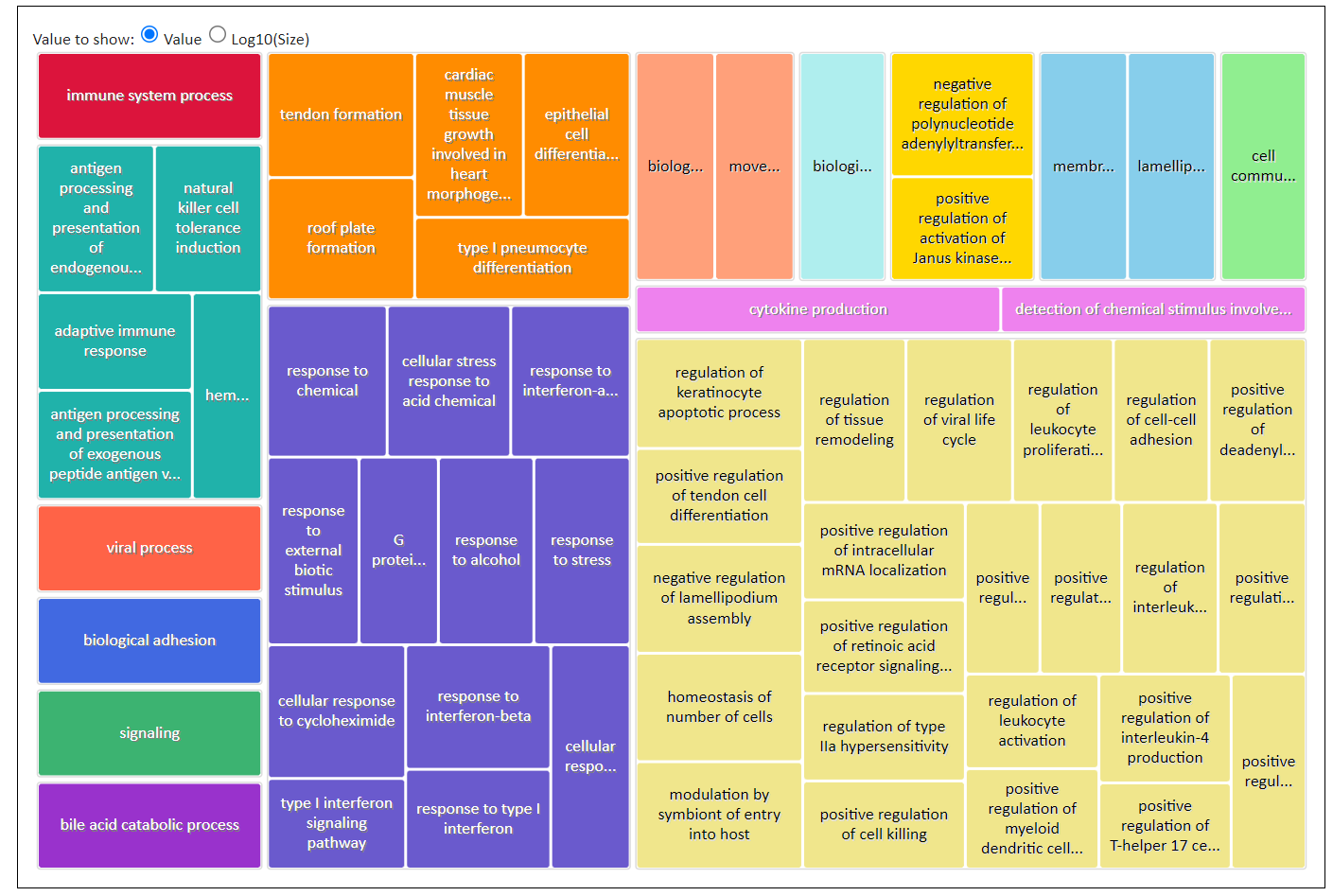


**Figure S10**: REVIGO treemap summarizing Gene Ontology (GO) enrichment analysis on genes enriched in hyper-methylated sites.


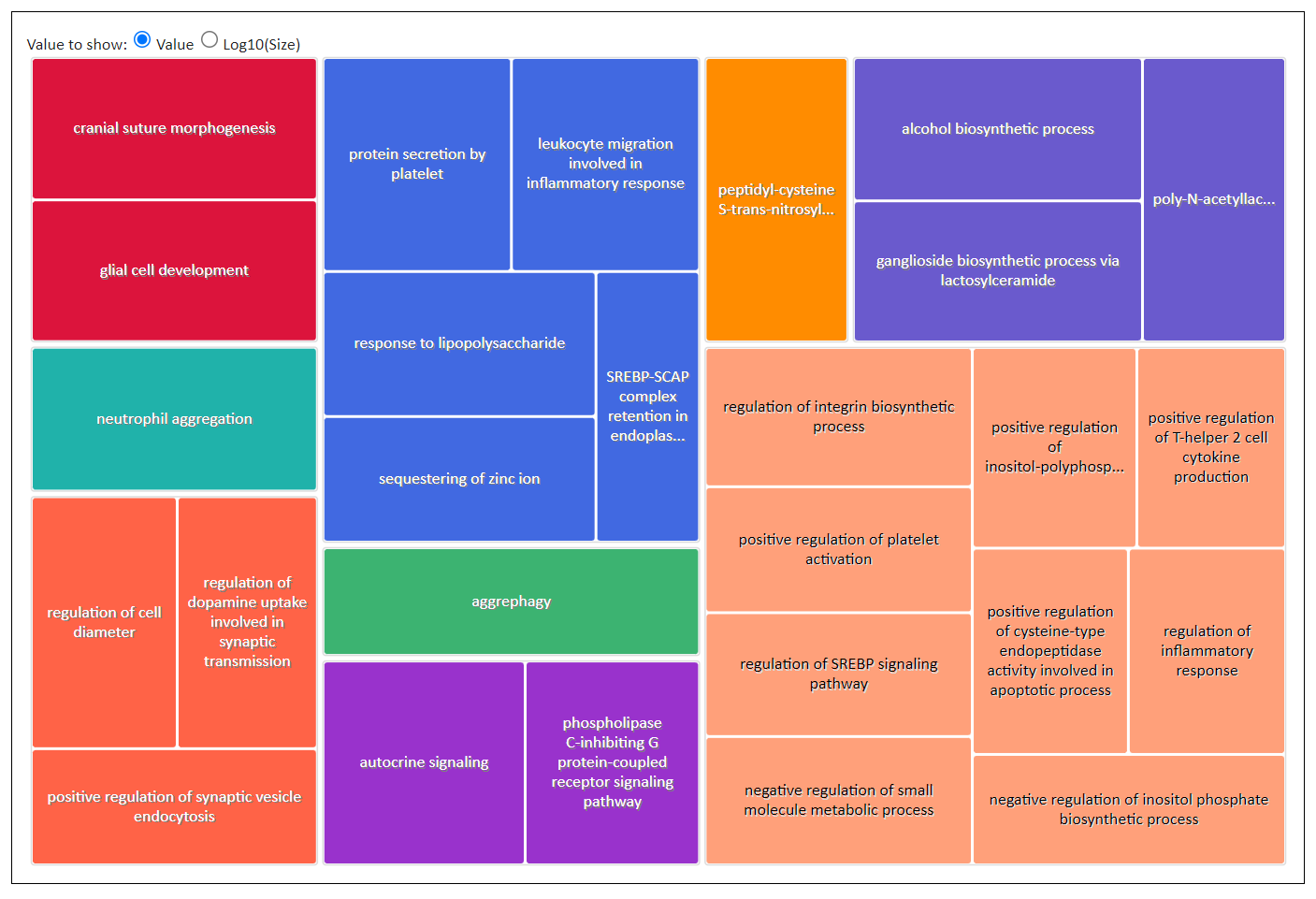


**Figure S11:** REVIGO treemap summarizing Gene Ontology (GO) enrichment analysis on genes enriched in hypo-methylated sites.

The top 100 ranked differentially methylated gene list was further investigated by using the Gene Set Enrichment Analysis (GSEA) (Mootha V.K. et al., 2003; Subramanian A. et al., 2005) through the WEB-based GEne SeT AnaLysis Toolkit (WebGestalt - http://www.webgestalt.org/). The results are shown as a Bar chart (Figure S12).


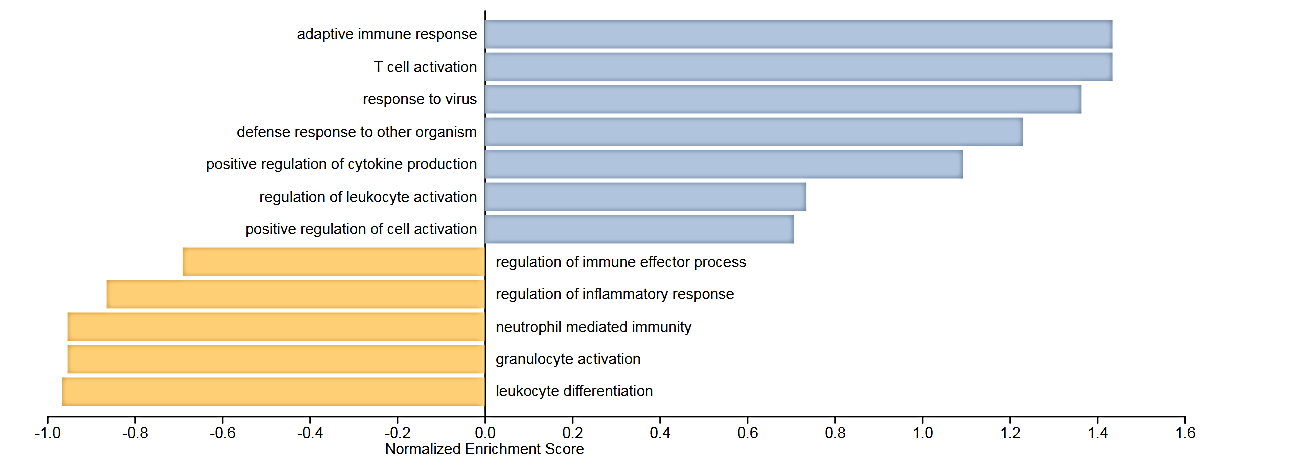


**Figure S12:** Results of gene set enrichment analysis (GSEA). Bars represent pathways/GO terms enriched in hyper- (light blue) and hypo-methylated (yellow) genes, respectively. Categories are indicated using the enrichment of genes present in each category and the number of genes identified in the GO library (p < 0.05).

Confirming the previous results we found significant enrichment of immune-related terms including “adaptative immune response” o “response to virus” (among “hyper-methylated” terms) and “regulation of inflammatory response” (among “hypo-methylated” terms).

**Promoters:**

The same approach was applied to promoter regions as the comparison yielded the same results obtained for gene regions (**Figure S13**).


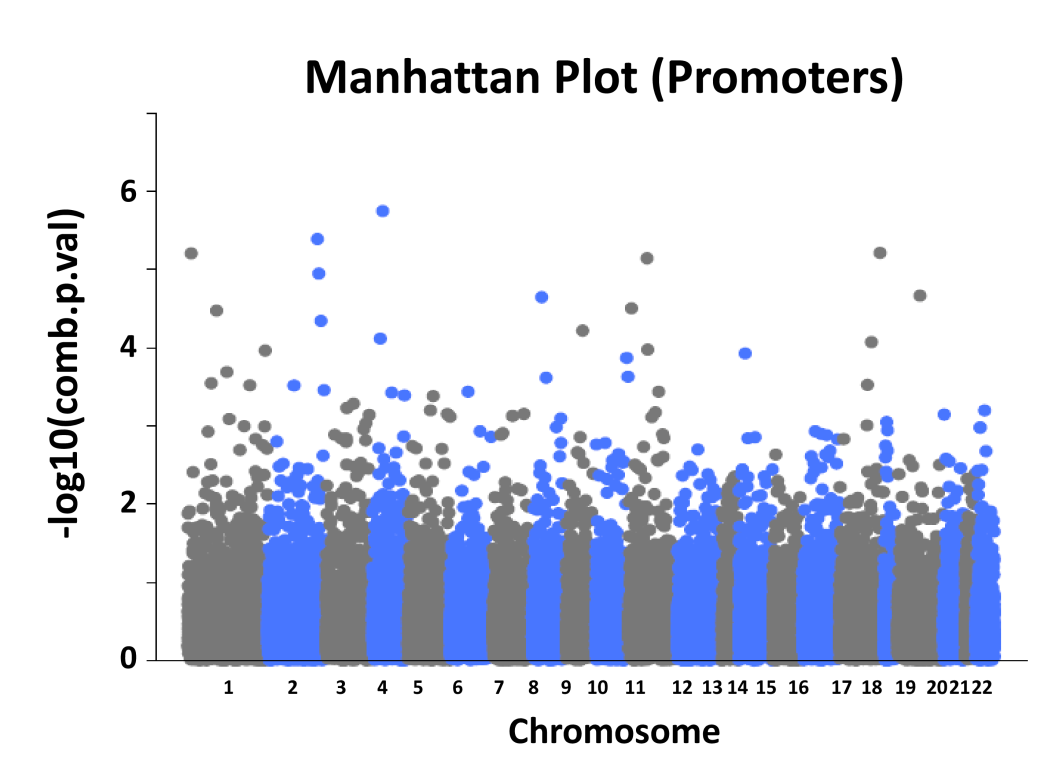


**Figure S13**: Manhattan plot showing the distribution of p-values of differentially methylated promoters. The ordinate axis represents the negative log10 of the unadjusted p-value of methylation mean differences between “severe” and control “mild” groups while the abscissa axis is the location of differentially methylated points in relation to chromosomes.

Relative treemaps on top 100 ranked promoters are provided as **Figure S14** and **S15** for hyper- and hypo-methylated promoters, respectively.


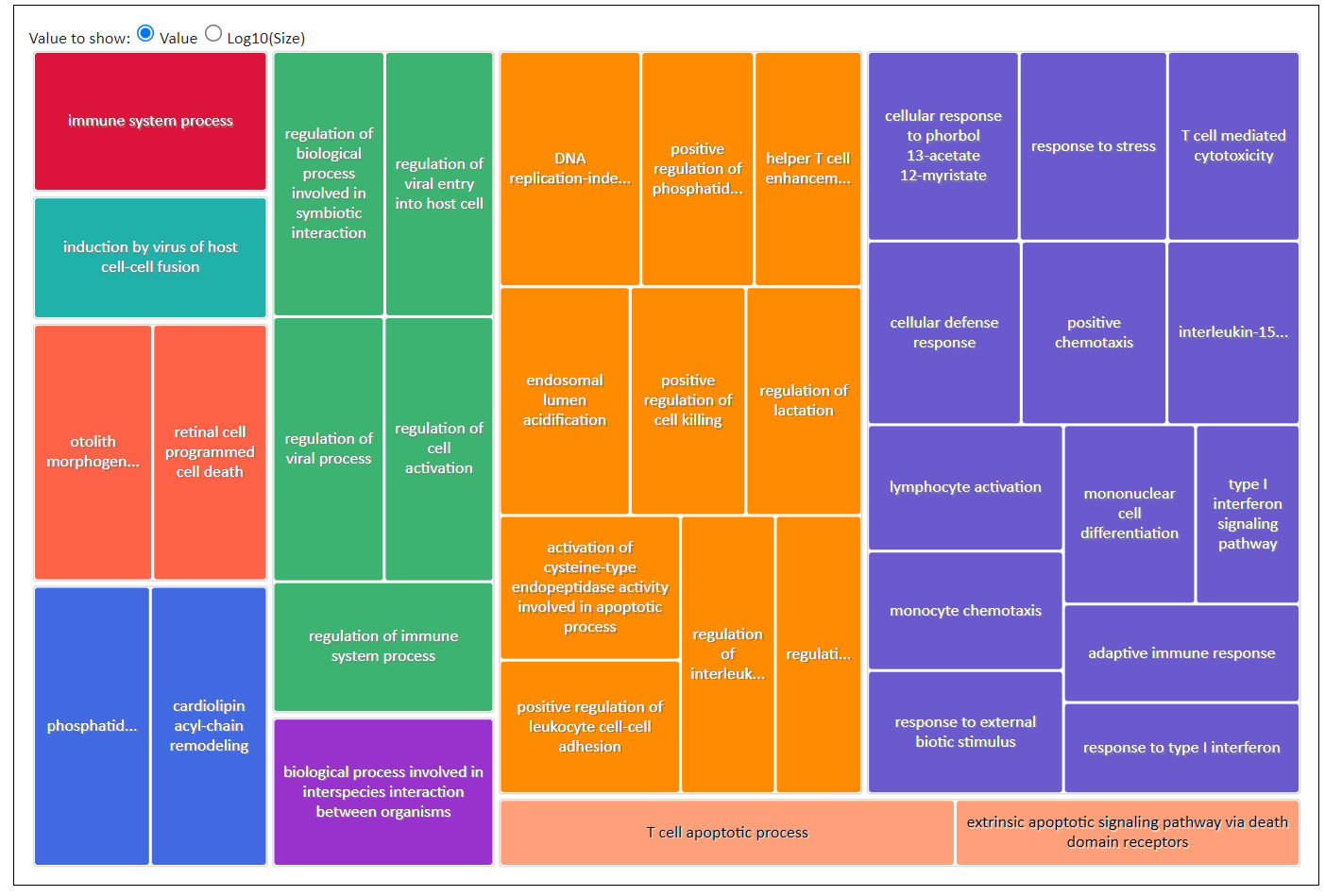


**Figure S14:** REVIGO treemap summarizing Gene Ontology (GO) enrichment analysis on promoters enriched in hyper-methylated sites.


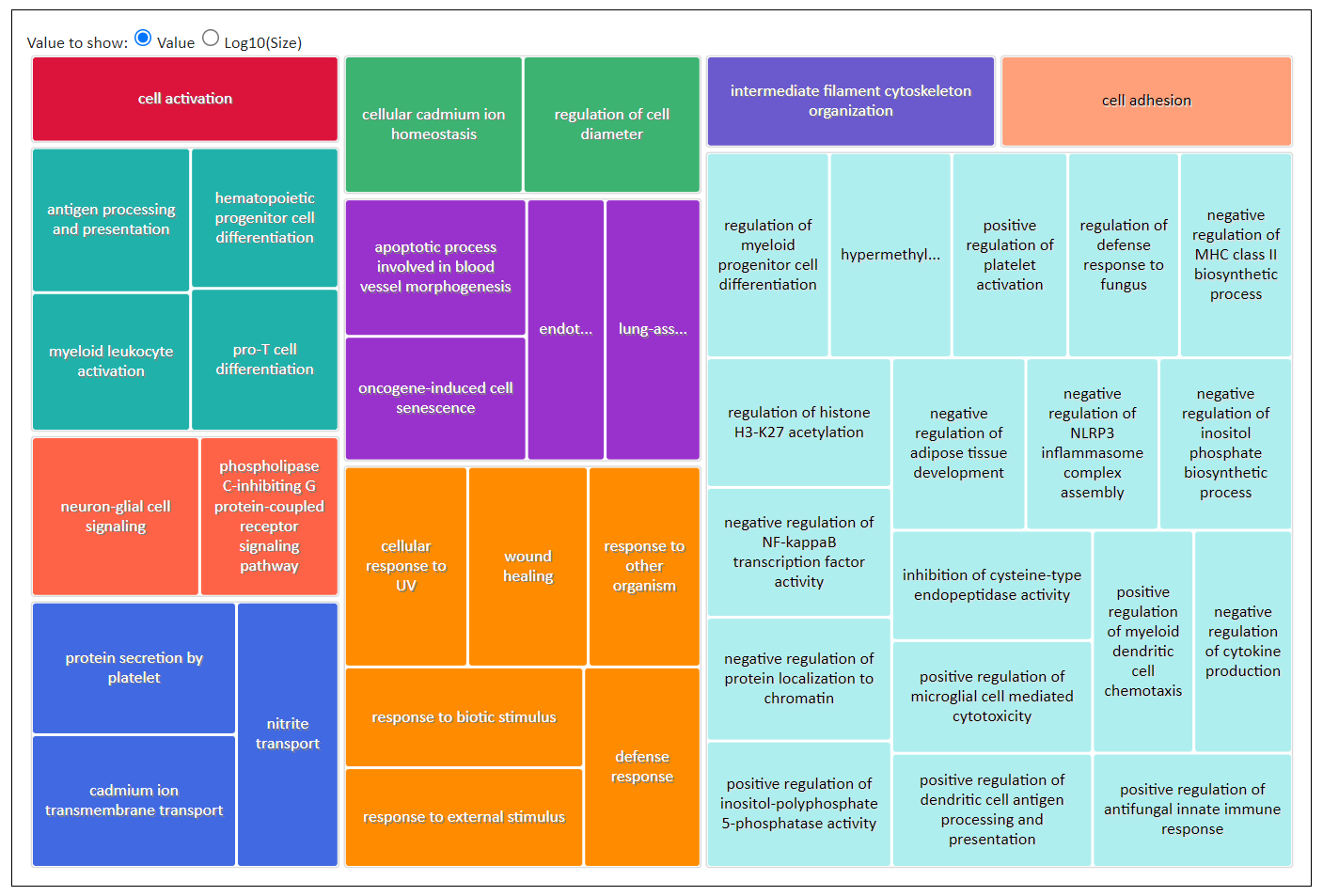


**Figure S15:** REVIGO treemap summarizing Gene Ontology (GO) enrichment analysis on promoters enriched in hypo-methylated sites.

**4)Validation of the 21 CpG sites' episignature**

1. ***Cohort of Covid19- subjects***

To validate the '21 CpG' epi-signature, we used a cohort of subjects negative for Covid-19. Pre-pandemic blood samples from 75 gender- and age-matched subjects were analyzed. The age distribution between the groups was very similar, with no significant differences observed (**Figure S16**):

**
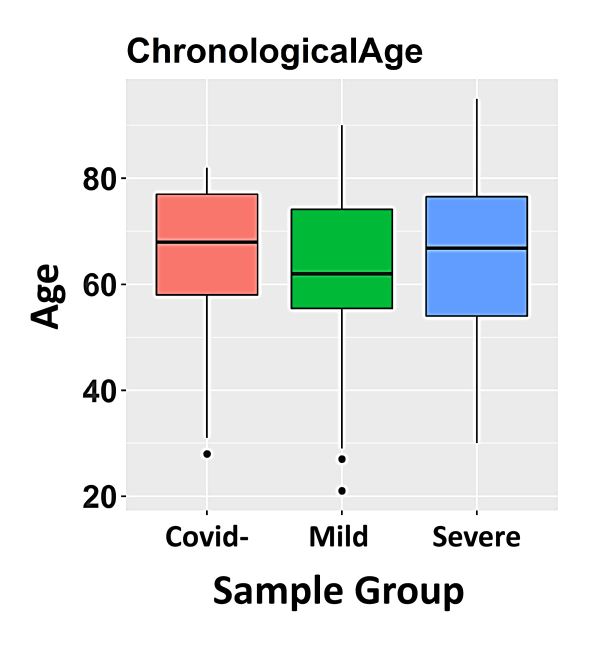
**

**Figure S16:** Boxplot showing the distribution of chronological age in Covid19-, Covid19+ mild and Covid19+ severe patients.

The thick horizontal line in the box represents the median of the distribution while the box represents the interquartile range. Whiskers are set as the default option for the “ggplot” boxplot function and extend to the most extreme data point, which is no more than 1.5 times the interquartile range from the box. Dots represent outliers (single values exceeding 1.5 interquartile ranges).

No differences between the three groups were detected by considering the methylation status of all CpG sites (PCA analysis - **Figure S17**).


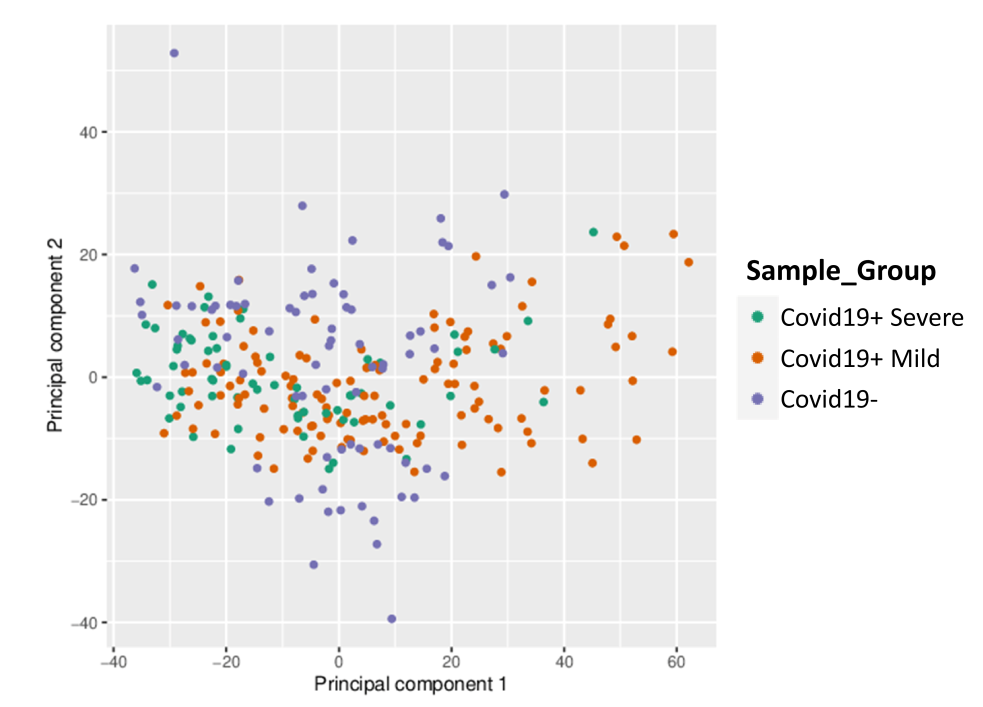


**Figure S17:** Scatter plot of principal component analysis (PCA). Scatter plot distribution of samples along with the first two principal components at site level.

The results of the PCA analysis restricted to the 21 CpG epi-signature indicates that the Covid- negative group had a DNA methylation profile that was similar to the Covid19+ Mild cohort. (**Figure S18**).


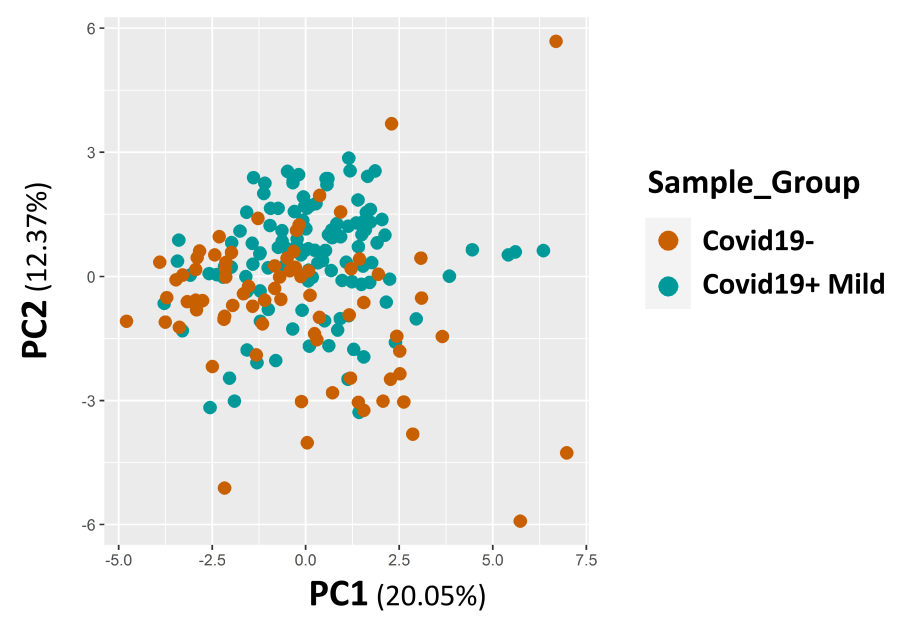


**Figure S18**: Scatter plot distribution of the methylation profiles of Covid19 negative patients vs Covid19+ Mild samples restricted to the 21 CpG sites constituting our Covid19 episignature.

On the contrary, the “21 CpG” epi-signature is efficiently able to discriminate Covid19+ Severe patients from Covid19 negative individuals (**Figure S19**).


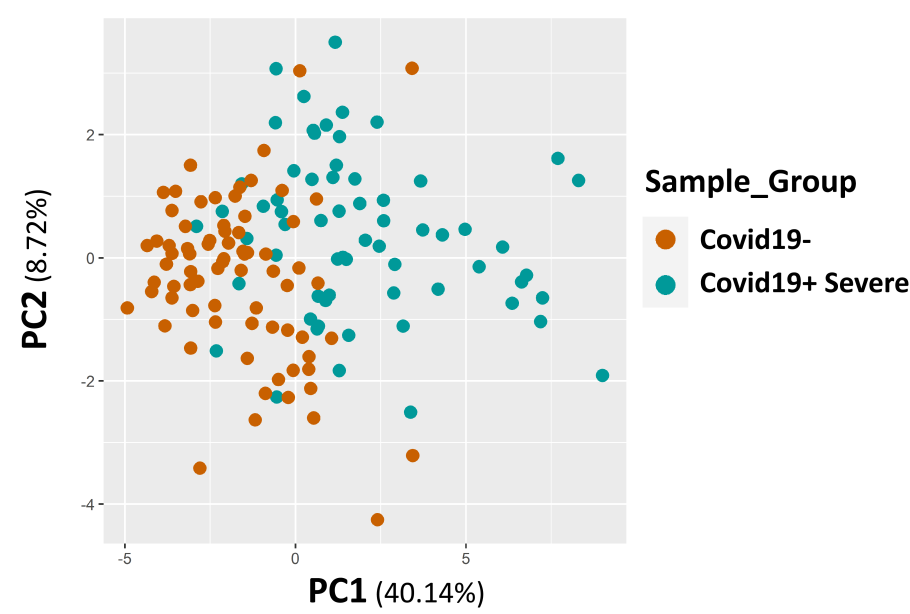


**Figure S19**: Scatter plot distribution of the methylation profiles of Covid19 negative patients vs Covid19+ Severe samples restricted to the 21 CpG sites constituting our Covid19 epi-signature.

Similar to what has been done with our (Mild vs Severe) dataset, we evaluated PC1 as a biomarker of COVID-19 evolution by testing PC1 scores vs Sample_group status by logistic regression (Covid19+ Mild vs Covid19 Negative: OR p-value= ns; Covid19+ Severe vs Covid19 Negative: OR=2.3 (95% CI:1.78-3.17).

1. ***GSE168739***

The first dataset, GSE168739 (Castro de Moura et al, 2021), includes 407 patients who were either non-hospitalized with mild symptoms or hospitalized with varying levels of respiratory support needs. The authors of this study identified a set of 44 differentially methylated CpG sites, known as the EPICOVID signature. It was not possible to re-analyze the raw data, so we performed a validation by comparing and intersecting the lists of differentially methylated CpG sites from this study (EPICOVID) with our own list of 21 CpG sites. As a quality control step, we checked for the presence of these sites in our complete methylation dataset and found that 17 were not present. After intersecting the remaining 27 CpG sites from the EPICOVID signature with our list of 21 CpG sites, we did not find any shared positions.

1. ***GSE167202***

The second series GSE167202 (Konigsberg et al, 2021) includes 525 methylation profiles comprising 164 individuals who tested positive for SARS-CoV-2 (including both mild and severe cases), 296 healthy individuals (SARS-CoV-2-Negative), and 65 patients with other respiratory infections. The authors used a machine learning approach for multiple group comparisons. According to our classification method, we divided the 164 COVID-19 patients into two groups of 48 severe and 115 mild individuals (1 sample was discarded due to incomplete information about the clinical evolution of the disease). The matrix of β-values was generated by using the relative RnBeads module. Signal intensities were normalized using the BMIQ (Beta MIxture Quantile) normalization method. Quality control step discarded 200320 probes:

1. SNP-enriched probes (139721 sites),
2. cross-reactive probes (34264),
3. unreliable measurements (Greedycut algorithm) (8963 sites),
4. context-specific (1018 sites),
5. on sex chromosomes probes (16159 sites),
6. with many missing values (195 sites).

As an outcome of the filtering procedures, 666575 CpG sites and 163 samples were retained. To test the efficacy of the 21 CpG epi-signature, a PCA was carried out: as shown in **Figure S15**, the epi-signature is efficiently able to discriminate between the two cohorts along PC1.


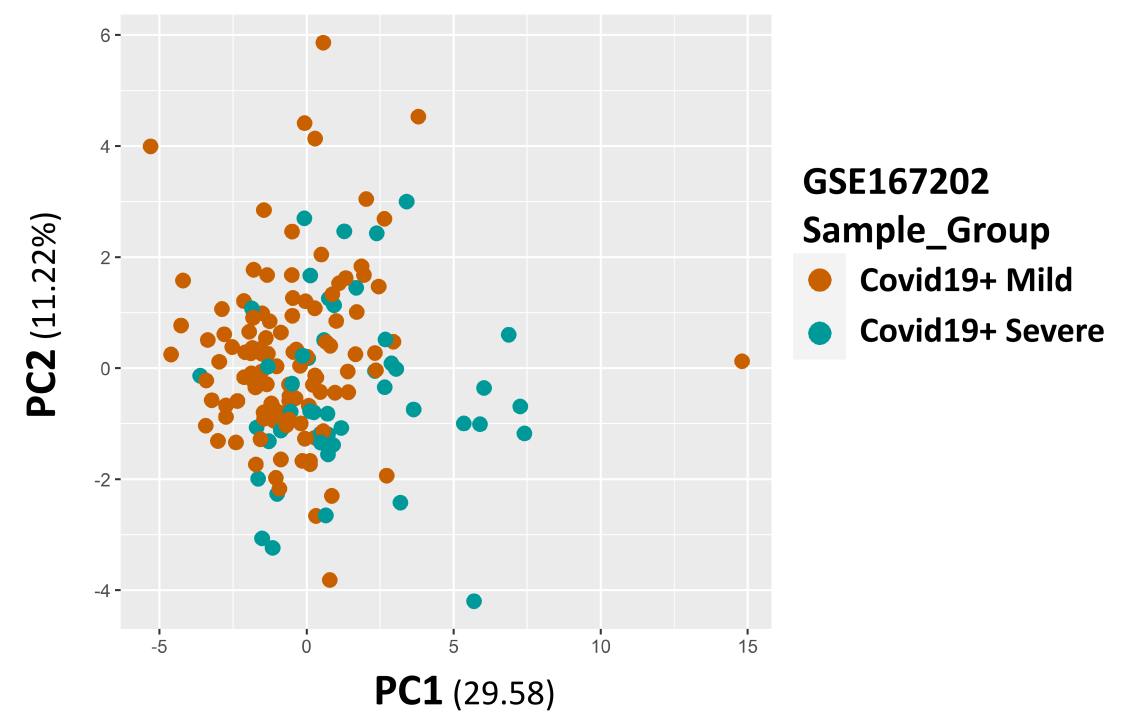


**Figure S15**: Scatter plot distribution of the methylation profiles of 163 samples (GSE167202) restricted to the 21 CpG sites constituting our covid signature.

Similar to what has been done with our dataset, we evaluated PC1 as a biomarker of COVID-19 evolution by testing PC1 scores vs Sample_group status by logistic regression. The analyses yielded an odds ratio of: OR=1.4 (95% CI:1.19-1.68).

1. ***GSE174818***

The third series GSE174818 (Balnis et al, 2021) is composed of 128 methylation profiles of both healthy (collected in the pre-covid era) (n=26) and COVID-19 (n=102) subjects. The authors performed various sub-group comparisons but when they used the GRAM-risk score as a parameter to discriminate patients with a severe COVID-19 evolution, the analysis led to the identification of 77 differentially methylated sites. Similar to what was done previously, we firstly ran another quality control check (68 CpG retained) and intersected the two lists: likewise, no sharing was observed. The available clinical phenotypes allowed us to constitute severe and mild cohorts by adopting our classification criterion. Since two raw data referred to as GSM5332004 and GSM5332115 were discarded due to ambiguous anagraphical/clinical assignment, the final groups were as follows: severe (n=55) and mild (n=45). Then we extracted from the GSE174818 methylation dataset the methylation profiles of our 21 episignature list. The matrix of β-values was generated by using the relative RnBeads module. Signal intensities were normalized using the BMIQ (Beta MIxture Quantile) normalization method. Quality control module discarded 194852 probes:

1. SNP-enriched probes (139721 sites),
2. cross-reactive probes (34264),
3. unreliable measurements (greedycut algorithm) (3165 sites),
4. context-specific (1180 sites),
5. on sex chromosomes probes (16378 sites),
6. with many missing values (144 sites).

As a final outcome of the filtering procedures, 672043 CpG sites and 100 samples were retained. To test the efficacy of the 21 CpG episignature a Principal Component Analysis was carried out: as shown in figure **Figure S16**, the signature is efficiently able to discriminate the two cohorts along PC1.


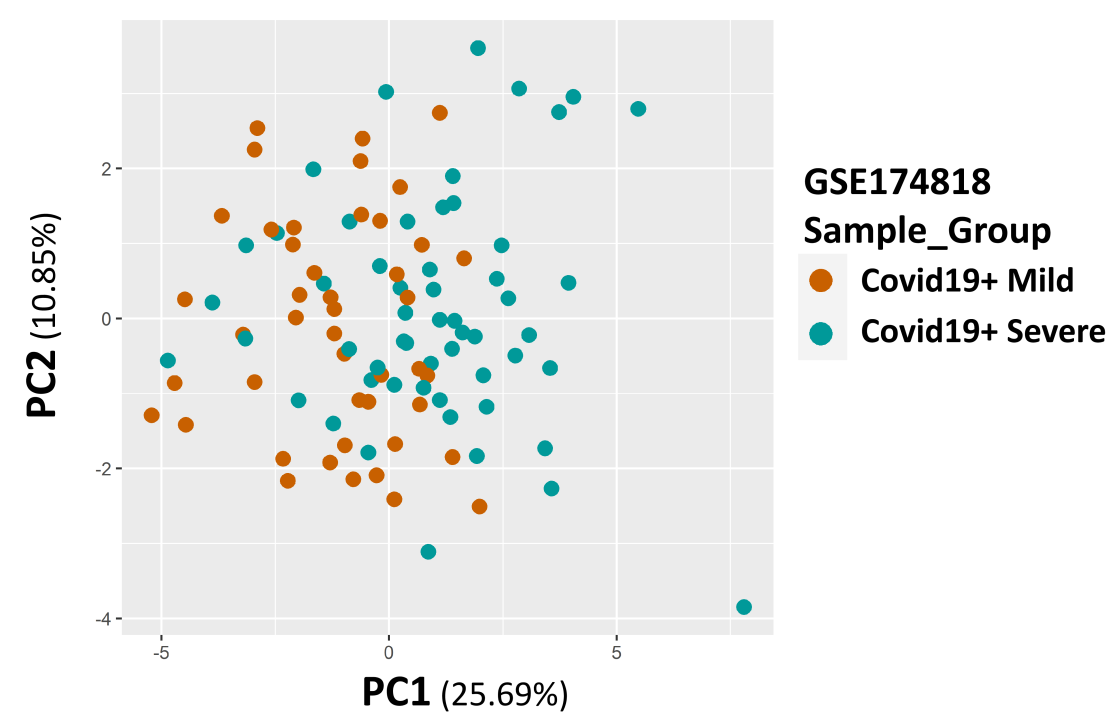


**Figure S16**: Scatter plot distribution of the methylation profiles of 100 samples (GSE174818) restricted to the 21 CpG sites constituting our covid19 episignature.

Similar to what has been done with previous datasets, PC1 was evaluated as a biomarker of COVID-19 evolution by testing PC1 scores vs Sample_group status by logistic regression. The analyses yielded an odds ratio of: OR=1.69 (95% CI:1.34-2.23).

**6) Stochastic Epigenetic Mutations (SEMs)**

The sample group comparison (severe vs mild) was carried out by considering the hyper- and hypo-methylated lists individually (obtained from the lists of total SEMs). Although a well-defined difference trend is still visible, statistical differences were mainly found in hypo-methylated lists (**Figures S17)**.


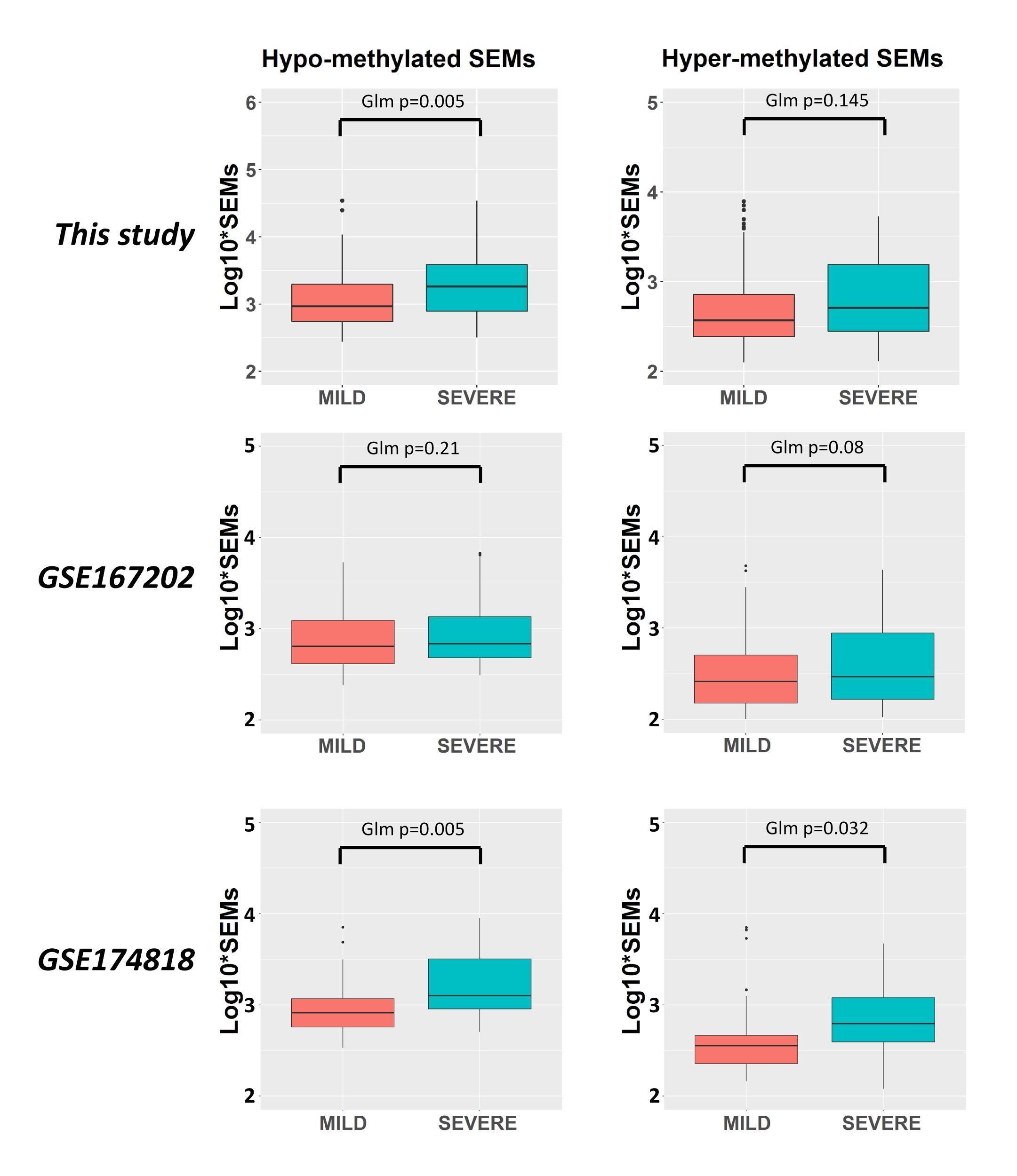


**Figure S17:** Boxplots of hypo- (left panel) and hyper-methylated (right panels) SEMs, obtained from the three datasets.

**References**

Mootha VK, Lindgren CM, Eriksson KF, Subramanian A, Sihag S, Lehar J, et al. PGC-1alpha-responsive genes involved in oxidative phosphorylation are coordinately downregulated in human diabetes. Nature genetics. 2003;34(3):267-73.

Subramanian A, Tamayo P, Mootha VK, Mukherjee S, Ebert BL, Gillette MA, et al. Gene set enrichment analysis: a knowledge-based approach for interpreting genome-wide expression profiles. Proc Natl Acad Sci U S A. 2005;102(43):15545-50.

Supek F, Bosnjak M, Skunca N, Smuc T. REVIGO summarizes and visualizes long lists of gene ontology terms. PLoS One. 2011;6(7):e21800.
